# 40th International Symposium on Intensive Care & Emergency Medicine 2021

**DOI:** 10.1186/s13054-021-03769-1

**Published:** 2021-11-15

## P001

### AGTR1 rs275651 AA genotype links with better survival in sepsis patients in ICU

#### A. Chumachenko, V. Pisarev, E. Grigoryev

##### Federal Research and Clinical Center of Intensive Care Medicine and Rehabilitology, V. A. Negovsky’ Research Institute of General Reanimatology, Moscow, Russian Federation

*Critical Care* 2021, **25**(**Suppl 1**): P001

**Introduction**: International studies demonstrate an annual increase in the frequency and impact of sepsis in intensive care units (ICU). Dysregulation of blood pressure makes a significant contribution during sepsis, especially septic shock. Angiotensin II receptor type 1 (AGTR1) affects the condition of the vessel wall and arteriolar tone. The AGTR1 gene encodes angiotensin II receptor type 1, which is involved in cardiovascular diseases. AGTR1 rs275651 AA genotype is associated with unstable angina. So, the aim of our study was to define the contribution of AGTR1 rs275651 genotypes to the course and outcomes of critical illness, complicated with sepsis.

**Methods**: Study cohort included 145 ICU patients diagnosed with sepsis. 60% of patients underwent surgery, and 42% were diagnosed with diabetes as a major comorbidity. AGTR1 rs275651 polymorphism was studied by using specific polynucleotide tetraprimer set to amplify gene fragments from each patient’s DNA followed by analysis of PCR products in a 2% agarose gel electrophoresis.

**Results**: Septic shock occurred more frequently in patients with AT or TT genotype, than in homozygotic carriers of AGTRI A (p = 0.01, Fisher's exact test). Also, patients with more common genotype AA and with sepsis often survived (p = 0.039, Fisher`s exact test, Fig. 1). SOFA values on days 1 were not different in patients of distinct AGTR1 genotypes. Expectedly SOFA values on day 2 and middle day in ICU were significantly lower in patients AGTR1 AA genotype compared to T carriers of (6.8 vs. 7.9, p = 0.04, t test and 6.9 vs. 8.4, p = 0.024, respectively).

**Conclusions**: AGTR1 rs275651 AA genotype associates with survival advantage in sepsis patients in ICU setting presumably because of contribution to lower multiorgan failure and decreased occurrence of septic shock.

**Figure:**
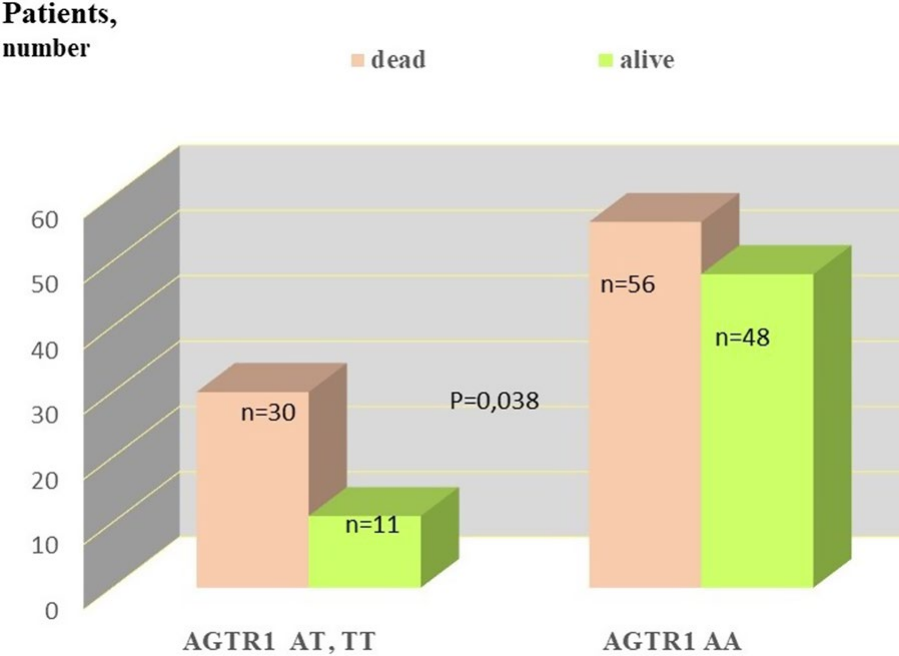
**Fig. 1** **(abstract P001)** Survival of sepsis patients depends on AGTRI rs275651 genotypes

## P002

### Effects of xenon on proinflammatory activation and apoptosis of human neutrophils (ex vivo study)

#### R. A. Cherpakov, A. V. Ershov, O. A. Grebenchikov, V. V. Antonova, A. N. Kuzovlev

##### Federal research and clinical center of intensive care medicine and rehabilitology, Moscow, Russian Federation

*Critical Care* 2021, **25**(**Suppl 1**): P002

**Introduction**: The aim of the study was to evaluate the effect of xenon on the activation of human neutrophils in ex vivo conditions.

**Methods**: After receiving informed consent, 10 healthy volunteers had their blood drawn twice: before xenon inhalation (xenon - 30 vol.%, oxygen - no more than 40 vol.%, the rest - nitrogen) and immediately after. The duration of inhalation in all patients was 60 min. After neutrophil isolation from the patient’s serum, lipopolysaccharide (LPS) was added to the 4 million/ml cell concentrate at a dose of 200 ng/ml. The effect of xenon on the severity of inflammatory activation of neutrophils was assessed by the level of expression of CD11b and CD66b adhesion molecules on their surface and phosphorylation of pro-inflammatory kinases: ERK1/2 and kinase-p38.

**Results**: The addition of lipopolysaccharide to the neutrophil incubation medium caused their activation, significantly increasing the phosphorylation of the key pro-inflammatory kinases of neutrophils: ERK1/2 and kinase-p38. After xenon anesthesia, there was a decrease in the expression of CD11b and CD66b adhesion molecules on the neutrophil surface and reducing the phosphorylation (activation) of pro-inflammatory kinases: ERK1/2 and MAP-kinase p38, which demonstrated its anti-inflammatory effect. The addition of LPS to the neutrophil incubation medium reduced their ability to spontaneous apoptosis 22 h after isolation, which was 22.6%, which was 60% less than in the control group-56.3% (p < 0.05). Xenon inhalation significantly increased to 41.35% (p < 0.05) the ability of neutrophils to spontaneous apoptosis after incubation with LPS.

**Conclusions**: Inhalation of xenon 30 vol% for 60 min has a pronounced anti-inflammatory effect on neutrophils, reducing their activation by inhibiting pro-inflammatory kinases: ERK1/2 and MAP-kinase p38, reducing the expression of activation markers CD11b and CD66b on the surface of neutrophils and increasing their ability to spontaneous apoptosis.

## P003

### The systemic inflammatory response induced by lipopolysaccharide administration is more pronounced in women than in men

#### A. Jansen, N. Bruse, N. Waalders, P. Pickkers, M. Kox

##### Department of Intensive Care Medicine, Radboud University Medical Center, Nijmegen, Netherlands

*Critical Care* 2021, **25**(**Suppl 1**): P003

**Introduction**: A better understanding of the potential sex-specific differences in the immune response may facilitate personalized treatment approaches for sepsis. We investigated whether sex affects the immune response and the development of endotoxin tolerance in a large cohort of volunteers undergoing repeated experimental human endotoxemia, an established in vivo model capturing many hallmarks of both early sepsis and sepsis-induced immunoparalysis.

**Methods**: Subjects (54 females and 56 males) were intravenously challenged with 1 ng/kg bacterial lipopolysaccharide (LPS) twice: on day 0 to determine the extent of the inflammatory response and again on day 7 to determine the degree of endotoxin tolerance. Area under the plasma cytokine time-concentration curves (AUCs) were calculated to provide an integral measure of the cytokine response.

**Results**: Median [interquartile range] age was 23 [21–25] years for males and 23 [21–24] years for females (p = 0.18), whereas BMI was 23.0 [20.8–25.1] and 23.6 [21.9–25.7] kg/m^2^, respectively (p = 0.12). Compared with males upon the first LPS challenge, females produced significantly higher levels of tumour necrosis factor (TNF, 41% higher AUC), interleukin (IL)-6 (+ 50%), interferon gamma -nduced protein (IP)-10 (+ 47%), and IL-1 receptor antagonist (+ 112%), but not IL-10 (-4%, Fig. 1). Although a tolerant response was observed for all measured cytokines (all p < 0.0001 vs. first challenge), no differences in the degree of endotoxin tolerance between the sexes were observed.

**Conclusions**: We demonstrate that females mount a more pronounced proinflammatory cytokine response following LPS administration than males, while levels of the anti-inflammatory cytokine IL-10 and the development of endotoxin tolerance were not different between the sexes. These findings indicate sex-specific regulation of the innate immune response. Sex hormone profiles are currently being determined to assess whether these differences have a hormonal origin.

**Figure:**
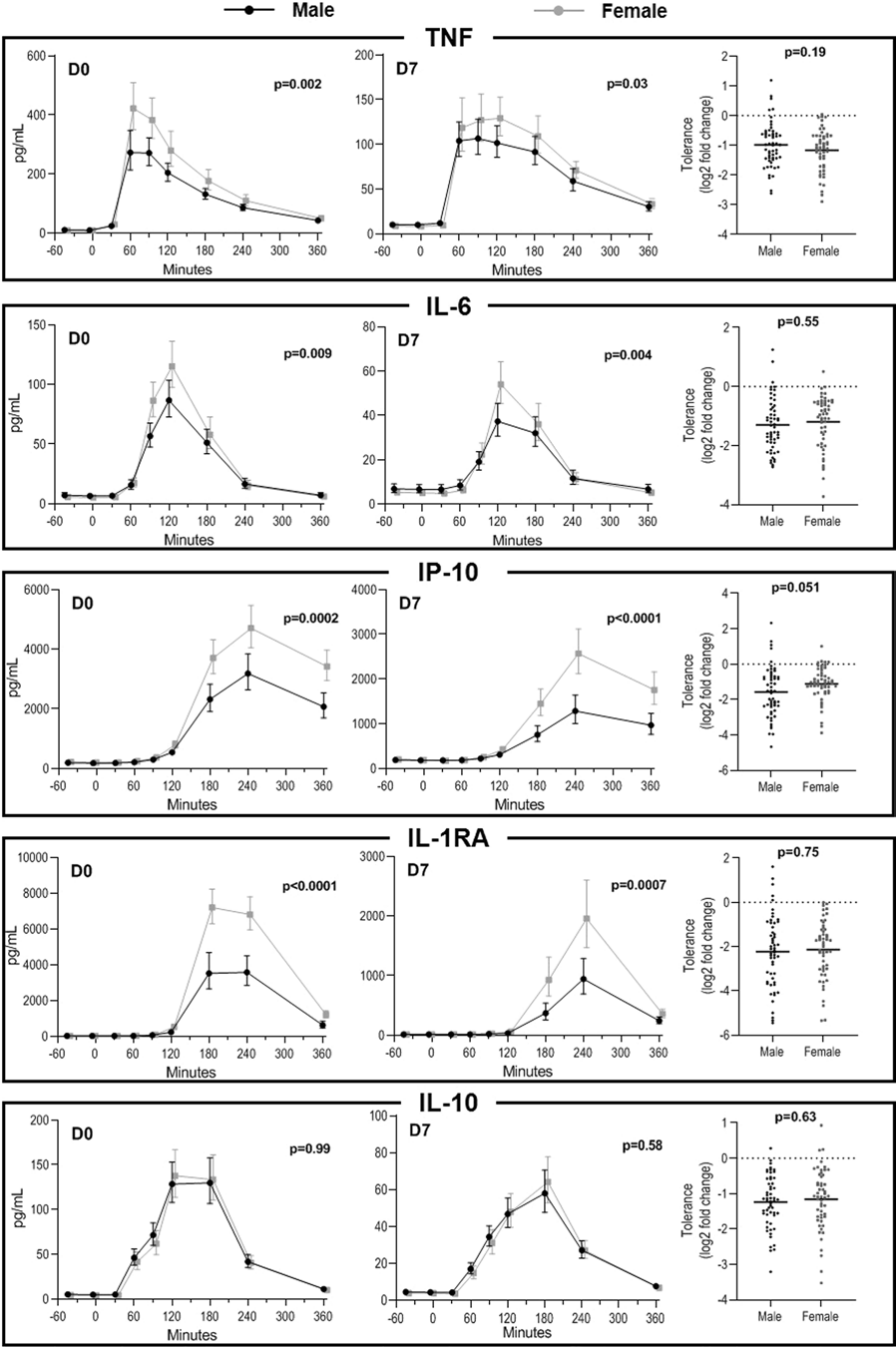
**Fig. 1** **(abstract P003)** Sex-specific differences in plasma levels of inflammatory mediators following repeated LPS administration on day 0 (D0, left panels) and day 7 (D7, center panels). On both days, 1 ng/kg LPS was administered at t = 0. Right panels display the degree of endotoxin tolerance, presented as the log2 fold change in the area under the time-concentrations curves between day 7 and day 0. Data in left and center panels are presented as geometric mean and 95%-confidence intervals, whereas data in the right panels are presented as scatter plots with the horizontal line indicating the mean. P-values were calculated using unpaired student’s t-test on log-transformed area under the time-concentrations curve data. TNF = tumor necrosis factor, IL = interleukin, IP = interferon gamma-induced protein, RA = receptor antagonist

## P004

### No interplay between gut microbiota composition and the lipopolysaccharide-induced innate immune response in humans in vivo

#### Q. Habes, M. Kox, P. Pickkers

##### Intensive Care, Radboud University Medical Center, Nijmegen, Netherlands

*Critical Care* 2021, **25**(**Suppl 1**): P004

**Introduction**: Animal studies have demonstrated the extensive interplay between the gut microbiota and immunity. Moreover, in critically ill patients, who almost invariably suffer from a pronounced immune response, a shift in gut microbiota composition is associated with infectious complications and mortality. We examined the relationship between interindividual differences in gut microbiota composition and variation in the in vivo cytokine response induced by bacterial lipopolysaccharide (LPS). Furthermore, we evaluated whether an LPS challenge alters the composition of the gut microbiota.

**Methods**: Healthy male volunteers received an intravenous bolus of 2 ng kg^−1^ LPS (n = 70) or placebo (n = 8). Serial plasma concentrations of tumor necrosis factor-α, interleukin (IL)-6, IL-8 and IL-10 were measured, and subjects were divided into high and low cytokine responders. Gut microbiota composition was determined using 16 s RNA gene sequencing of fecal samples obtained 1 day before (baseline) and 1 day and 7 days following the LPS challenge.

**Results**: Baseline microbiota composition, analyzed by principal coordinate analysis and random forest analysis, did not differ between high and low responders for any of the four measured cytokines. Furthermore, baseline microbiota diversity (Shannon and Chao indices) was similar in high and low responders. No changes in microbiota composition or diversity were observed at 1 and 7 days following the LPS challenge (Fig. 1).

**Conclusions**: Our results indicate that existing variation in gut microbiota composition does not explain the observed variability in the LPS-induced innate immune response. These findings strongly argue against the interplay between the gut microbiota composition and the innate immune response in humans.

**Figure:**
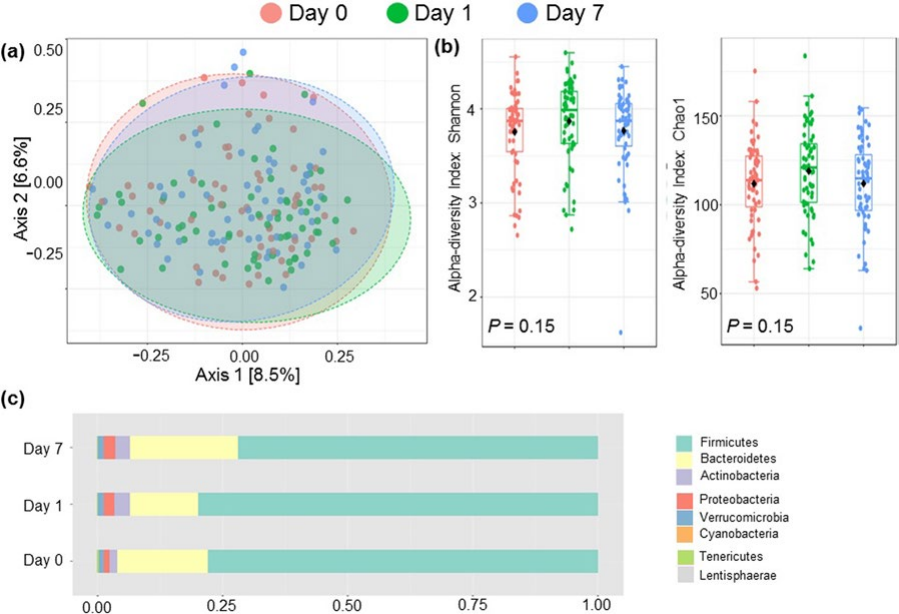
**Fig. 1** **(abstract P004)** Effect of LPS challenge on gut microbiota composition. a) Principal coordinates analysis (PCoA) of microbiota composition over time following LPS challenge (n = 63, paired samples across timepoints). PERMANOVA on the Bray–Curtis dissimilarity index: F-value: 0.44, R-squared = 0.01, p = 1.00. b) Gut microbiota diversity over time following LPS challenge (n = 63, paired samples across timepoints), reflected by Shannon index and Chao1 index. p values calculated by Friedman tests. c) Relative abundance of gut microbiota (phylum level) over time following LPS challenge (n = 63, paired samples across timepoints)

## P005

Withdrawn

## P006

### Body mass index and mortality in COVID-19 and other diseases: a cohort study in 35,506 ICU patients

#### E. J. Kooistra^1^, S. Brinkman^2^, P. H. Van der Voort^3^, N. F. De Keizer^2^, D. A. Dongelmans^4^, M Kox^1^, P. Pickkers^1^

##### ^1^Department of Intensive Care Medicine, Radboud University Medical Center, Nijmegen, Netherlands; ^2^National Intensive Care Evaluation (NICE) Foundation, Amsterdam UMC, Amsterdam, The Netherlands; ^3^Department of Critical Care, University Medical Center Groningen, University of Groningen, Groningen, Netherlands, ^4^Department of Critical Care, Amsterdam University Medical Center, Amsterdam, The Netherlands

*Critical Care* 2021, **25**(**Suppl 1**): P006

**Introduction**: Obesity is a risk factor for severe coronavirus disease 2019 (COVID-19) and might play a role in its pathophysiology. It is, however, unknown whether BMI is related to clinical outcome following intensive care unit (ICU) admission, as observed in various other categories of critically ill patients. We investigated the relationship between body mass index (BMI) and in-hospital mortality in critically ill COVID-19 patients and in cohorts of ICU patients with non-SARS-CoV-2 viral pneumonia, bacterial pneumonia, and multiple trauma.

**Methods**: We performed a multicenter observational cohort study using data from 35.506 consecutive patients from 82 ICUs participating in the Dutch National Intensive Care Evaluation quality registry. Patient characteristics and clinical outcomes were compared between four cohorts (COVID-19, non-SARS-CoV-2 viral pneumonia, bacterial pneumonia, and trauma) and between BMI categories within cohorts. Adjusted analyses of the relationship between BMI and in-hospital mortality within each cohort were performed using multivariable logistic regression.

**Results**: COVID-19 patients were more likely male, had a higher BMI, lower PaO_2_/FiO_2_ ratio, and were more likely mechanically ventilated during the first 24 h in the ICU compared to the other three cohorts. COVID-19 patients had longer ICU and hospital length of stay, and higher in-hospital mortality. Odds ratios for in-hospital mortality for patients with BMI ≥ 35 kg/m^2^ compared with normal weight in the COVID-19, non-SARS-CoV-2 viral pneumonia, bacterial pneumonia, and trauma cohorts were 1.15 [0.79–1.67], 0.64 [0.43–0.95], 0.73 [0.61–0.87] and 0.81 [0.57–1.15], respectively (Table 1).

**Conclusions**: The obesity paradox, which is the inverse association between BMI and mortality in critically ill patients, is not present in ICU patients with COVID-19-related respiratory failure, in contrast to ICU patients suffering from non-SARS-CoV-2 viral and bacterial respiratory infections.

**Table:** **Table 1** **(abstract P006)** Odds ratios of in-hospital mortality of BMI categories in the multivariate logistic regression model, with BMI 18.5–25 kg/m^2^ used as reference category

	**COVID-19 (n = 2635)**	**non-SARS-CoV-2 viral pneumonia (n = 2940)**	**Bacterial pneumonia (n = 14,250)**	**Trauma (n = 15,681)**
BMI < 18.5 kg/m^2^	1.92 [0.51–7.13]	1.50 [0.95–2.37]	1.88 [1.57–2.25]	1.23 [0.86–1.78]
BMI 18.5–25 kg/m^2^	1.0 reference	1.0 reference	1.0 reference	1.0 reference
BMI 25–30 kg/m^2^	0.95 [0.75–1.21]	0.78 [0.61–0.99]	0.78 [0.70–0.86]	0.90 [0.78–1.03]
BMI 30–35 kg/m^2^	0.87 [0.65–1.16]	0.76 [0.55–1.04]	0.81 [0.70–0.93]	0.99 [0.79–1.23]
BMI ≥ 35 kg/m^2^	1.15 [0.79–1.67]	0.64 [0.43–0.95]	0.73 [0.61–0.87]	0.81 [0.57–1.15]

Covariates used for this analyses included sex, age, medical history (chronic diagnoses), APACHE III acute physiology score (APS), vasoactive medication and mechanical ventilation and PaO_2_/FiO_2_ ratio on ICU admission.

## P007

### NO in COVID-19 patient: beyond vasodilation

#### M. Zanzotti^1^, L. Gottin^2^, E. Polati^1^, K. Donadello^1^

##### ^1^U.O.C. Anestesia e Rianimazione B, AOUI Verona, Verona, Italy; ^2^USD Anestesia e Terapia Intensiva in Chirurgia Cardiaca e Toracica, AOUI Verona, Verona, Italy

*Critical Care* 2021, **25**(**Suppl 1**): P007

**Introduction**: NO is involved not only in the regulation of vascular tone, but also in the inhibition of endothelial adhesion of leukocytes and inhibits inflammatory processes by reducing the synthesis of cytokines by NF-κB [1, 2]. The aim of our work was to evaluate whether in patients with COVID-19 ARDS and treated with iNO this reduction of local inflammatory damage results in a concomitant reduction of systemic inflammatory indices.

**Methods**: In this retrospective observational study, were included all patients who in the period from March 2020 to April 2021 were treated with iNO for at least 7 days. For each patient, the values of: P/F, CRP, leukocytes, PCT, ferritin, fibrinogen and D-dimer were recorded before the start of therapy with iNO and subsequently on the first, third and seventh day. Data were compared with Student's t test and p < 0.05 were considered statistically significant.

**Results**: Twenty-four patients were enrolled in the study period, with an average age of 62 years. Table 1 shows the data collected. As can be seen, the improvement in the P/F ratio was immediate and has been maintained over time (pre-iNO: 95, G7: 167). A statistically significant reduction in CRP (pre-iNO: 129 mg/l, G7: 73 mg/l) and fibrinogen (pre-iNO: 6.08 g/l, G7: 4.76 g/l) was observed at three and seven days, at seven days of D-dimer (pre-iNO: 1.52 mg/l, G7: 1.1 mg/l). Ferritin showed a decreasing trend compared to baseline, reaching significance only on the third day. GB and PCT, on the other hand, did not show significant reductions.

**Conclusions**: This study, while taking into account the limitation of the sample size and the absence of a control group, allowed to highlight a correlation between the use of nitric oxide and a reduction in systemic inflammation indices. Larger studies with control groups are needed to confirm these preliminary data.

**References**

1. El Kebir D et al. Can J Physiol Pharmacol 83:252–8, 2005
2. Sun Z et al. Inflamm Res 55:430–40, 2006

**Table:** **Table 1** **(abstract P007)** Data collected

	**pre-iNO**	**D1**	**D3**	**D7**
P/F ratio	95	151 p < 0.001	168 p < 0.001	167 p < 0.001
CPR (mg/l)	129	109 p = 0.098	79 p = 0.004	73 p = 0.001
WBC (10^9^/l)	11.72	12.14 p = 0.292	10.82 p = 0.134	12.39 p = 0.284
PCT (mcg/l)	7.79	2.29 p = 0.085	0.84 p = 0.070	0.63 p = 0.073
Ferritin (mcg/l)	2191	1931 p = 0.120	1564 p = 0.040	1590 p = 0.060
Fibrinogen (g/l)	6.08	5.88 p = 0.235	5.27 p = 0.043	4.76 p = 0.002
D-dimer (mg/l)	1.52	1.09 p = 0.066	1.20 p = 0.055	1.10 p = 0.047

## P008

### Prognostic factors and predictive markers of early and delayed mortality in patients with sepsis

#### M. Babaev^1^, N. Kostrica^2^, M. Petrushin^3^

##### ^1^Intensive Care Unit, Petrovsky National Research Centre of Surgery, Moscow, Russian Federation; ^2^Faculty of Medicine, Lomonosov Moscow State University, Moscow, Russian Federation; ^3^ICU, Tver Regional Hospital, Tver, Russian Federation

*Critical Care* 2021, **25**(**Suppl 1**): P008

**Introduction**: A personalized approach in sepsis therapy is based on the study of prognostic and predictive risk factors of adverse outcomes. Our objectives were to identify prognostic factors and predictive markers of early and delayed mortality within the first 72 h of hospital stay.

**Methods**: This retrospective study included 136 patients with sepsis (according to the SEPSIS-3 criteria) of pulmonary (37%) and abdominal (63%) etiology at the age from 19 to 81. The anamnestic, clinical and laboratory data were collected. Patients received therapy according to the accepted recommendations. Early mortality was assessed in two time intervals: within the first 72 h of admission and between 4th and 7th day of admission. Delayed mortality was registered after 8th and 15th day. The SPSS program was used for statistical processing.

**Results**: The overall mortality rate was 61%. Within the first 72 h, predictors of deaths (13% of cases) were SOFA score of 8 or more (OR 1.272, p = 0.012, AUC 0.648); lactate level 2 ng/ml or more (OR 1.556, p = 0.025, AUC 0.704); arterial blood pH level 7.22 or less (OR 1.245, p = 0.005, AUC 0.704). Mortality in the period of 4–7 days (18%) was significantly influenced by the level of procalcitonin more than 9 ng/ml on admission (OR 1.199, p = 0.028, AUC 0.604). The prognostic mortality factor for all time periods was the Charlson comorbidity index of more than 4 points (OR 1.515, p = 0.001, AUC 0.721) for early mortality and 3 points (OR 1.498; p = 0.013, AUC 0.666) for delayed mortality (33%).

**Conclusions**: Patients with multiple chronic diseases, severe organ disorders and high microbial load, represent the most vulnerable group in terms of early unfavorable outcomes of the course of sepsis and, apparently, require an immediate start of complex targeted sepsis therapy.

## P009

### Combination of 2B4 and CD28 on T lymphocytes predicts poor prognosis in sepsis patients: a prospective observational study

#### Q. Liu^1^, J. Xie^2^, Y. Yang^2^

##### ^1^Critical Care Medicine, Zhongda Hospital, Nanjing, China; ^2^Critical Care Medicine, Zhongda Hospital affiliated to Southeast University, Nanjing, China

*Critical Care* 2021, **25**(**Suppl 1**): P009

**Introduction**: 2B4 and CD28 are important co-signal molecules that regulate the T cell function. Regulating 2B4 and CD28 pathway was demonstrated to improve sepsis mortality in animal studies. We aim to determine the effect of them on mortality in patients with sepsis.

**Methods**: This was a single-center, prospective observational study. Patients with sepsis who admitted to the ICU in Zhongda Hospital affiliated to Southeast University from April 2019 to December 2020 were included in this study. 2B4 and CD28 expression on CD4 + and CD8 + T cells were tested on days 1, 3 and 7 after sepsis diagnosis. The association between 2B4 and CD28 expression and mortality were analyzed.

**Results**: A total of 152 septic patients [age, M (IQR) 64 (50–71) year; 105 (69.1%) male] were included in this study. At day 30 after enrolment, 39 (25.7%) patient died. Compared with the survivors, the expression of 2B4 on CD4 + T cells was significantly higher [9.50% (4.33–15.44) vs. 6.25% (2.87–12.13) p = 0.019] while the expression of CD28 on CD4 + T cells was significantly lower [92.67% (84.74–96.11) vs. 95.26% (89.10–97.89), p = 0.031] in the non-survivors. Similarly, the expression of 2B4 on CD8 + T cells in the non-survivors was significantly higher [79.17% (58.56–87.41) vs. 61.68% (43.50–82.07), p = 0.001] in survivors. However, there was no difference of expression of CD28 on CD8 + T cells between survivors and non-survivors (p = 0.543). Multivariate logistic regression analysis revealed that APACHE II score, 2B4 expression on CD8 + T cells and BMI were associated with 30-day mortality in patients with sepsis. Kaplan–Meier survival analysis revealed that higher expression of 2B4 on CD4 + T cells and CD8 + T cells and lower expression of CD28 on CD4 + T cells were associated with higher mortality.

**Conclusions**: 2B4 and CD28 expression on T cells were associated with 30-day mortality in ICU patients with sepsis.

## P010

### Decreased survival of sepsis patients with diabetes associates with single-nucleotide polymorphism A(-777) > T of angiotensin II type 1 receptor gene AGTR1

#### V. Pisarev, A. Chumachenko, E. Grigoriev

##### Federal Research and Clinical Center of Intensive Care Medicine and Rehabilitology, V.A.Negovsky Institute of General Reanimatology, Moscow, Russian Federation

*Critical Care* 2021, **25**(**Suppl 1**): P010

**Introduction**: Dysregulation of blood pressure significantly impacts the course of sepsis, especially septic shock. The AGTR1 gene encodes angiotensin II receptor type 1, which affects the vascular tone and contributes to septic shock [1]. Our study aims to define whether the AGTR1 polymorphism contributes to the course and outcomes of sepsis in patients admitted to the city hospital ICU facility.

**Methods**: Study cohort included 157 ICU patients diagnosed with sepsis (SEPSIS-3, 2016); 66 patients were diagnosed with diabetes as major comorbidity. Functional AGTR1 rs275651 polymorphism [2, 3] was studied using a polynucleotide tetra primer set to amplify gene fragments followed by the analysis of PCR products by 2% agarose gel electrophoresis.

**Results**: The study revealed no differences in septic shock and lethality in patients with no diabetes differing in AGTR1 rs275651 genotypes. The septic shock occurred more frequently in patients with diabetes and minor T allele (AT and TT genotypes) than in homozygotic carriers of AGTRI AA and diabetes: p = 0.006 (Fisher test), odds ratio (OR) 11.692. There were no differences in shock during ICU hospitalization between AA genotype carriers vs. allele T carriers in a cohort of sepsis patients with no diabetes (n = 91, p = 0.8). Significant differences in lethality between diabetic sepsis patients and non-diabetic patients (Chi-square 7.698 (Yates); OR = 2.76, 95% CI: 1.389 – 5.484, p = 0.006) were due to significantly increased lethality in diabetes patients with AT or TT AGTR1 genotype: OR = 11.034, p = 0.007 by Fisher test (Fig. 1). T allele carriers with sepsis and diabetes experienced increased SOFA and CIRS values.

**Conclusions**: In sepsis, patients with diabetes minor mutation A > T within AGTR1 rs275651 associates with shock and increased lethality, vascular comorbidity, and organ failure.

**References**

1. Correa TD et al. Crit Care 19:98–103, 2015.
2. Brugts JJ et al. Eur Heart J 31:1854–1864, 2010.
3. GhafilF A. et el. Indian J Clin Biochem. 36:81–87, 2021.

**Figure:**
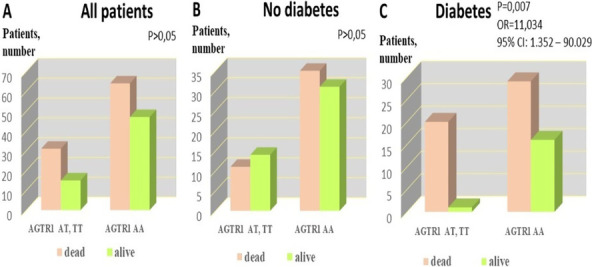
**Fig. 1** **(abstract P010)** Poor survival of patients with diabetes and sepsis associates with allele T rs275651 AGTR1

## P011

### Prognostic value of novel sepsis biomarkers in adult patients undergoing VA-ECMO for cardiocirculatory shock

#### M. Taiana^1^, F. Romagnosi^2^, K. Donadello^3^, E. Polati^3^, L. Gottin^1^

##### ^1^Unit of Cardiothoracic Anesthesia and Intensive Care, Department of Emergencies and Intensive Care, University Hospital of Verona, Verona, Italy; ^2^Unit of Anesthesia and Intensive Care A, Department of Emergencies and Intensive Care, University Hospital of Verona, Verona, Italy; ^3^Unit of Anesthesiology and Intensive Care B, Department of Surgery, Dentistry, Gynecology and Pediatrics, AOUI, University of Verona, Verona, Italy

*Critical Care* 2021, **25**(**Suppl 1**): P011

**Introduction**: Although sepsis is one of the major factors affecting VA-ECMO patients' outcomes, its diagnosis is problematic because of the inflammatory cascade provoked by extracorporeal support. The study aimed to investigate the capacity of current (procalcitonin [PCT]) and novel biomarkers (presepsin [P-SEP]) to predict both severity of illness and mortality in VA-ECMO patients.

**Methods**: We performed a prospective single-center observational study of adult patients undergoing VA-ECMO following refractory cardiocirculatory shock, admitted to the Cardiothoracic ICU, University Hospital Of Verona, Italy. Subjects underwent daily plasmatic samples for dosage of PCT and P-SEP (sCD14-ST) during the total duration of 5 days from VA-ECMO cannulation. Blood cultures and tracheal aspirates were also collected daily. SOFA score was also assessed daily as the index of illness severity. The outcomes were considered in-hospital mortality and global illness severity. Sepsis was defined as the presence of both SOFA score > 7 and bacterial on any cultures.

**Results**: A total of 19 patients were enrolled in the study with a mean age of 58 ± 12 years. The average SOFA score was 11.2 (95% CI; 9.2–13.2). The majority (74%) met our sepsis criteria. In-hospital mortality was 32%. Median PCT levels were significantly higher in patients with sepsis (11.4 ± 10 ng/ml Vs. 2.6 ± 13 ng/ml, p = 0.001). The same relationship was observed for P-SEP (1410 pg/l Vs.685 pg/l, p = 0.01). Strong correlation was observed between the first 24 h SOFA score and P-SEP (r = 0.95; p > 0.001) than PCT (r = 0.48; p = 0.035). ROC curve analyses were performed. At chosen cut-off values, PCT and P-SEP accurately predict mortality in VA-ECMO patients (Fig. 1) but was inaccurate for sepsis (P-SEP, AUC 0.36).

**Conclusions**: In our VA-ECMO patients, PCT and P-SEP were not able to predict sepsis. Instead, these biomarkers were strongly related to the severity of multi-organ dysfunction, and mortality. In particular, P-SEP was strongly associated with illness severity.

**Figure:**
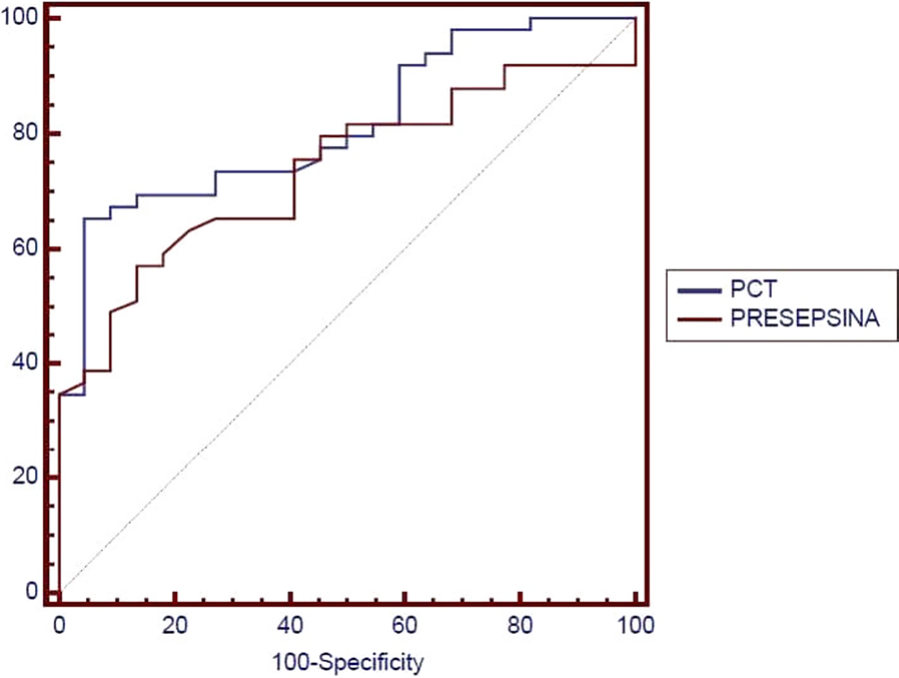
**Fig. 1** **(abstract P011)** Receiver operating characteristic curves of P-SEP and PCT level in predicting patients’ mortality (PCT, AUC 0.815; P-SEPS, AUC 0.736)

## P012

### Serum SP-A, SP-D and CC16 in COVID-19 patients

#### M. Khadzhieva^1^, A. Gracheva^2^, A. Ershov^3^, A. Kuzovlev^4^

##### ^1^Federal Research and Clinical Center of Intensive Care Medicine and Rehabilitology, Dmitry Rogachev National Research Center of Pediatric Hematology, Oncology and Immunology, and Vavilov Institute of General Genetics, Russian Academy of Sciences, Moscow, Russian Federation; ^2^Federal Research and Clinical Center of Intensive Care Medicine and Rehabilitology and Vavilov Institute of General Genetics, Russian Academy of Sciences, Moscow, Russian Federation; ^3^Federal Research and Clinical Center of Intensive Care Medicine and Rehabilitology and Sechenov First Moscow State Medical University, Moscow, Russian Federation; ^4^Federal Research and Clinical Center of Intensive Care Medicine and Rehabilitology, Moscow, Russian Federation

*Critical Care* 2021, **25**(**Suppl 1**): P012

**Introduction**: New knowledge about candidate molecular markers of damage to the structures of the aerohematic barrier in a new coronavirus infection COVID-19 will allow us to develop algorithms for early diagnosis and prediction of outcomes of acute respiratory failure in intensive care patients with COVID-19. The aim of this study was to investigate the relationship between surfactant proteins SP-A and SP-D and Club cell protein CC16 levels in blood serum and outcomes of COVID-19 patients.

**Methods**: We retrospectively investigated 109 COVID-19 patients: survivors (n = 90) and non-survivors (n = 19). The data analysis was carried out taking into account the day of illness at the time of biomaterial collection, clinical and laboratory data.

**Results**: The deceased patients presented with higher SP-A level (p = 0.009) (Fig. 1), age (p = 0.002), red blood cell distribution width (RDW) (p = 0.020), mean platelet volume (MPV) (p = 0.048), D-dimer (p = 0.049), blood urea nitrogen (p = 0.013), creatinine (p = 0.035) and lower mean corpuscular hemoglobin (MCH) (p = 0.028), mean corpuscular hemoglobin concentration (MCHC, g/l) (p = 0.002) as compared to that of survivors on 1 – 10 days of illness. On 11 – 20 days of illness the, deceased patients also presented with higher age (p = 0.042), neutrophils (p = 0.010), neutrophil-to-lymphocyte ratio (NLR) (p = 0.004), RDW (p = 0.041), lactate dehydrogenase (LDH) (p = 0.009) and lower CC16 level (p = 0.031), lymphocytes (p = 0.005) as compared to survivors. The serum SP-A and CC16 levels increased during the disease in patients with a favorable outcome (Kruskal–Wallis criterion, p = 0.035 and p = 0.018 respectively).

**Conclusions**: Serum SP-A and CC16 are associated with COVID-19-related death.

**Figure:**
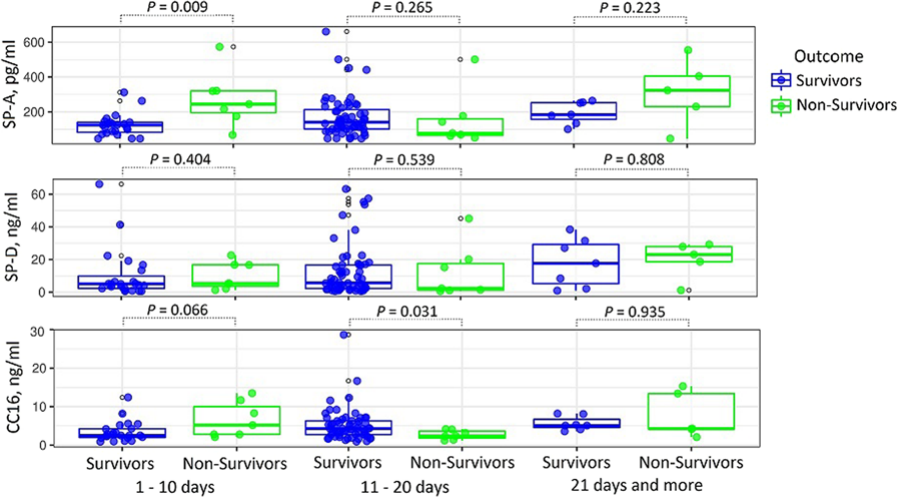
**Fig. 1** **(abstract P012)** Comparison of serum SP-A, SP-D and CC16 levels between survivor and non-survivor COVID-19 patients (Mann–Whitney U test)

## P013

### A 29-mRNA host response risk classifier enhances severity prediction in patients with suspected infection in the emergency department – a multicenter, observational study

#### F. Uhle^1^, E. Giamarellos-Bourboulis^2^, R. L. Humphries^3^, O. Liesenfeld^1^, E. Michelson^4^, E. P. Rivers^5^, J. S. Steingrub^6^, T. E. Sweeney^1^, A. Weissman^7^, D. W. Wright^8^

##### ^1^Inflammatix, Inc., Clinical Affairs, Burlingame, USA; ^2^4th Department of Internal Medicine, National and Kapodistrian University of Athens, Athens, Greece; ^3^Department of Emergency Medicine, University of Kentucky Chandler Medical Center, Lexington, USA; ^4^Department of Emergency Medicine, Texas Tech University Health Sciences Center, El Paso, USA; ^5^Department of Emergency Medicine, Henry Ford Hospital, Detroit, USA; ^6^Department of Medicine, Division of Pulmonary and Critical Care, Baystate Medical Center, Springfield, USA; ^7^Department of Emergency Medicine, University of Pittsburgh Medical Center, Pittsburgh, USA; ^8^Department of Emergency Medicine, Emory University, Atlanta, USA

*Critical Care* 2021, **25**(**Suppl 1**): P013

**Introduction**: Rapid risk stratification for patients with suspected infections is critical for appropriate resource utilization and disposition. Validated clinical risk tools like qSOFA can be calculated at the bedside but suffer from limited accuracy in predicting outcomes, while time delay between infection, host response, and disease progression hampers the utility of routine biomarkers. We examined the performance of a 29-mRNA-based algorithm (IMX-SEV-3) as a predictor of clinical deterioration based on early host response patterns to acute infection.

**Methods**: A total of 568 adult patients presenting to 6 US and 1 European emergency departments with suspected acute infection and/or sepsis of any etiology were enrolled (ClinicalTrials.gov: NCT03744741). Following consent, expression levels of 29 genes (+ 4 housekeeping genes) were measured on NanoString nCounter® and used as an input for a proprietary machine-learning classifier (IMX-SEV-3). Output severity scores were separated into three risk bands (low, moderate, and high) according to a priori locked cut-offs and compared to qSOFA for the composite endpoint “clinical deterioration” (need for vasopressors or mechanical ventilation within 7d, and/or 30d in-hospital mortality).

**Results**: 65.3% of patients were judged by forced expert adjudication to have bacterial, viral, or co-infection. Overall, 46 patients (8.1%) reached the composite severity endpoint. qSOFA positive (≥ 2 points) patients had a risk of 21.4% to reach the composite endpoint, while qSOFA negative (≤ 1 points) patients had a risk of 6.6% (Fig. 1). Adding the IMX-SEV-3 classifier improved stratification in both qSOFA subgroups effectively: For high qSOFA, the endpoint was reached in 8%, 18%, and 50% (low, moderate, and high bands, respectively), while for low qSOFA, the endpoint was reached in 2%, 11%, and 18%.

**Conclusions**: qSOFA risk estimation for increased severity and adverse outcome can be enhanced by combining with the novel IMX-SEV-3 score.

**Figure:**
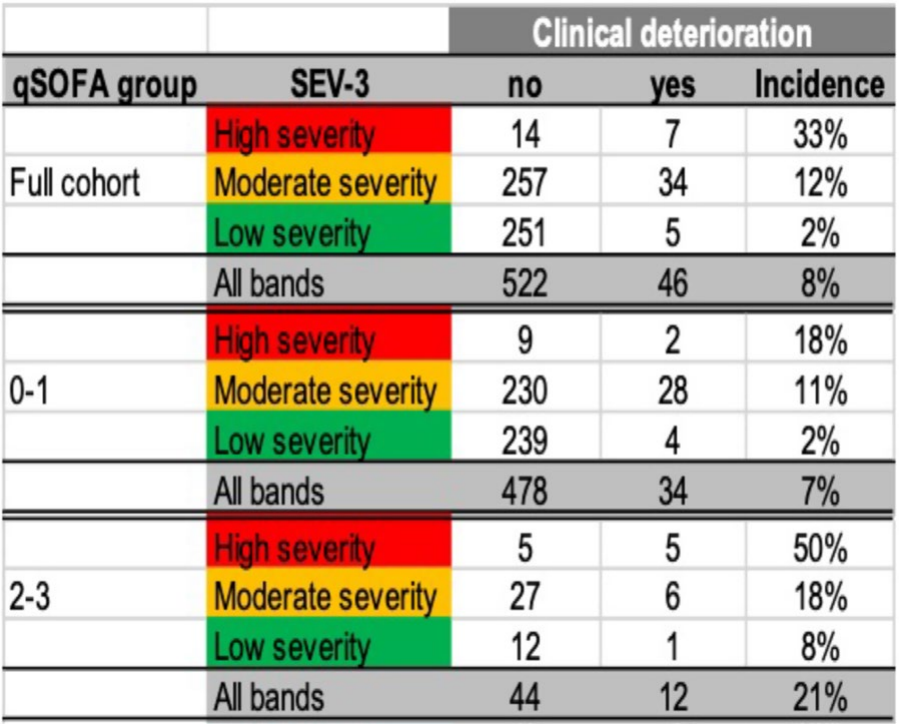
**Fig. 1** **(abstract P013)** Distribution of patients over qSOFA and SEV-3 subgroups with corresponding proportion of patients reaching the composite endpoint “clinical deterioration”

## P014

### Performance of monocyte distribution width (MDW) for the identification of COVID-19

#### C. Morales^1^, N. Robert^2^, A. Mendoza^2^, G. Rocamora^2^, A. Leis^1^, M. D. Quesada^3^, I. Castro^4^, D. Careaga^4^, L. Tejidor^4^, R. Magari^5^

##### ^1^Laboratory Medicine Department, Hospital Universitari Germans Trías i Pujol, Badalona, Spain; ^2^Emergency Department, Hospital Universitari Germans Trías i Pujol, Badalona, Spain; ^3^Microbiology Department, Hospital Universitari Germans Trías i Pujol, Badalona, Spain; ^4^Clinical Affairs, Beckman Coulter Inc., Miami, USA; ^5^Bioinformatics/Biostatistics, Beckman Coulter Inc., Miami, USA

*Critical Care* 2021, **25**(**Suppl 1**): P014

**Introduction**: This clinical study evaluated MDW, a hematology parameter that is available as part of a routine CBC-DIFF, to aid in the early identification of SARS-CoV-2 infected patients.

**Methods**: A retrospective cohort study was conducted in Spain evaluating adult patients who presented to the emergency department (ED) with symptoms consistent of COVID-19 disease and had a CBC-DIFF performed. In this preliminary analysis, forty-five (45) RT-PCR + patients were selected across the time frame of the study (March 18, 2020 – May 4, 2020). MDW results at baseline, based on a cut-off of 21.5 [1], were compared to RT-PCR results. The negative arm for this analysis constituted patients (N = 680) enrolled in a sepsis trial performed at the same site in Spain, prior to the pandemic [1]. Laboratory test results, microbial testing and radiological studies were extracted from the medical charts. Whole blood venous samples collected in K3EDTA were analyzed for MDW measurement on a UniCel DxH 900 analyzer (Beckman Coulter, Inc., Brea, CA).

**Results**: The mean values of MDW were significantly higher in SARS-CoV-2-infected patients (RT-PCR +  = 28.64 ± 3.98 vs pre-pandemic arm = 22.00 ± 4.25; p < 0.05). ROC analysis yielded an area under the curve (AUC) of 0.889 (95% CI 0.855 – 0.923) for the initial assessment of MDW levels vs. RT-PCR. At a cut-off of 21.5 units, MDW effectively differentiated SARS-CoV-2 infected patients with a sensitivity of 100% (95% CI 92.1 – 100), specificity of 53.8% (95% CI 50.1 – 57.5), PPV of 12.53% (95% CI 9.5 – 16.36), and NPV of 100% (95% CI 98.9 – 100).

**Conclusions**: An MDW cut-off of 21.5, previously validated for identification of sepsis in the ED, also reliably detects patients infected with SARS-CoV-2 virus. In this initial study, MDW demonstrated a high clinical sensitivity and negative predictive value to identify those at a lower risk for COVID-19 disease.

**Reference**

1. Hausfater P et al. Crit Care 25:227, 2021

## P015

### Cytokine storm in COVID-19: experience at an ICU in northern Mexico

#### H. R. Ramirez, A. R. Razcon, V. S. Sanchez, C. C. Chavez

##### Critical Care, Hospital San Jose Tecsalud, Monterrey, Mexico

*Critical Care* 2021, **25**(**Suppl 1**): P015

**Introduction** Mexico has been one of the hardest hit countries in the world by the ongoing COVID-19 pandemic. Death toll estimates by the John Hopkins University put the country-wide death rate at 9.4% [1]. Multiple factors have an influence on the outcome in COVID-19, with one of such being the "cytokine storm" [2]. Several tools have been created to detect this condition, including Hscore and serum levels of biomarkers, but more data is needed to assess their role and significance in the critically ill patient.

**Methods**: We performed a single-center retrospective descriptive transversal study at an ICU in a dedicated COVID-19 hospital. We recruited all severe COVID-19 confirmed adult patients with serum Interleucin-6 levels on admittance, with invasive mechanical ventilation requirements, starting March 2020 and until September 2020. After full laboratory and demographic data were collected, Hscore was calculated and put through statistical analysis with comparison between survivors at end of the follow up versus not survivors.

**Results**: A total of 820 patients with PCR-confirmed COVID-19 were identified, of which 130 required invasive mechanical ventilation during their stay. Of that group 123 patients had interleukin-6 levels screened on admittance. Both survivor and not survivor groups had similar demographic and laboratory parameters, with a non-significant difference in Hscore, ferritin and IL-6 between the groups. A positive significant association was found with Interleukin-6 levels and length of stay in the hospital (p = 0.001).

**Conclusions**: Cytokine storm as such diagnosed by Hscore seems to have no significant association with survival on a invasive mechanical ventilation population with COVID-19. Interleukin-6 is strongly associated with a lengthened hospital stay.

**References**

1. https://coronavirus.jhu.edu/data/mortality. Accessed 21–6-21
2. Fajgenbaum DC et al. N Engl J Med 383:2255–2273, 2020

## P016

### Prognosis and selection of immunotherapy in bacterial sepsis and severe COVID-19 through clinical phenotyping: a cohort study

#### E. Karakike^1^, S. Metalidis^2^, G. Poulakou^3^, G. Poulakou^3^, M. Kosmidou^4^, N. Gatselis^5^, V. Petrakis^6^, N. Rovina^7^, N. Rovina^7^, E. Gkeka^8^, M. Kyprianou^1^, E. J. Giamarellos-Bourboulis^1^

##### ^1^4th Department of Internal Medicine, National and Kapodistrian University of Athens, Athens, Greece; ^2^1st Department of Internal Medicine, Aristotle University of Thessaloniki, Thessaloniki, Greece; ^3^3rd Department of Internal Medicine, National and Kapodistrian University of Athens, Athens, Greece; ^4^1st Department of Internal Medicine, University General Hospital of Ioannina, Ioannina, Greece; ^5^Department of Internal Medicine, Larissa University General Hospital, University of Thessaly, Larissa, Greece; ^6^2nd Department of Internal Medicine,University General Hospital of Alexandroupolis, Alexandroupolis, Greece; ^7^1st Department of Pulmonary Medicine,National and Kapodistrian University of Athens, Athens, Greece; ^8^Intensive Care Unit, AHEPA University General Hospital of Thessaloniki, Thessaloniki, Greece

*Critical Care* 2021, **25**(**Suppl 1**): P016

**Introduction**: Patients with bacterial sepsis may be classified into four distinct clinical phenotypes [1]. Whether this classification may translate into a prognostic tool and treatment guidance is unclear. We aimed to extend and assess the usefulness of a simplified algorithm in phenotyping sepsis and severe COVID-19.

**Methods**: We analyzed a cohort of 1498 patients (620 with bacterial sepsis, 878 with severe COVID-19). Patients with bacterial sepsis were classified into phenotypes using a digital algorithm, based on six baseline parameters (creatinine, lactate, aspartate transaminase, bilirubin, C-reactive protein, international normalized ratio), as previously described [2]. All patients with severe COVID-19, included in an open-label immunotherapy trial (NCT04357366; NCT 04,339,712), were assessed during April to June 2020 and July to December 2020. All patients during the second period received dexamethasone. Stepwise Cox regression analysis with Acute Pathophysiology And Chronic Health Evaluation (APACHE) II as predefined variable was used to assess phenotype impact on 28-day mortality.

**Results**: Phenotypes α and γ were most prevalent in bacterial sepsis (41.8% and 22.4% respectively); δ phenotype was associated with the highest mortality (Fig. 1A). Phenotype α was seen in younger patients, mainly presenting pneumonia and respiratory failure. Phenotype assignment was an independent determinant of outcome (adjusted p: 0.007). Phenotype distribution and outcomes in severe COVID-19 were similar to those of bacterial sepsis (Fig. 1B; adjusted p: 0.0023). Phenotypes α and γ displayed favorable response to anakinra (p < 0.001), while phenotypes β and δ showed trend to improvement with tocilizumab (p = 0.047).

**Conclusions**: A simple operational algorithm classified patients with sepsis in phenotypes and predicted outcome, independently of initial severity. This classification may guide treatment response in severe COVID-9.

**References**

1. Seymour CW et al. JAMA 321:2003–17, 2019
2. Karakike E et al. Crit Care 24(Suppl 2): P589, 2020

**Figure:**
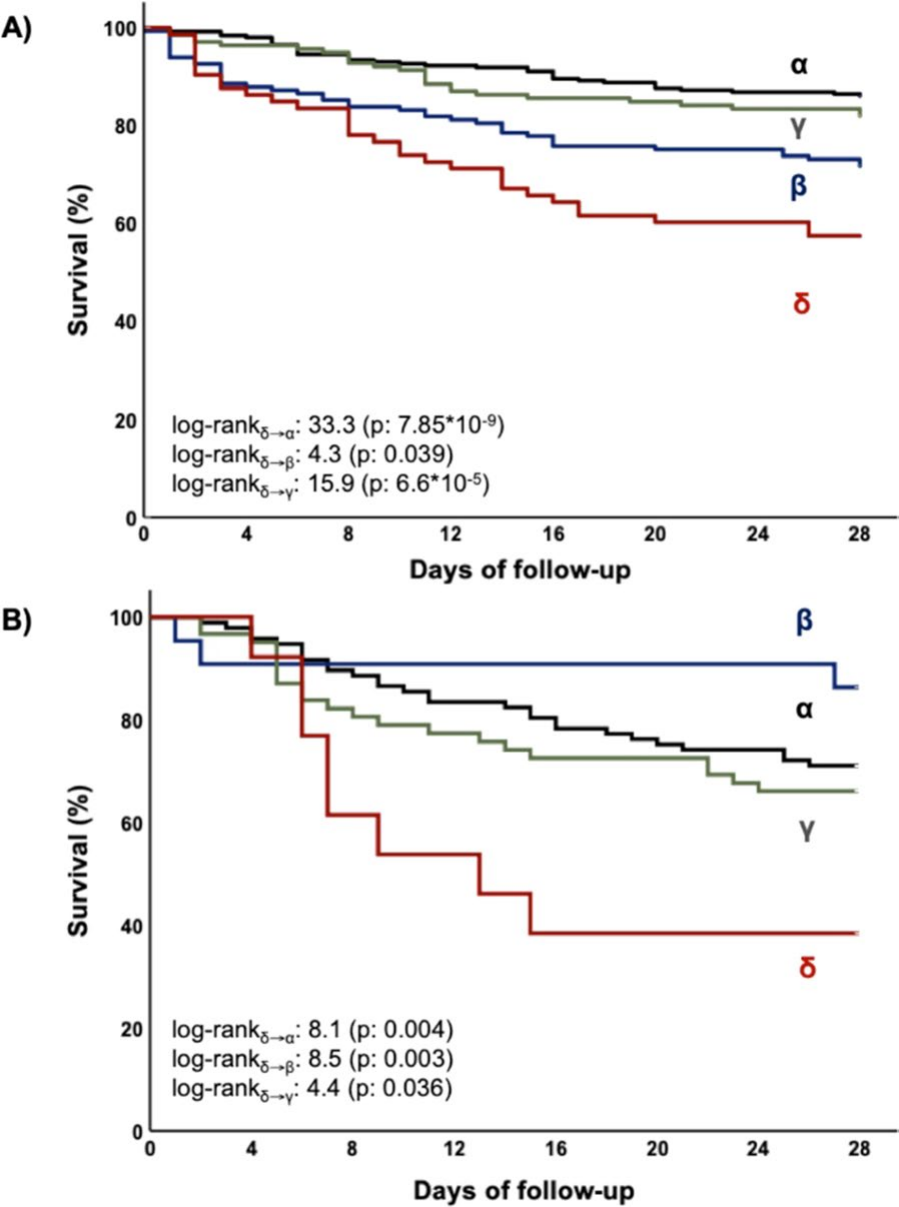
**Fig. 1** **(abstract P016)** 28-day survival by clinical phenotype (α, β, γ and δ) among patients with A) bacterial sepsis and B) severe COVID-19

## P017

### Usefulness and cost-effectiveness of procalcitonin in critical care patients admitted to the East Sussex Healthcare NHS Trust (ESHT)

#### C. Cabaret^1^, J. Perrin^2^, J. Evans^1^

##### ^1^Critical Care Medicine, East Sussex Healthcare NHS Trust, Eastbourne, UK; ^2^Pharmacy, East Sussex Healthcare NHS Trust, Eastbourne, UK

*Critical Care* 2021, **25**(**Suppl 1**): P017

**Introduction**: This study evaluated the usefulness and cost-effectiveness of procalcitonin (PCT) following its introduction to aid antibiotics discontinuation in critical care patients admitted to ESHT during the COVID-19 pandemic.

**Methods**: Non-surgical critical care patients with a diagnosis of sepsis or lower respiratory tract infection during their admission between 01st January and 30th June 2020. Retrospective analysis of data using ICCA (IntelliSpace Critical Care and Anaesthesia) to compare the number of antibiotic doses administered per patient before and after introduction of PCT. After PCT introduction, we recorded the number of PCT levels requested, their frequency as well as the level of PCT and when discontinuation occurred.

**Results**: Eighty-one patients were included—13 admitted before PCT introduction and 68 after (this important increase in the number of patients is explained by the increased proportion of patients with COVID-19 pneumonitis). The average dose of antibiotics administered per patient was reduced by 28.8% (70.24 vs 49.98) following introduction of PCT. Despite an incurred cost of £12 per PCT assay, the overall average cost per patient was reduced by £59.60 (£257.94 vs £146.78). A lack of consistency in the frequency of PCT level request was observed.

**Conclusions**: Introduction of PCT to aid discontinuation of antibiotics resulted in a 28.8% reduction in average antibiotics prescription and an overall cost reduction of £59.60 per patient. The reduction in antibacterial exposure also brings non-financial benefits such as increased patient safety through experience of less side effects, reduction in antibiotics resistance among others. The lack of consistency in the requests of PCT resulted in the design of a protocol for its use within ESHT.

## P018

### Proadrenomedullin assessment of multi-organ failure in COVID-19 sepsis (PAMOCOS): a prospective, multicentric observational study

#### S. Lhote^1^, N. Van Grunderbeeck^1^, D. Colling^2^, S. Verchain^3^, C. Varillon^4^, P. Floch^5^, C. Vinsonneau^6^, T. Caulier^7^, M. Granier^8^, J. Mallat^9^

##### ^1^Réanimation Polyvalente/USC, Centre Hospitalier de Lens, Lens, France; ^2^Réanimation Polyvalente/USC, Centre Hospitalier de Roubaix, Roubaix, France; ^3^Biochemistry Laboratory, Centre Hospitalier d´Arras, Arras, France; ^4^Réanimation Polyvalente/USC, Centre Hospitalier de Dunkerque, Dunkerque, France; ^5^Réanimation Polyvalente/USC, Centre Hospitalier de Boulogne, Boulogne, France; ^6^Réanimation Polyvalente/USC, Centre Hospitalier de Béthune, Béthune, France; ^7^Réanimation Polyvalente et Maladies Infectieuses, Centre Hospitalier de Tourcoing, Tourcoing, France; ^8^Réanimation Polyvalente/USC, Centre Hospitalier d´Arras, Arras, France; ^9^ICU, Cleveland Clinic, Abu Dhabi, United Arab Emirates

*Critical Care* 2021, **25**(**Suppl 1**): P018

**Introduction**: COVID-19 pandemic has emphatized the need for patients' good orientation, through overflow of critical care induced by surges. Mid-regional proadrenomedullin (ProADM) was suggested as an early biomarker of endothelial damage and organ failures (OF) in sepsis. We aimed to assess its predictive value in COVID-19 patients admitted in ICUs, since endotheliitis is present in COVID-19 sepsis.

**Methods**: Prospective multicentric observational study in 8 ICUs of northern France (July 2020 to February 2021). Main objective was to study association between proADM blood levels at day 1 and day 3 OF, in groups with / without worsening of OF, assessed with SOFA score. Demographic, clinical and biological data were recorded at days 1 and 3 from admission. Univariate and multivariate analysis (logistic regression) were performed. Secondary objectives studied association of proADM levels with need of mechanical ventilation (MV) at day 3 and day 28 mortality.

**Results**: A total of 170 patients were analyzed. Median age was 62; 74.7% were male, and 40.6% presented with cardiovascular comorbidity. Day 28 mortality was 20%. In univariate analysis, worsening of SOFA at day 3 was associated with day 1 proADM levels (1.21 nmol/ml [0.83–1.85] vs 0.89 nmol/mL [0.71–1.12]; p < 0.001), and also with age (p = 0.007), male (p = 0.06), Charlson comorbidity index (p = 0.02), cardiovascular comorbidity (p = 0.02), respiratory rate (p = 0.045) National Early Warning Score (p = 0.01), PaO2/FiO2 ratio (p = 0.06), KDIGO level (p = 0.043), and lymphocytes' count (p = 0.002). In multivariate analysis, there was a trend towards association of SOFA worsening at day 3 with day 1 proADM levels, without reaching significance (OR = 1.94 [0.90–4.15] p = 0.08). Secondary objectives shew in univariate analysis association of day 1 proADM with MV at day 3 (p < 0.001) and day 28 mortality (p = 0.002).

**Conclusions**: Patients' number was calculated from first-wave data, and may have been insufficient to reach significance. ProADM could be a promising biomarker in COVID-19 sepsis, regarding OF.

## P019

### Dexamethasone and tocilizumab treatment nullifies the value of C-reactive protein and procalcitonin to detect secondary bacterial infections in COVID-19 patients

#### E. J. Kooistra^1^, M. Van Berkel^2^, N. F. Van Kempen^3^, C. R. Van Latum^3^, N. Bruse^3^, T. Frenzel^3^, M. J. Van den Berg^3^, J. A. Schouten^3^, M. Kox^3^, P. Pickkers^3^

##### ^1^Intensive Care, Radboud University Medical Center, Nijmegen, Netherlands; ^2^Department of Laboratory Medicine, Radboud University Medical Center, Nijmegen, The Netherlands; ^3^Department of Intensive Care Medicine, Radboud University Medical Center, Nijmegen, Netherlands

*Critical Care* 2021, **25**(**Suppl 1**): P019

**Introduction**: Procalcitonin (PCT) and C-reactive protein (CRP) were shown to have value for the detection of secondary infections in critically ill COVID-19 patients. Since the use of immunomodulatory therapy, the value of these biomarkers is unclear. We investigated PCT and CRP kinetics in critically ill COVID-19 patients treated with dexamethasone (DEXA) with or without tocilizumab (TOCI), and assessed the value of these biomarkers to detect secondary infections.

**Methods**: Patients were divided into three groups: no DEXA/no TOCI (D-T-, n = 66), DEXA/no TOCI (D + T-, n = 44), and DEXA + TOCI (D + T + , n = 23). Serial PCT and CRP data were analyzed in the days before and after cessation of DEXA treatment. Furthermore, changes in PCT and CRP kinetics upon occurrence of a secondary infection and accuracy of these biomarkers for the detection of a secondary infection were assessed.

**Results**: Following cessation of DEXA, there was a rebound in PCT and CRP, in particular in the D + T- group. Within both the D + T- and D + T + groups, no significant increase in PCT was observed upon occurrence of a secondary infection compared to patients that did not develop an infection (p = 0.45 and p = 0.29, respectively) (Fig. 1). Although CRP increased in patients of the D + T- group who developed a secondary infection compared to those who did not (p = 0.003), this rise was only apparent from day 2 post-infection onwards. CRP remained completely suppressed in the D + T + group irrespective of the occurrence of a secondary infection. Receiver operating curve analysis of PCT and CRP levels yielded area under the curves of 0.57 and 0.59, respectively, much lower than those obtained in patients not treated with DEXA/TOCI (0.80 and 0.76, respectively).

**Conclusions**: Cessation of dexamethasone treatment in critically ill COVID-19 patients results in a rebound increase in PCT and CRP levels. Immunomodulatory treatment with dexamethasone and tocilizumab nullifies the value of PCT and CRP for detection of secondary infections.

**Figure:**
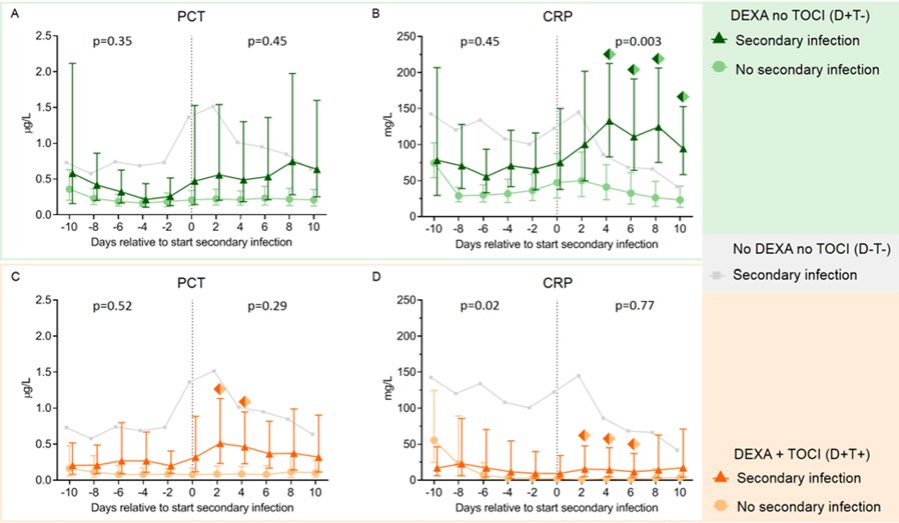
**Fig. 1** **(abstract P019)** Levels of a) procalcitonin (PCT) and b) C-reactive protein (CRP) over time within 10 days prior to and 10 days following the day of secondary infection in the D + T- group, and levels of c) PCT and d) CRP over time in the D + T + group. Day of secondary infection was designated day 0 (alignment day). Data of the no secondary infection groups were aligned on the median alignment day, which was day 12 following ICU admission. The light gray line reflects previously reported data of D-T- patients as reference. Data are presented as geometric mean with 95% confidence intervals. P-values were calculated using mixed-models analyses (time*group interaction factor). P-values in left and right parts of each panel reflect between-group differences in kinetics from day -10 until day 0 and from day 0 until day 10, respectively. Colored diamonds reflect p values of < 0.05 on the individual timepoints, calculated using Sidak’s post hoc multiple comparisons tests

## P020

### Computational endotypes of sepsis and septic shock derived from time series data

#### J. Guo^1^, H. Kim^2^, R. Stevens^2^

##### ^1^Johns Hopkins University, Baltimore, USA; ^2^Johns Hopkins School of Medicine, Baltimore, USA

*Critical Care* 2021, **25**(**Suppl 1**): P020

**Introduction**: While sepsis has been characterized through the sepsis-3 consensus criteria as organ dysfunction and dysregulated host response to infection, there remains significant heterogeneity in the prediction, identification, treatment, and outcomes of sepsis and septic shock. This project aims to discover novel subphenotypes of sepsis and septic shock using highly granular time-series data from electronic health records.

**Methods**: From the Philips multicenter eICU database, we calculated sequential organ failure assessment (SOFA) scores and determined infection status by diagnosis and/or positive culture for each adult patient every 5 min. Based on the sepsis-3 criteria met at a timepoint, patients were categorized into four data-driven cohorts, or “statuses” (No Sepsis, Suspected Sepsis [only organ dysfunction], Sepsis, or Septic Shock). 24 clinical and physiological features from 24 to 48 h after ICU admission were used for unsupervised clustering algorithms. Endotypes from clustering were characterized by length of stays (LOS) and hospital discharge outcomes.

**Results**: Clustering analysis identified five distinct groups (Fig. 1). Cluster 1 contained a large proportion of suspected sepsis patients and 3 contained most septic patients. Clusters 2 and 4 contained the majority of septic shock patients, with 2 having the larger proportion and the overall longest hospital LOS (10.7 days) and largest mortality (35%). Cluster 5 contained nearly all non-septic patients. Clusters with mixes of statuses highlight potential similarities among patients with different sepsis-3 classifications, and the limitations of consensus criteria.

**Conclusions**: Using temporal data with a broader range of features, we identified five distinct endotypes for sepsis and septic shock. The high-frequency time series may allow for a more refined, data-driven method to characterize the evolution of sepsis and stratify patients that may be misidentified through sepsis-3, better informing prediction, detection, and treatment.

**Figure:**
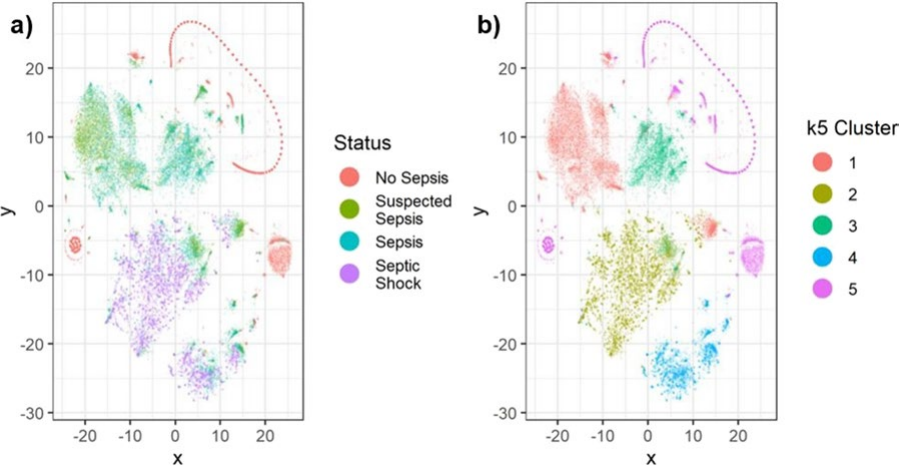
**Fig. 1** **(abstract P020)** Identification of computational endotypes. a) Visualization of patient cohorts, or “statuses,” with t-distributed stochastic neighbor embedding (tSNE). The scaled dataset containing 24 clinical and physiological variables was visualized with tSNE, a dimensionality reduction algorithm that probabilistically maps complex high-dimensional data (i.e. 24-dimensional data) to lower dimensions and identifies similar points. Each point represents one 5 min timepoint sample for each patient from 24-48 h after ICU admission. 13,487 timepoints (80% of septic shock timepoints) were randomly sampled for each status. b) Visualization of clusters from K-means (k5) on scaled data overlapped on previous tSNE plot

## P021

### Predicting the outcome in chronic critical illness (CCI) patients with health-associated infection (HAI) by peptide array technology

#### V. Pisarev^1^, A. Chapoval^2^, M. Petrova^3^

##### ^1^Federal Research and Clinical Center of Intensive Care Medicine and Rehabilitology, V.A.Negovsky Institute of General Reanimatology, Moscow, Russian Federation; ^2^Russian-American anti-Cancer Center, Altai State University, Barnaul, Russian Federation; ^3^Federal Research and Clinical Center of Intensive Care medicine and Rehabilitology, Moscow, Russian Federation

*Critical Care* 2021, **25**(**Suppl 1**): P021

**Introduction**: HAI may complicate the course of CCI increasing ICU lethality. We hypothesize that outcome of CCI patients with HAI may associate with the adaptive immune system signature exhibiting increased and decreased responses to antigenic peptides. Our goal was to determine the feasibility of the use of randomly synthesized peptides for linking antibody signatures and outcome in CCI.

**Methods**: Prospective cohort included 38 CCI patients admitted at neuroICU 32 ± 27 days (M ± SD) post-stroke and 10 healthy volunteers (control). Hospitalization lasted for 50 ± 20.8 days. Sera samples prepared from blood withdrawn on the first 24 h on admittance were stored at -18 oC. After storage, 1 μl of sera was applied on > 125 000 randomly synthesized 12-mer peptide microarrays (Biodesign Institute, Tempe, AZ, USA) and reactions were detected with Alexa Floor 647 labeled anti-IgG and laser scanner. Data analyzed using the BRB-ArrayTools software.

**Results**: On admittance, patients exhibited 15 ± 8.2 scores on NIHSS and 13 ± 2.0 scores on Glasgow Coma Scale. Ten patients deceased during the study. Twenty patients developed severe pneumonia: Group 1 complicated with sepsis (SEPSIS-3, n = 6, 1 patient survived) and Group 2 (pneumonia, no sepsis; n = 14), with decreased lethality (p = 0.002, Fisher exact test). Decreased and increased IgG responses to several peptides significantly discriminated patients from Group 1 vs. Group 2 and Group 1 patients vs. all other patients (p = 0.019) or vs. control cohort (Fig. 1). Results merit clinical validation of candidate immunosignatures followed by developing clinically relevant, simplified test format.

**Conclusions**: Repertoire of epitope-specific IgG antibodies to > 120 000 randomly synthesized peptides in CCI patients is discriminative for pneumonia vs. sepsis. Altered immunosignature may serve as an early predictive candidate biomarker of unfavorable outcome in CCI.

**Figure:**
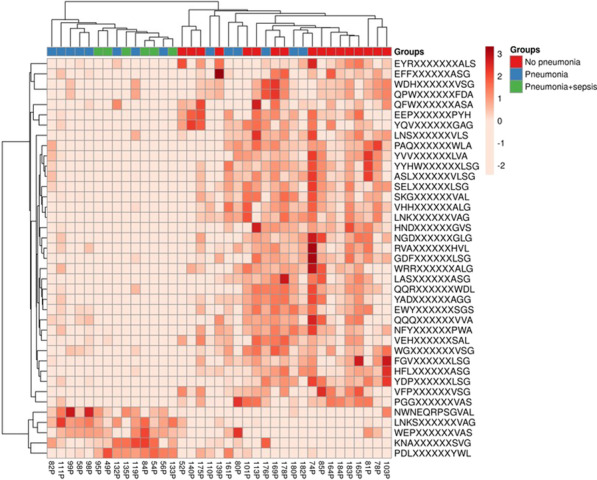
**Fig. 1** **(abstract P021)** Immunosignature by epitope-specific IgG responses in chronic critical illness patients with and without health care-associated infection in ICU

## P022

### Evaluation of endotoxin activity in critically ill patients with COVID-19

#### R. Di Mussi^1^, R. D. Iannuzziello^2^, F. M. Murgolo^2^, I. M. Magnesa^2^, A. M. Miccolis^2^, E. A. Annicchiarico^2^, F. P. Pugliese^2^, A. C. Civita^2^, S. G. Grasso^2^

##### ^1^Department of Emergencies and Organ Transplant, Bari University "A. Moro", Bari, Italy; ^2^Department of Emergencies and Organ Transplant, Bari, Italy

*Critical Care* 2021, **25**(**Suppl 1**): P022

**Introduction**: Current literature shows that an elevated endotoxin activity is often present in critically ill patients with COVID-19. SARS – CoV 2 spike protein can bind lipopolysaccharide (LPS) and may induce a “cytokine storm”. At the same time, the COVID-19 trophism for the ACE2 receptor, which is expressed also in small intestinal enterocytes, may cause a “leak gut” syndrome altering intestinal permeability and promoting the LPS translocation. Our objectives were to evaluate endotoxin activity at the admission in intensive care unit (ICU) and during ICU stay.

**Methods**: We assessed the endotoxin activity assay (EAA), a rapid in vitro diagnostic test that utilizes a specific monoclonal antibody. (Estor, Italy). EAA was measured at the ICU admission (baseline), day 3 and day 7. EAA levels were distinguished in high (EAA > 0.6) and low (EAA < 0.6) endotoxin activity. Blood gas analysis, hemodynamic and breathing pattern parameters were also recorded. We also considered the infections developed during ICU stay and the inflammatory parameters.

**Results**: The figure shows the percentage of patients with high and low EAA, respectively. Of the 40 patients enrolled, 14 had an elevated EAA at the first EAA determination. In this group, the mortality was 57%. In the group of patients without preexisting elevated EAA the mortality was 42%. At baseline, we could not find any correlation between EAA and clinical, ventilatory and hemodynamic characteristics. At day 3 and 7, lactates, presepsin and SOFA score kidney component values were significantly higher in the group of patients with EAA > 0.06.

**Conclusions**: We found that elevated EAA was 35%. This raises the question if a “hypoperfusion”, hyperinflammatory state or a direct action of SARS-CoV-2 on small intestinal enterocytes may induce LPS translocation. Further studies are needed to evaluate if high EAA at ICU admission may allow an early recognition of patients with worse ICU outcome.

**Figure:**
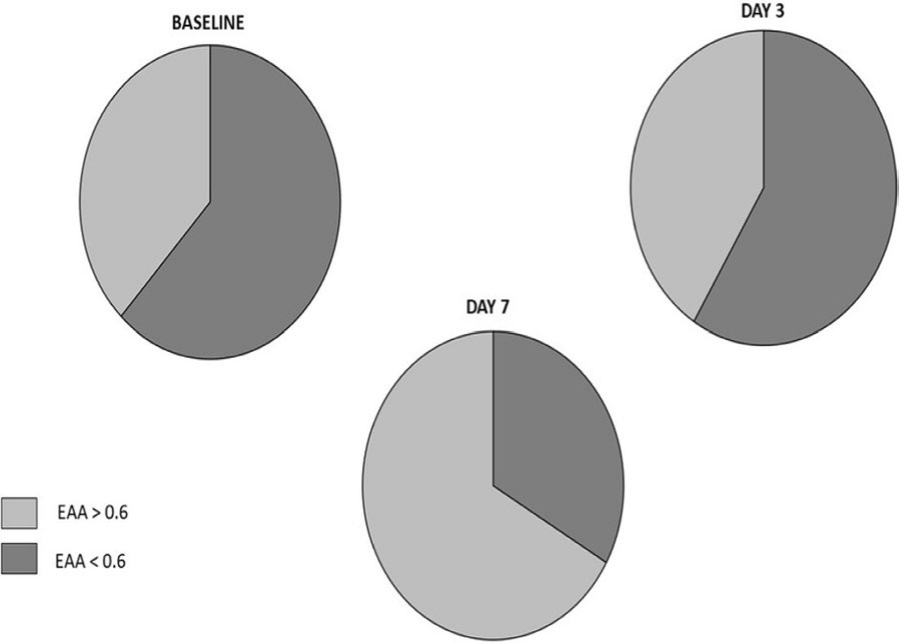
**Fig. 1** **(abstract P022)** Percentage of patients with EAA high and low, respectively during the 7 days of the study

## P023

### Outbreak of *Stenotrophomonas maltophilia* pneumonia in ICU COVID-19 patients

#### M. Raad^1^, M. Abi Haidar^2^, R Ibrahim^3^, R. Rahal^1^, J. Abou Jaoude^1^, E. Ayoub^2^, C. Harmouche^1^, G. Saliba^3^, G. Sleilaty^3^, M. Riachy^1^

##### ^1^Pulmonary and Critical Care Department, Hotel Dieu de France-St Joseph University, Beirut, Lebanon; ^2^Anesthesia and Critical Care Department, Hotel Dieu de France-St Joseph University, Beirut, Lebanon; ^3^Infectious Disease Department, Hotel Dieu de France-St Joseph University, Beirut, Lebanon

*Critical Care* 2021, **25**(**Suppl 1**): P023

**Introduction**: Secondary bacterial pneumonia is an important complication of many viral illnesses particularly in ICU COVID-19 patients. It contributes to the high morbidity and mortality in this vulnerable population. Pathogens are variable and include *Pseudomonas*, *Klebsiella*, *Acinetobacter* and *Staphylococcus* species. Recently, a new surge of many environmental bacteria was described particularly *Stenotrophomonas maltophilia*. The aim of this study was to determine the prevalence of this new outbreak of *Stenotrophomonas maltophilia* pneumonia in COVID-19 ICU patients.

**Methods**: This study was conducted in a tertiary university hospital with a setting of 23 ICU beds. All COVID-19 ICU patients admitted between March 2020 and April 2021 were analyzed. Statistical analyses were conducted to determine the prevalence of bacterial secondary pneumonia and those caused by *Stenotrophomonas maltophilia*.

**Results**: A total of 155 ICU COVID-19 admitted patients with 76% men were analyzed. Mean age is 66.5 (13.5) years and APACHE score was 13.2 (6.5). Mean ICU stay was 15.2 (15.7) days. 42 (27.5%) nosocomial pneumonia events were diagnosed. The most common isolated pathogens were *Stenotrophomonas Maltophilia* (41%), *Pseudomonas* (26%), *Enterobacteriacae* (23%), *Staphylococus* (2%) and fungal infections (3%). Death was reported in 45 patients (29%).

**Conclusions**: ICU COVID-19 patients have a multifactorial immunosuppression and are prone to nosocomial bacterial pneumonia. *Stenotrophomonas Maltophilia*, a resistant environmental bacteria, is now leading the incriminate pathogens. This outbreak is worrisome and can contribute to the high mortality rate during their long ICU stay.

## P024

### Association between clinical phenotype cohesiveness, sepsis transitions, and mortality in the ProCESS trial

#### J. Kennedy^1^, E. B. Brant^1^, C. C. Chang^2^, S. W. Wang^3^, D. A. Angus^1^, C. S. Seymour^1^

##### ^1^Clinical Research, Investigation, and Systems Modeling of Acute Illness (CRISMA) Center, Department of Critical Care Medicine, University of Pittsburgh, Pittsburgh, USA; ^2^Department of Biostatistics, School of Public Health, University of Pittsburgh, Pittsburgh, USA; ^3^Department of Biostatistics, University of Florida, Gainesville, USA

*Critical Care* 2021, **25**(**Suppl 1**): P024

**Introduction**: Sepsis is common, deadly, and heterogeneous. Prior work proposed clinical sepsis phenotypes at presentation and explored change in phenotype over time. However, little is known about how change in phenotype is associated with outcome. We explored trajectories of phenotypes in the ProCESS trial and the association between change in phenotype and outcome.

**Methods**: We analyzed a cohort of 815 adult sepsis encounters from the ProCESS trial in the Usual Care and EGDT arms that survived in-hospital for at least 72 h post-randomization. We predicted clinical phenotypes at randomization and 72-h post-randomization using Euclidean distance anchored to previously published cluster centroids (α,β,y,∂). We defined core members as ≥ 90% and marginal as < 90% probability of phenotype membership at randomization. We used logistic regression to determine the odds of phenotype transition by phenotype, treatment arm, and membership probability. We explored the relationship between treatment arm, phenotype transition, and in-hospital mortality within the ∂-phenotype.

**Results**: We studied 815 adult sepsis encounters from the ProCESS trial (median age 60, [IQR 50–72]; 56% male, median SOFA at arrival 7, [IQR 4–9]) surviving for at least 72 h post-randomization. At presentation, encounters were 36% α-type, 37% β-type, 13% y-type, and 25% ∂-type. Phenotype was unchanged over 72 h in 46% of encounters. Marginal membership was (OR 1.9, 95% CI 1.4–2.6, p < 0.01), but treatment arm was not (OR 0.9 95% CI 0.7–1.2, p = 0.33, Fig. 1A) associated with increased odds of phenotype transition. Within ∂-type, core membership and EGDT were associated with increased in-hospital mortality (Fig. 1B).

**Conclusions**: Approximately half of patients changed phenotypes within 72 h and changes were most common in marginal members. Treatment response may differ by phenotype membership probability and is an important area for further research.

**Figure:**
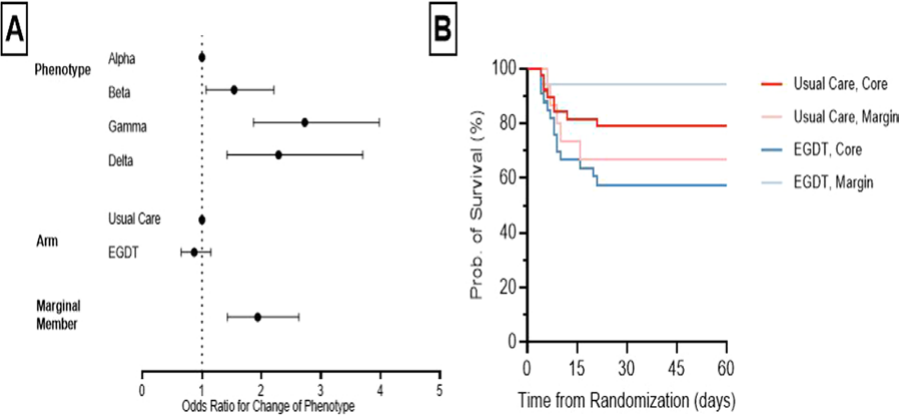
**Fig. 1** **(abstract P024)** (A) Forest plot showing probability of change in phenotype by phenotype, treatment arm, and probability of membership. (B) Kaplan–Meier Curve of 60-day in-hospital mortality, stratified by treatment arm and probability of membership within the delta phenotype

## P025

### Machine learning-guided early treatment of sepsis

#### E. Brant^1^, J. N. Kennedy^1^, C. H. Chang^2^, L. Tang^2^, C. W. Seymour^3^

##### ^1^Department of Critical Care Medicine, University of Pittsburgh, Pittsburgh, USA; ^2^Biostatistics, University of Pittsburgh, Pittsburgh, USA; ^3^Critical Care Medicine and Emergency Medicine, University of Pittsburgh, Pittsburgh, USA

*Critical Care* 2021, **25**(**Suppl 1**): P025

**Introduction**: Sepsis, dysregulated host immune response to infection resulting in organ dysfunction, accounts for more than 1 in 5 deaths worldwide. Evidence suggests a precision treatment approach of IV fluids and vasopressors may improve early sepsis care.

**Methods**: We developed a precision treatment policy for i.v. fluids and vasopressors in early sepsis using Q-learning in clinical Electronic Health Record data. We identified patients with Sepsis-3 features within the first 6 h of presentation at 14 academic and community UPMC hospitals in Pennsylvania, USA, from 2013–2017. We organized 38 model features into 4-h timesteps from hospital arrival until 48-h after sepsis onset. We defined the state space using K-means clustering. The action space was a 5 × 5 matrix of i.v. fluid and vasopressor doses, including no drug administered then doses divided into quartiles. Awards and penalties were applied, maximizing 90-day patient survival. We compared AI and Clinician policy performance using weighted importance sampling with bootstrapped confidence intervals and determined absolute risk difference in predicted 90-day mortality across patient- and hospital-level subgroups.

**Results**: We studied 30,687 early sepsis patients (mean age 64 (SD, 16) years; mean SOFA 3.5 (SD, 2.9)). The value of the AI treatment policy was significantly higher than the Clinician policy value (41.9, 95% CI: 41.2–42.7 vs 40.8, 95% CI 39.9–41.6). Predicted risk of 90-day mortality was lower among subgroups with advanced age, higher SOFA scores and non-surgical sepsis treated per the AI policy (Fig. 1A). The AI policy value exceeded the Clinician policy across all study hospitals and predicted mortality risk difference ranged from 0.01–1.18% (Fig. 1B).

**Conclusions**: Precision treatment using i.v. fluids and vasopressors is associated with lower 90-day mortality risk among early sepsis patients compared to patients treated per clinician policy, particularly among patients with advanced age, surgical sepsis and severe organ dysfunction.

**Figure:**
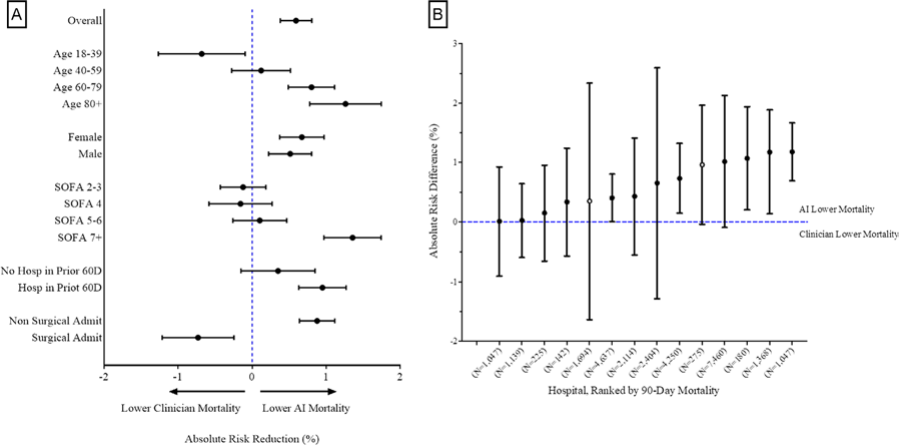
**Fig. 1** **(abstract P025)** (A) Forest plot of absolute risk difference (95% CI) in predicted 90-day mortality between AI and clinician policies by patient subgroup. X-axis corresponds to absolute risk difference (B) Caterpillar plot of absolute risk difference (95% CI) in predicted 90-day mortality between AI and clinician policies by hospital. X-axis is hospital with corresponding sepsis patient count (N). Y-axis is absolute risk difference

## P026

### Sampling peritoneal fluid during emergency laparotomy influences 40% of subsequent antimicrobial prescriptions on the ICU

#### E. Pye, S. Muschik, C. Goodwin, J. Butler, A. Georgiou

##### Intensive Care Unit, Royal United Hospitals Bath, Bath, UK

*Critical Care* 2021, **25**(**Suppl 1**): P026

**Introduction**: A key tenet of ICU care following emergency laparotomy is administration of appropriate empirical antimicrobials, with prescribing adjustment based on microbiological results. We conducted a quality improvement project with the intention of increasing the frequency of intraoperative peritoneal fluid sampling during emergency laparotomy to see how this would affect postoperative antimicrobial prescribing on the ICU.

**Methods**: A baseline audit showed the frequency of fluid sampling to be 31%. Through collaboration with our surgeons, we encouraged peritoneal fluid sampling for microbiological analysis at the time of emergency laparotomy. Any prescribing changes required from our empirical antibiotics (amoxicillin, metronidazole and gentamicin) were noted.

**Results**: Over an 18-month period, 120 emergency laparotomies were performed on 110 patients. A sample was sent from the peritoneal cavity during 76 operations (63%). Fifty-three samples yielded positive growth. Of these 36 (68%) samples showed antibiotic resistance patterns: 22 (42%) to amoxicillin/co-amoxiclav, 3 (6%) to gentamicin, and 4 cultures (7%) to both. Six (11%) grew candida, one of which was resistant to voriconazole. Of the samples yielding positive growth, a total of 31 (58%) therefore yielded results which would influence a change in prescribing from the hospital’s empirical antibiotics. For patients in whom a sample was sent for analysis, a prescription change would therefore be expected in 40%.

**Conclusions**: Our results suggest that sampling peritoneal fluid during emergency laparotomy may alter 40% of antibiotic prescriptions on ICU. Further work is required to understand how this affects patient outcome, but in the absence of these data it seems reasonable to advocate peritoneal fluid sampling during emergency laparotomy to guide subsequent antibiotic decision making on the ICU.

## P027

### Eclipse-it: elderly critically ill patient representation in interventional trials, a systematic review

#### R. Carignan^1^, M. F. Forget^1^, M. Gravel^1^, C. Durivage^1^, J. Bienvenue^1^, A. Dessureault^1^, M. Bouchard^1^, L. Munshi^2^, H. T. Wang^1^

##### ^1^Hôpital Maisonneuve-Rosemont, Montreal, Canada; ^2^Critical Care, Mount Sinai Hospital MSICU, Toronto, Canada

*Critical Care* 2021, **25**(**Suppl 1**): P027

**Introduction**: Older adults (65 years and above) represent 40 to 50% of the ICU population. Unfortunately, critical care evidence-based guidelines for this population are lacking and its representation in randomized control trials (RCTs) is unknown. We aimed to evaluate older adults’ participation in critical care RCTs and whether age is considered during analysis.

**Methods**: We conducted a systematic review of systematic reviews in Pubmed, Ovid, Central and Cochcrane, from January 1, 2009, to October 16, 2019. We included critical care systematic reviews and meta-analysis of RCTs based on five ICU topics deemed important for older ICU patients: Acute respiratory distress syndrome, sepsis, nutrition, mobilization and sedation. We collected studies’ baseline characteristics, intervention type, and extracted any age information in methods and results. We calculated the proportion of RCTs excluding older adults, with an age central tendency above 65 years, and with outcomes stratified by age and with age-specific subgroup analysis. We used Chi-squared and Kruskal–Wallis tests for comparison between groups.

**Results**: We included 152 RCTs in our systematic review. Older age was an exclusion criterion in 17 (11%) RCTs. Between them, average age was over 65 years for 4 (24%) RCTs and over 70 years for 1 (1%). Those proportions were similar to the 132 trials without age exclusion (p = 0.581). Through the 152 RCTs, outcomes were stratified by age for 5%. Age-specific exploratory subgroup analysis was performed in 5 (3%) trials and 1 RCT reported an interaction between age and intervention, and reported different outcomes in older adults and not for younger ones.

**Conclusions**: Older adults’ participation remains underrepresented in intensive care RCTs. The treatment effect differences are not documented. This heightens the need for more age consideration in studies and more studies dedicated for older adults in order to help clinician treat this growing population.

## P028

### Trends in the epidemiology and treatment of sepsis—a nationwide observational study

#### E. Vesteinsdottir^1^, M. I. Sigurdsson^1^, A. Blondal^2^, M. Gottfredsson^3^, S. Karason^1^

##### ^1^Department of Anesthesia and Intensive Care, Landspitali - The National University Hospital of Iceland, Reykjavik, Iceland; ^2^Department of Anesthesia and Intensive Care, Akureyri Hospital, Akureyri, Iceland; ^3^Department of Infectious Disease, Landspitali - The National University Hospital of Iceland, Reykjavik, Iceland

*Critical Care* 2021, **25**(**Suppl 1**): P028

**Introduction**: Registry-based studies have shown a trend towards increasing incidence of sepsis, with declining mortality rates in recent years, but there is concern that this might be due to variability in coding practices. The objectives of this study were to describe the incidence and outcome of sepsis using observed clinical criteria rather than discharge coding.

**Methods**: This was a retrospective, observational study. All adult admissions to Icelandic (ICUs) during six years (2006, 2008, 2010, 2012, 2014 and 2016) were screened for severe sepsis or septic shock by ACCP/SCCM criteria. Population incidence, patient characteristics, treatment received and outcome were compared across the study years.

**Results**: During the six study years, 9166 patients were admitted to Icelandic ICUs, 971 (10.6%) because of severe sepsis or septic shock. The crude incidence of sepsis requiring admission to ICU remained stable between 0.55–0.75 per 1000 inhabitants. No significant trends were observed over time in patient age (mean 64.7 years), APACHE II score (mean 21.4), SOFA score (mean 8.1) or Charlson Comorbidity Index (mean 4.2). Mortality rates declined slightly from the first study year (2006) to the last (2016), both 28 day (27.7% to 22.2%, p = 0.25) and one year 45.8% to 38.2%, p = 0.17). Patients admitted to the ICU from the emergency department (ED) were 477 (49.1%). Length of stay in the ED before ICU admission increased from 2:52 (hr:min) in 2006 to 4:53 (p = 0.003) in 2016 and the time to drawing of blood cultures increased from 0:41 in 2008 to 1:21 in 2016 (p = 0.013). The time to a lactate measurement decreased from 4:03 in 2006 to 1:09 in 2016, p < 0.001). Achievement of the goals of the Surviving Sepsis Campaign Guidelines was not associated with better 28-day survival (p = 0.60).

**Conclusions**: Using observed clinical criteria in a nationwide population, the incidence and outcome of sepsis did not change over an 11-year period. Variations in treatment parameters are likely explained by organizational alterations.

## P029

### Changing the treatment for *Clostridium difficile* infection

#### P. Geceviciene, D. Adukauskiene, R. Mickus, A. Dambrauskiene

##### Intensive Care Unit, Lithuanian University of Health Sciences, Kaunas, Lithuania

*Critical Care* 2021, **25**(**Suppl 1**): P029

**Introduction**: Oral vancomycin (V) is an antibiotic (A) of choice for *Clostridium difficile* infection (CDI) since 2017 [1]. The aim of study was to compare clinical and treatment aspects of CDI in 2014–2015 and 2019–2020.

**Methods**: A retrospective observational study was carried out in Intensive Care Clinic of LUHS. Data of Department of Infection Control records of diarrhetic patients (pt) with positive CD toxins A/B in stools were used, including: risk factors (age ≥ 65 years (yr), Charlson Comorbidity Index (CCI) > 5), initial treatment (IT) (V/metronidazole), duration of IT, route of administration (adm) (oral/ intravenous (i.v.), rate (R) of recurrent (RC) CDI, admission to intensive care unit (ICU) due to CDI and in-hospital mortality (IHM). Level of statistic significance – p < 0.05.

**Results**: A total number of 100 pt in 2014–2015 and 71 pt in 2019–2020 were included. 54% (n = 54) of pt ≥ 65 yr old in 2014–2015 and 53.5% (n = 38) in 2019–2020, p ≥ 0.05. CCI > 5 was found in 48% (n = 48) of pt in 2014–2015, and in 54.9% (n = 39) in 2019–2020, p ≥ 0.05. IT with V was given to 9% (n = 9) of pt in 2014–2015 and to 39.4% (n = 28) of pt in 2019–2020, p < 0.05. A median duration of IT was 10 (7;14)d in 2014–2015 and 8.5 (4;12)d in 2019–2020,p = 0.039. i.v. adm of A was used in 12% (n = 12) in 2014–2015, and in 2.8% (n = 2) in 2019–2020, p = 0.073. RC CDI was diagnosed in 13% (n = 13) in 2014–2015 and 7% (n = 5) in 2019–2020, p ≥ 0.05. 6% (n = 6) of pt were admitted in ICU due to CDI in 2014–2015 and 2.8% (n = 2) in 2019–2020, p ≥ 0.05. IHM R for pt with CDI was 27% (n = 27) in 2014–2015 and 16.9% (n = 12) in 2019–2020, p ≥ 0.05.

**Conclusions**: Rates of patients in elderly and comorbidities as risk factors for CDI did not differ. Increased rate of vancomycin use for initial treatment and shorter duration of treatment were found in 2019–2020. Rates of intravenous antibiotic administration for CDI, cases of recurrent CDI, admission to ICU due to CDI and in-hospital mortality for CDI patients in 2019–2020 decreased insignificantly.

**Reference**

1. McDonald LC et al. Clin Infect Dis 66:e1–48, 2018

## P030

### Beta-blocker therapy in patients with sepsis - beneficial effects on mortality with pre-exposure or newly initiated treatment: a systematic review and meta-analysis

#### I. Lakbar^1^, A. Lopez^1^, L. Delamarre^1^, L. Boyer^2^, S. S. Scholz^3^, D. Luque-Paz^4^, S. Rehberg^3^, M. Leone^1^

##### ^1^Anesthesie Reanimation, Assistance Publique des Hôpitaux de Marseille, Marseille, France; ^2^Santé Publique, Assistance Publique des Hôpitaux de Marseille, Marseille, France; ^3^Department of Anaesthesiology, Intensive Care, Emergency Medicine, Transfusion Medicine and Pain Therapy, University Hospital of Bielefeld, Bielefeld, Germany; ^4^Service des Maladies Infectieuses et de Réanimation Médicale, Centre Hospitalier Universitaire de Rennes, Rennes, France

*Critical Care* 2021, **25**(**Suppl 1**): P030

**Introduction**: Adrenergic antagonism has been reported to reduce mortality in sepsis, whether administered before or during the septic episode. We aimed to systematically review the literature relating to the outcomes of patients with sepsis exposed to beta-blockers either before or during their septic episode.

**Methods**: We searched PubMed, Medline, the Web of Science and the Cochrane Library databases for studies reporting mortality outcomes in septic patients receiving beta-blockers up to 1 May 2021. We conducted analyses to measure the crude and adjusted mortality rates, along with sensitivity analyses. We evaluated the certainty of evidence according to the GRADE approach.

**Results**: We identified and included 25 studies reporting data for 63,218 patients. The mortality rates were lower in patients receiving beta-blockers either before or during their septic episode, compared to the controls, with mortality odds ratios (OR) of OR 0.82 (95%CI [0.74–0.91], p < 0.001) and 0.52 (95%CI [0.31–0.85], p = 0.01) respectively. Pooled, adjusted and sensitivity analyses revealed a protective association between beta-blocker exposure and mortality with a low certainty of evidence.

**Conclusions**: In conclusion, our meta-analysis suggests that, in patients with sepsis and septic shock, pre-exposure to beta-blockers and the introduction of beta-blocker therapy during sepsis treatment may be associated with reduced mortality.

## P031

### Co-infections and superinfections in COVID-19 patients – is there any argument of antimicrobial use?

#### I.A. Florea, I.R. Darie, I.M. Dumitru, C. Cobilinschi, M. Tiglis, A. Baetu, I.M. Grintescu

##### Anaesthesia and Intensive Care, Clinical Emergency Hospital of Bucharest, Bucharest, Romania

*Critical Care* 2021, **25**(**Suppl 1**): P031

**Introduction**: The outcome of patients with viral respiratory infections may be affected by the simultaneous bacterial infection occurrences. Considering the widespread overuse of antibiotics during coronavirus disease-2019 (COVID-19) pandemic, the rate of co-infections and/or superinfections in patients infected with the novel severe acute respiratory distress syndrome-coronavirus-2 (SARS-CoV-2) remains to be elucidated.

**Methods**: In order to evaluate the emerging rate of bacterial co-infections and/or superinfections, defined according to Centers for Disease Control and Prevention criteria, we performed a retrospective, observational study that included 157 critically ill patients with severe SARS-CoV-2 infection. Survival rate and the main risk factors were also analyzed in the study group.

**Results**: In the study group, only 33.12% met the criteria for coinfection and15.92% (n = 25) for superinfection. Survival rate evaluated based on the analysis of Kaplan–Meier curves highlighted that patients with co-infections have a median survival of 8 days vs.18 days for those with superinfections, p = 0.0074. Patients suffering from diabetes mellitus registered an increase of superinfections rate (chi-squared = 6.295, p = 0.01, OR = 2.97). Moreover, patients previously treated with remdesivir recorded a higher risk of superinfections (coefficient = 1.26, p = 0.02, OR = 3.55).

**Conclusions**: The rate of co-infections and/or superinfections in COVID-19 patients is rather low, therefore the rationale of the antimicrobial therapy use should be reconsidered. As already stated, co-infections are associated with a poorer survival rate in patients with severe viral respiratory infections, even with the novel coronavirus. The main risk factors for hospital-acquired superinfections in COVID-19 patients were diabetes mellitus and the use of antiviral therapy.

## P032

### Extracorporeal hemoperfusion with an LPS-selective mesoporous polymeric adsorbent decreases neutrophil-to-lymphocyte ratio (NLR) in septic shock patients

#### V. Pisarev^1^, M. Magomedov^2^, T. Kim^2^, S. Masolitin^2^, A. Yaralian^2^, E. Kalinin^2^

##### ^1^Federal Research and Clinical Center of Intensive Care medicine and Rehabilitology, V.A.Negovsky Institute of General Reanimatology, Moscow, Russian Federation; ^2^Pirogov City Clinical Hospital No.1, Moscow, Russian Federation

*Critical Care* 2021, **25**(**Suppl 1**): P032

**Introduction**: Extracorporeal hemoperfusion (EH) eliminates excessive endotoxin or cytokines from circulation to increase survival [1]. NLR represents a sensitive and reliable prognostic/predictive biomarker of stress and inflammation [2, 3]. Decreasing the NLR links to a favorable outcome in critically ill patients [4]. We investigated whether diminishing the NLR is feasible following EH with a novel adsorber based by hypercrosslinked styrene–divinylbenzene copolymer with immobilized lipopolysaccharide-selective (LPS) ligand designed to bind LPS and other biologically active molecules [5–7].

**Methods**: The study group included 9 septic shock post-surgery patients with abdominal sepsis, pyelonephritis, severe pneumonia, and endotoxin activity by EAA test exceeding 0.6 units. EH was performed using the Efferon LPS adsorber (Efferon, Moscow, Russia).

**Results**: EH resulted in a rapid decrease in both interleukin-1 and endotoxin levels, SOFA and APACHE II score values, increased PaO_2_/FiO_2_ oxygenation index, restoration of blood pressure values. Requirements for norepinephrine were decreasing gradually and completely resolved in 1–3 days. Plasma lactate and pH levels returned to norm values by day 2–3. NLR was significantly decreased on day 5 post-EH (Fig. 1). Seven patients survived with clinical improvements. Unfortunately, two patients exhibiting extraordinary increased pre-EH NLR (21.5 and 98), APACHE (> 30), APTT (> 40), and endotoxin activity (> 0.9) died on days 4 and 8.

**Conclusions**: EH with Efferon LPS adsorber decreased the NLR on day 5 post-EH associated with survival of patients exhibiting pre-EH NLR values < 16 and endotoxin activity < 0.9 by EAA test.

**References**

1. Rachoin J et al. Crit Care Explor. 2: e0083, 2020.
2. Zahorec R. Bratisl Lek Listy 118,321–323, 2017.
3. Liu Y et al. J Clin Lab Anal. 33: e22942, 2019.
4. Ham S et al. Sci Rep. 10: 21,513, 2020.
5. Morozov AS et al. General Reanimatology 12:82–107, 2016.
6. Khoroshilov S. et al. General Reanimatology14: 51–60, 2018.
7. Ushakova N et al. General Reanimatology 16: 14–20, 2020.

**Figure:**
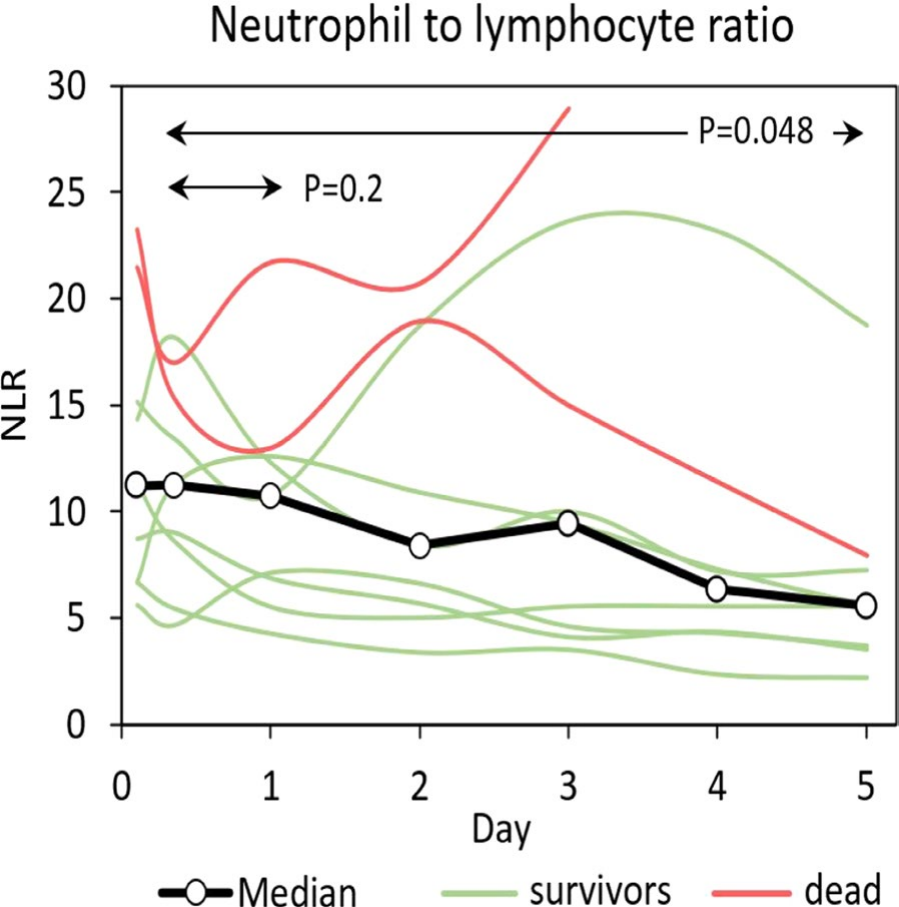
**Fig. 1** **(abstract P032)** NLR dynamics following extracorporeal hemoperfusion

## P033

### Bioelectrical impedance analysis as a bedside tool to estimate volume of distribution of hydrophilic antibiotics in critically ill patients

#### M. Gijsen^1^, E. Simons^1^, P. Annaert^2^, J. Wauters^3^, Y. Debaveye^4^, I. Spriet^5^

##### ^1^Pharmacy Department, UZ Leuven, Leuven, Belgium; ^2^Drug Delivery and Disposition, Department of Pharmaceutical and Pharmacological Sciences, KU Leuven, Leuven, Belgium; ^3^Medical Intensive Care Unit, UZ Leuven, Leuven, Belgium; ^4^Laboratory for Intensive Care Medicine, Department of Cellular and Molecular Medicine, KU Leuven, Leuven, Belgium; ^5^Clinical Pharmacology and Pharmacotherapy, Department of Pharmaceutical and Pharmacological Sciences, KU Leuven, Leuven, Belgium

*Critical Care* 2021, **25**(**Suppl 1**): P033

**Introduction**: Increases in distribution volume (Vd) have been suggested to be responsible for subtherapeutic exposure to hydrophilic antibiotics in critically ill patients with sepsis. Until today, no simple and accurate tool has been found to quickly quantify the Vd of antibiotics. Therefore, the primary aim of the present study was to explore the correlation between bioelectrical impedance analysis (BIA) estimated fluid volumes and the Vd of several hydrophilic antibiotics in critically ill patients. Furthermore, the relationship between BIA measurements and the sequential organ failure assessment (SOFA) score and cumulative fluid balance was evaluated.

**Methods**: A prospective observational study was carried out in patients treated with amoxicillin/clavulanic acid, meropenem, piperacillin/tazobactam or vancomycin, admitted to the intensive care unit (ICU) of UZ Leuven. BIA measurement was performed during the same dosing interval as the collection of blood samples to calculate the Vd of the administered antibiotic. Vd was calculated using non-compartmental analysis.

**Results**: In total, 68 patients were included. As shown in Table 1, significant correlations between Vd and BIA measurements of body water were found for amoxicillin/clavulanic acid (r > 0.78; strong), meropenem (r > 0.43; moderate) and piperacillin/tazobactam (r > 0.51; moderate). When all antibiotics were pooled, correlation remained significant, albeit weak (r > 0.32). No significant correlation was found with the Vd of vancomycin. Patients with an abnormal BIA estimated hydration status appeared more severely ill (i.e., higher SOFA scores). No association with cumulative fluid balance was found.

**Conclusions**: This study demonstrated the existence of a significant positive correlation between BIA assessed fluid status and the Vd of antibiotics at the ICU. However, correlations were not as strong as expected and absolute BIA measurements were not found to be useful in clinical practice.

**Table:** **Table 1** **(abstract P033)** Results of correlation between BIA estimated fluid volumes and the volume of distribution of several antibiotics (separate and all antibiotics pooled)

Antibiotic	TBW (L)	ECW (L)	ICW (L)	ECW (%)
Amoxicillin/clavulanic acid (n = 10)	45.85 ± 11.02; r = 0.90* [0.66;1.00]	22.84 ± 6.31; r = 0.78* [-0.003;1.00]	23.01 ± 5.31; r = 0.88* [0.55;1.00]	49.55 ± 4.07; r = -0.042 [-0.80;0.81]
Meropenem (n = 21)	44.05 ± 9.78; r = 0.47* [0.03;0.76]	22.49 ± 6.35; r = 0.43* [0.03;0.71]	21.56 ± 3.65; r = 0.50* [0.21;0.71]	50.43 ± 3.47; r = 0.25 [-0.21;0.59]
Piperacillin/tazobactam (n = 16)	39.90 ± 8.78; r = 0.34 [-0.22;0.73]	19.33 ± 4.56; r = 0.51* [0.13;0.76]	20.56 ± 4.48; r = 0.16 [-0.4;0.62]	48.31 ± 2.66; r = 0.57* [0.04;0.86]
Vancomycin (n = 21)	40.87 ± 11.89; r = 0.28 [-0.22;0.70]	20.06 ± 6.45; r = 0.20 [-0.30;0.61]	20.81 ± 5.78; r = 0.29 [-0.20;0.67]	48.90 ± 3.46; r = -0.21 [-0.66;0.28]
Total – all antibiotics pooled (n = 68)	42.36 ± 10.45; r = 0.33* [0.10;0.55]	21.05 ± 6.05; r = 0.32* [0.09;0.53]	21.31 ± 4.78; r = 0.33* [0.10;0.52]	49.33 ± 3.42; r = 0.12 [-0.16;0.39]

Values are represented as mean ± SD. ECW = extracellular water; ICW = intracellular water; r = correlation coefficient [95% confidence interval]; TBW = total body water. * p < 0.05

## P034

### Vancomycin-resistant ***Enterococcus faecium*** bacteremia in an intensive care unit: incidence and risk factors

#### W. Sellami, I. Ben Mrad, M. Zakraoui, S. Bougheriou, M. Ferjani

##### Department of Anesthesiology and Intensive Care Unit, Military Hospital of Tunis, Tunis, Tunisia

*Critical Care* 2021, **25**(**Suppl 1**): P034

**Introduction**: Vancomycin-resistant *Enterococcus faecium* (VREF) is one of important etiologies of nosocomial infections in critically ill patients. VREF bacteremia was associated with a poorer outcome, given that overall mortality rates may reach values higher than 60% with an attributable mortality of around 40%. Few data are available concerning factors associated with mortality in the context of VREF bacteremia in different centers. The aim of our study was to determine incidence and risk factors associated with VREF bacteremia in an intensive care unit.

**Methods**: A retrospective case–control study was performed in the ICU from January 2014 to December 2020. Cases were defined as septic patients with VREF isolated from a blood culture. VREF was defined as an *E. faecium* isolate with an MIC of vancomycin ≥ 32 μg/ml by the E-test according to the standards of the Clinical and Laboratory Standards Institute (CLSI). Control patients were randomly drawn from 50 hospitalized patients with vancomycin-susceptible *E. faecium* (VSEF) isolated from a blood culture.

**Results**: Seventeen case patients and 50 control patients with at least one positive *E. faecium* blood culture were identified. The demographic and clinical characteristics of the case and control groups were similar, except for mean duration of length of stay (68 ± 9 vs 22 ± 8, p < 0.0001). Mortality did not differ significantly between those with VREF (25%) and those with VSEF (14%) isolates (p = ns). In the univariate analysis, the significant risk factors for VREF bloodstream infections included diabetes mellitus (p = 0.04), end-stage renal disease (p = 0.03), prior exposure to corticosteroids (p = 0.02), prior receipt of vancomycin (p = 0.04) and a prolonged length of stay in ICU (p < 0.01) (Table 1).

**Conclusions**: VREF bacteremia in critically ill patients was associated with a poorer outcome. Several risk factors have been identified and they should be considered in infection control practice to prevent VREF infection or colonization and to reduce they duration.

**Table:** **Table 1** **(abstract P034)** Risk factors for vancomycin-resistant *Enterococcus faecium* bacteremia

	**VREF Case group (n = 17)**	**VSEF Control group (n = 50)**	**p value**
Length of stay in ICU (mean ± SD, days)	68 ± 9	22 ± 8	< 0.01
Prior receipt of vancomycin	14	5	0.04
Diabetes mellitus	10	1	0.04
End-stage renal disease	7	0	0.03
Prior exposure to corticosteroids	10	6	0.02

## P035

### Impact of intensive care unit acquired infections caused by multidrug resistant organisms on costs: preliminary findings of a Brazilian multicenter cohort study

#### A.P. Nassar Jr, I.L. Bezerra, M. Rodrigues, E.C. De Sousa, P.H. De Lucas, A. De Carvalho, A.C. Da Cruz, A.H. De Souza, D.T. Malheiro, A.J. Pereira

##### Social Responsibility Institute, Hospital Israelita Albert Einstein, São Paulo, Brazil

*Critical Care* 2021, **25**(**Suppl 1**): P035

**Introduction**: The aim of this study was to assess the impact of MDR infections acquired in intensive care units (ICU) on patients’ costs during ICU stay.

**Methods**: We included all consecutive patients ≥ 18 years admitted to 10 Brazilian ICUs from October 2019 to December 2020. We measured all fixed (personnel, overheads, depreciation) and variable costs (lab/image tests, transfusions, renal replacement therapy, medical materials and drugs) during ICU stay ICU-acquired infections were those diagnosed 48 h or later after ICU admission. We considered carbapenem-resistant *Acinetobacter*, *Pseudomonas* and *Enterobacteriaceae*; vancomycin-resistant *Enterococcus*; methicillin-resistant *Staphylococcus*; and imidazole-resistant *Candida* as MDR microorganisms. We considered non-multidrug resistant (NMR) all other infections. We built a linear mixed model, with ICU as a random-effect, and age, sex, type of admission (medical, elective surgical and emergent surgical), Charlson Comorbidity Index (CCI), frailty measured by the Modified Frailty Index (MFI) and SAPS 3 as confounders to assess the association between ICU-acquired infection by MDR microorganisms and daily patient cost.

**Results**: A total of 3077 patients were included, from whom, 70 (2.3%) acquired ICU MDR infections and 237 (7.3%) acquired NMR infections. Demographic, clinical and patients’ outcomes are presented in Table. ICU mortality and length-of-stay were increased in patients with NMR and MDR ICU-acquired infections. Fixed and variable costs were increased in patients with NMR and MDR ICU-acquired infections. MDR infections (β = USD 52.34; CI 95%, 12.60–92.07) but not NMR infections (β = USD 16.13; CI 95%, 6.55–38.80) were associated with increased daily patient ICU costs.

**Conclusions**: MDR ICU-acquired infections are associated with increased daily and total ICU costs. Its prevention may have a considerable impact on healthcare costs.

**Table:** **Table 1** **(abstract P035)** Clinical, demographic, outcomes and ICU costs of included patients

	**MDR ICU-acquired infection (N = 70)**	**NMR ICU-acquired infection (N = 237)**	**No ICU-acquired infection (N = 2770)**	**p**
Age, mean (SD)	59.21 (15.93)	58.79 (16.48)	59.40 (17.81)	0.88
SAPS 3	62.26 (17.62)	56.69 (16.34)	50.53 (18.43)	< 0.01
ICU length-of-stay, days, mean (SD)	23.93 (14.95)	16.56 (11.39)	5.62 (6.25)	< 0.01
ICU mortality, N (%)	35 (50.0)	115 (48.5)	853 (30.8)	< 0.01
Total fixed costs, USD, mean (SD)	52,604.41 (49,003.76)	29,174.87 (25,423.15)	12,172.63 (15,936.33)	< 0.01
Total variable costs, USD, mean (SD)	16,957.23 (13,046.32)	10,469.82 (9,993.17)	3,085.50 (4,621.09)	< 0.01
Daily patient cost, USD, mean (USD)	2,963.59 (1,607.81)	2,454.47 (920.41)	2,747.10 (1,517.97)	< 0.01

## P036

### The risk-adjusted association between antibiotic timing and mortality among clinical sepsis phenotypes

#### A. Yang^1^, J. N. Kennedy^2^, G. Phillips^3^, K. M. Terry^3^, M. M. Levy^4^, C. W. Seymour^5^

##### ^1^Department of Internal Medicine, UPMC, Pittsburgh, USA; ^2^Critical Care Medicine, University of Pittsburgh, Pittsburgh, USA; ^3^IPRO, Lake Success, USA; ^4^Department of Pulmonary and Critical Care Medicine, Brown University School of Medicine, Providence, USA; ^5^Department of Critical Care Medicine and Emergency Medicine, University of Pittsburgh, Pittsburgh, USA

*Critical Care* 2021, **25**(**Suppl 1**): P036

**Introduction**: Sepsis is a common, deadly, and heterogeneous syndrome. Four clinical phenotypes of sepsis are proposed, yet the association between sepsis phenotypes and treatment response is unknown. To address this knowledge gap, we investigated the relationship between antibiotic treatment delay and mortality when modified by clinical sepsis phenotype.

**Methods**: We analyzed a retrospective cohort of adult patients with sepsis and septic shock as reported to the New York State Department of Health (2015 to 2017), and identified clinical sepsis phenotypes (α,β,γ,∂) using a modified approach from the Sepsis ENdotyping in Emergency Care (SENECA) algorithm [1]. We used multivariable logistic regression to understand association between time to antibiotic administration and in-hospital mortality by sepsis phenotype.

**Results**: We studied 55,169 encounters (median age 72 years, [IQR 60–83 years]; 52% male, 22% in-hospital morality) and found 34% α-type, 30% β-type, 19% γ-type, and 17% ∂-type. The α-type was younger with the lowest in-hospital mortality, the β-type was older with more renal dysfunction, and ∂-type had elevated lactate levels with the highest proportion of septic shock. Antibiotic treatment delay was associated with increased risk-adjusted in-hospital mortality across all phenotypes, but effect size varied by phenotype (α: OR 1.03, 95%CI 1.00–1.05, p = 0.08; β: OR 1.04, 95%CI 1.02–1.06, p < 0.01; γ: OR 1.02, 95%CI 0.99–1.05, p = 0.18; ∂: OR 1.07, 95%CI 1.05–1.10, p < 0.01; Interaction p-value 0.045).

**Conclusions**: More rapid completion of administration of antibiotics, particularly among the delta clinical phenotype, was associated with lower risk-adjusted in-hospital mortality.

**Reference**

1. Seymour CW et al. JAMA 321: 2003–2017, 2019

**Figure:**
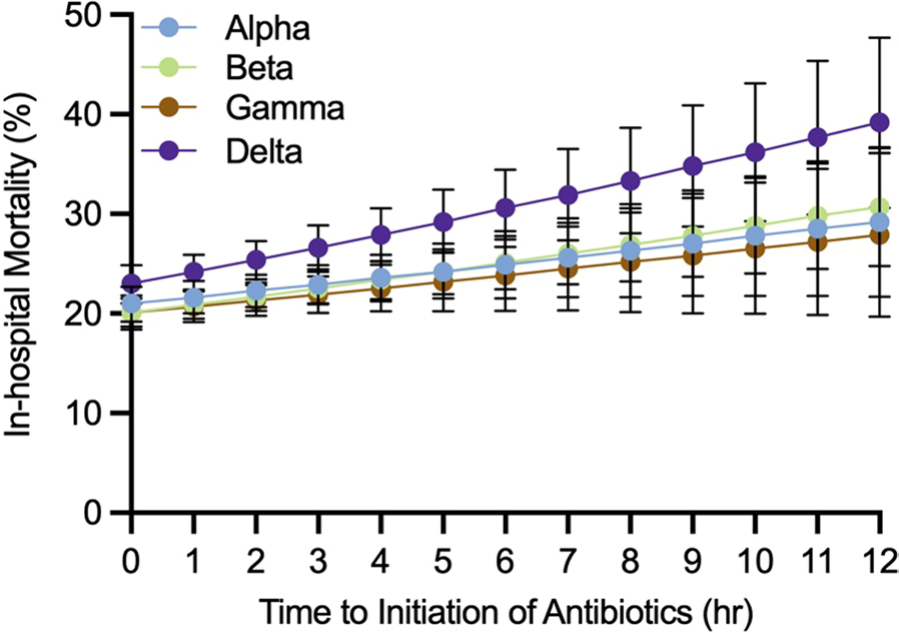
**Fig. 1** **(abstract P036)** Predicted risks of risk-adjusted in-hospital death by clinical sepsis phenotype across time after protocol initiation, for initiation of antibiotics. Bars represent 95% confidence intervals

## P037

### AseptiMab®, a humanized monoclonal antibody candidate immunotherapeutic to effectively treat sepsis

#### R.J. Webber^1^, R.M. Sweet^2^, D.S. Webber^1^

##### ^1^Research & Diagnostic Antibodies, Las Vegas, USA; ^2^Renal Department, School of Medicine, University of California, San Francisco, USA

*Critical Care* 2021, **25**(**Suppl 1**): P037

**Introduction**: Circulating inducible nitric oxide synthase (iNOS) was discovered by us, and it is an accurate and predictive biomarker for the onset of sepsis [1]. Our further discoveries include (a) circulating extracellular microvesicle-associated iNOS (MV-A iNOS) and (b) MV-A iNOS's putative role of causing the cellular damage, vascular leak, and organ dysfunction that are the hallmarks of sepsis by its production of toxic quantities of nitric oxide (NO). We developed a humanized anti-MV-A iNOS monoclonal antibody candidate immunotherapeutic, AseptiMab®, to specifically target circulating MV-A iNOS and to neutralize its harmful effects.

**Methods**: The ability of AseptiMab to inhibit the sepsis cascade and rescue challenged animals from sepsis was tested in three different animal models of sepsis. One model used an LD80 dose of endotoxin (LPS); another model used an LD80 dose of TNF-α, and the third model used an LD80 dose of MV-A iNOS. At 0, 2, or 6 h after the animals were challenged, they were administered either saline (as placebo controls), a low dose of AseptiMab, or a high dose of AseptiMab.

**Results**: In all three animal models of sepsis tested, AseptiMab was effective at rescuing challenged animals from sepsis compared to the saline control groups. The Kaplan–Meier curves conclusively demonstrate that treating the challenged animals with AseptiMab was effective. Up to 80% of the challenged animals can be rescued from sepsis by AseptiMab. Its effectiveness was both time and dose dependent: earlier and higher doses result in improved survival. If a high dose was administered early after the challenge, all the animals could be rescued.

**Conclusions**: In three different animal models of sepsis, AseptiMab effectively treated the sepsis pathology as compared to placebo.

**Reference**

1. Webber RJ et al. J Appl Lab Med 3:698–711, 2019.

## P038

### Early outcomes and intensive care unit length of stay with rezafungin once-weekly echinocandin in invasive candida disease

#### A. Soriano^1^, S. Dickerson^2^, A. Das^3^, T. Sandison^3^, G. R. Thompson^4^

##### ^1^Department of Infectious Diseases, Hospital Clínic de Barcelona, IDIBAPS, University of Barcelona, Barcelona, Spain; ^2^Mundipharma, Cambridge, UK; ^3^Cidara Therapeutics, San Diego, USA; ^4^University of California Davis Medical Center, California, USA

*Critical Care* 2021, **25**(**Suppl 1**): P038

**Introduction**: The Phase 2 double-blind randomized STRIVE trial (NCT02734862) examined the efficacy of rezafungin, a next-generation once-weekly echinocandin demonstrating a prolonged half-life and high front-loaded plasma exposures, in the treatment of candidemia and invasive candidiasis (IC). Early treatment outcomes and length of stay (LOS) in the intensive care unit (ICU) are presented.

**Methods**: Adults (≥ 18 years) with candidemia and/or IC were randomized to receive rezafungin 400/400 mg (400 mg once-weekly), rezafungin 400/200 mg (Week 1: 400 mg; 200 mg once-weekly thereafter), or caspofungin (Day 1: 70 mg; 50 mg daily thereafter) for ≤ 4 weeks. Day 5 overall cure and mycological success rates were evaluated. Post hoc analysis examined time to negative blood culture, blood culture clearance at 24 and 48 h, and ICU LOS (based on discharge from ICU excluding discharge due to death) data.

**Results**: The percentages of patients with negative blood cultures are presented in Table 1 along with early overall and mycological outcomes. At enrolment, 44% (80/183) of subjects were in the ICU, with a further 6% (11/183) admitted during the trial (microbiological intention-to-treat [mITT] population). Table 1 shows median ICU LOS data for each study arm.

**Conclusions**: Early treatment efficacy of rezafungin was demonstrated by the outcomes of negative blood culture at 24 and 48 h and day 5 overall response rate. A shorter ICU median LOS was also observed in the rezafungin group. These findings support the high front-loaded plasma exposure as a pharmacometric determinant of efficacy in patients with candidemia or IC in the ICU.

**Table:** **Table 1** **(abstract P038)** Early treatment outcomes and ICU length of stay in the STRIVE Trial (mITT population)

Parameter	Rezafungin 400/400 mg (N = 76)	Rezafungin 400/200 mg (N = 46)	Caspofungin 70/50 mg (N = 61)
Negative blood culture at 24 h, % (n/N)	74.1 (43/58)	73.7 (28/38)	54.0 (27/50)
Negative blood culture at 48 h, % (n/N)	86.0 (49/57)	86.1 (31/36)	65.3 (32/49)
Mycological Cure (Day 5), % (n)	65.8 (50)	76.1 (35)	62.3 (38)
Overall success (Day 5), % (n)	55.3 (42)	73.9 (34)	55.7 (34)
ICU patients, % (n)	31.6 (24)	39.1 (18)	37.7 (23)
Median length of stay in the ICU, days (Min, Max)	13.0 (1–79)	13.0 (2–48)	18.0 (1–61)

## P039

### Corticosteroids versus COVID-19 patients admitted to the intensive care unit (ICU): real decrease in in-hospital mortality?

#### F. Righetti^1^, E. Colombaroli^2^

##### ^1^Intensive Care Unit, Emergency Department, San Bonifacio, Verona, Italy; ^2^Intensive Care Unit, San Bonifacio, Verona, Italy

*Critical Care* 2021, **25**(**Suppl 1**): P039

**Introduction**: The use of corticosteroids in the treatment of COVID-19 patients is a matter of debate. The available evidence is uncertain and knowledge on the subject is evolving [1]. The purpose of our observational retrospective study is to evaluate the association between corticosteroid therapy and hospital mortality in COVID-19 positive ICU patients after balancing for possible confounding factors.

**Methods**: A total of 164 COVID-19 positive patients admitted to ICU were included, divided into two groups: 110 patients (Group A) received corticosteroid therapy while 54 (Group B) did not. The daily dose was 8 mg of dexamethasone for a duration of 7 days subsequently reduced until discontinuation.

**Results**: In group A, 28 patients (25%) died while in group B 15 patients (27%). In-hospital mortality was similar between the two groups after adjustment for possible confounding factors (ORadj 1.09, 95% CI 0.78–1.46, p = 0.69). However, corticosteroid exposure was not associated with in-hospital mortality after balancing with the overlapping weight propensity score (adjusted p = 0.2).

**Conclusions**: Corticosteroid treatment did not affect hospital mortality in ICU patients admitted to COVID-19 after balancing confounders. A possible advantage of corticosteroid therapy could be that of reducing ICU admissions when administered to all COVID-19 positive patients with respiratory failure who access the emergency room.

**Reference**

1. Yang X et al. Lancet Respir Med 8:475–481, 2020.

## P040

### Intravenous immunoglobulins decrease the incidence of bacterial secondary infections in severe COVID-19 patients

#### M. Benlabed^1^, S. Benlabed^2^, R. Gaudy^3^, S. Nedjari^4^

##### ^1^Anesthesiology, Lille University, Lille, France; ^2^Internal Medicine, Free University of Brussels, Brussels, Belgium; ^3^Intensive Care, Lille University, Lille, France; ^4^Anesthesiology and Intensive Care, Algiers University, Algiers, Algeria

*Critical Care* 2021, **25**(**Suppl 1**): P040

**Introduction**: Secondary bacterial infections in COVID-19 patients are associated with increased mortality [1]. So, several guidelines advocate the use of empirical antibiotics for patients with severe COVID-19. The aim of our study was to evaluate the administration of intravenous immunoglobulins (IVIG) combined with antibiotics to decrease the incidence (I) of superinfections in severe COVID-19 patients mechanically ventilated for ARDS.

**Methods**: We performed a RCT from January 2021 to May 2021. We enrolled 40 patients, 65 ± 12 years old, admitted in a teaching hospital ICU. We randomized 2 groups of 20 patients: A first group receiving broad spectrum antibiotics (ANT) and IVIG and a second group (grp) receiving the same antibiotics and placebo (C). IVIG were administered after the first dose of ANT, at a dose of 0.5 g/kg at flow of 3 ml/kg/h during the first 3 days. We recorded in the two groups: SOFA score Day 1 and Day 7, PaO_2_/FiO_2_ day 1 and day 7, duration of mechanical ventilation, ICU stay, hospital length of stay (LOS), the I of secondary bacterial infections at Day 14 and 28-day mortality.

**Results**: In IVIG grp, the mean I of superinfections was less than in the C grp respectively 13.5% vs 26% (Table 1). The I of blood stream infections was reduced in IVIG grp comparatively to C grp respectively 8% vs 14.9%. The I of ventilation-associated pneumonia was reduced in IVIG grp comparatively to C grp respectively 20% vs 40%. The I of Gram + infections was reduced in IVIG grp comparatively to C grp respectively19% vs 37%. Mean SOFA score day 7 increased in C grp comparatively to IVIG grp respectively 7.33 ± 0.72 vs 4.45 ± 0.50, p < 0.003. PaO_2_/FiO_2_ at day 7 improved in IVIG grp comparatively to C grp respectively 310.8 ± 7 vs 132.4 ± 4.5, p < 0.003. ICU stay (days) was more elevated in C grp comparatively to IVIG grp, respectively 30 ± 6 vs 18 ± 7, p < 0.001.

**Conclusions**: Early initiation of high doses IVIG with ANT decrease the I of superinfections and are associated with better outcome in severe COVID-19 patients.

**Reference**

1. Liu X et al. Front Immunol 11:1660, 2020.

**Table:** **Table 1** **(abstract P040)** IVIG vs placebo in severe COVID-19 patients

	**IVIG-ANT**	**PLACEBO-ANT**	**p**
Mean SOFA score Day 7	4.45 ± 0.5	7.33 ± 0.72	< 0.003
PaO_2_/FiO_2_	310.8 ± 7	132.4 ± 4.5	< 0.0001
ICU stay	18 ± 7	30 ± 6	< 0.001
LOS	40 ± 12	60 ± 10	< 0.0001
Mean incidence of superinfections day 14	13.5%	26%	
28-day mortality	50%	75%	

## P041

### Hemoperfusion with the Efferon CT extracorporeal adsorbers containing mesoporous styrene–divinylbenzene copolymer (SDC) in patients with severe COVID-19

#### M. Magomedov^1^, T. Kim^1^, S. Masolitin^1^, A. Yaralian^1^, E. Kalinin^1^, V. Pisarev^2^

##### ^1^Pirogov City Clinical Hospital No. 1, Moscow, Russian Federation; ^2^Federal Research and Clinical Center of Intensive Care Medicine and Rehabilitology, V.A.Negovsky Institute of General Reanimatology, Moscow, Russian Federation

*Critical Care* 2021, **25**(**Suppl 1**): P041

**Introduction**: Direct extracorporeal removal of inflammatory mediators with various adsorbents has been suggested as a novel treatment modality for COVID-19 patients [1]. Our study determined safety, feasibility and effectiveness of clinical use of a hemoperfusion (HP) with a novel SDC adsorber to remove pro-inflammatory molecules from the bloodstream of COVID-19 patients.

**Methods**: A total of 42 COVID-19, PCR + patients, age < 81 years, average 63 (SD range, 54–69) years, 52% men, lethality 48%, underwent standard treatment for COVID-19. On day 0, patients exhibited (median and IQR): PaO_2_/FiO_2_ 160 (130–200); APACHE II 18 (18–20); SOFA 6.5 (6–8); IL-6 841 (709–1424) pg/ml, CRP 109 (84–140) mg/l; ferritin 628 (547–678) µg/l. Group 1 (n = 29) continued to receive standard treatment whereas group 2 (n = 13) received HP procedure once, for 3–4 h, using Efferon CT adsorbers containing mesoporous SDC beads uptaking 6–60 kD molecules [2, 3] followed by continuous veno-venous hemodiafiltration. Groups matched on age and sex; group 2 included more severe patients requiring HP support. Data were treated using R-statistics by XLStat.

**Results**: Despite significant intergroup differences in disease severity as shown by SOFA, ALT, AST, SpO_2_, pH, lactate, LDH, and IL-6 alterations on day 0, the HP treatment resulted in no difference in lethality between groups. Patients on HP experienced pH increase and rapid decline of SOFA and lactate as covariates most contributed to lethal outcome; IL-6 and ferritin levels significantly decreased vs. group 1 (Fig. 1). Decreased lethality by HP was significant in a subset of patients on mechanical ventilation exhibiting SOFA < 9 (p = 0.024 vs. group 1).

**Conclusions**: Anti-cytokine HP with Efferon CT adsorbers is feasible and safe method providing a lifesaving promise for a subset of patients with COVID-19 that warrants extended clinical trials.

**References**

1. Ronco C. et al. Blood Purif 50:17–27, 2021.
2. Tsyurupa MP et al. React Funct Polym 53:193–203, 2002.
3. Bessonov IV et al. WO 2018/217137 Al, 2018

**Figure:**
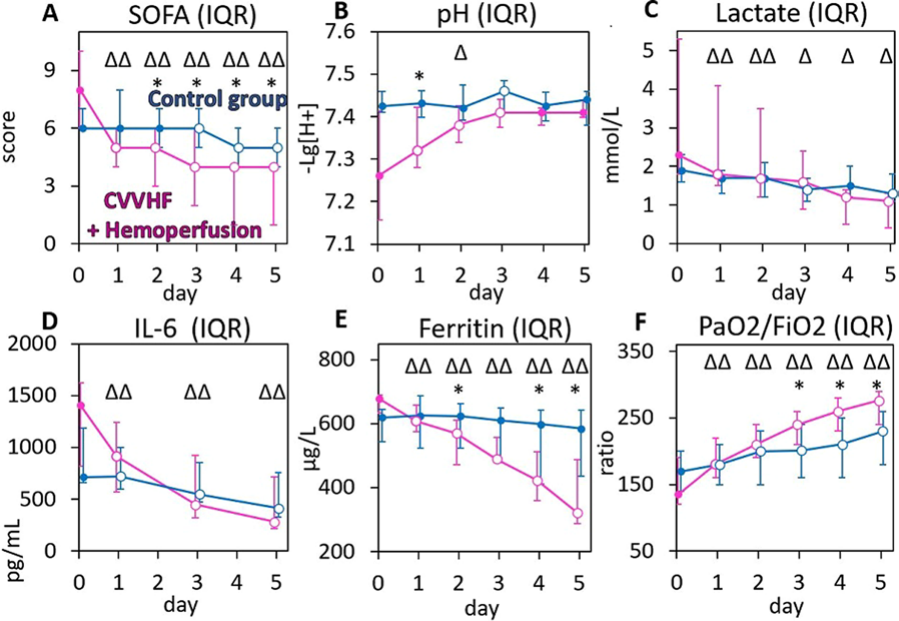
**Fig. 1** **(abstract P041)** Dynamics of covariates following hemoperfusion (HP) with Efferon CT of severe COVID-19 patients. A-C: covariates contributed most (> 30%, p < 0.005) to lethality in the cohort (n = 42), logistic regression model. D-E: other clinically significant covariates recovered following HP. * - p < 0.05, Mann–Whitney U test, HP (pink line) vs. no HP (blue line). Δ - p < 0.05, Δ Δ - p < 0.001, Mann–Whitney U test for relative changes (from day 0 to day N) of variables, HP (pink line) vs. no HP (blue line)

## P042

### Estimating the effects of single-patient use electrocardiogram monitoring as a means of infection prevention in saphenous vein grafts through a healthcare model

#### R. Saunders^1^, E. Pervolaraki^2^

##### ^1^Coreva Scientific, Königswinter, Germany; ^2^Cardinal Health, High Wycombe, UK

*Critical Care* 2021, **25**(**Suppl 1**): P042

**Introduction**: Saphenous vein grafts (SVGs) are common during coronary artery bypass graft surgery (CABG). Surgical site infections (SSI) are rare but potentially dangerous and costly adverse events. SSI rates during these procedures were assessed for multiple UK healthcare centers and a published health-economic model was used to estimate the associated burden of these events. Single-patient use electrocardiogram (spECG) monitoring may contribute to the prevention of cross contamination and help reduce the risk of an SSI. The cost-saving potential of spECG was also assessed.

**Methods**: NHS digital data for SVGs (K401-K404) taking place between March 2019 to February 2020 were assessed for SSIs (T814/T826/T827/T846) occurring during the index event or in the 90 days post-discharge. We included 20 centers with > 300 procedures. Combined outcomes data were used to update a published health-economic model of the CABG care pathway. SSI burden is reported as additional length of stay (LOS), readmissions, and cost.

**Results**: A combined 11,770 SVG procedures were reported across the 20 centers. SSIs occurred in 6.1% (716) of procedures, which increased LOS from 12.2 to 30.0 days. The 351 SSI-related, post-discharge readmissions had a mean LOS of 11.6 days. Based on the inputs above, the estimated cost of care was £8,502 per patient; closely aligned to official reports of £7,830 to £8,784.^1^ Introduction of spECG was estimated to reduce the cost of care to £8,372 per patient; a saving of £130 per patient. This translates to more than a tenfold return on investment. The main drivers for these savings were fewer SSIs, resulting in reduced LOS and fewer readmissions. Individual hospital savings depended on the SSI rate reported.

**Conclusions**: The model results estimate that the routine use of spECG may reduce the cost burden of SSIs following CABG using the SVG method.

^1^R040X: Specialty group costs - inpatients in all specialties (exc long stay), cardiac surgery data used.

## P043

### Significance of admission troponin I in critically unwell COVID-19 patients

#### E.H. Hughes, J.A. McBurney, N.M. Robin

##### Intensive Care Unit, Countess of Chester Hospital, Chester, UK

*Critical Care* 2021, **25**(**Suppl 1**): P043

**Introduction**: Troponin I (TnI) is a suggested predictor of mortality in patients with COVID-19 [1, 2]. Studies highlight differences in mortality and intensive care unit (ICU) admissions between sexes [3]. We aimed to assess correlation between ICU admission TnI measurements and mortality, acute cardiac events, and abnormal echocardiogram (ECHO) results in patients with COVID-19. We hypothesize with increasing admission TnI, subsequent rates of mortality and cardiac events will increase.

**Methods**: A retrospective analysis of online notes of 48 COVID-19 ICU patients between March and July 2020. 99th percentile upper limit of normal range was used for TnI. Admission TnI levels and subsequent outcomes were compared between patients. Proportions for categorical variables were compared using the Fisher exact test in Microsoft Excel.

**Results**: 41 patients had admission TnI measured. The patient male:female ratio was 31:10. The median patient age was 58. Peak admission TnI levels for patients were 8662.9 for men and 27.9 for women. Median admission TnI was 18.84 for males and 7.75 for females. The interquartile ranges between admission TnI were 20.05 and 4.625 for males and females respectively. Figure 1 shows the percentage of patients against measured outcomes, with admission TnI.

**Conclusions**: This small study contributes to the body of evidence that supports TnI as an indicator for outcomes in COVID-19 patients. Those with a high admission TnI have statistical significance with cardiac events during their ICU stay. There is a trend towards a higher admission TnI being associated with higher mortality, although there was no statistical significance.

**References**

1. Nie S et al. Circulation 142:608–610, 2020
2. Du R et al. Eur Respir J 55:2,000,524, 2020
3. Gebhard C et al. Biol Sex Differ 11:29, 2020

**Figure:**
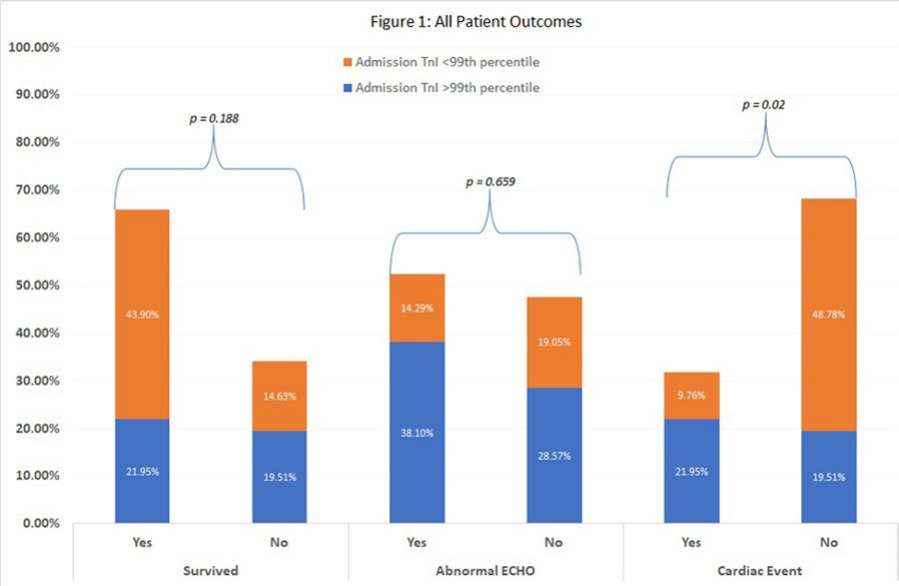
**Fig. 1** **(abstract P043)** Percentage of patients against measured outcomes, with admission TnI

## P044

### Association between dyspnea and right ventricular dysfunction in patients with isolated mitral stenosis

#### R. Benmalek, S. Abouradi, K. Badaoui, H. Bendahou, E. Aqli, E. Aqli, I. Azanai, I. Azanai, S. Serbout, L. Azzouzi, R. Habbal

##### Cardiology, University Hospital Ibn Rochd, Casablanca, Morocco

*Critical Care* 2021, **25**(**Suppl 1**): P044

**Introduction**: Patients with severe mitral stenosis (MS) can present with different degrees of symptomatology, they can be asymptomatic, mildly symptomatic or have severe dyspnea. The right ventricular (RV) systolic function is an important determinant of clinical symptoms, exercise capacity, preoperative survival, as well as postoperative outcomes in patients with MS. The aim of our study was to compare RV function in symptomatic and asymptomatic patients with isolated MS.

**Methods**: We led a prospective observational study over a period of 2 years from October 2018 to January 2021, collected from the day hospital and echocardiography vacations in the cardiology department of Ibn Rochd University Hospital of Casablanca. We included all patients with pure severe and very severe MS, divided into two groups according to their functional capacity: Group 1 (asymptomatic group) with NYHA class I and group 2 (symptomatic group) with Dyspnea stage > II. The RV systolic function was evaluated by both Doppler tissue imaging and pulsed Doppler. A RV dysfunction was defined by a TAPSE < 16 mm and S’ < 9.5 cm/s according to the current recommendations.

**Results**: The study included 468 patients; among them, 145 patients had severe isolated MS, mean age was 42.1 years (± 14.3); sex ratio M/F was 0.8 and women were found more symptomatic than men (84.4% versus 69.23%). Group 1 consisted of 44 patients (30.3%) versus 101 patients in group 2 (69.7%). RV was dilated in 31.81% patients of the first group versus 22.77% of group 2. RV systolic dysfunction was found in 29.7% of symptomatic patients versus 22.7% in asymptomatic patients (p = 0.02).

**Conclusions**: Patients with an impaired RV function are more symptomatic than those with better RV systolic function, thus proving the importance of the RV function assessment in such patients.

## P045

### Evaluation of the prognosis role of obesity in pulmonary embolism in a Moroccan population

#### R. Benmalek, S. Zanouaki, H. Bendahou, Y. Ettagmouti, I. Nouamou, S. Arous, M. E. Benouna, A. Drighil, L. Azouzi, R. Habbal

##### Cardiology, University Hospital Ibn Rochd, Casablanca, Morocco

*Critical Care* 2021, **25**(**Suppl 1**): P045

**Introduction**: Obesity is associated with many cardiovascular risk factors that could trigger venous thrombo-embolism such as venous stasis and decreased mobility. Its prevalence is quickly rising in developed countries and all over the world. Obesity is known to be an independent risk factor for pulmonary embolism (PE). However, few studies have studied the prognosis role of this parameter in PE.

**Methods**: We conducted a retrospective study of 308 patients hospitalized between July 2017 and February 2021 for acute pulmonary embolism in the cardiology department of University hospital Ibn Rochd of Casablanca. We divided our populations into 2 groups depending on their body mass index (BMI): Group 1 (G1) with obesity: BMI > 30 kg/m^2^, and group 2 (G2) without obesity BMI < 30 kg/m^2^. We compared their clinical, electric, echocardiographic and prognosis data.

**Results**: Mean age was 54.3 ± 16.77, sex ratio = 0.3. 28% of patients were in G1. Clinically, 56% in G1 versus 43% in G2 had dyspnea, 20% vs. 10% had edema, 12% vs. 2% had hemoptysis and 10% vs. 3% presented with signs of cardiogenic shock (p < 0.001). ECG signs of acute cor pulmonale were not more frequent in G1 than in G2. Diagnosis was assessed by echocardiography and Chest Computed tomography angiography (CTA). A cardiac thrombi was found in 8% vs. 2%, right ventricular dysfunction in 24% vs. 15% (p = 0.016). Paradoxal septum and pulmonary hypertension occurred in more than 56% of G1 vs. 34% in G2 (p = 0.004). Thrombolysis was used in 4% of G1, unlike patients in G2 who didn’t undergone thrombolysis (p < 0.001). In-hospital mortality was not significantly different between the 2 groups (p = 0.17).

**Conclusions**: In our study, patients with obesity presented with more severe symptoms with more cardiogenic shock and had more significant echocardiographic signs of PE than patients with no obesity. However, the difference of in-hospital mortality was not significant in our study.

## P046

### Efficacy and safety of postoperative use of morphine, fentanyl and remifentanyl after coronary artery bypass grafting

#### D. Loncar Stojiljkovic^1^, M. P. Stojiljkovic^2^

##### ^1^Institute for Cardiovascular Surgery Dedinje, Belgrade, Serbia; ^2^Faculty of Medicine, University of Banja Luka, Pharmacology, Toxicology and Clinical Pharmacology, Banja Luka, Bosnia and Herzegovina

*Critical Care* 2021, **25**(**Suppl 1**): P046

**Introduction**: Pain after cardiac surgery is significant and may affect postoperative cardiovascular stability and may develop into chronic pain thereafter [1]. This postoperative pay should be carefully managed. The aim of this study was to compare the analgesic effects and safety of morphine, fentanyl and remifentanil in patients after coronary artery bypass grafting (CABG) surgery.

**Methods**: Study was prospective, randomized and double-blinded. Forty-five patients undergoing coronary artery bypass surgery were included in the study. All patients received a standardized anesthesia. They were randomized into 3 groups consisting of 15 patients each. M group received morphine HCl (1 mg/ml) with an infusion rate of 0.3 mg/h and 1-mg bolus doses; F group received fentanyl (50 µg/ml) with an infusion rate of 1 µg/kg/h and 10-µg bolus; and R group received remifentanil (50 µg/ml) with an infusion rate of 0.05 µg/kg/min and 0.5-µg/kg bolus, respectively. Continuous infusion was started immediately after transfer to ICU.

**Results**: Pain was assessed by using a numerical scale (0–10) and sedation was assessed with the Ramsey sedation score (1–6) 30 min, 1, 2, 4, 12, and 24 h after extubation. The number of boluses and demands, time to extubation and side effects were analyzed. Numerical scale for pain scores, sedation scores and mean extubation times were similar in all groups. Total number of boluses and demands were statistically higher in the R group. Nausea and vomiting occurred more often in group M group (p < 0.05), whereas pruritus was most frequently registered in group F (p < 0.05).

**Conclusions**: Despite the different durations of action of these three opioid agents, the infusion dose of remifentanil was as effective as morphine and fentanyl after CABG surgery, with fewer side effects.

**Reference**

1. de Hoogd S et al. Trials 15:466, 2014

## P047

### QTC prolongation in critically ill patients with SARS-CoV-2 infection

#### K. Abdallah

##### ITU, West Hertfordshire Hospital, Cambridge, UK

*Critical Care* 2021, **25**(**Suppl 1**): P047

**Introduction**: Several reports linked the use of repurposed drugs such as hydroxychloroquine, azithromycin and lopinavir /ritonavir with QT prolongation in patient with SARS-CoV-2.

**Methods**: Conducting retrospective analysis of critically ill patient admitted to ICU with prolonged QT interval definded as QT more than 500 ms. Patient demographics, baseline characteristics, laboratory values and medications known or suspected to prolong QT interval were collected.

**Results**: Out of the 111 critically ill patients with SARS-CoV-2 infection, Qtc was significantly prolonged in 47 of them, patient with history if cardiac disease/surgery showed higher proportion of significant prolonged QTc. Additionally patient with hypokalemia, male patient had higher degree of QT prolongation. Respectively a total of 46 patient received HCQ, 28 received lopinavir and 5 received azithromycin. Multivariate regression analysis showed that cardiac disease was the only independent factor associated with significant prolonged QTc.

**Conclusions**: The prevalence of clinically significant QTC prolongation in critically ill patient with SARS-CoV-2 was high and independent of drug used.

## P048

### Evaluation of the prognosis of anemia in patients with acute coronary syndrome: a Moroccan center experience

#### R. Benmalek, H. Choukrallah, I. Nassour, Y. Hamine, Y. Hamine, Z. Ammouri, I. Nouamou, S. Arous, M. Benouna, R. Habbal

##### Cardiology, University Hospital Ibn Rochd, Casablanca, Morocco

*Critical Care* 2021, **25**(**Suppl 1**): P048

**Introduction**: Anemia is a well-known marker as an independent risk factor in coronary patients. The aim of our work was to determine the relationship between hemoglobin levels and the evolution of patients admitted into the cardiology intensive care department at the Ibn Rochd University Hospital.

**Methods**: We prospectively included 364 patients admitted to the cardiology intensive care unit of Ibn Rochd Hospital in Casablanca between September 2019 and January 2021 for ACS with or without persistent ST segment elevation (STEMI n = 128 and NSTEMI n = 236). The patients were subdivided into 2 groups: group 1 (n = 119) with a hemoglobin level < 11 g/dl and group 2 (n = 245) with a hemoglobin level > 11 g/dl.

**Results**: Among the 364 included patients, the average age was 60.27 ± 12.35 years, with a male predominance (64.7% of the population). The anemic subjects were older than the non-anemic: 63 ± 9.9 years versus 57 ± 11 years. The renal function was more impaired in anemic patients with a clearance of creatinine to 54.66 ml per minute versus 93.62 ml per minute in non-anemic patients. Group 1 patients had more frequently signs of left ventricular (LV) heart failure than group 2 (46% versus 10%). Systolic LV function, however, seemed unaffected by this rate with a mean ejection fraction measured in both groups (43% in group 1 versus 45% in group 2). Diastolic function was more often impaired with higher LV filling pressures in anemic patients (66% versus 30%). The mean length of stay was longer for patients with anemia (12.5 ± 10.1 days versus 7.9 ± 4 days).

**Conclusions**: Anemia at admission for ACS is linked to a pejorative prognosis. A fairly strong relationship is found between low values of hemoglobin and the evolution of patients especially in terms of clinical or latent heart failure.

## P049

### Evaluation of the prognostic value of diastolic dysfunction in the acute phase of myocardial infarction

#### R. Benmalek, A. Maaroufi, A. Asklou, I. Nassour, A. Errami, I. Nouamou, S. Arous, M. E. Benouna, R. Habbal

##### Cardiology, University Hospital Ibn Rochd, Casablanca, Morocco

*Critical Care* 2021, **25**(**Suppl 1**): P049

**Introduction**: During acute myocardial ischemia, left ventricular (LV) systolic dysfunction is accompanied by diastolic dysfunction which sometimes precedes systolic dysfunction. The recent development of Doppler echocardiography has allowed the noninvasive measurement of LV diastolic filling, which correlates well with other measures of LV filling. The aim of our study was to evaluate LV filling pressures (LVFP) and to demonstrate the prognostic value of their elevation in the acute phase of acute coronary syndrome (ACS).

**Methods**: We prospectively included 364 patients admitted to the cardiology intensive care unit of Ibn Rochd Hospital in Casablanca between September 2019 and January 2021 for ACS with or without persistent ST segment elevation. All patients had Doppler echocardiography within the first four days of admission.

**Results**: The average age of our patients was 60.27 ± 12.35 years [28–85 years]. There was a male predominance (64.7% of the population). More than two-thirds of the patients were smokers (76.36%). One-quarter were hypertensive (23.63%), nearly half were diabetic (50.9%), and one quarter of our population had dyslipidemia. Coronary inheritance was found in 7.27% of our patients. 16.36% of our patients had high LVFP. Elevation of LVFP was a significant predictor of mortality (p = 0.01) in addition to the occurrence of major cardiovascular events at 6 months of follow-up in our study population (p = 0.001).

**Conclusions**: The study of diastolic function and especially the evaluation of the LVFP at the acute phase of acute coronary syndrome allows to refine the stratification of patients admitted for an acute coronary syndrome and to identify a group of high-risk patients to benefit from more intensive treatment.

## P050

### Evaluation of the association between C-reactive protein levels and coronary lesions severity and mortality in acute coronary syndromes

#### R. Benmalek, M. Selmaoui, H. Zahidi, F. Karim, F. Karim, H. Karmouchi, I. Nouamou, S. Arous, M. E. Benouna, R. Habbal

##### Cardiology, University Hospital Ibn Rochd, Casablanca, Morocco

*Critical Care* 2021, **25**(**Suppl 1**): P050

**Introduction**: Inflammation is a major component of the response to tissue injury caused by acute myocardial infarction. C-reactive protein (CRP) levels might be a simple marker of the severity of this inflammatory response, providing prognostic information. The aim of this study was to evaluate the association between CRP elevation and coronary lesions severity as well as in-hospital mortality in patients presenting with acute coronary syndrome (ACS).

**Methods**: We prospectively included 364 patients admitted to the cardiology intensive care unit of Ibn Rochd Hospital in Casablanca between September 2019 and January 2021 for ACS with or without persistent ST segment elevation. These patients were divided into two groups: Group 1 (N = 201) patients with CRP > 5 mg/l and group 2 (N = 163) with CRP ≤ 5 mg/l.

**Results**: The average age of our patients was 60.27 ± 12.35 years [28–85 years]. There was a male predominance (64.7% of the population). More than two-thirds of the patients were smokers (76.36%). One-quarter were hypertensive (23.63%), nearly half were diabetic (50.9%), and one quarter of our population had dyslipidemia. The angiographic findings showed multi-troncular lesions in 60% of group 1 patients versus 26% of the second group. After a multivariate analysis, In-hospital mortality and mortality after 3 months of follow-up were significantly higher in group 1 compared to group 2 (p < 0.0001 and p = 0.019 respectively).

**Conclusions**: Our results demonstrate a strong association between CRP on admission and in-hospital mortality after an ACS, thus suggesting that CRP can be a marker of inflammatory response to myocardial ischemia, providing prognostic information.

## P051

### Correlation between dyslipidemia on admission and the severity of acute coronary syndrome: a prospective Moroccan study

#### R. Benmalek, H. Choukrallah, S. Dghoughi, S. Ejjebli, K. Mouammine, I. Nouamou, S. Arous, M. E. Benouna, R. Habbal

##### Cardiology, University Hospital Ibn Rochd, Casablanca, Morocco

*Critical Care* 2021, **25**(**Suppl 1**): P051

**Introduction**: The lipid panel measures should be systematically performed on admission in patients admitted for acute coronary syndrome (ACS). However, few data have examined the predictive performance of these tests in our center. The aim of this study was to assess the prognosis impact of serum lipid levels in patients admitted for ACS in our unit.

**Methods**: We prospectively included 364 patients admitted to the cardiology intensive care unit of Ibn Rochd Hospital in Casablanca between September 2019 and January 2021 for ACS. We divided them into 2 groups: Group 1 (N = 158) defined by lipid abnormalities at admission (CHT > 2 g/l, HDL > 0.65, LDL > 1.8 or TG > 2 g/l) and Group 2 (N = 206) defined by normal values of lipid parameters. We compared their clinical, echocardigraphic, biological, angiographic and evolutionary data.

**Results**: Patients in group 1 were younger than the second group (56.8 years vs 63 years respectively), more often presenting with hypertension (56.2% vs 43%), diabetes mellitus (54.1% vs 33.9%), and dyslipidemia (17.28% vs 1.45%) but with less chronic smokers (35.7% versus 60.7%). LVEF was not significantly different in both groups. Both average HS troponin (1582 vs 897 ng/l) and CRP (84.3 vs 42 mg/l) were higher in group 1. No significant association was found between CHT, TG, LDL-c, LDL/HDL and CHT/HDL ratios, and the Syntax score in our series; however, low HDL-c levels were significantly associated with a high Syntax score > 32 (p = 0.031). Moreover, no difference was noted between the two groups in terms of in-hospital mortality, but mortality after 3 months was significantly highter in the first group (7.8% versus 1.3%, p = 0.037).

**Conclusions**: High lipid levels on admission were predictive of poor outcomes and higher 3 months mortality in patients with ACS. A low HDL-c level was significantly associated with pluritroncular or left main coronary lesions in our population, unlike other lipid parameters that were not correlated to the severity of coronary lesions assessed by the Syntax scoring.

## P052

### Kinetics of glycogen phosphorylase BB after stress testing-induced myocardial ischemia

#### M. Bakula^1^, K. Mucic^2^, M. Bakula^3^, N. Maric^4^, I. Kruljac^5^, G. Milicevic^6^

##### ^1^Department of Endocrinology, Diabetes and Metabolic Diseases, Department of Internal Medicine, Clinical Hospital Sveti Duh, Zagreb, Croatia; ^2^School of Medicine, University of Zagreb, Zagreb, Croatia; ^3^University Clinic for Diabetes and Metabolism Vuk Vrhovac, Clinical Hospital Merkur, Zagreb, Croatia; ^4^Department of Intensive Care, Department of Internal Medicine, Clinical Hospital Sveti Duh, Zagreb, Croatia; ^5^Department of Endocrinology, Diabetes and Metabolic Diseases, Department of Internal Medicine, Clinical Hospital Sestre Milosrdnice, Zagreb, Croatia; ^6^Department of Cardiology, Department of Internal Medicine, Clinical Hospital Sveti Duh, Zagreb, Croatia

*Critical Care* 2021, **25**(**Suppl 1**): P052

**Introduction**: Glycogen phosphorylase BB (GPBB) is a potentially valuable biochemical marker in myocardial ischemia. The aim of our study was to define the kinetics and diagnostic value of GPBB in diagnosing myocardial ischemia using a model of transient ischemia induced by ergometric stress testing.

**Methods**: The study included 30 subjects with a positive ergometric test result and coronary artery disease confirmed by coronary angiography (ischemic group), and 15 healthy volunteers with a negative ergometric test result (control group). Plasma GPBB concentration was measured basally at one point, prior to the stress test, and at 9 points after the stress test for the next 6 h.

**Results**: The kinetics of GPBB was significantly different between the ischemic group and the control group (p < 0.001). Analysis of the GPBB dynamics curve in the ischemic group shows a statistically significant difference in comparison with the basal concentration in the measurement taken after 0.5 h, and is also observed in the measurements taken 1 and 1.5 h after ergometric stress testing, while disappearing in the measurement taken after 2 h (Fig. 1). Analysis of GPBB increase in the ischemic group showed that the median of the highest absolute GPBB concentrations was statistically significantly higher in comparison with the control group (9.34 ng/ml in the ischemic group; 5.90 ng/ml in the control group). Therefore, a GPBB concentration of 6.775 ng/ml was determined as the optimal cut-off value for predicting myocardial ischemia, with 83% sensitivity and 87% specificity.

**Conclusions**: GPBB is a valuable biochemical marker in myocardial ischemia, with high sensitivity and specificity. The optimal timeframe for sampling and predicting recent myocardial ischemia by means of determining GPBB concentration is between 0.5 and 1.5 h after the onset of ischemia.

**Figure:**
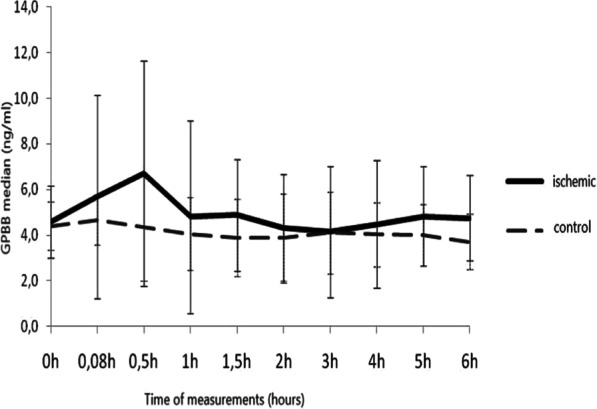
**Fig. 1** **(abstract P052)** Time of measurements of GPBB in ischemic group and in control group

## P053

### Evaluation of the prognosis value of pulmonary embolism severity index in acute pulmonary embolism: experience of the Ibn Rochd Cardiology Department

#### R. Benmalek, S. Zanouaki, H. Mechal, K. Mounaouir, I. Nouamou, S. Arous, M. E. Benouna, A. Drighil, L. Azzouzi, R. Habbal

##### Cardiology, University Hospital Ibn Rochd, Casablanca, Morocco

*Critical Care* 2021, **25**(**Suppl 1**): P053

**Introduction**: Pulmonary Embolism Severity Index (PESI) and simple PESI (sPESI) scores are helpful prognosis assessment tools for acute pulmonary embolism (PE) to assess the severity of PE on admission and initiate the proper management. The aim of our study was to evaluate the prognosis value of PESI score in patients admitted for PE in our Unit.

**Methods**: We conducted a retrospective study of 308 patients hospitalized between July 2017 and February 2021 for acute pulmonary embolism in the cardiology department of University hospital Ibn Rochd of Casablanca. Risk stratification of our the patients was performed using the PESI score based on age, sex, history of cancer, heart failure, chronic lung disease, heart rate, systolic blood pressure, respiratory rate, temperature, oxygen saturation and mental status.

**Results**: 308 patients (mean age 54.3 ± 16.7 years, 77.5% female) with a confirmed PE were included in this study. As for risk factors for thromboembolic disease, 23.3% had cancer history, prolonged bed rest in 25%, oral contraception in 14%, obesity in 28.1%. Right ventricular dysfunction was assessed by echocardiography in 41.6% cases and pulmonary hypertension in 66.7%. D-dimer was high in half our patients. The PESI score averaged 98 points. It was class I in 11.6%, class II in 25.8%, class III in 41.6% and class IV to V in 20.8%. Twenty-seven patients were transferred to intensive care units, from which 19 had a class IV to V PESI score. The evolution was marked by the death of 9 patients with class V PESI.

**Conclusions**: The PESI score is significantly correlated with mortality. Our data indicate that the PESI can be used to predict the prognosis of patients with PE and in making decisions in the medical management of these patients.

## P054

### High and intermediate-high pulmonary embolism treated with alteplase in a tertiary hospital acute intermediate care unit

#### A. F. Costa^1^, R. Alves^2^, J. Miguel Maia^3^, M. Bento Ricardo^2^, A. Pinto^2^

##### ^1^Internal Medicine, Hospital Distrital da Figueira da Foz, Figueira da Foz, Portugal; ^2^Intensive Care Unit, Centro Hospitalar e Universitário do Porto, Portugal; ^3^Intensive Care Unit, Centro Hospitalar de Trás-os-Montes e Alto Douro, Vila Real, Portugal

*Critical Care* 2021, **25**(**Suppl 1**): P054

**Introduction**: Systemic fibrinolysis (SF) is well accepted in hemodynamically unstable patients with acute PE. The guidelines advice that SF should be reserved for high risk PE and for those with intermediate risk that evolved with hemodynamic instability.

**Methods**: Retrospective review of acute PE treated with 100 mg rtPA (2010–2019) in an acute intermediate care unit.

**Results**: Revision of demographic, clinical and imaging data, bleeding events and 30 days mortality of 98 patients; 68.4% female, mean age 62.96 y (± 19.43, door-to-needle time 727 min (± 559 min). Minor bleeding: 27 (27.6%), major bleeding: 6 (6.1%). No intracranial or fatal events found. Mortality of 7.1% at 30 days. Comorbidities and VTE risk factors: obesity 39.7%, HF 22.4%,peripherical venous insufficiency 19.4%,COPD 4.1%, active cancer 10%, autoimmune disease 7.1%, major surgery 10.2%, immobilization 17.3%, previous VTE 9.2%. Intermediate-risk PE 65 (66.3%) and high-risk PE 33 (33.7%) according to current guidelines. Initial TTE (documented in 90%): RV dysfunction 88.6%, RV dilation 90.9%. AngioCT: RV/LV coefficient 1.63 mm (± 0.46). High PE are older (69.61 ± 16.99 vs. 59.58 ± 19.84, p = 0.015) and more associated with active cancer (70% vs 29.5%, p = 0.013), higher lactate and urea, lower PaO_2_/FiO_2_ and statistical difference in arterial pH (Table). Dead at 30 days: the former showed lower baseline Hb and arterial pressure, and higher FiO_2_ needs.

**Conclusions**: The authors highlight the absence of ICH in patients undergoing SF and the low percentage of major bleeding, compared to that found in the PEITHO study [1] and in the international registry, ICOPER [2]. This could be explained by the non-overlapping of rtPA with hypocoagulation,which explains our high time door to needle.The authors are convinced that thrombolysis could be considered in asubgroup of patients with intermediate risk PE with tachycardia, lactatemia and left preload compromise, despite the absence of hypotension or shock.

**References**

1. Meyer G et al. N Engl J Med 370: 1402–1411, 2014
2. Goldhaber S et al. Lancet 353: 1386–1389, 1999

**Table:** **Table 1** **(abstract P054)** Comparison between high vs intermediate-risk pulmonary embolism

	**High vs intermediate-risk**	**p value**	**Death by day 30 vs alive at day 30**	**p value**
Systolic arterial pressure at unit admission (mmHg)	108.30 (SD 23.47) vs. 127.71 (SD 17.95)	< 0.001	85.75 (SD 29.87) vs 123.07 (SD 19.59)	< 0.001
Diastolic arterial pressure at unit admission (mmHg	61.74 (SD 13.97) vs. 73.37 (SD 12.53)	0.001	47.75 mmHg (SD 15.71) vs. 70.62 (SD 12.93)	0.001
PaO_2_/FiO_2_ ratio at hospital admission	224.133 (SD 64.448) vs. 281.314 (SD 77.489)	< 0.001	223.82 (SD 93.47) vs. 265.09 (SD 77.03)	0.252
FiO_2_ (%) at hospital admission	38.13 (SD 20.668) vs. 29.66 vs (17.408)	0.057	47.50 (SD 20.39) vs. 31.33 (SD 18.38)	0.041
Lactate initial levels (mmol/l)	2.873 (SD 2.1339) vs. 1.783 (SD 1.2276)	0.013	2.467 (SD 1.583) vs. 2.120 (SD 1.666)	0.622
Hemoglobin (g/dl)	12.60 (SD 1.99) vs. 13.55 (SD 1.86)	0.017	11.50 (SD 2.48) vs. 13.35 (SD 1.74)	0.017
Urea at unit admission (mg/dl)	62.58 (SD 35.24) vs. 47.84 (SD 29.77)	0.046	91.67 (SD 62.09) vs 50.38 (SD 28.10)	0.165

## P055

### Incidence of thromboembolic events in patients who admitted in hospital through emergency department with COVID-19 infection

#### A. Lavasani Rad, A. M. Majeed, L. Rengarajan, U. Kahara, A. Chaudory

##### Queen Elizabeth Hospital Birmingham, Emergency Department, Birmingham, UK

*Critical Care* 2021, **25**(**Suppl 1**): P055

**Introduction**: Thromboembolic event (TE) is a major challenge in COVID19 patients but the data remains inadequate. Published data suggests a variable risk (around 20% prevalence [1]) of TE associated with coronavirus infection. Lack of robust evidence makes it difficult to develop a standardized approach in managing these patients in Emergency Department (ED). In this study, we aim to assess incidence of TE among patients with COVID-19 infection admitted to hospital.

**Methods**: We conducted a single center retrospective study in the ED of Queen Elizabeth Hospital Birmingham, evaluating TE in patients with COVID-19 infection who required hospitalization between March 2020 and March 2021. We only present initial 20% of cases that data analysis conducted electronically.

**Results**: A total of 527 patients included. 43% of patients were between 40 to 65 yrs followed by 23% of patients with age range of 65-80yrs. We performed D-dimmer test in 379 (71%) patients in the ED, 250 (65%) patients had positive D-dimer test with a wide range of levels as described in Table 1. CTPA performed in 22% patients, pulmonary embolism (PE) reported in 34% patients. 2 patients reported to have confirmed deep vein thrombosis (DVT) by Doppler ultrasound scan, although they had no correlation with confirmed PE cases. Only 71% were suspected to have TE and 21 cases had confirmed diagnosis. Overall TE rate was 4% (0.4% DVT & 3.6% PE).

**Conclusions**: We believe our study is the first to discuss TE on patient presentation in the ED. The previous published data suggests overal incidence of TE around 20% [1]. Our initial data shows a lower incidence of TE in ED patients with confirmed COVID-19 in comparison with similar published studies. Robust evidence from ongoing clinical studies is needed to determine the frequency of TE in patients with COVID-19 infections presenting to ED.

**Reference**

1. Malas MB et al. EClinicalMedicine 29:100,639, 2020

**Table:** **Table 1** **(abstract P055)** D-dimer range on positive patients

D-dimer range	number of patients
< 250	129
250–500	116
500–1000	56
1000–2000	32
> 2000	46

## P056

### Comparison of cardiac index trending (ΔCI): estimates by body surface temperatures (BST) combined with biometric data and basic monitoring parameters (CI_CNI) vs. uncalibrated pulse contour analysis (Flotrac and Clearsight) vs trans-cardiopulmonary thermodilution (CI_TD) derived cardiac index

#### J. Mangold^1^, U. Mayr^2^, W. Huber^2^, T. Lahmer^2^

##### ^1^Klinikum Rechts der Isar, II. Med. Klinik, Munich, Germany; ^2^Klinikum Rechts der Isar, Munich, Germany

*Critical Care* 2021, **25**(**Suppl 1**): P056

**Introduction**: BST provides a rough estimate of CI. With non-contact infrared thermometers (“Thermofocus”, Tecnimed) BSTs can be measured more accurately than just by clinical examination. We hypothesized BSTs combined with biometric data and basic monitoring parameters might be able to track changes in cardiac index as well as the Clearsight- or Flotrac-system (both Edwards Lifesciences) using the trans-cardiopulmonary thermodilution derived changes of CI_TD (ΔCI_TD) of PiCCO (Pulsion) as gold-standard.

**Methods**: From 7/2017 till 1/2018 in 31 patients (APACHE II 29 ± 5) 248 data sets were recorded (8 per patient within 24 h). Immediately before transcardiopulmonary thermodilution CI_CS and CI_FT were recorded, BSTs were measured on forehead, forearm (middle and distal), index and great toe, and basic monitoring parameters were recorded and resulted in CI_CNI using multiple regression analysis. After calculating the percentage of changes in CI, trending was assessed with concordance analysis with four quadrant plot, polar plot analysis and polar concordance rate. We established exclusion zones for all ΔCI < 0.1%. Statistics: IBM SPSS 25, Microsoft Excel.

**Results**: Concordance in four quadrant plot was < 95% for all devices and CI_CNI, Polar concordance was < 92% for all devices and CI_CNI, Angular Bias was acceptable for CI_CNI and CI_FT, Radial LoA were acceptable for CI_FT and almost for CI_CNI as in the Table.

**Conclusions**: Concordance rates and polar concordance rates were far too low for all devices and CI_CNI. CI_CNI and CI_FT showed acceptable angular bias (< 5°). CI_FT showed acceptable radial limits of agreement within the ± 30° borders, CI_CNI was close. CI_CS had neither an acceptable angular bias nor acceptable limits of agreement. In contrast to CI_CS, CI_¬CNI provides comparable estimates of changes in cardiac index to CI_FT.

**Acknowledgements**: Accuracy and precision for CI_CNI for a smaller data set were presented at ISICEM 2018.

**Table:** **Table 1** **(abstract P056)** Results

	CI_CNI	CI_CS	CI_FT
Concordance (4QP)	40%	39.4%	39.1%
Angular bias	3.2°	19.8°	4.5°
Radial LoA	31.9°	− 9.53°to 49.15°	± 28°
Polar concordance (PP)	36.2%	30.3%	36.5%

## P057

### Improvement of accuracy and precision of cardiac power index (CPI) calculated from Flotrac- and Clearsight data by using a non-invasive estimate of CI compared to CPI derived from transpulmonary thermodilution (TPTD)

#### J. Mangold^1^, U. Mayr^2^, W. Huber^2^, T. Lahmer^2^

##### ^1^Klinikum Rechts der Isar, II. Med. Klinik, Munich, Germany; ^2^Klinikum Rechts der Isar, Munich, Germany

*Critical Care* 2021, **25**(**Suppl 1**): P057

**Introduction**: Non- or semi-invasive systems for hemodynamic monitoring (e.g. Flotrac- & Clearsight-system, both Edwards Lifesciences) do not provide CPIs.

**Methods**: From 7/2017 till 1/2018 in 31 patients (APACHE II 29 ± 5) 248 data sets were recorded (8 per patient within 24 h). We calculated CPI_FT and CPI_CS from Flotrac- and Clearsight data and CPI_TD as gold-standard from CI derived by TPTD (CPI = MAP*CI*k). For MAP_CS Bland–Altman analysis showed acceptable bias (3.8 mmHg) and quite good SD (12.13 mmHg), PE (28.4%) and LoA (-20.1 mmHg to 27.6 mmHg) for MAP_CS leading to minimal deviation in error grid analysis (zone “A”: 79.0%, “B”: 18.5%, “C”: 2.4%, no pairs in zone “D” and “E”). But due to poor results of CI-data of FT & CS (CI_FT: bias 0.03 l/min/m^2^, PE 63.3%, LoA -2.64 to 2.71 l/min/m^2^; CI_CS: bias 0.81 l/min/m^2^, PE 51.4%, LoA -1.36 to 2.98 l/min/m^2^) we hypothesized, that we could improve CPI_FT and CPI_CS by combining their corresponding CIs with a regression derived estimate of CI (CI_CNI, bias 0.035 l/min/m^2^, PE 46.1%, LoA -1.91 to 1.98 l/min/m^2^)) based on BST, biometrics and basic monitoring data (CI_FT_CNI & CI_CS_CNI). Using CI_FT_CNI and CI_CS_CNI we then calculated CPI_FT_CNI and CPI_CS_CNI.

**Results**: CPI_FT had a bias of 0.01 W/m^2^ with LoA from -0.48 to 0.51 W/m^2^ and PE of 63.6%. CPI_CS had a bias of 0.17 W/m^2^ with LoA from -0.23 to 0.57 W/m^2^ and PE of 51.5%. CPI_FT_CNI had a bias of 0.00 W/m^2^ with LoA from -0.33 to 0.34 W/m^2^and PE of 43.2%. CPI_CS_CNI had a bias of 0.03 W/m^2^ with LoA from -0.33 to 0.40 W/m^2^and PE of 47.2%.

**Conclusions**: CPI_FT had smaller and acceptable bias than CPI_CS, but bigger PE and wider LoA (as their corresponding CIs). Using combined information for CI from CI_CNI and FT or CS data improved accuracy of CPI_CS and little for CPI_FT, but precision was still poor for both.

**Acknowledgement**: Derivation and validation of CI_CNI on a smaller data set have been presented at ISICEM 2018, for CI_FT_CNI and CI_CS_CNI at ESICM 2018.

## P058

### Personalized hemodynamics - influence of underlying disease on hemodynamic parameters

#### J. Steibl^1^, S. Rasch^1^, S. Schmid^2^, T. Lahmer^1^, R. Schmid^1^, W. Huber^1^

##### ^1^Klinik und Poliklinik für Innere Medizin II, Klinikum Rechts der Isar der Technischen Universität München, München, Germany; ^2^Klinik für Anästhesiologie und Intensivmedizin, Universitätsklinikum Ulm, Ulm, Germany

*Critical Care* 2021, **25**(**Suppl 1**): P058

**Introduction**: Sepsis and ARDS remain pathologies with high mortality in ICUs, despite decades of advancement. We exploratively investigated hemodynamic monitoring parameters derived from transpulmonary thermodilution (TPTD) and their interrelation among 3 distinct groups of patients with multiorgan failure.

**Methods**: We analyzed data of a prospectively maintained database including 1495 TPTD-measurements (PiCCO; Pulsion; Germany) of 81 ICU-patients in three groups: 30 sepsis (SEP) patients, 18 with pneumonia/ARDS (PUL) and 33 with HRS, GI-bleeding, pancreatitis or other (HBPO). Primary endpoints: TPTD parameters of hemodynamic profile (HI, GEDVI, ELWI), mortality. To create a total PiCCO predictive probability of death score (T3PDS; Min 0, max 15), the last five TPTD measurements of HI > 3, GEDVI > 700 and ELWI < 10 in ICU were analyzed.

**Results**: Patients: 55.6% female, age 67 ± 13.4 y; APACHE II 20.4 ± 7.9, SOFA 10 ± 4.7, SAPSII 43.6 ± 17. In-ward mortality 39.4% HBPO, 50% PUL, 30% SEP, add. postICU mortality in SEP of 13.3% (p = 0.084). SEP group showed a significantly elevated heart rate (p = 0.019), a decreased MAP (p < 0.036) and lower CVP (p < 0.004). PUL and SEP groups showed decreased GEF (p = 0.009) and prolonged ICU stay (p < 0.025). A median split dividing short and long term ICU stays failed to prove a significant difference in mortality among the groups (p < 0.914). The established T3PDS showed a 1.21 × increase for SEP (p = 0.028) and a 1.24 × increase of probability for PUL (p = 0.023) to die with higher scores. The T3PDS cut-off analysis showed a probability of death increase with > 6.4 pts (AUC: 0.86 ± 0.1, 95%, p = 0.021).

**Conclusions**: The study shows that the commonly used algorithms in achieving hemodynamic stability provide a solid therapeutic concept irrespective of the underlying cause. In case of septic patients we recommend early and continuous monitoring in case of typical signs of tachycardia, hypotension and high CVP. We advise to consider a prolonged stay for septic patients. The T3PDS has to be validated in a larger patient population.

## P059

### Impact on the vital signs of COVID-19 intensive care patients during inter-hospital helicopter transfer

#### C. Slagt

##### Anesthesiologie, Pijn en Palliatieve Geneeskunde, RadboudUMC, HEMS Lifeliner 3 en 5, Nijmegen, Netherlands

*Critical Care* 2021, **25**(**Suppl 1**): P059

**Introduction**: The Helicopter Emergency Medical Service (HEMS) Lifeliner 3 of the Radboud University Medical Center organized the first intensive care (IC) helicopter (Lifeliner 5) to transport critically ill COVID-19 patients in the Netherlands. In-hospital, pre-hospital and inter-hospital transfer of IC patients all bear the risk of complications. Most of the complications have impact on patients vital signs [1–3]. The goal of this study is to analyze the impact of the helicopter transfer of COVID-19 IC patients on the vital signs during take-off, midflight and landing.

**Methods**: This prospective observational study was performed during the second COVID-19 outburst in the Netherlands. All inter-hospital helicopter transfers of COVID-19 IC patients who were monitored including non-invasive electrical cardiometry cardiac output (CO) were included in this study. Patients were included from November 2020–June 2021. Three predefined time frames were analyzed. The first time frame of 10 min started with the actual take-off. The second 10 min frame represents mid-flight. The third 10 min time frame ends with the actual landing. In each patient the vital signs including CO data extracted in each time frame were averaged and analyzed.

**Results**: Up to now 89 patients are included in this analysis. Mean age is 62.3 ± 11.5 yr. Seventy percent are male. Mean weight was 91.3 ± 16.0 kg. Body mass index 29.9 ± 5.6. Vital sign data during the three time frames are shown in Table 1. Cardiac output data were available in 83 patients.

**Conclusions**: These preliminary results suggest that helicopter transfers of IC COVID-19 patients have minimal impact on vital signs during take-off, mid-flight and landing.

**References**

1. Beckmann U et al. Intensive Care Med 30:1579–85, 2004.
2. Flabouris A et al. Anaesth Intensive Care 34:228–36, 2006
3. Duke GJ et al. Med J Aust 174:122–125, 2001

**Table:** **Table 1** **(abstract P059)** Vital sign data during the three time frames

	**Take-off**	**Mid-flight**	**Landing**	**p value**
HR (min-1)	72.3 [58.7–84.0]	71.2 [57.2–83.4]	70.9 [57.1–83.3]	0.76
SpO_2_ (%)	93.5 [92.0–95.2]	94.1 [92.3–95.6]	93.5 [92.0–95.2]	0.45
Map (mmHg)	84.5 [75.2–92-9]	82.2 [73.8–89.3]	80.6 [73.0–87.8]	0.23
Et-CO_2_ (mmHg)	38.5 [35.1–42.7]	37.3 [33.9–40.4]	37.4 [34.5–40.5]	0.42
SV (ml)	87.2 [70.7–122.7]	104.6 [82.8–130.6]	99.4 [80.1–128.9]	0.04*
CO (Lmin-1)	6.9 [5.0–8.8]	7.4 [5.7–9.6]	7.3 [5.6–9.7]	0.22
TSVR (dyne*cm-5 m-2)	1017.0 [723.8–1415]	866.0 [668.9–1246]	881.9 [681.9–1173]	0.11

HR = heart rate; SpO_2_ = peripheral oxygen saturation; Map = mean arterial blood pressure; Et-CO_2_ = end tidal carbon dioxide; SV = stroke volume; CO = cardiac output; TSVR = total systemic vascular resistance. Data analyzed using Kruskal–Wallis. p < 0.05 being statistically significant

## P060

### Bedside diagnosis of right mediastinal shift using point of care ultrasound

#### A. M. Kothekar, N. L. Lodh, V. P. Patil

##### Dept of Anaesthesia, Tata Memorial Center, Critical Care and Pain, Mumbai, India

*Critical Care* 2021, **25**(**Suppl 1**): P060

**Introduction**: Mediastinal shift, especially a large one, can cause obstructive shock due to cardiac herniation or twisting of great vessels [1]. Early identification of mediastinal shift may help in initializing rapid therapy for reversal of shift to prevent significant hemodynamic compromise and cardiac arrest. We report two cases of right-sided mediastinal shift identified using Point of Care Ultrasound (POCUS) based on the location of parasternal cardiac window and probe direction of subcostal window.

**Methods**: Case 1. A young adult had sudden hypotension on postoperative day 1 of right pneumonectomy. POCUS failed to obtain cardiac image with left parasternal cardiac window. On further interrogation, parasternal cardiac window was obtained in right second intercostal space. Subcostal cardiac view present towards right side instead of usual left side. This raised suspicion of significant mediastinal shift towards right causing obstructive shock, further supported by dilated inferior vena cava (IVC) and good left ventricular contractility ruling out hypovolemic or cardiogenic shock.

Case 2. Patient was admitted with febrile neutropenia and septic shock following cytotoxic chemotherapy for hemato-lymphoid malignancy. Routine POCUS evaluation indicated similar findings indicating mediastinal shift.

**Results**: Right mediastinal shift was confirmed with urgent chest X rays (Fig. 1). In first case, injection of air in the right pleural cavity through right ICD improved hemodynamic parameter. In second case, mucus plug obstructing right main bronchus was identified and removed with bronchoscopy causing opening up of the collapsed right lung.

**Conclusions**: Right mediastinal shift can be identified on POCUS examination by shifting of parasternal and subcostal cardiac window from left to right. This should prompt a search for cause of mediastinal shift and treatment.

**Reference**

1. Nechala P et al. Ann Thorac Surg 82:1916, 2006

**Figure:**
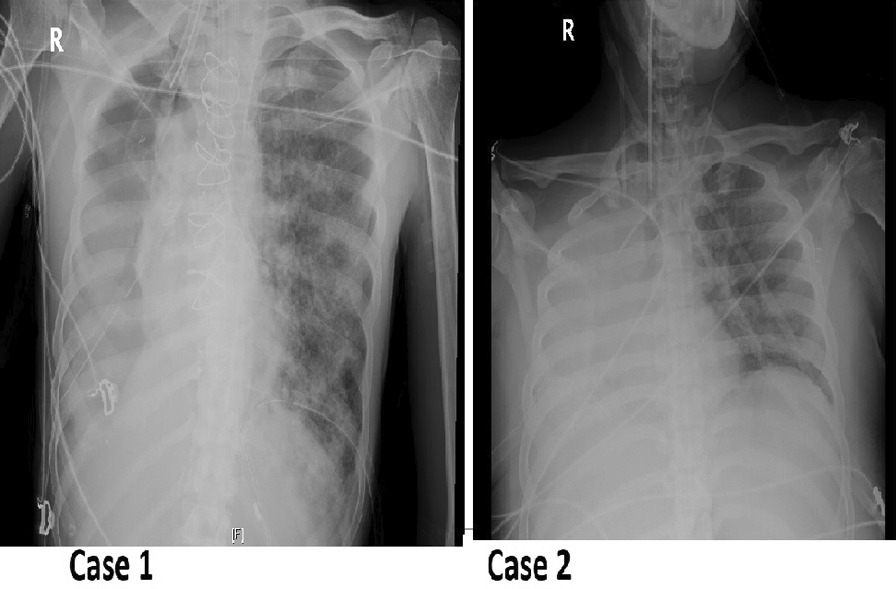
**Fig. 1** **(abstract P060)** Right mediastinal shift

## P061

### Assessment of radial and ulnar artery size and flows in intensive care patients with identification of patients at risk of limb ischemia

#### R. Underwood^1^, N. Abdulla^2^, P. Parulekar^2^

##### ^1^Emergency Department, Medway Maritime Hospital, Gillingham, UK; ^2^ICU, William Harvey Hospital, Ashford, UK

*Critical Care* 2021, **25**(**Suppl 1**): P061

**Introduction**: Radial artery cannulation allows for continuous blood pressure monitoring in Intensive Care Unit (ICU) [1]. Studies have assessed for ischemic complications of radial artery cannulation [2], but there is lack of evidence using ultrasound to determine whether limbs at risk of ischemia are being cannulated and for the presence of collateral ulnar flows. The objective was to assess collateral ulnar flow, compare flow and size of radial and ulnar arteries and to detect limbs at risk of distal ischemia using ultrasound in ICU patients.

**Methods**: A prospective cohort study was conducted at William Harvey Hospital ICU, UK. From 1/12/2019–9/3/2020, data were collected from patients on ICU. Vascular probe ultrasound was used to determine the size of radial and ulnar arteries bilaterally, presence of adequate color flow and flow velocity through each vessel. Complications associated with the arterial line were noted.

**Results**: A total of 53 patients were included. A total of 34 patients had radial arterial lines in situ. The mean longitudinal diameter for radial and ulnar arteries was 0.23 cm and 0.22 cm, respectively, both larger than the internal diameter of cannulae used (0.8 mm). Eighteen patients had at least one at risk limb (≥ 1 artery with anything less than ‘good’ visual flow on color Doppler). 50% had an arterial line present in at risk limb. There were no major complications noted.

**Conclusions**: 24% of patients had at least one ‘at risk’ limb with 15% of upper limbs having moderate/poor collateral ulnar flow. This indicates a significant proportion of patients on ICU at risk of digital ischemia and the need for assessment of radial and ulnar arterial size and flow before cannulation.

**References**

1. Marino P. The ICU Book. 4th ed. Philadelphia: Wolters Kluwer Health/Lippincott Williams & Wilkins, pp.123–134, 2014.
2. Scheer B et al. Crit Care 6:199–204, 2002

**Figure:**
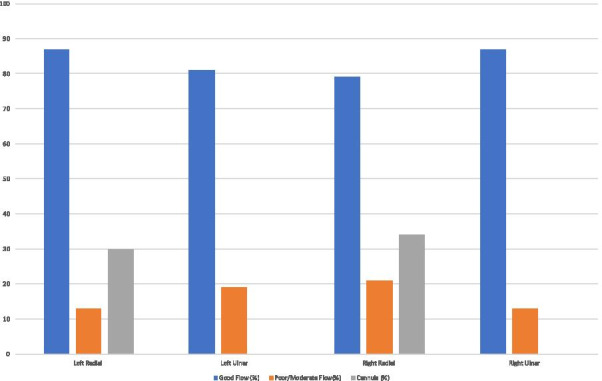
**Fig. 1** **(abstract P061)** Flow in upper limb arteries

## P062

### Cardiac dysfunction in intubated COVID-19 and the effects of inhaled nitric oxide

#### O. W. Wall^1^, D. T. Törnberg^2^, M. H. Hedenstierna^2^, C. S. Svensén^1^, E. J. Joelsson-Alm^1^, M. C. Cronhjort^1^, J. O. Oesman^2^, M. W. Widaeus^2^, K. S. Shahgaldi^2^

##### ^1^Department of Clinical Science and Education, Karolinska Institutet, Södersjukhuset, Stockholm, Sweden; ^2^Department of Clinical Sciences, Karolinska Institutet, Danderyd Hospital, Stockholm, Sweden

*Critical Care* 2021, **25**(**Suppl 1**): P062

**Introduction**: Cardiac involvement of COVID-19 is not fully explored in intubated ICU-patients although findings suggest a link to worsened outcome. To the best of our knowledge there are no prospective studies evaluating changes over time using ultrasound. We aimed to evaluate right ventricular (RV) and left ventricular (LV) systolic function, and resolution of pulmonary hypertension (PH).

**Methods**: We performed a sub-study of a prospective RCT at a Swedish ICU (NO SARS-COVID) examining the effects of inhaled nitric oxide (iNO) on the recovery of ICU-patients treated for COVID-19. Adult patients were included within 72 h of intubation and evaluated by echocardiography at study inclusion and at follow-up 3–5 days after randomization.

**Results**: Thirty-nine patients were included, 33 completed follow-up (Table 1). At inclusion, eight patients had RV dysfunction evaluated by tricuspid annular plane systolic excursion (TAPSE) and eight evaluated by right global longitudinal strain (RVGLS). None had LV systolic dysfunction evaluated by ejection fraction (EF), but 10 evaluated by global longitudinal strain (GLS). Thirty-two patients had signs of PH, median (IQR) mean pulmonary artery pressure (MPAP) 35.3 mmHg (28.0–46.8). At follow-up, none had RV failure evaluated by TAPSE in the control group and 1 in the iNO group, but 3 in the control group and 5 in the iNO group evaluated by RVGLS. No patients in either group had LV systolic failure at follow up evaluated by EF, but GLS unmasked dysfunction in 4 patients in the control group and 1 in the iNO group. All patients in the control group had signs of PH, MPAP 56 (38–62) and 17 in the iNO group, MPAP 39 mmHg (28–54).

**Conclusions**: As expected, PH was frequently seen in our cohort of intubated patients with COVID-19 and did not resolve after administration of iNO. We encourage use of strain analysis to assess chamber function more accurately beside conventional echocardiography parameters to unmask subclinical cardiac dysfunction.

**Table:** **Table 1** **(abstract P062)** Patient characteristics

	**All**	**Inhaled nitric oxide**	**Control**
Age (years)	61 (50–69)	61 (50–69)	60 (49–69)
Male	31 (79%)	17 (81%)	14 (78%)
BMI (kg/m^2^)	29.7 (27.1–34.4)	29.2 (26.6–34.2)	30.8 (27.3–35.0)
SAPS III	66 (58–72)	66.0 (59.0–77.0)	68.0 (57.0–71.0)
APACHE II	31 (30–36)	33.0 (31.0–36.5)	29.5 (26.7–30.0
Vasoactive inotropic score	6 (2–11)	4 (1–10)	8 (3–15)

Values are presented as median with (IQR) or numbers (percentages) of patients. SAPS III = Simplified Acute Physiology Score III. APACHE II = Acute physiology and Chronic Health Evaluation II

## P063

### CVC tip positioning by transthoracic contrast-enhanced echocardiography: a comparison study with two different TTE techniques, CXR and TEE

#### L. Tecchi^1^, R.Biagini^1^, A. Isirdi^2^, E. Taddei^2^, F. Guarracino^3^, F. Forfori^1^, F. Corradi^1^

##### ^1^Department of Surgical, Medical and Molecular Pathology and Critical Care Medicine, Azienda Ospedaliero Universitaria Pisana, University of Pisa, Pisa, Italy; ^2^Azienda Ospedaliero Universitaria Pisana, Pisa, Italy; ^3^Department of Cardiothoracic and Vascular Anaesthesia and Critical Care Medicine, Azienda Ospedaliero Universitaria Pisana, Pisa, Italy

*Critical Care* 2021, **25**(**Suppl 1**): P063

**Introduction**: To determine the usefulness of two different transthoracic echocardiography techinques or chest X-ray, to evaluate central venous catheter misplacements with trans-esophageal echocardiography as reference. After the insertion of a central venous catheter, chest radiograph is usually obtained to ensure correct positioning of the catheter tip.

**Methods**: Ninety-nine consecutive patients undergoing CVC positioning, using a landmark or ultrasound-guided technique. A post-procedural TEE was obtained in all patients and was considered as reference technique. A TTE through the subcostal acoustic window along the short heart axis or through the apical four-chamber view was performed at the end of the procedure in order to infer correct catheter tip position. All patients also underwent CXR.

**Results**: Using TEE as a reference standard, and considering intravascular and intracardiac malpositioning altogether, catheter tip malpositions were detected in 27 patients by TTE through the subcostal acoustic window along the short heart axis, in 8 patients by TTE through the apical four-chamber view and in 17 patients by CXR. For the detection of catheter tip malposition, TTE through the subcostal acoustic window has shown to be by far the most accurate method providing 96% sensitivity, 98% specificity and 96% diagnostic accuracy if compared with the apical four-chamber view TTE or CXR. Concordance with TEE was 94% (p < 0.001).

**Conclusions**: The close concordance between TTE through the subcostal acoustic window and TEE justifies the use of ultrasounds as a standard technique to ensure the correct positioning of the catheter tip after central venous catheter cannulation, in order to optimize use of hospital resources and minimize radiation. CXR will be necessary when sonographic examination is impossible to perform by technical limitations.

**Table:** **Table 1** **(abstract P063)** Diagnostic accuracy and concordance

	**Sensitivity (%)**	**Specificity (%)**	**Accuracy (%)**	**k-Cohen (%)**
CE-TTE subcostal	96	98	96	94
CE-TTE apical	18	97	69	18
CXR	38	94	77	33

## P064

### Reliability of bioreactance and pulse power analysis in measuring cardiac index during cardiac surgery with cardiopulmonary bypass

#### L. Ylikauma^1^, K. Lanning^1^, P. Ohtonen^1^, T. Erkinaro^1^, M. Vakkala^1^, J. Liisanantti^1^, T.Juvonen^2^, T. Kaakinen^1^

##### ^1^Research Group of Surgery, Anesthesiology and Intensive Care Medicine, Oulu University Hospital, Oulu, Finland, ^2^ Department of Cardiac Surgery, Heart and Lung Center, Helsinki University Hospital, Helsinki, Finland

*Critical Care* 2021, **25**(**Suppl 1**): P064

**Introduction**: Measuring cardiac output is essential when treating critically ill patients and patients undergoing high-risk surgery [1]. Pulmonary artery catheter is considered as the gold standard for measuring cardiac output, but it is invasive and can be harmful to the patients [2]. Less invasive monitors have recently been developed. We compared the accuracy, precision and trending ability of non-invasive bioreactance-based Starling SV and mini-invasive pulse power device LiDCOrapid to bolus thermodilution technique with pulmonary artery catheter (TDCO) when measuring cardiac index (CI) in the setting of cardiac surgery with cardiopulmonary bypass (CPB).

**Methods**: Twenty patients undergoing cardiac surgery with CPB were monitored with Starling SV, LiDCOrapid and TDCO intraoperatively in the OR and postoperatively in the ICU resulting in simultaneous 513 CI measurements. We used the Bland–Altman method to investigate the agreement between the devices and four-quadrant plots with error grids to assess the trending ability.

**Results**: The agreement between TDCO and Starling SV was associated with a bias of 0.43 l/min/m^2^ (95% confidence interval, 95% CI, 0.37 to 0.50), wide limits of agreement (LOA, –1.07 to 1.94 L/min/m^2^) and a percentage error (PE) of 66.3%. (Fig. 1a) The agreement between TDCO and LiDCOrapid was associated with a bias of 0.22 l/min/m^2^ (95% CI 0.16–0.27), wide LOA (–0.93 to 1.43 l/min/m^2^) and a PE of 53.2% (Fig. 1b). Trending ability was not acceptable, since with Starling SV only 26% and with LiDCOrapid 39% of measurements changed in the same direction to the same extent as with TDCO.

**Conclusions**: CI measurements with bioreactance-based Starling SV and pulse power device LiDCOrapid were not interchangeable with TDCO, and their ability to track changes in CI was poor. These results do not support their use in monitoring CI reliably during and after cardiac surgery with CPB.

**References**

1. Marik PE. J Cardiothorac Vasc Anesth 27:121–134, 2013
2. Swan HJC et al. N Engl J Med 283:447–451, 1970

**Figure:**
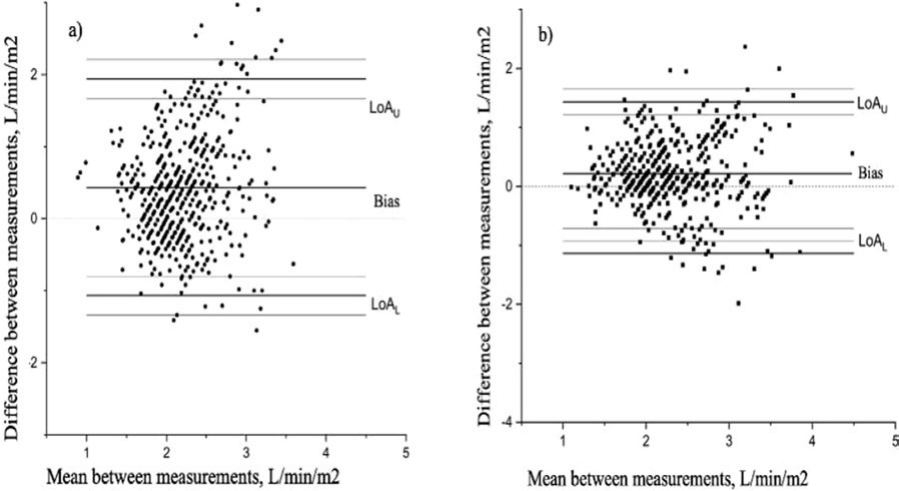
**Fig. 1** **(abstract P064)** **a**The Bland–Altman plot for cardiac index determined by the bolus thermodilution technique with a pulmonary artery catheter and bioreactance-based Starling SV, all measurement points. The lines for bias, limits of agreement (LOA) and 95% confidence intervals are shown. **b**. The Bland–Altman plot for cardiac index determined by the bolus thermodilution technique with a pulmonary artery catheter and pulse power device LiDCOrapid, all measurement points. The lines for bias, limits of agreement (LOA) and 95% confidence intervals are shown

## P065

### Goal directed fluid therapy during major liver resections shows altered sublingual and intestinal microcirculation when compared to low central venous pressure

#### Z. Uz^1^, I. M. Jongerius^2^, L. Shen^1^, T. H. Mungroop^2^, D. P. Veelo^2^, T. M. Van Gulik^3^, C. Ince^4^

##### ^1^Intensive Care Adults, Erasmus MC, Rotterdam, Netherlands; ^2^Anaesthesiology, Amsterdam UMC, University of Amsterdam, Amsterdam, Netherlands; ^3^Surgery, Amsterdam UMC, University of Amsterdam, Amsterdam, Netherlands; ^4^Intensive Care Adults, Amsterdam UMC, University of Amsterdam, Amsterdam, Netherlands

*Critical Care* 2021, **25**(**Suppl 1**): P065

**Introduction**: The effect of low central venous pressure (CVP) or goal-directed fluid therapy (GDT) during major liver resection on organ perfusion is undefined. The present study aimed to assess sublingual and intestinal organ microcirculation during low CVP or GDT fluid management in patients undergoing major liver resection.

**Methods**: In this single-center, surgeon and patient-blinded, randomized controlled trial, patients undergoing major open liver resections (≥ 3 segments) were randomized to receive either GDT or low-CVP. As a substudy of this trial, sublingual and intestinal microcirculation were assessed using the handheld video microscope, IDF imaging. The sublingual and intestinal microcirculations were measured after skin incision (T0) and before skin closure (T1); 24 h after surgery sublingual microcirculation was measured again (T2). Patients’ baseline characteristics, intra-operative parameters and outcomes were analyzed.

**Results**: A total of 38 patients were included for analysis, 18 in the GDT group and 20 in the low-CVP group. Sublingual microcirculation in the GDT group showed a significant decrease in total vessel density 24 h after surgery, and a significant decrease in intestinal microcirculatory density at the end of surgery. The GDT group showed a more positive fluid balance intraoperatively when compared to the low-CVP group. No differences in sublingual and intestinal microcirculation were found in the low-CVP group. Patient outcomes showed no differences between the groups.

**Conclusions**: Perioperative monitoring of organ microcirculation revealed altered intestinal and sublingual microcirculation in the GDT group, whereas the low-CVP group showed no changes. Outcome of patients were similar between the study groups, suggesting recovery of the microcirculation in the GDT group over time. Monitoring of the organ microcirculation is a potential measure to control and evaluate the effects of fluid therapy on the perfusion.

## P066

### Fluid responsiveness assessment by passive leg raising and mini-fluid challenge in major surgery, a prospective study

#### L. Foti^1^, M. Suppressa^1^, G. Iacopetti^1^, G. Villa^1^, Z. Ricci^2^, A. Messina^3^, S. Romagnoli^1^

##### ^1^Department of Anesthesiology & ICU, Azienda Ospedaliero Universitaria Careggi, Florence, Italy; ^2^Department of Anesthesiology & ICU, Azienda Ospedaliero Universitaria Meyer, Florence, Italy; ^3^Department of Anesthesiology & ICU, Humanitas University, Milan, Italy

*Critical Care* 2021, **25**(**Suppl 1**): P066

**Introduction**: The aim of this study was to evaluate fluid responsiveness preoperatively by passive leg raising (PLR), and intraoperatively by mini-fluid challenge (mFC). Guidelines recommend 2-h fasting for clear liquids to ensure euvolemia before surgery. Few methods are available to assess fluid responsiveness in awake spontaneously breathing patients: PLR and mFC are among them. Fluid responders (FRs) are those who increase stroke volume (ΔSV) > 10% after PLR or mFC. The Starling™SV monitor (Cheetah Medical-Baxter) identifies FRs thanks to bioreactance technology.

**Methods**: Preoperative PLR test was performed to patients undergoing major surgery before the anesthesia induction. FRs received 250 ml of balanced crystalloid solution and fluid responsiveness was re-tested at the end of the bolus and again until a non-FR state was achieved. Baseline intraoperative fluid therapy was set at 5 ml/kg/h until the end of surgery. Hourly, mFCs with 100 ml of crystalloids balanced solutions were performed and FRs received the 250 ml-fluid bolus. Blood [Hb]-concentration was measured before surgery and hourly.

**Results**: Twenty-two patients were enrolled. Fifty% of the patients were FRs and average fasting time was 12 (3.16) h in FRs and 13.8 (4.35) h in non-FRs (Fig. 1). After the first crystalloid bolus, 9 (82%) FRs became non-FRs. Intraoperatively, 47 mFCs were performed in the first 3 h and 9 FRs were found (19%). In all FRs, after the bolus, the mFC test was re-performed and no patient resulted to be FR. The [Hb] variation before and after 60 min from surgery start was 0.71 (0.66) g/dl for FRs and 0.32 (0.56) g/dl for non-FRs.

**Conclusions**: Preoperative fasting does not correlate with PLR test. After having tested for fluid responsiveness and corrected volemia, [Hb]-concentration can be used as a sensitive indicator of fluid expansion-restriction in non-bleeding patients. PLR and mFC tests, are easy, reliable and may be efficient tools to guide volume optimization during major surgery.

**Figure:**
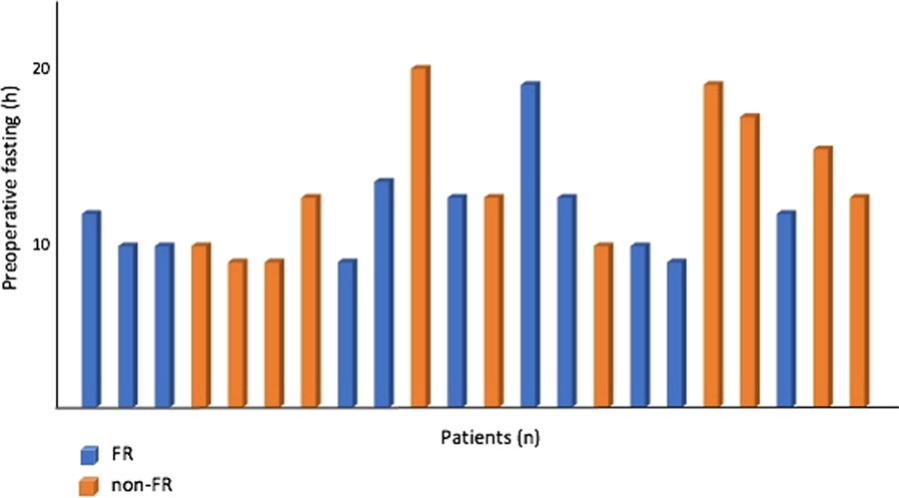
**Fig. 1** **(abstract P066)** Average fasting times

## P067

### Timing of dynamic measurement of stroke volume and patient outcome: results from examination of a large administrative database

#### J. Sahatjian^1^, I. D. Douglas^2^, A. H. Holder^3^, H. L. Latham^4^, G. M. Martin^3^, S. S. Simpson^4^, D. M. Hansell^5^

##### ^1^Baxter Healthcare, Newton Center, USA; ^2^Denver Health Hospital, Denver, USA; ^3^Emory University, Atlanta, USA; ^4^Kansas University, Kansas City, USA; ^5^Massachusetts General Hospital, Boston, USA

*Critical Care* 2021, **25**(**Suppl 1**): P067

**Introduction**: Intravenous (i.v.) fluid resuscitation is a central component of septic shock resuscitation but in excess is associated with adverse effects. Dynamic measurement of stroke volume (SV) following a fluid challenge is a safe and feasible method of rapidly predicting the effectiveness of fluid-induced augmentation of cardiac output. We have previously shown that SV guided dynamic assessment could alter the amount of i.v. fluid administered to patients with septic shock and improve patient outcomes. We sought to evaluate the timing of the initiation of dynamic methods using a large number of patients within the Premier dataset.

**Methods**: We used the 2013–2019 Premier Hospital Discharge database to analyze the timing of initiating dynamic methods to guide fluid administration in 1,123 patients with severe sepsis and septic shock who were admitted to an ICU from the ER. Patients who received dynamic monitoring initiated days 1–2 were compared to those who received dynamic monitoring on days 3 and later.

**Results**: The analysis set consisted of 1123 discharges from 19 hospitals. The mean patient age was 68.6 years of age and 42.2% were female. There was no significant difference in demographics or organ failures (Fig. 1). Notably, patients who received monitoring on days 1–2 exhibited a decreased ICU length of stay (4.5 vs 10.7 days, p < 0.0001) and need for vasopressors (2.5% vs 17.4%, p < 0.0001) and lower risk of death (27% vs 40%, p = 0.001) compared with patients who had monitoring started on Days 3 + ( Fig. 1).

**Conclusions**: The present results suggest that timing of resuscitation efforts is critically important, as patients who received earlier dynamic monitoring exhibited improved outcomes.

**Figure:**
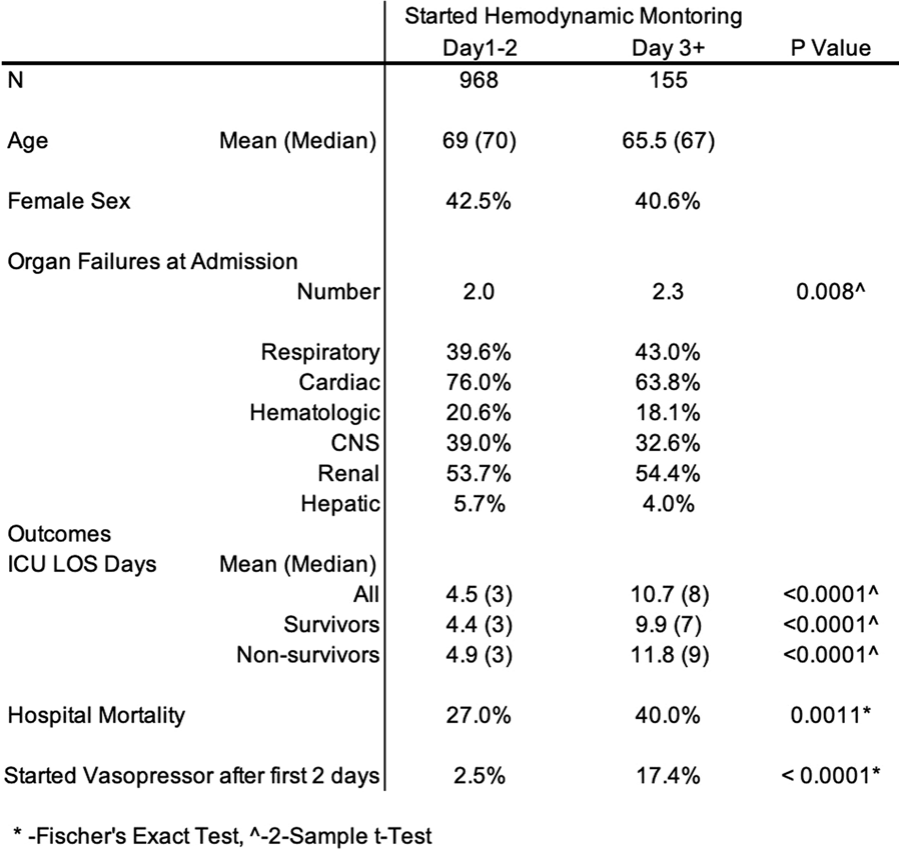
**Fig. 1** **(abstract P067)** Patient characteristics and outcomes

## P068

### Stroke volume variation (SVV): difference of information derived from fingerplethysmography (“Clearsight”), uncalibrated pulse contour analysis (“Flotrac”) and calibrated pulse contour analysis (PiCCO)

#### J. Mangold^1^, U. Mayr^2^, W. Huber^2^, T. Lahmer^2^

##### ^1^Klinikum Rechts der Isar, II. Med. Klinik, Munich, Germany; ^2^Klinikum Rechts der Isar, Munich, Germany

*Critical Care* 2021, **25**(**Suppl 1**): P068

**Introduction**: Values of SVV higher than 13% in some cases predict fluid responsiveness. SVV is derived from pulse contour analysis of arterial blood pressure curve. The uncalibrated “Flotrac”-device (Edward Lifesciences) estimates SV (and so SVV) with an unpublished formula. In contrast to the PiCCO device (Pulsion), where SV (and so SVV) is derived from pulse contour analysis calibrated to individual patient. But also with fingerplethysmographically (“Clearsight”, Edward Lifesciences) derived blood pressure signals SVV can be obtained (using uncalibrated pulse contour analysis).

**Methods**: We compared 256 simultaneously derived measurements of SVV from Clearsight (SVV_CS), Flotrac (SVV_FT) and PiCCO (SVV_PiCCO) as gold-standard, directly after calibration of the PiCCO-device in 32 ICU patients. Primary endpoint: Concordance of the measurements within the categories “ < 9%”, “9–13%” and “ > 13%”. Statistics: Microsoft Excel, IBM SPSS 25.

**Results**: n = 32 (m = 22, f = 10; age 69 ± 13), APACHE II 29 ± 6, vasopressors 175/256. SVV_PiCCO could not be derived in 22/256 (8.8%) pairs of measurement, whereas Clearsight- and Flotrac-signals always could be obtained. SVV_PiCCO was higher (13 ± 7.6%) than SVV_FT (9 ± 4.9%) and SVV_CS (10 ± 5.4%). Classification within the categories “ < 9%”, “9–13%” and “ > 13%” for SVV_FT and SVV_PiCCO agreed in 118 of 234 (50.43%) measurements and for SVV_CS and SVV_PiCCO in 116 of 234 (49.57%) measurements. When anticipating fluid responsiveness at a cut-off of 13%, SVV_FT predicted fluid-responsiveness correctly in 38 of 113 cases (33.6%) and false positive in 3 of 113 cases (2.7%). SVV_CS predicted fluid-responsiveness correctly in 53 of 113 cases (46.9%) and false positive in 17 of 113 cases (15%).

**Conclusions**: SVV-PiCCO was higher than SVV_FT or SVV_CS. There was only about 50% chance that SVV_FT, respectively SVV_CS values fell in the same categories as SVV_PiCCO. For the relevant category of SVV_PiCCO > 13 there was even lower concordance, especially for SVV_FT.

## P069

### Use of PEEP to detect fluid responsiveness in patients suffering from acute respiratory distress syndrome

#### M. Benlabed^1^, S. Benlabed^2^, A. Ladjouze^3^, R. Gaudy^4^, S. Nedjari^3^

##### ^1^Anesthesiology, Lille University, Lille, France; ^2^Internal Medecine, Free University of Brussels, Brussels, Belgium; ^3^Anesthesiology and Intensive Care, Algiers University, Algiers, Algeria; ^4^Intensive Care, Lille University, Lille, France

*Critical Care* 2021, **25**(**Suppl 1**): P069

**Introduction**: The prediction of fluid responsiveness is required to avoid unnecessary volume expansion in patients with ARDS. So the objective of our study was to assess whether hemodynamic changes during a short elevation of PEEP would predict fluid responsiveness in patients with ARDS.

**Methods**: This prospective observational study conducted from april 2018 to april 2020 enrolled 30 patients presenting a mild ARDS according the classification of Berlin.They were 60 ± 10 years old and were submitted to a protective ventilation with PEEP 8, with the use of “tidal volume challenge” to improve the reliability of pulse pressure variation (PPV) [1]. A PEEP challenge was performed in patients hemodynamically stable, and consisted of increasing PEEP progressively from 8 cmH_2_O to 12 cmH_2_O with a time of ten minutes to reach 12 cmH_2_O. We recorded continuously, systolic pressure variation (SPV), mean arterial pressure (MAP) and PPV from the arterial wave line. We measured also cardiac index (CI) with echocardiography. Central venous pressure (CVP), PPV, SPV, MAP, and CI, were evaluated at PEEP 8 and at PEEP 12.

**Results**: Statistical analysis was performed using student’s t test. Results were expressed as mean ± std deviation. We observed finally that PEEP challenge from PEEP 8 (control) increases PPV, SPV, CVP and decreases MAP (Fig. 1). MAP (mmHg) decreases from 70.23 + -2.02 to 63.56 + -2.01 (p < 0.001). PPV (%) increases from 10.023 ± 1.24 to 17.65 ± 1.62 (p < 0.001). SPV (%) increases from 9.42 ± 1.25 to 15.72 ± 1.7 (p < 0.001). CVP (cmH_2_O) increases from 10.62 ± 1.14 to 14.23 ± 1.32 (p < 0.003) and finally CI (l/min/m^2^) decreases from 3.8 ± 0.3 to 2.5 ± 0.2, p < 0.003.

**Conclusions**: In patients with mild ARDS, PEEP challenge could detect fluid responsiveness. The absence of significant increase of PPV, during an elevation of PEEP might be used to identify the patients who are not responders.

**Reference**

1. De Backer D et al. Intensive Care Med 31:517–23, 2005.

**Figure:**
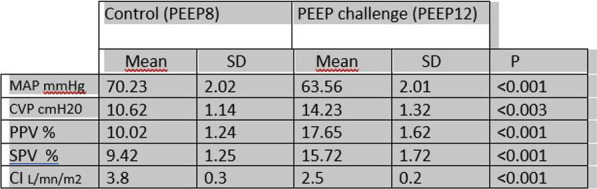
**Fig. 1** **(abstract P069)** Hemodynamic parameters at PEEP 8 and at PEEP 12

## P070

### Tidal volume challenge to predict preload responsiveness in patients with acute respiratory distress syndrome under prone position

#### R. Shi, S. Ayed, F. Moretto, N. De Vita, F. Gavelli, S. Carelli, A. Pavot, C. Lai, X. Monnet, J. L. Teboul

##### Service de Médecine Intensive-Réanimation, Hôpital de Bicêtre, Université Paris-Saclay, AP-HP, DMU CORREVE, Inserm S_999, FHU SEPSIS, Groupe de Recherche Clinique CARMAS, Le Kremlin-Bicêtre, France

*Critical Care* 2021, **25**(**Suppl 1**): P070

**Introduction**: Testing preload responsiveness in patients with acute respiratory distress syndrome (ARDS) during prone position without requiring cardiac index (CI) measurements has been poorly investigated [1, 2]. Our study aimed to evaluate the ability of pulse pressure variation (PPV) and its changes during a 1-min tidal volume (TV) challenge (TVC) to assess preload responsiveness in ARDS patients under prone position.

**Methods**: Patients with ARDS ventilated with a 6 ml/kg TV under prone position were prospectively included. By using a pulse contour analysis monitor, we measured PPV and changes in CI during a Trendelenburg maneuver (ΔCITREND). After transiently increasing TV to 8 ml/kg, we measured first absolute changes in PPV during TVC (ΔPPV TVC6-8) and then changes in CI during end-expiratory occlusion (EEO) (ΔCI EEO8). Preload responsiveness was defined by both ΔCITREND ≥ 8% [1] and ΔCI EEO8 ≥ 5% [2]. Preload unresponsiveness was defined by both ΔCITREND < 8% and ΔCI EEO8 < 5%.

**Results**: Eight-four cases were analyzed in 58 patients (65 ± 11 y.o.) under prone position for 11 (2–14) hours. The mean arterial pressure was 82 (75–90) mmHg (under norepinephrine in 83% cases). The driving pressure was 12 (10–18) cmH_2_O, the respiratory system compliance was 32 (21–40) ml/cmH_2_O and the ratio of partial pressure arterial oxygen and fraction of inspired oxygen was 104 ± 27 mmHg. Forty-two cases were classified as preload responders. The baseline PPV predicted preload responsiveness with an area under the receiver operating characteristic curve (AUROC) of 0.85 ± 0.04 (threshold 5%; sensitivity: 74%, specificity: 79%). The ΔPPV TVC6-8 predicted preload responsiveness with an AUROC of 0.94 ± 0.03 (threshold 2%; sensitivity: 98%, specificity: 88%) (p = 0.03 vs. PPV).

**Conclusions**: In patients with ARDS under protective ventilation during prone position, the changes in PPV during a 1-min TVC could reliably assess preload responsiveness without the need of CI measurements.

**References**

1. Yonis H et al. Crit Care 21:295, 2017.
2. Gavelli F et al. Ann Intensive Care 10:65, 2020

## P071

### Risk factors for mortality in ICU cardiac arrest

#### G. Jansen^1^, O. Sauzet^2^, R. Borgstedt^3^, S. Entz^4^, F. Holland^5^, S. Lamprinaki^4^, K. Thies^1^, S. Scholz^1^, S. Rehberg^1^

##### ^1^Department of Anaesthesiology, Intensive Care, Emergency Medicine, Transfusion Medicine, and Pain Therapy, Protestant Hospital of the Bethel Foundation, University Hospital OWL, University of Bielefeld, Bielefeld, Germany; ^2^Bielefeld School of Public Health & Center for Statistics, Bielefeld University, Bielefeld, Germany; ^3^Protestant Hospital of the Bethel Foundation, University Hospital OWL, University of Bielefeld, Bielefeld, Germany; ^4^Clinic for Internal Medicine and Gastroenterology, Protestant Hospital of the Bethel Foundation, University Hospital OWL, University of Bielefeld, Bielefeld, Germany; ^5^Clinic for Internal Medicine and Nephrology, Protestant Hospital of the Bethel Foundation, University Hospital OWL, University of Bielefeld, Bielefeld, Germany

*Critical Care* 2021, **25**(**Suppl 1**): P071

**Introduction**: This study evaluates the incidence, risk factors of mortality and 1-year-survival in patients with cardiac arrest in the ICU (ICUCA).

**Methods**: At a German university hospital with 78 non-cardiac-surgical ICU beds (surgical 41, medical 37), all adult patients with ICUCA, defined as need for performing chest compressions and/or defibrillation for the first time on the ICU were included. Primary endpoint was the occurrence of ICUCA. Secondary endpoints included conditions associated with ICUCA, rates of no-return-of-spontaneous-circulation (ROSC), risk factors of no-ROSC, survival to hospital discharge, 1-year survival and cerebral performance category (CPC) 1 year after cardiac arrest.

**Results**: We observed 114 ICUCA out of 14,264 ICU-admissions and 64,809 occupancy days according to an incidence of 0.8% of ICU-admissions (95%CI: 0.7–1.0) and 0.2% of ICU occupancy days (95%CI: 0.1–2). Conditions associated with ICUCA were cardiocirculatory (n = 88 [77%]), induction of anaesthesia (n = 26 [23%]), respiratory failure [n = 19 [17%]) and airway complications (n = 14 [12%]). Thirty-four patients (30%) had no-ROSC. Risk factors of no-ROSC were cardiac (odds ratio (OR): 5.4; 95%CI: 1.4–20.7)/hepatic comorbidities (OR: 7.2; 95%CI: 0.9–54.6), SOFA ≥ 2 (OR: 8.3; 95%CI: 0.9–74.8) and continuous-renal-replacement-therapy before ICUCA (OR: 5.9; 95%CI: 1.7–20.8). Interestingly, HCO_3_^−^levels > 21 mmol/l in combination with cardiac comorbidities were associated with a higher mortality-risk (HCO_3_^−^ < 21 mmol/l: 13%; 21–26 mmol/l: 45%; > 26 mmol/l: 42%) / SOFA ≥ 2 (HCO_3_^−^ < 21 mmol/l: 8%; 21–26 mmol/l: 36%; > 26 mmol/l: 33%). Hospital-mortality was 78% (n = 89). 1-year-survival-rate was 10% (95%CI: 5.5–17.7), survival with a good neurological (CPC1-2) outcome was 6.1% (95%CI: 2.5–12.2).

**Conclusions**: ICUCA is a rare but serious complication in the ICU. Further research should concentrate on identifying early predictors of survival, such as HCO_3_^−^ levels, and subsequently on the prevention of ICUCA.

## P072

### Identification of physiological subphenotypes in post-cardiac arrest patients

#### H. Kim, A. Afshar, R. Stevens

##### Department of Anesthesiology and Critical Care Medicine, Johns Hopkins University School of Medicine, Baltimore, USA

*Critical Care* 2021, **25**(**Suppl 1**): P072

**Introduction**: The management of postcardiac arrest (CA) patients may be ineffective or even harmful due to heterogeneity of treatment effects. Heterogeneity could be mitigated if undifferentiated CA populations could be resolved into biologically more uniform subphenotypes. We hypothesized that computational analysis of physiological time series (PTS) data recorded in the first 3 days of intensive care would uncover latent, clinically meaningful subphenotypes.

**Methods**: Patients admitted to ICU after CA were identified in the multi-center Philips eICU database, and PTS signals recorded in first 72 h after ICU admission were extracted (heart rate, blood pressure, pulse oximetry, and respiratory rate). Temporal, frequency, and information theory-based PTS features were derived and analyzed using consensus clustering, an unsupervised machine learning approach which reconciles clustering information about the same dataset coming from different sources or from different runs of the same algorithm.

**Results**: From 2,095 CA patients in eICU we derived 91 PTS features, from which 3 unique clusters were identified, each associated with a distinct clinical outcome distribution, leading to the designation of low, intermediate, and high risk subphenotypes (respectively clusters 1, 3 and 2 in Fig. 1). Clusters were differentiable by PTS features but not by demographic and/or other clinical characteristics (Fig. 1C).

**Conclusions**: Among CA patients admitted to ICU, unsupervised learning applied to data routinely recorded in the ICU indicates the presence distinct physiological subphenotypes associated with specific outcome probabilities. These subphenotypes were latent, as they could be differentiated on the basis of clinical features.

**Figure:**
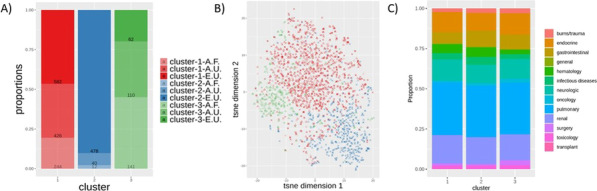
**Fig. 1** **(abstract P072)** A) Proportion plot of identified 3 clusters of CA patients. B) t-SNE dimension reduced representation. C) Proportion of diagnosis received within first 6 h of ICU admission stratified by subphenotypes. [A.F (alive favorable outcome), A.U (alive unfavorable outcome), E. U (Expired unfavorable outcome)]

## P073

### Hyperacute prediction of targeted temperature management outcome after cardiac arrest: a computational approach

#### J. Hsu

##### Biomedical Engineering and Computer Science, Johns Hopkins University, Baltimore, USA

*Critical Care* 2021, **25**(**Suppl 1**): P073

Introduction: Targeted temperature management (TTM) is associated with higher odds of neurological recovery in comatose survivors of cardiac arrest. However, the efficacy of TTM is not consistently observed, possibly due to heterogeneity of treatment effects. The aim of this study is to determine if models leveraging data available in the first 6 h after ICU admission (hyperacute phase) are predictive of short-term outcomes after TTM.

**Methods**: Adult patients receiving TTM after cardiac arrest were selected from a multicenter ICU database. Predictive features were extracted from clinical, physiologic, and laboratory data available in the hyperacute phase. Primary endpoints were survival and favorable neurological outcome, determined as a motor Glasgow Coma Scale (mGCS) of 6 upon discharge. Three machine learning algorithms were trained: GLM, random forest (RF), and gradient boosting (XGboost). Models with optimal features from forward selection were tenfold cross-validated and resampled 10 times.

**Results**: Data were available on 969 cardiac arrest patients who received TTM, of whom 491 survived and 237 had favorable neurological discharge. The GLM performed best, with an AUROC of 0.702 ± 0.029, sensitivity 0.621 ± 0.116, and specificity 0.666 ± 0.117 for the prediction of survival and an AUROC of 0.678 ± 0.041, sensitivity 0.691 ± 0.149, and specificity 0.565 ± 0.137 for the prediction of favorable neurological function. Highly ranked features predictive of survival and favorable neurological outcome included male gender, higher respiratory rate, and greater systolic blood pressure range.

**Conclusions**: In patients receiving TTM after cardiac arrest, short-term outcomes can be predicted with data routinely collected in the first 6 h after ICU admission. Ongoing model iterations will integrate novel features and include external validation. Hyperacute prediction could increase the effectiveness of clinical decision-making in the post-cardiac arrest setting.

## P074

### Measuring non-technical skills during prehospital advanced cardiac life support: a pilot study

#### M. Vanneste, P. Dewolf, D. Desruelles, L. Wauters

##### Department of Emergency Medicine, University Hospitals Leuven, Leuven, Belgium

*Critical Care* 2021, **25**(**Suppl 1**): P074

**Introduction**: Emergency mobile medical teams (MMT) are faced with out-of-hospital cardiac arrests (OHCA) daily. Despite their efforts, patient survival is limited. Research has demonstrated that optimal CPR efforts not only require technical skills and knowledge, but also non-technical skills (NTS) such as leadership, teamwork, and task management [1]. This prospective observational study analyses the NTS of MMTs during OHCA, using the validated Team Emergency Assessment Measure (TEAM). Also, the correlation between NTS and outcome is researched.

**Methods**: Adult patients who suffered from an OHCA between July 2016, and June 2018, and treated by MMT from the University Hospital Leuven, were eligible for the study. Resuscitations were video recorded by the team leader using a GoPro body camera. The recordings were independently scored by 2 ACLS-certified emergency physicians.

**Results**: A total 114 OHCAs were analyzed. The mean TEAM score was 34.4/44. The mean item score was 3.1/4; ‘effective team communication’ scored the lowest (2.4), while ‘acting with composure/control’ and ‘following of approved standards/guidelines’ scored the highest (3.4). The average theme scores were 2.9 for ‘Leadership’, 3.1 for ‘Teamwork’ and 3.3 for ‘Task management’. ‘Leadership’ was rated significantly lower than ‘Teamwork’ (p = 0.004) and ‘Task management’ (p < 0.001). No significant correlation was found between TEAM and ROSC (p = 0.574) or one month survival (p = 0.225).

**Conclusions**: Although the TEAM score was good, large differences were observed. Task management was scored high, while leadership and team communication received lower scores. Despite the growing evidence, in this study no correlation was found between NTS and survival. Introduction of a NTS program into ACLS courses, with a focus on leadership and communication, might improve NTS. A constructive feedback platform can encourage post-event ‘hot debriefs’ to identify points of improvement. TEAM can be a helpful tool to achieve this.

**Reference**

1. Dewolf P et al. AEM Educ Train 5:e10522, 2020.

## P075

### Dispatcher-assisted conventional cardiopulmonary resuscitation and outcomes for pediatric out-of-hospital cardiac arrests

#### Y. Goto^1^, T. Maeda^1^, Y. Goto^2^, A. Funada^3^

##### ^1^Department of Emergency and Critical Care Medicine, Kanazawa University Hospital, Kanazawa, Japan; ^2^Department of Cardiology, Yawata Medical Center, Komatsu, Japan; ^3^Department of Cardiology, Saiseikai Senri Hospital, Suita, Japan

*Critical Care* 2021, **25**(**Suppl 1**): P075

**Introduction**: In pediatric out-of-hospital cardiac arrest (OHCA), as asphyxia cardiac arrest is more common than cardiac arrest from a primary cardiac event, effective ventilation is crucial during CPR. We hypothesized that dispatcher-assisted conventional CPR would be better than dispatcher-assisted compression-only CPR as a bystander CPR instruction.

**Methods**: We analyzed the records of 8172 children who received bystander dispatcher-assisted CPR after OHCA using the All-Japan Utstein-style registry for 13 years (2005–2017). Patients were divided into conventional CPR (n = 3077) and compression-only CPR (n = 5095) groups. The primary study endpoint was 1-month neurological intact survival, defined as a Cerebral Performance Categories score of 1–2 (CPC 1–2). Secondary study endpoints were 1-month survival and pre-hospital return of spontaneous circulation (ROSC).

**Results**: The CPC 1–2 rate was significantly higher in the conventional CPR group than in the compression-only CPR group (before propensity score [PS] matching, 5.7% [175/3077] vs. 3.1% [160/5095], p < 0.0001; after PS matching, 6.0% [156/2618] vs. 2.6% [69/2618], p < 0.0001). Multivariable logistic regression analysis revealed that compared with compression-only CPR, conventional CPR was associated with increased odds of CPC 1–2 (before PS matching, adjusted OR 2.48, 95% CI 1.91–3.22, p < 0.0001; after PS matching, adjusted OR 2.42, 95% CI 1.76–3.32, p < 0.0001). There were significant differences in the survival and ROSC analysis between the conventional CPR and compression-only CPR groups (before PS matching, 13.4% [412/3077] vs. 10.3% [523/5095] for survival, p < 0.0001, 8.0% [246/3077] vs. 6.2% [315/5095] for ROSC, p < 0.01; after PS matching, 13.9% [365/2618] vs. 9.4% [245/2618] for survival, p < 0.0001, 8.5% [223/2618] vs. 5.2% [135/2618] for ROSC, p < 0.0001).

**Conclusions**: Dispatcher-assisted conventional CPR may be preferable to dispatcher-assisted compression-only CPR as an optimal CPR instruction for coaching callers to perform bystander CPR.

## P076

### Comparing the efficiency of ventilation between CPR protocols during a prolonged cardiac arrest

#### J. Kopra^1^, M. Skrifvars^2^, E. Litonius^2^, P. Pekkarinen^2^, J. A. Heinonen^2^, L. Fontanelli^2^, T. Mäkiaho^2^

##### ^1^Department of Diagnostics and Therapeutics, University of Helsinki, Helsinki, Finland; ^2^University of Helsinki, Helsinki, Finland

*Critical Care* 2021, **25**(**Suppl 1**): P076

**Introduction**: In case of prolonged out-of-hospital cardiac arrest (OHCA), the patient is commonly transported to hospital using mechanical CPR and ventilation with 100% oxygen. Despite this many patients arriving to hospital have severe hypercapnia and hypoxia. One possible reason for this is impaired ventilation due to the counterpressure caused by the continuous chest compressions. We hypothesized that a compression/ventilation ratio of 30:2 would provide better ventilation and gas exchange compared to continuous compressions over ventilation during prolonged CPR.

**Methods**: We randomized 30 anesthetized domestic swine (weight approximately 50 kg) with electrically induced ventricular fibrillation to continuous or intermittent chest compressions and bag-valve-mask ventilation with 100% FiO_2_. We started CPR after a 5-min no-flow period and continued it up to 40 min from the induction of VF. Chest compressions were performed with a mechanical chest compression device (LUCAS®, Stryker Medical). We collected arterial blood gas samples every 5 min during CPR. We compared PaO_2_, PaCO_2_ and lactate over time using a mixed linear model.

**Results**: There were no statistically significant differences in PaO_2_ (p = 0.40), PaCO_2_ (p = 0.79) or lactate (p = 0.37) between continuous and 30:2 compression/ventilation (Fig. 1). The significance values for the interaction between compression/ventilation group and time were as follows: p = 0.43 for PaO_2_; p = 0.37 for PaCO_2_; p = 0.55 for lactate.

**Conclusions**: Prolonged CPR with mechanical chest compressions performed either continuously or with a 30:2 compression/ventilation ratio resulted in similar arterial levels of oxygen, carbon dioxide and lactate.

**Figure:**
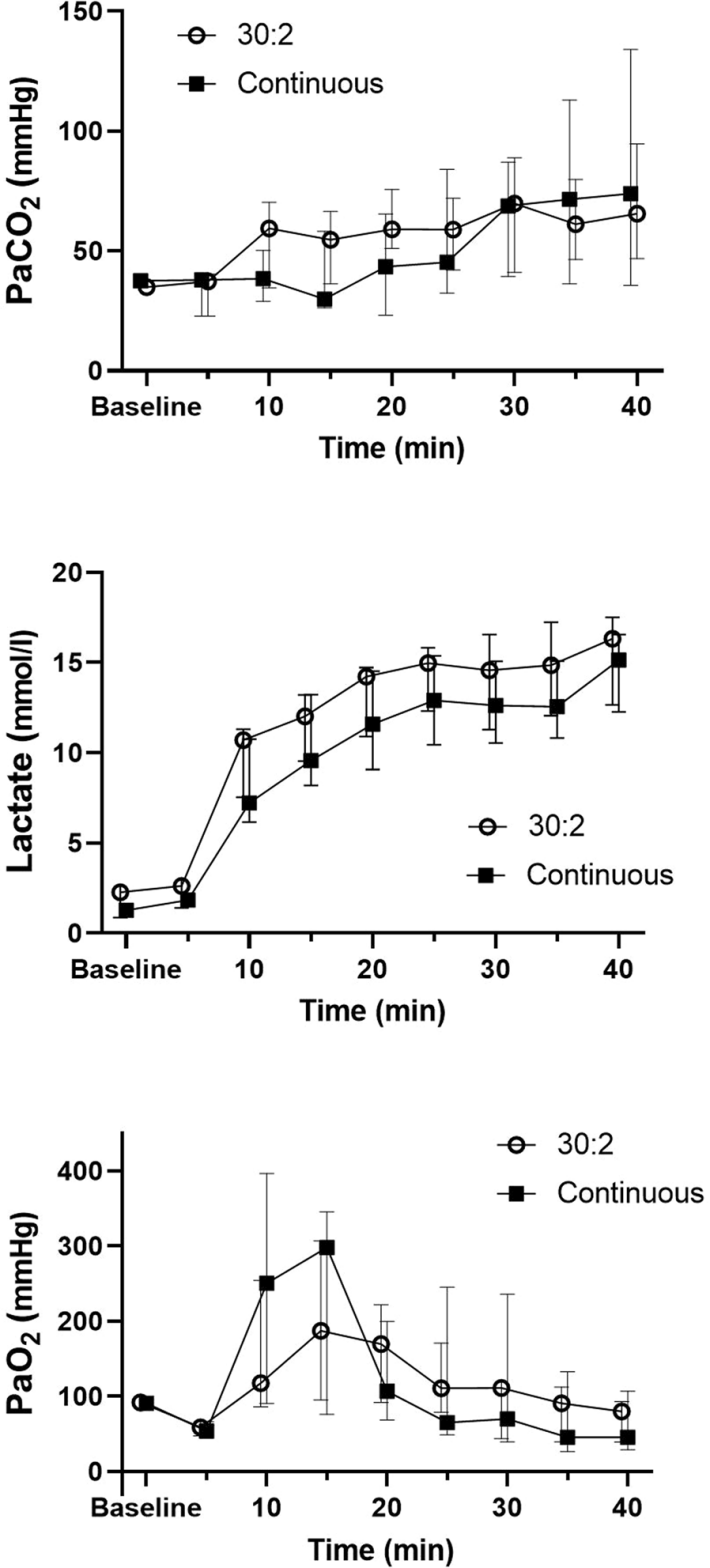
**Fig. 1** **(abstract P076)** PaCO_2_, lactate and PaO_2_ median values and IQRs in the 30:2 compression/ventilation and continuous compression/ventilation groups at baseline measurements and at 5 min intervals after the cardiac arrest. The CPR was started at 5 min time point

## P077

### Long-term survival and neurological outcome of adult patients with cardiac arrest treated with extracorporeal versus conventional CPR: a systematic review and meta-analysis

#### L. Bambini^1^, L. Mascia^2^, E. Maietti^2^, M. Bordini^1^, I. Cavalli^1^, G. Pizzilli^3^, P. Rucci^2^, V. M. Ranieri^1^, T. Tonetti^1^

##### ^1^Department of Medical and Surgical Sciences, Alma Mater Studiorum University of Bologna, Bologna, Italy; ^2^Department of Biomedical and Neuromotor Sciences, Alma Mater Studiorum University of Bologna, Bologna, Italy; ^3^Department of Anesthesia and General Intensive Care Unit, Sant’Orsola-Malpighi Hospital, Bologna, Italy

*Critical Care* 2021, **25**(**Suppl 1**): P077

**Introduction**: Long-term survival and good neurological outcome after cardiac arrest remain low [1]. International resuscitation guidelines suggest that extracorporeal cardiopulmonary resuscitation (ECPR) may play a role in improving survival and rate of favorable neurological outcome (identified as Cerebral Performance Categories or CPC score = 1–2) [2], but there are still uncertainties about the precise benefit size. We designed a systematic review and meta-analysis aiming to evaluate whether long-term survival and long-term neurological outcome are better in adult patients with cardiac arrest treated with ECPR compared to those treated with conventional CPR (CCPR).

**Methods**: We performed a literature search on Pubmed of all ECPR trials published between 2005 and 2019. Only trials with a control group treated with CCPR were considered eligible. Eighteen out of 1238 examined studies were included. All data about survival and neurological outcome at 1 months, 6 months, 1 year and 2 years from cardiac arrest were extracted from each study. We performed separate meta-analyses for studies with matched and unmatched data for each outcome and time point.

**Results**: ECPR was shown to improve survival at 1 month (RR = 0.83, CI 95%: 0.73 – 0.94), at 6 months (RR = 0.81, CI 95%: 0.70 – 0.93) and at 1 year from cardiac arrest (RR = 0.87, CI 95%: 0.78 – 0.97). We also found an association between ECPR implementation and favorable neurological outcome (CPC score = 1–2) at 1 month (RR = 0.89, CI 95%: 0.85 – 0.93) and 6 months (RR = 0.89, CI 95%: 0.81 – 0.97) from cardiac arrest (Fig. 1).

**Conclusions**: ECPR is an advanced treatment that appears to be associated with increased long-term survival and long-term favorable neurological outcome (identified as CPC score = 1–2). To our knowledge, this systematic review and meta-analysis is the first to analyze long-term survival and neurological outcome after ECPR.

**References**

1. Benjamin EJ et al. Circulation 137: E67–E492, 2018
2. Kratzert WB et al. J Cardiothorac Vasc Anesth 34:1195–1197, 2020

**Figure:**
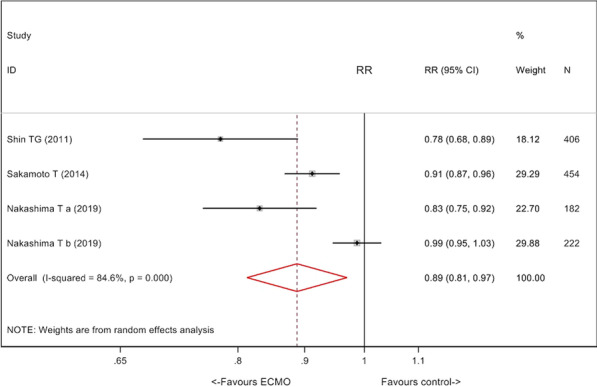
**Fig. 1** **(abstract P077)** CPC score at 6 months

## P078

### Early recognition of clinical deterioration - the key in preventing in-hospital cardiac arrest and impact on mortality

#### P. A. Ramos^1^, A. M. Oliveira^1^, D. C. Gonçalo^2^, Â. Simas^1^, R. Duarte^3^, J. Gonçalves Pereira^1^

##### ^1^Intensive Care Unit, Hospital Vila Franca de Xira, Lisboa, Portugal; ^2^Internal Medicine Department, Centro Hospitalar do Oeste - Unidade Caldas da Rainha, Caldas da Rainha, Portugal; ^3^Cardiology Department, Hospital Vila Franca de Xira, Lisboa, Portugal

*Critical Care* 2021, **25**(**Suppl 1**): P078

**Introduction**: The annual incidence of in-hospital cardiac arrest (ihCA) in Europe ranges between 1.5–2.8/1000 admissions and is associated with high mortality. Up to 80% of ihCA are preceded by easily identifiable signs of clinical deterioration. A Rapid Response Team (RRT) may help to minimize in-hospital morbidity and mortality. However, the RRT activation is delayed or even not activated in 30–78% of patients who might benefit. A Modified Early Warning Score (MEWS) may allow the early detection of these patients with a high mortality risk, prevent ihCA and allow prompt admission to the ICU.

**Methods**: Retrospective, observational, single center study. Data were collected from the RRT activation registries and hospital clinical database. We included all adult RRT activations between January 2018 and December 2020. Clinical signs during the 24 h before the index activation were collected for patients with ihCA.

**Results**: During the study period there were 144 RRT adult activations, including 54 ihCA. Of these, 9 were excluded due to incomplete data. The 45 ihCA remaining patients were old (mean age 75 ± 15 years) and 28 (62%) were male. Patients with a RRT activation for another cause were younger (63 ± 22 years), and 37 (41%) were male, p < 0.001. The MEWS score was elevated 24 h before ihCA patients in 67% of patients. In 14 patients (31%), there were criteria for the activation of the RRT (MEWS ≥ 4). The most common initial rhythm was assistole (91%); the median time of the cardiopulmonary resuscitation attempt was 18 min [10–25] , but only 7% of patients had return of spontaneous circulation. The ihCA hospital mortality was 93%. On opposite, the other RRT activations, without ihCA, had a mortality rate of only 8% (Table 1).

**Conclusions**: During the study, period ihCA had a very high mortality rate. Early recognition of signs of clinical deterioration (using the MEWS score) and prompt activation of a RRT may help to prevent ihCA and decrease mortality.

**Table:** **Table 1** **(abstract P078)** Comparison between patients of ihCA and non ihCA activation

	**ihCA activations**	**Non ihCA activations**
Male (%)	62	41
Age (mean; standard deviation)	75 ± 15	63 ± 22
Hospital mortality (%)	93	8

## P079

### Contribution of simulation learning management of cardiocirculatory arrest

#### W. Sellami^1^, K. Raddaoui^2^, T. Hannachi^3^, I. Ben Mrad^1^, I. Labbene^1^, M. Ferjani^1^

##### ^1^Department of Anesthesiology and Intensive Care Unit, Military Hospital of Tunis, Tunis, Tunisia; ^2^Department of Anesthsiology and Intensive Care Unit, Institut Mohamed Kassab, Manouba, Tunisia; ^3^Department of Anesthsiology and Intensive Care Unit, Bougatfa Hopsital, Bizerte, Tunisia

*Critical Care* 2021, **25**(**Suppl 1**): P079

**Introduction**: Medical simulation is both a means of assessment and training. The aim of this study is to study the medium-term educational contribution of high-fidelity simulator compared to conventional training.

**Methods**: A population of anesthesist-intensive care and emergency medicine residents; already familiar with simulation for clinical situations other than cardiocirculatory arrest at the IMS simulation center; were included in the study and divided into two groups. A group “ACC” for Cardio-Circulatory Arrest, which was specifically trained on a simulator on this topic. A control group “C” who had only theoretical training. Each of the residents was assessed on their management of critical situation: refractory ventricular fibrillation (VF) at six weeks and then at 6 months from the date of initial practical training for the ACC group. The scenario was the same for all residents.

**Results**: Twenty residents in intensive care anesthesia and 10 in emergency medicine were included with 15 residents per group (10 anesthesia-intensive care and 5 emergency medicine). The scores obtained by ACC group were significantly higher than those of the C group either at 6 weeks or at 6 months, and also for the “diagnosis” and “compliance with the algorithm” subgroups. There is no significant improvement in scores between the different assessment times for the ACC group. On the other hand, for group C there is a significant improvement in grades at 6 months (T1) compared to grades obtained at 6 weeks. The ratings of participants during the study were positive.

**Conclusions**: This study confirms the short and medium term educational benefit of simulator training versus traditional training. The use of simulation allows knowledge retention for up to 1 year after the initial practical training phase.

## P080

### AiCR 1, artificial intelligence in cardiac arrest 1: automatized extraction tool by text mining in cardiac arrest

#### R. Lombardi^1^, T. Le Blévenec^2^, A. Mazeraud^3^, D. Duhautbout^4^, G. Bernardin^1^, P. Deswardt^5^, P. M. Bertrand^6^, J. Dellamonica^1^, T. Sharshar^3^

##### ^1^Médecine Intensive-Réanimation, CHU L´Archet-Nice, Nice, France; ^2^Talan solutions, Paris, France; ^3^Neurointensive Care and Neuroanesthesia Department, GHU Paris Psychiatrie et Neuroscience, Paris, France; ^4^AiiNTENSE, Evry, France; ^5^Service de Réanimation Polyvalente, Centre Hospitalisation d’Antibes-Juan les Pins, Antibes, France; ^6^Service de Médecine-Intensive Réanimation, Centre hospitalier de Cannes, Cannes, France

*Critical Care* 2021, **25**(**Suppl 1**): P080

**Introduction**: Cardiac arrest is a public health key, with 10.985 patient/year, with an extremely high death rate (overall survival rate at hospital discharge is 10%) [1, 2]. In the literature, there are many examples of the artificial intelligence (AI) efficiency, specially, in intensive care unit (ICU), in the prediction, in database creation, in the therapeutic help [3–5]. In some case, it could be fastidious to create a database, so we developed an automatized extraction tool, based on a text mining approach.

**Methods**: We used unstructured data, including computerized hospital report (CHR), from two centers. Successive samples of CHR have been drawn randomly and annotated, to reach a total of 143 annotated CHR. We incrementally increased the size of samples. If the concordance rate exceeded or if it was equal to 90%, for every interest variable, we considered the sample validated. Once the data are extracted, we performed a clustering, to identify patterns in the CHR, a missing value and a miss extraction value analysis.

**Results**: After the full development and the training of our tool, we can extract 51 variables, with a mean accuracy of 92.75%, with the better performance in admission biology, patient characteristics, and the deceased status (Fig. 1). An analysis on algorithms errors showed that the main extractions errors were attributable to the complexity context and to the complexity variables (36% both). The most missing values were found in the biology variables (59.27%). The clustering results showed two group of CHR, differing for death, witness presence, multi-organ failure, status epilepticus and Glasgow scale.

**Conclusions**: We developed a reliable extraction tool, fully automated and adaptative, able to extract hundreds of data in seconds (0.27 s for one CHR and 137.6 s for 504 CHR). It is useful for data base creation, by is simplicity and its quick use.

**References**

1. Berdowski J et al. Resuscitation 81:1479–87, 2010
2. Gräsner JT et al. Resuscitation 161:61–79, 2021
3. Komorowski M et al. Nat Med 24:1716–20, 2018
4. Alex B et al. J Biomed Semant 10:23, 2019
5. Meng F et al. J Am Med Inform Assoc 22:980–6, 2015

**Figure:**
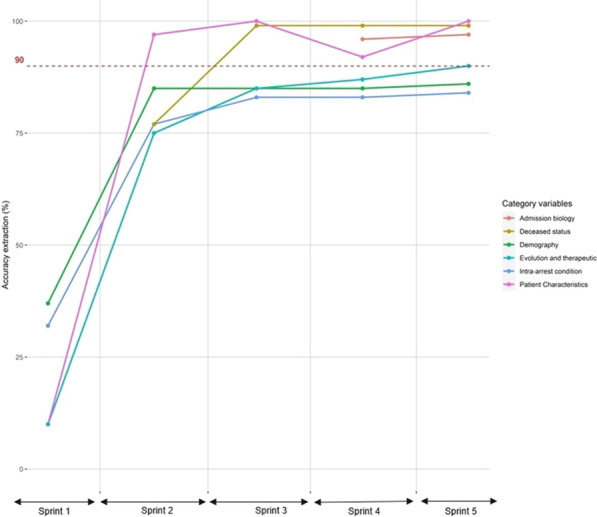
**Fig. 1** **(abstract P080)** Accuracy extraction, by category, in the training sample (143 CHR), Sprint corresponding to 2 months

## P081

### Persistent pre-eclampsia: survey of Moroccan women

#### R. Benmalek^1^, A. Asklou^2^, S. Harouna^2^, A. El Jazouli^2^, S. Ejjebli^2^, M. Bensouda^3^, R. Habbal^2^

##### ^1^Cardiology, University Hospital Ibn Rochd, Casablanca, Morocco; ^2^University Hospital Ibn Rochd, Casablanca, Morocco; ^3^Gynecology and Obstetrics Department, Ibn Rochd Hospital, Casablanca, Morocco

*Critical Care* 2021, **25**(**Suppl 1**): P081

**Introduction**: Preeclampsia (PE) is the combination of pregnancy-induced hypertension (≥ 140/90 mmHg) and proteinuria greater than or equal to 300 mg per 24 h above 20 weeks of amenorrhea. It usually disappears immediately after delivery in 24 to 48 h. However, it can persist after placental evacuation and up to 6 weeks after delivery. Hence the importance of early diagnosis of persistent preeclampsia (PPE) postpartum and detect signs of severity, in order to establish adequate driving in time.

**Methods**: We led a retrospective study of 547 cases of preeclampsia collected in the Gynecology and Obstetrics department of the Ibn Rochd Hospital over a period of 3 years. Two groups were identified. Group 1 (n = 504) included patients with preeclampsia immediately disappearing in the postpartum and a group 2 (n = 43) of patients with PPE. We followed them before and after delivery.

**Results**: Persistent preeclampsia represented 8%. Mean age of our patients was 29.15 ± 15.13 years. Factors related to the persistence of preeclampsia were mainly pauciparity in 61%, history of PE in 4.7%, gestational age < 36 weeks in 56%, severe preeclampsia in 14.7%, hypotrophy in 6% and eclampsia in 2%, and massive 24 h proteinuria ≥ 3 mg/24 h in 60% (all p were 160/110 mmHg in 20.4%, a SBP > 170 mmHg in 13%, a DBP > 110 mmHg in 9%. Edema was present in 70% of cases and was generalized in 9% of cases. The vaginal delivery was done in 44% of cases, Caesarean section was recommended in 56% of cases. Conservative treatment was adopted in 22% of cases; 15.05% required immediate use of intravenous antihypertensive treatments.

**Conclusions**: Preeclampsia is still common in developing countries. It remains a major cause of maternal and fetal morbidity and mortality. Our study confirms the seriousness of persistent pre-eclampsia, which carries a high risk of maternal complications (eclampsia, acute renal failure, cytolysis, etc.) and maternal mortality, which can be reduced at the cost of a better detection and treatment policy.

## P082

Withdrawn

## P083

### MIS-C or missed diagnosis?

#### M. Wiggelinkhuizen, G. J. Jaspers

##### Pediatric Intensive Care, RadboudUMC, Nijmegen, Netherlands

*Critical Care* 2021, **25**(**Suppl 1**): P083

**Introduction**: The objective of this case description is to create awareness among pediatric intensivists about the diagnostic pitfalls regarding Multisystem Inflammatory Syndrome in Children (MIS-C) and its differential diagnosis.

**Methods**: By means of two case descriptions, we illustrate and reflect on the pitfalls that led to both under- and overdiagnosis of MIS-C.

**Results**: Brief patient descriptions are presented in Table 1. The 3-year-old patient was misdiagnosed for MIS-C and turned out to have a perforated appendicitis with peritonitis. The suspicion of MIS-C was based on clinical symptoms in combination with the elevated NT-pro-BNP, which is often seen in MIS-C. However, elevated cardiac biomarkers as NT-pro-BNP and Troponin-T may also be caused by non-cardiac causes, like sepsis, shock and ARDS [1]. The combination of fever, gastrointestinal symptoms and hypotension that were present in this girl, are often presenting symptoms of patients with MIS-C. However, they are not specific and are seen in a range of other diagnoses as well. The 8-year-old girl, with the same symptoms of fever, gastrointestinal symptoms and hypotension, was initially diagnosed for acute appendicitis, but turned out to have MIS-C with left ventricular failure. While no clear symptom or biomarker can distinguish between MIS-C and its differential diagnosis, the presence of conjunctivitis, skin rash and/or coronary dilatation on echocardiography are suggestive for MIS-C, while blood cultures can help to differentiate MIS-C from septic shock / toxic shock syndrome.

**Conclusions**: Intensivists have to be aware of the existence of MIS-C and the pitfalls of its broad clinical and biochemical abnormalities. Accurate physical examination, including examination for conjunctivitis and rash, echocardiography and blood culture results can be supportive to make the correct diagnosis and to start the right treatment, right on time!

Consent to publish was obtained from parents of both patients.

**Reference**

1. Yoldas T et al. Pediatric Cardiology 40:1638–1644, 2019

**Table:** **Table 1** **(abstract P083)** Brief case description of two patients

Age (years)	Presenting clinical features	Results	Primary diagnosis and therapy	Final diagnosis
3	abdominal pain, fever, hypotension, vomiting, diarrhea, no conjunctivitis or skin rash	CRP 270 mg/l, troponin-T < 3 ng/l, NT-proBNP 1000 pg/ml, PCR nCoV neg, nCoV pos contact unknown, blood culture neg, abd. ultrasound tubid ascites	MIS-C or acute abdomen Diagnostic laparoscopy	Perforated appendix with generalized peritonitis
8	abdominal pain, fever, hypotension, vomiting, headache, skin rash, no conjunctivitis	CRP 140 mg/l, troponin-T 124 ng/l, NT-proBNP 13,000 pg/ml, PCR nCoV neg, nCoV pos contact yes, blood culture neg, echo left ventricular failure	Acute appendicitis Laparoscopy: appendix sana	MIS-C

## P084

### Etiology of acute intoxications in children

#### L. Petcu^1^, B. Golovin^2^, S. Lupu^2^, M. Pestereanu^2^, N. Catanoi^2^, N. Doni^2^, A. Rabovila^2^

##### ^1^State Medical and Pharmaceutical, Emergency Doctor, National Centre of Pre-hospital Emergency Medicine, Chisinau, Moldova; ^2^State Medical and Pharmaceutical, National Centre of Pre-hospital Emergency Medicine, Chisinau, Moldova

*Critical Care* 2021, **25**(**Suppl 1**): P084

**Introduction**: Acute intoxications in children are an important problem in pediatric practice due to their frequency and severity [1]. To detect as early as possible the type of intoxication in the child, the time interval since the intoxication occurred and to act promptly and correctly in providing first aid and treatment.

**Methods**: The study included 92 medical records from the Emergency Department for the period 01.01.2021–02.06.2021.

**Results**: As a result of the study, it was found that out of the total number of patients with acute intoxications in 82 children (89%), the Emergency care team was requested, resulting in being transported to the Emergency Department and 10 children (1.08%) were referred independently. 46 children (50%) male, 46 children (50%) female. It was estimated that after age, the peak incidence includes children aged 1–5 years - 53 children (57.6%), intoxications being accidentally produced. Children aged 13–17 years -28 children (30.4%) poisonings being caused by suicide. Children aged 6–12 years - 11 children (11.9%). According to the etiology of intoxication, it was determined that it detoxifies intoxications with drugs-50 children (52.6%), chemicals - 19 children (20.6%), CO-10 children (10.8%), hydrocarbons -3 children (3, 2%), alcohol -3 children (3.2%), organophosphorus-2 children (2.1%), pesticides - 1 child (1.08%), nitrates -1 child (1.08%). Out of the total number of children, 75 children (81.5%) were hospitalized in the toxicology department, 16 children (17.3%) received treatment and dynamic monitoring in the Emergency Department, after which they were discharged at home.

**Conclusions**: The most important element of a favorable prognosis in acute intoxications in children consists in the early detection of acute intoxication, providing first aid at the prehospital stage, transport in the shortest time to the Emergency Department and providing treatment according to the protocol.

**Reference**

1. Ciofu E. Essentials in Pediatrics IV edition, page 566.

## P085

### Implementation of machine learning algorithm in the emergency department for the prediction of hospital admission

#### A. Sakagianni^1^, G. Feretzakis^2^, G. Karlis^3^, E. Loupelis^4^, D. Kalles^5^, L. Tzelves^6^, R. Chatzikyriakou^7^, N. Trakas^8^, V. Kaldis^9^

##### ^1^Intensive Care Unit, Sismanoglio General Hospital, Athens, Greece; ^2^Department of Quality Control, Research and Continuing Education, Sismanoglio General Hospital, Athens, Greece; ^3^Intensive Care Unit, Thoracic Diseases General Hospital Sotiria, Athens, Greece; ^4^IT Department, Sismanoglio General Hospital, Athens, Greece; ^5^School of Science and Technology, Hellenic Open University, Patras, Greece; ^6^Second Department of Urology, Sismanoglio General Hospital, National and Kapodistrian University of Athens, Athens, Greece; ^7^Hematology Laboratory, Sismanoglio General Hospital, Athens, Greece; ^8^Biochemistry Department, Sismanoglio General Hospital, Athens, Greece; ^9^Emergency Department,Sismanoglio General Hospital, Athens, Greece

*Critical Care* 2021, **25**(**Suppl 1**): P085

**Introduction**: In the Emergency Department (ED) rapid triage and timely interventions are critical for high-risk patients. One of the main ED priorities is to quickly identify those who will need hospital admission. This study evaluates the performance of a simple logistic model on its ability to predict whether a patient visiting the ED will subsequently be admitted to the hospital or not, based on initial ED patients’ data retrieved from the Biochemistry and Hematology Laboratories. Our aim is to find an algorithm using ML techniques to assist clinical decision-making in the emergency setting.

**Methods**: A total of 3,204 ED visits were analyzed during the study period (14 March-4 May 2019). The anonymous data set under investigation contained the following variables: serum levels of urea, creatinine, lactate dehydrogenase, creatine kinase, C-reactive protein, complete blood count with differential, hemoglobin, platelet count, activated partial thromboplastin time, D-Dimer, International Normalized Ratio, age, gender, triage disposition to ED unit, ambulance utilization and admission to hospital. All raw data was retrieved from a standard Hospital Information System (HIS) and a Laboratory Information System (LIS). The analysis was performed using the Waikato Environment for Knowledge Analysis (WEKA) software. In our analysis we evaluated the simple logistic classifier for building linear logistic regression model [1] using the LogitBoost algorithm.

**Results**: The simple logistic classifier weighted average results achieved an F-measure of 0.696, a precision value of 0.703, a recall value of 0.712 and an area under receiver operating characteristic curve (AUC ROC) of 0.755.

**Conclusions**: We present an inexpensive clinical decision support tool derived from readily available ED patient data. This tool intends to aid the emergency physician regarding hospital admission decisions, as the development of machine learning models represents a rapidly evolving field in healthcare.

**Reference**

1. Landwehr N et al. Machine Learning 59:161–205, 2005

## P086

### First responder Advanced technologies for Safe and efficienT Emergency Response: FASTER project

#### A. M. Cintora^1^, S. Gomez^2^, J. Ruiz^3^, O. Carrillo^3^, F. J. Carrillo^3^, M. R. Rodríguez^3^, A. K. Coll^4^, M. Á. Semprún^3^, C. Méndez^5^

##### ^1^Research Department, Emergencies Health Service of Madrid Community, Madrid, Spain; ^2^Emergency Health Service SUMMA112, Emergencies Health Service of Madrid Community, Madrid, Spain; ^3^Emergencies Health Service of Madrid Community, Madrid, Spain; ^4^Firefighter of Madrid Community, Madrid, Spain; ^5^Catastrophe Department, Emergencies Health Service of Madrid Community, Madrid, Spain

*Critical Care* 2021, **25**(**Suppl 1**): P086

**Introduction**: FASTER [1] is a H2020 research project [2] aimed at optimizing the response capacity of first responders in disaster situations with the objective of increasing the resilience of European emergency professionals to catastrophes.

**Methods**: It has been developed new technologies for International Urban Search and Rescue (USAR) [3] operational support. Innovative drones for mapping and logistical operations, autonomous vehicles with 3D and thermal camera, portable control center and resilient communications structure have been introduced in USAR procedures for increase the efficiency in the disaster response. It has been designed a scenario involving a simulated earthquake, with an area of collapsed buildings. FASTER tools have been testing in it, to confirm their usability in realistic, and hazardous environment (Fig. 1). User feedback and KPI has been collected regarding the functionality and effectiveness of the FASTER tools.

**Results**: The initial assessment of the disaster area has been improved with the results obtained within the UAVs aerial imagery. Risky maneuvers have also been decreased by performing the 3D inspection, thermal vision and agile communications.

**Conclusions**: The tools tested have given a major step forward in improving the work dynamics of the health emergency and firefighter professionals [4]. The planning process and the management of the disaster have been facilitated and enhanced using innovative technologies. Professional methods of Assessment, Search and Rescue (ASR) of INSARAG (NATO) [3] have been increased its efficiency.

**References**

1. https://cordis.europa.eu/project/id/833507/es Accessed 6–6-21
2. https://www.faster-project.eu/ Accessed 6–6-21
3. https://www.insarag.org/ Accessed 6–6-21
4. https://tinyurl.com/aw4ybbyf Accessed 6–6-21

**Figure:**
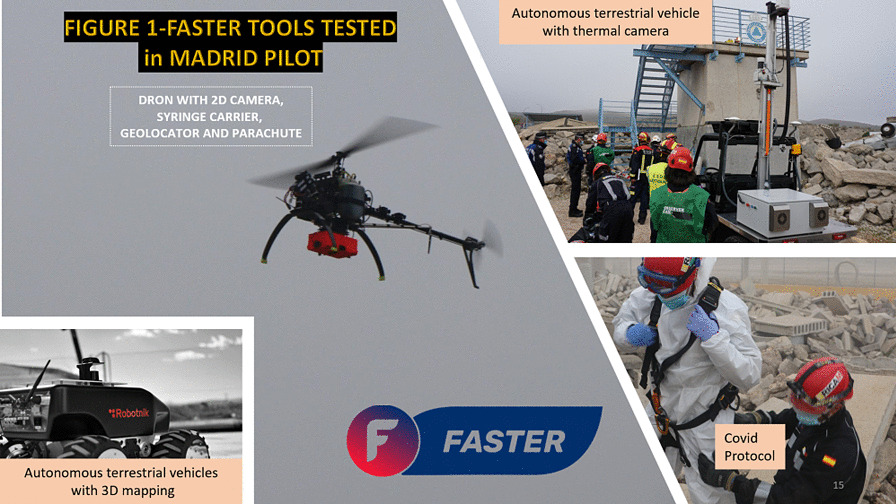
**Fig. 1** **(abstract P086)** FASTER Tools tested in Madrid Pilot with INSARAG procedures

## P087

### Communication knowledge of bad news in medical students

#### L. S. Costa^1^, F. Miranda Pacheco^1^, R. De Oliveira Ceciliano^1^, C. Mendes Lopes^2^, G. Olguin De Matos Costa Silva^1^, N. Folhadella Soares^1^, R. Xavier Millen Penedo^1^, V. Tokunaga Ferreira^1^

##### ^1^Medical Emergencies, Estacio de Sá University, Rio de Janeiro, Brazil; ^2^Drama School,Estacio de Sá University, Rio de Janeiro, Brazil

*Critical Care* 2021, **25**(**Suppl 1**): P087

**Introduction**: Several studies have shown a lack of effective communication skills amongst medical students, particularly concerning how to deliver bad news. Currently, discussions with undergraduated students about communication, including ‘SPIKES' protocol are necessary but the act of transmitting bad news must be present. The aim of this study is to investigate the perspective of students related to this subject.

**Methods**: Anonymous online surveys were applied to 70 fifth-year medical students, containing quantitative and qualitative questions, on the 'SPIKES' protocol and describing their perception of training for difficult news communication techniques during academic training.

**Results**: A total of 70 students completed the assessments, mean age 26.4 and 68.1% (n = 48) female. They were asked if they had practical training on the subject until their respective current periods and 77.1% (n = 54), denied having had any kind of practice, 84.3% said being not prepared to give difficult news to patients and family members, although the vast majority of participants demonstrated empathy during communication processes. At last we found that only a little more than half of the students had theoretical contact with protocols and preparation to give difficult news, and that most did not receive any practical training for such a scenario.

**Conclusions**: The medical students could gain competencies in breaking bad news using a practical approach, valued theoretical and practical ones. The implementation in teaching of these skills in medical schools is essential for greater safety of the professional in their daily practice and for the best care of patients and their relatives.

## P088

### Monocyte distribution width* in patients with COVID-19: indicator of disease severity

#### S. Naseem^1^, N. Varma^1^, I. Bihana^1^, P. Sharma^1^, N. Khaire^1^, P. Malhotra^1^, B. Verma^1^, S. Bastian^2^, E. Sukhacheva^3^

##### ^1^Post-Graduate Institute of Medical Education & Research, Chandigarh, India; ^2^Beckman Coulter India Pvt. Ltd., Mumbai, India; ^3^Medical and Scientific Affairs, Beckman Coulter Eurocenter, Nyon, Switzerland

*Critical Care* 2021, **25**(**Suppl 1**): P088

**Introduction**: Identification of patients with COVID-19, who may have severe illness is important to timely intervene and to decrease the fatality rate. In this study, we evaluated the utility of monocyte distribution width (MDW) as marker for severity detection and outcome in COVID-19 infection.

**Methods**: A total of 145 patients with PCR confirmed COVID-19 infection were included in the study. Complete blood count with differential count (CBC-Diff) was done in all patients on DxH 900 Automated Hematology Analyser (Beckman Coulter, USA). Patients were categorized into 2 groups: Group-1 (n = 122) - including asymptomatic patients and those with mild and moderate disease and Group 2 - including patients (n = 23) with severe disease. The performance of MDW was evaluated by calculating the area under the receiver operating characteristic curve (AUC), sensitivity, specificity, positive predictive value (PPV) and negative predictive value (NPV). Additional analysis was conducted for outcome, comparing COVID-19 patients who were discharged (n = 135) vs COVID-19 deceased patients (n = 10).

**Results**: MDW as a marker of disease severity demonstrated in ROC analysis AUC of 0.702 (95% CI 0.620–0.775). If MDW is considered as a marker of patient outcome, comparing COVID-19 deceased patients vs those who survived, AUC was 0.916 (95% CI 0.862–0.953). Sensitivity, specificity, PPV and NPV at different cut-offs for both scenario (for COVID-19 severity and for outcome) are presented in Table 1.

**Conclusions**: MDW can be considered as useful tool in predicting severity of COVID-19 disease and patient’s outcome.

*For scientific discussion only, the measurement of MDW on the UniCel DxH 900 analyzer is intended for use with adult patients presenting to the emergency department, on whom a white cell differential test has been ordered, as an aid in the early detection of patients with or developing sepsis.

**Table:** **Table 1** **(abstract P088)** MDW performance in COVID-19 patients as a marker of disease severity and patient outcome

MDW cut-off for severity	Sensitivity	Specificity	PPV	NPV
> 18.31	100%	9.8%	17.2%	100%
> 24.48	60.9%	77.9%	34.1%	91.3%
> 28.74	17.4%	95.1%	40.0%	85.9%
**MDW cut-off to predict outcome**	**Sensitivity**	**Specificity**	**PPV**	**NPV**
> 25.4	100%	79.2%	23.8%	100%
> 30.0	30%	95.5%	30%	95.5%

## P089

### Indications and measures of medical emergency teams - an evaluation of in-hospital emergency operations of the German Resuscitation Registry

#### G. Jansen^1^, S. Scholz^1^, S. Rehberg^1^, J. Wnent^2^, J. T. Gräsner^2^, S. Seewald^3^

##### ^1^Department of Anaesthesiology, Intensive Care, Emergency Medicine, Transfusion Medicine, and Pain Therapy, Protestant Hospital of the Bethel Foundation, University Hospital OWL, University of Bielefeld, Bielefeld, Germany; ^2^Institute for Emergency Medicine, University Hospital Schleswig–Holstein, Kiel, Germany; ^3^Department of Anesthesiology and Intensive Care Medicine, University Hospital Schleswig–Holstein, Kiel, Germany

*Critical Care* 2021, **25**(**Suppl 1**): P089

**Introduction**: The present study examines the characteristics and measures of in-hospital emergency interventions by Medical Emergency Teams (MET) using data from the German Resuscitation Registry.

**Methods**: Between 2014–2019, all in-hospital emergencies outside the operation theatre or intensive care unit from hospitals with established MET, defined as proportion of resuscitations to all MET calls < 50% in patients ≥ 18 years of age, were included. These were analyzed for age, gender, the emergency event as per the ABCDE scheme, the arrival and care times as well as the performed interventions (intubation, invasive blood pressure measurement, catecholamine therapy). The study was approved by the ethics committee of the University of Kiel (Ref. No.: D 648/20).

**Results**: Between 2014–2019, a total of 14,166 in-hospital emergencies (male 8,033 (57%); mean age 64 ± 18 years) were treated by MET from 62 German hospitals. The causes for MET activation were mostly C-problems (5,760 (41%)), followed by D-problems (4,076 (29%). A B-problem caused a MET-activation in 26% (n = 3,649) and an A-problem in 11% (n = 1,589). The MET arrived after 4 ± 3 min with a time of care of 24 ± 23 min. Table 1 shows selected invasive and therapeutic measures by MET. 201 (11%) had a difficult airway (> 1 intubation attempt and/or change of procedure) with a need for coniotomy in 8 of these patients (4%).

**Conclusions**: In-hospital emergency care is demanding and requires advanced competencies in invasive hemodynamic and airway interventions of the attending physicians. Taking these special features into account, there is a need for specific recommendations regarding training and equipment of MET, that need to consider the resources and the patient population of the respective hospitals.

**Table:** **Table 1** **(abstract P089)** Invasive and therapeutic measures by MET

	**Overall [n(%)]**	**A-B [n(%)]**	**C [n(%)]**	**D [n(%)]**
Intubation	1757(12)	937(24)	459(17)	488(18)
Invasive blood pressure	1074(8)	514(13)	394(15)	234(9)
Catecholamine therapy	2421(17)	1060(27)	1060(27)	492(18)
Epinephrine	430(3)	195(5)	205(8)	42(2)
Norepinephrine	1.520(11)	735(19)	729(27)	342(13)
Others	820(6)	316(8)	439(16)	199(7)

## P090

### Violence against women in Tunisia: post-traumatic stress disorder and COVID-19 pandemic impact

#### N. E. Nouira^1^, W. Demni^2^, H. Gharbi^3^, N. Masmoudi^3^, E. M. Ben Othmane^2^, A. Lahouegue^2^, D. Hamdi^2^, W. Bahria^2^, M. Ben Cheikh^2^

##### ^1^Emergency Department, Mongi SLIM Academic HospiTAL, Tunis, Tunisia; ^2^Emergency Medicine, Mongi SLIM Academic Hospital, Tunis, Tunisia; ^3^Psychological Assistance Unit, Mongi SLIM Academic Hospital, Tunis, Tunisia

*Critical Care* 2021, **25**(**Suppl 1**): P090

**Introduction**: The pandemic caused by COVID-19 has been an exceptional universal social, psychological and health emergency. Quarantine measures adopted to contain this infection may prompt episodes of aggressionspecially against women who are already victims of violence. The Aim: to assessthe physical and psychological damage of violenceagainst women and to report the impact of the COVID-19 on the upsurge of this aggression in a sample of the Tunisian population.

**Methods**: A prospective study, including 300 women consulting the emergency department for aggression between October 2017 and October 2019. Sociodemographic data was collected, physical damage and post-traumatic stress disorder (PTSD) was evaluated. With a telephonic survey within 6 months and during the COVID-19 confinement, by an emergency physician and a psychologist, to assess the recurrence of violence and how to assist these victims.

**Results**: The average age was 36 ± 10 years, 35% had a university degree. Physical, verbal and sexual abuse were reported respectively in 95%, 92% and 2%of the cases. Women suffered from high rate of PTSD (35 ± 20 SD), 38% required psychiatric follow-up and 10% required social assistance from women's protection associations. The average number of sick leave days was 10 ± 5. After 6 months 17% are still abused. A significant increase in domestic violence during the current pandemic was reported (p < 0.01; OR = 5; CI [1.02;20.19]).

**Conclusions**: Violence against women is a global public health problem. It takes many different forms and leads to significant physical and psychological consequences. Forced home isolation to contain COVID-19 infections in Tunisia has increase the domestic violence.

## P091

### Neurobehavioral and physiological effects of traumatic brain injury in spontaneously hypertensive rats

#### C. Bondi, E. H. Moschonas, R. Reddy, P. Rennerfeldt, B. Wehrmeyer, T. Ranellone, M. Toader, N. S. Race, J. P. Cheng, A. E. Kline

##### PMR, University of Pittsburgh, Pittsburgh, USA

*Critical Care* 2021, **25**(**Suppl 1**): P091

**Introduction**: This study investigates the combined effects of traumatic brain injury (TBI) and hypertension in rats on motor coordination, spatial learning, sustained attention, and anxiety. The hypothesis is that hypertension will worsen TBI-induced deficiencies. Hypertension afflicts nearly half of American adults, thus it is critical to investigate TBI models that take this pre-existing condition into account.

**Methods**: A pathophysiological study was conducted on spontaneously hypertensive rats (SHR) compared to normotensive Wistar Kyoto (WKY) rats. Rats were assigned to receive a controlled cortical impact (CCI; 2.8 mm cortical deformation depth, 4 m/s) or a sham injury. Both sham and TBI rats underwent the Beam Walking Task (motor) as well as the Morris Water Maze (MWM; spatial learning). Open field testing (OFT) was performed to examine anxiety, while Shock Probe Defensive Burying Task (SPDB) inspected passive/active coping behavior. 3-Choice Serial Reaction Time Task (3-CSRT) was used in a separate cohort of SHR rats to examine sustained attention and distractibility.

**Results**: Adult male SHR TBI rats have on average 10% higher heart rate and 30% higher mean arterial pressure versus injured WKY rats. Injured SHR rats demonstrated impaired motor skills as well as diminished spatial learning compared to sham rats. SHR TBI rats also spent less time actively burying the shock probe, and displayed reduced percent accuracy and increased omissions during 3-CSRT, suggesting impaired sustained attention. Results indicate that TBI in rats with a hypertensive phenotype renders neurobehavioral deficits across a variety of behavioral tasks.

**Conclusions**: Findings from this array of behavioral paradigms establish neurobehavioral deficits in injured, hypertensive animals, with current work exploring comparisons with normotensive rats. In order to develop new methods of treatment, it is critical to understand the influence that underlying conditions, such as hypertension, have on TBI pre-clinically.

## P092

### Prevalence of clinically significant head injury among patients intubated in the field due to suspected severe traumatic brain injury

#### D. Epstein^1^, S. Rakedzon^2^, B. Kaplan^3^, H. Ben Lulu^4^, J. Chen^5^, N. Samuel^6^, A. Lipsky^7^, A. Miller^8^, H. Bahouth^4^, A. Raz^9^

##### ^1^Critical Care Division, Rambam Health Care Campus, Haifa, Israel; ^2^Department of Internal Medicine B, Rambam Health Care Campus, Haifa, Israel; ^3^Ruth and Bruce Rappaport Faculty of Medicine, Technion, Haifa, Israel; ^4^Trauma and Emergency Surgery, Rambam Health Care Campus, Haifa, Israel; ^5^Meir Medical Center, Kfar Saba, Israel; ^6^Pediatric Emergency Medicine Departement, Schneider Children´s Medical Center, Petah Tikva, Israel; ^7^Emergency Department, Emek Medical Center, Afula, Israel; ^8^Medical Intensive Care Unit, Rambam Health Care Campus, Haifa, Israel; ^9^Department of Anesthesiology, Rambam Health Care Campus, Haifa, Israel

*Critical Care* 2021, **25**(**Suppl 1**): P092

Introduction: Prehospital care of severe traumatic brain injury (TBI) focuses on preventing the secondary insult caused by hypoxemia and hypotension. Current guidelines advocate early endotracheal intubation (ETI) in patients with suspected severe TBI. Although potentially beneficial, prehospital ETI is associated with a high rate of complications. The ability to accurately diagnose TBI in the field is limited. We investigated the prevalence of clinically significant TBI among patients intubated in the field due to presumed severe TBI.

**Methods**: Data were retrospectively collected from EMS and hospital records of trauma patients for whom ETI was attempted on the scene (either successfully or not) and who were transferred to Rambam Health Care Campus, Israel between 2014 and 2020. The indication for ETI was extracted. The primary outcome was clinically significant head trauma (a significant lesion on head CT, need for neurosurgery, or abnormal neurologic examination after extubation) among patients intubated due to suspected severe TBI. We excluded patients intubated due to non-traumatic conditions, burns, during CPR, facial trauma compromising airway, hypoxemia, and severe shock.

**Results**: A total of 349 patients were included in the final analysis (Fig. 1) - 95.7% suffered blunt trauma, 82.8% were male, and the median age was 34 years (IQR 23–57). A total of 253 patients (72.5%) had clinically significant head trauma. In a multivariable analysis, risk factors for significant head injury were GCS < 9 (OR 3.58, 95% CI 1.96–7.69, p < 0.001) and alcohol intoxication (OR 0.16, 95% CI 0.06–0.46, p < 0.001).

**Conclusions**: A substantial portion of patients intubated in the field due to suspected severe TBI, did not suffer a clinically significant head injury. This population may be exposed to the risks and complications of prehospital ETI without any potential benefits. Patients with a higher field GCS and those suffering from intoxication have a higher risk of misdiagnosis and potentially unnecessary ETI.

**Figure:**
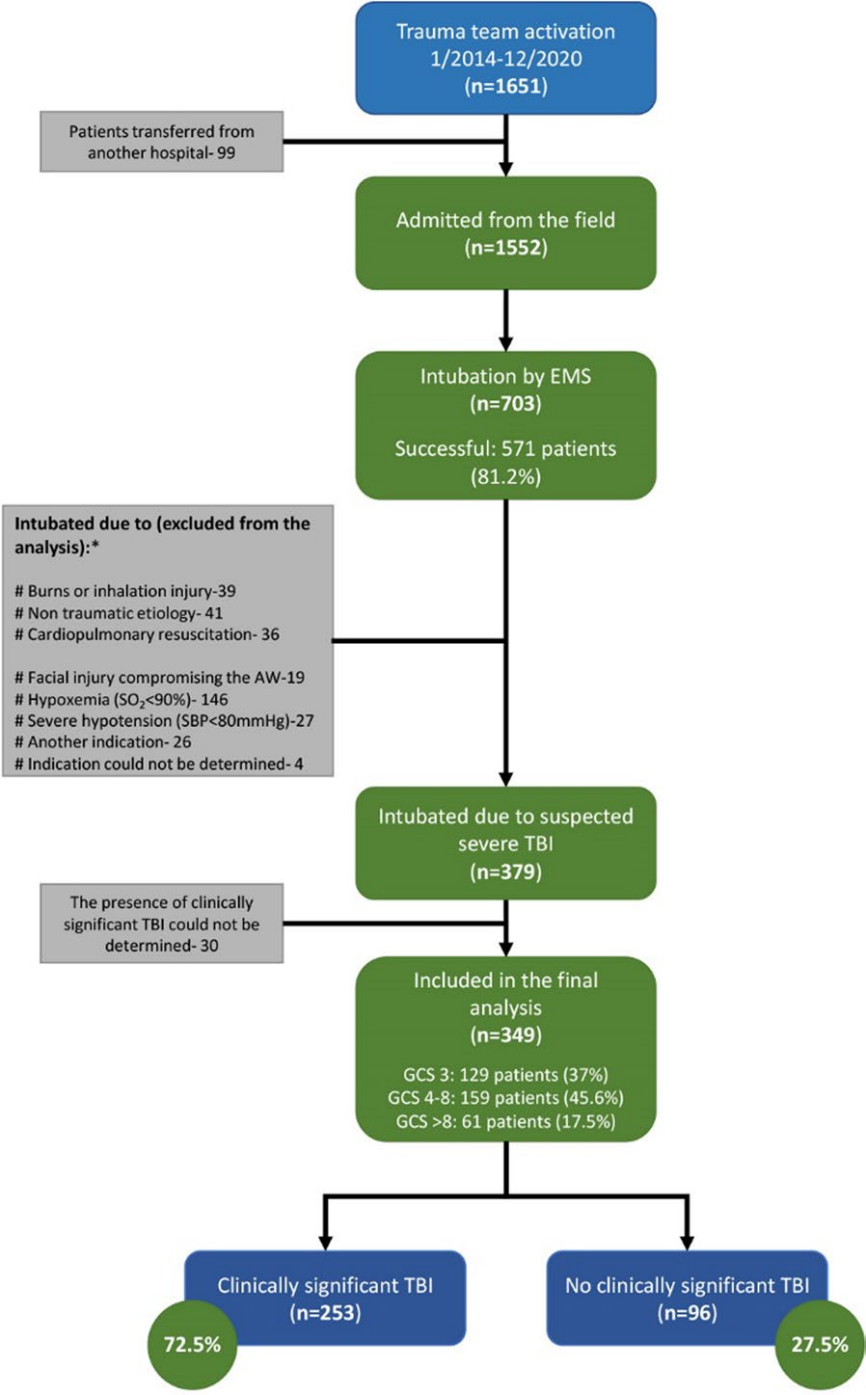
**Fig. 1** **(abstract P092)** Study flow chart

## P093

### Intracranial compliance does not ameliorate with decompressive craniectomy

#### S. Brasil, D. S. Solla

##### Neurology, University of São Paulo, São Paulo, Brazil

*Critical Care* 2021, **25**(**Suppl 1**): P093

**Introduction**: Decompressive craniectomy (DC) has demonstrated to be effective for intracranial pressure (ICP) control, although not relevant results have been observed concerning mortality reduction in TBI. One hypothesis for this outcome is that DC does not return intracranial compliance (ICC) for its previous state, before acute injury. The present study aimed to assess the morphological alterations in intracranial pressure pulse waveform (ICPPW) among neurocritical care patients with and without DC, by comparing the variations of ICPPW according to elevations in ICP by two techniques.

**Methods**: Patients requiring ICP monitoring because of severe traumatic or spontaneous conditions were included. ICP mean values were compared with ICPPW features (P2/P1 ratio, time-to-peak [TTP]) obtained from ICP invasive catheters and a new technology that captures beat-by-beat skull pulsations (B4C). Elevation of ICP was produced by means of ultrasound-guided internal jugular veins compression. Analysis of ICPPW features were compared between techniques and distributed for three groups: intact skull (exclusive burr hole for ICP monitoring), craniotomy/large fractures (group 2) or DC (group 3).

**Results**: 57 patients were analyzed, 32 with both techniques. 21 (36%) presented no skull defects, whereas 15 (26%) had DC. ICP was not significantly different between groups (p = 0.56). Significant elevation was observed for P2/P1 ratio for groups 1 and 2, whereas reduction was observed in group 3 (elevation of ± 0.09 for groups 1 and 2, whereas reduction of 0.03 for group 3, p = 0.01). B4C disclosed strong correlation with invasive ICPPW features (AUC 0.86 for P2/P1 ratio, 0.81 for TTP).

**Conclusions**: Intracranial compliance was significantly more impaired among decompressive craniectomy patients, although this technique seems to be protective for further influences of ICP elevations over the brain. Correlation between techniques was significant, indicating the B4C system as promising to monitor intracranial compliance noninvasively.

## P094

### Apoptosis activation in bronchoalveolar lavage fluid (BALF) corresponded with severity of brain injury-preliminary study

#### D. S. Siwicka-Gieroba^1^, S. T. Terpilowska^2^, M. B. Barud^1^, W. D. Dabrowski^1^

##### ^1^Anaesthesiology and Intensive Care, Medical University in Lublin, Lublin, Poland; ^2^Laboratory of Environmental Biology, Institute of Environmental Engineering, The John Paul II Catholic University of Lublin, Lublin, Poland

*Critical Care* 2021, **25**(**Suppl 1**): P094

**Introduction**: The mechanism of acute brain injury initiates cascades of consequences which are significant factors of poor neurological outcome [1–6].

**Methods**: The analysis was performed in patients with severe isolated TBI. Bronchoalveolar lavage fluid was collected at admission, for third and seventh day after incident. Activation of intrinistic, extrinistic and endoplasmic reticulum pathways were measured.

**Results**: Results showed significantly increased levels of selected apoptotic factors concentration after 72 h and on the 7thday after incident. We found a significant correlation between apoptotic factors, GCS and 28-day mortality. There were no statistically significant correlations between apoptosis and EVLWI and PVPI.

**Conclusions**: Activation and imbalance of apoptotic pathways seems to be an important process in lungs after severe brain trauma. Activation of apoptosis correlates with the severity of brain injury.

**References**

1. Pelosi P et al. Curr Opin Crit Care 11: 37–42, 2005
2. López-Aguilar J et al. Crit Care Med 33: 1077–83, 2005.
3. Tagami T et al. Ann Intensive Care 4: 27, 2014.
4. Martin LJ et al. J Comp Neurol 433: 299–311, 2001.
5. Fortin A et al. J Cell Biol 155: 207–16, 2001.
6. Lee KS et al. Respir Med 102:464–9, 2008

## P095

### Computational subphenotypes of traumatic brain injury in the ICU stratum

#### H. Kim, R. Stevens

##### Department of Anesthesiology and Critical Care Medicine, Johns Hopkins University School of Medicine, Baltimore, USA

*Critical Care* 2021, **25**(**Suppl 1**): P095

**Introduction**: Heterogeneity represents a major barrier in efforts to find effective treatments for patients with moderate and severe traumatic brain injury (TBI). One approach to address heterogeneity might be to resolve undifferentiated populations into subphenotypes which would have greater intrinsic biological uniformity. We hypothesized that clinically meaningful subphenotypes can be identified using unsupervised computational approaches applied to electronic health records and physiological time series (PTS) data.

**Methods**: Adult patients admitted to the ICU for management of TBI were selected from a multi-center database (eICU), and clinical, laboratory and physiological time series data were extracted. Unsupervised clustering algorithms were implemented accounting for mixed data types. Clusters were characterized according to outcome distributions at discharge, and differences in physiology. The clustering algorithm was validated externally on an analogous TBI population in an independent, single-center dataset (MIMIC-III).

**Results**: Among 4,450 patients admitted to intensive care with TBI, we identified four clusters (a, b, c, d) each with a distinct outcome probability distribution, and each associated with unique, clinically relevant pattern of laboratory/PTS signatures. Subphenotype (a) captured TBI patients who had the highest likelihood of survival and favorable neurological outcome, while patients in subphenotype (d) had the highest risk of death and unfavorable neurological outcome. Results were reproduced in the MIMIC III cohort when eICU clusters were assigned using a multi-class classification (Fig. 1).

**Conclusions**: Using unsupervised machine learning, we identified four distinct and clinically meaningful clusters in a large sample of TBI patents admitted to the ICU. Patients assigned to specific clusters had distinct outcome probabilities and unique data signatures suggesting that they are plausible candidate subphenotypes.

**Figure:**
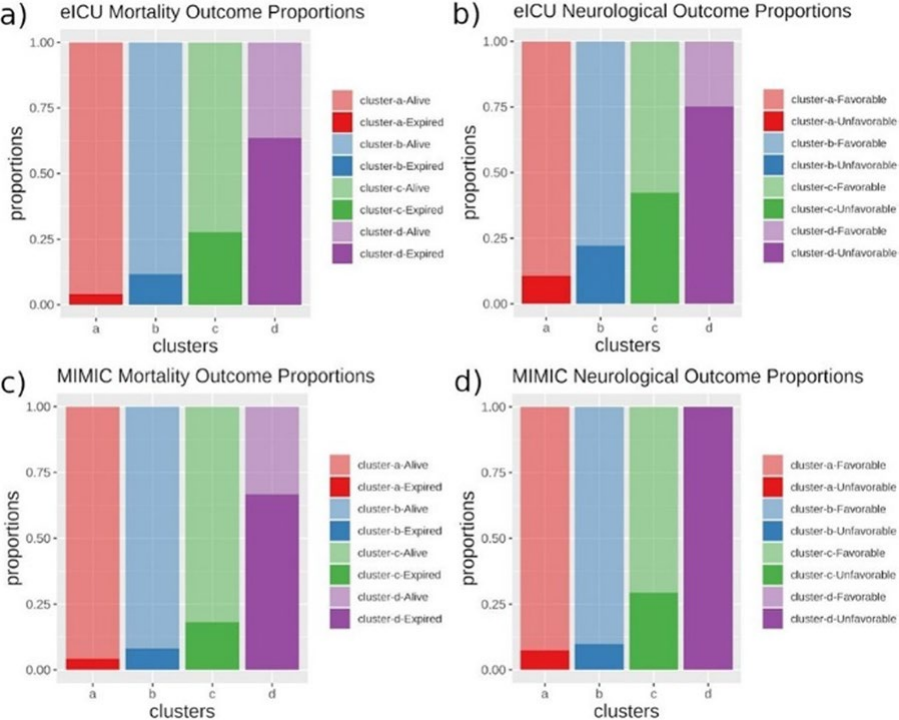
**Fig. 1** **(abstract P095)** Mortality and neurological outcome proportions corresponding to subphenotypes discovered in eICU (a, b) and externally validated in MIMIC III (c, d)

## P096

### Predictive data signatures in ICU-stratum traumatic brain injury patients: computational approach

#### H. Kim, R. Stevens

##### Department of Anesthesiology and Critical Care Medicine, Johns Hopkins University School of Medicine, Baltimore, USA

*Critical Care* 2021, **25**(**Suppl 1**): P096

**Introduction**: Traumatic brain injury (TBI) is a global health problem which constitutes a leading neurological cause of death and also results in life-long disability in many survivors. The aim of this study was to identify data signatures in TBI patients recorded in the first 24 h of ICU admission. We tested the hypothesis that these signatures are associated with short term clinical outcomes.

**Methods**: A total of 4,450 patients admitted to the ICU for management of TBI were selected from the multisite clinical database (eICU) and clinical, laboratory and physiological time series data were extracted. Outcomes were mortality and neurological outcome at discharge. The eICU developed machine learning algorithms were externally validated on an analogous TBI sample in an independent single-center ICU database (MIMIC-III). Model performance metrics were compared with reference International Mission for Prognosis and Analysis of Clinical Trials (IMPACT) and Corticoid Randomization After Significant Head injury (CRASH) logistic regression models.

**Results**: The computational models performed well for both neurological outcome prediction and mortality prediction, with test metrics that compared favorably with reference IMPACT and CRASH scores (Fig. 1). External validation utilizing MIMIC III corroborated the results from eICU for both neurological outcome and mortality suggesting robust generalizability. Top predictive features included age, PTS derived variables, glucose, platelet count, white blood cell count, and Glasgow Coma subscores, all originating from the first 24 h of ICU admission.

**Conclusions**: Results indicate that computational models trained with data available in the first 24 h after admission are predictive of short-term neurological outcome and mortality in ICU-stratum TBI patients. Timely characterization of severity and clinical trajectories could open a window for targeted interventions to ameliorate outcomes in patients with moderate and severe TBI.

**Figure:**
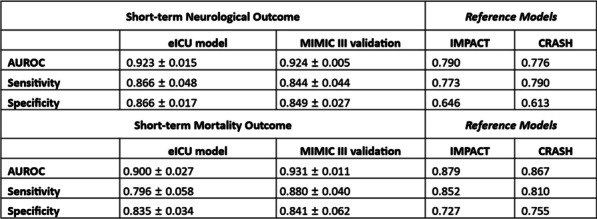
**Fig. 1** **(abstract P096)** Summarized eICU development and MIMIC III external validation results for short-term neurological outcome and mortality prediction

## P097

### New therapy intensity level (TIL) scale coefficient calculator: a helpful, insightful tool

#### N. Peeters^1^, S. Meeuws^2^, S. Vanvolsem^2^, T. Menovsky^3^

##### ^1^Intensive Care Unit, AZ Klina, Brasschaat, Belgium; ^2^Neurosurgery, Jessa Hospital, Hasselt, Belgium; ^3^Neurosurgery, University Hospital Antwerp, Edegem, Belgium

*Critical Care* 2021, **25**(**Suppl 1**): P097

**Introduction**: In ICU, raised ICP is a frequently encountered problem for which different management strategies can be adopted [1]. Intensity of these interventions can be quantified using the TIL scale, a reliable measurement with a high degree of validity for assessing ICP management in patients with traumatic brain injury (TBI) [2]. Studies showed significant center variation in TIL treatments for patients with TBI, possibly reflecting aggressive use or inappropriate escalation strategies [3].

**Methods**: We developed a user-friendly TIL scale calculator to improve ICP management. Based on the intuitively started ICP management, current ICP and desired target ICP, a TIL-coefficient will be calculated, which will numerically and visually reflect whether past and current treatment is appropriate, inadequate or overly aggressive. Additionally, a TIL scale-based algorithm can optimize ICP management. Hereby, inappropriate escalation or de-escalation could be avoided. The indication, relevance and ease of use of the TIL scale calculator was peer reviewed in three Belgian hospitals.

**Results**: The TIL scale calculator, with both TIL-coefficient and TIL scale algorithm, resulted in more awareness regarding ICP management. The numerical and visual feedback of past and current ICP management and possible treatment modalities was an advantageous tool, leading to more evidence-based decision making. However, suggestions from the TIL scale based algorithm should always be critically assessed prior to implementation in daily clinical practice.

**Conclusions**: This user-friendly TIL scale calculator is based on the TIL scale, which has been proven to be reliable and valid. Implementing this tool in daily clinical practice can lead to more specific and targeted ICP management. To assess reliability and validity of ICP management based on the TIL scale calculator, further studies are warranted.

**References**

1. Battaglini D et al. Front Neurol 11:602,114, 2020
2. Zuercher P et al. J Neurotrauma 33:1768–1774, 2016.
3. Huijben J et al. Crit Care 25:78, 2021

## P098

### HTR1A C(-1019)G polymorphism contributes to neurological impairment in a cohort of neuro-ICU patients

#### V. Pisarev^1^, A. Chumachenko^2^, I. Redkin^2^, V. Zakharchenko^2^, A. Kalov^2^, M. Petrova^2^

##### ^1^Federal Research and Clinical Center of Intensive Care Medicine and Rehabilitology, V.A.Negovsky Institute of General Reanimatology, Moscow, Russian Federation; ^2^Federal Research and Clinical Center of Intensive Care Medicine and Rehabilitology, Moscow, Russian Federation

*Critical Care* 2021, **25**(**Suppl 1**): P098

**Introduction**: The 1A receptor HTR1A is one of the most abundant serotonin receptors in the brain and immune cells that contribute to stress, anxiety, aggression, cognition, and immune responses [1, 2]. Single nucleotide polymorphism (SNP) HTR1A C(-1019)G (rs6295) site is located in promoter area and affects HTR1A gene transcription [1]. Allele G associates with depression, post-traumatic mental disorders, and resistance to antipsychotic drugs [1]. Altered expression of the HTR1A gene associates with increased oxidation due to a deficient anti-oxidation mechanism [3]. Our study aimed to investigate whether the HTR1A SNP links to neurological impairment and circulating oxidized DNA (oxDNA) in patients re-admitted to a neuro ICU.

**Methods**: Study cohort included 240 neuro ICU patients (median age 54 years, range 18–88 years) with consequences of trauma, anoxic brain injury, stroke, brain tumor. HTR1A rs6295 polymorphism was studied using an HTR1A specific oligonucleotide tetra primer set, PCR, and gel electrophoresis. Oxidized DNA (oxDNA) concentration in plasma was determined with antibodies to 8-oxo-2’-deoxyguanosine and membrane immunoassay.

**Results**: On day 1, NIHSS scores (but not Glasgow or SOFA scales) revealed differences between patients with CC genotype vs. G carriers: medians 8 (6.5;12.5) vs. 13 (9.2;18.0), respectively (p = 0.012, Mann–Whitney) (Fig. 1). Increased oxDNA values were associated with CC genotypes: odds ratio (OR) 2.213, 95%CI%: 1.201–4.077, p = 0.016 (Fisher test), n = 240. This association was significant only in a cohort of patients with no pneumonia (OR 2.991, 95%CI%: 1.170–7.645, p = 0.036, n = 92).

**Conclusions**: The results link enhanced neurologic impairment and decreased oxDNA in circulation in the cohort of carriers of HTR1A G resistant to a lung infection that may stem from the dual effect of the mutant gene in neural and immune cells.

**References**

1. Soga T et al. Front Genetics 11: 601,868, 2021.
2. Liu Y et al. Eur J Cancer 114:8–24, 2019.
3. Mössner R et al. J Neural Transm (Vienna) 109:557–65, 2002.

**Figure:**
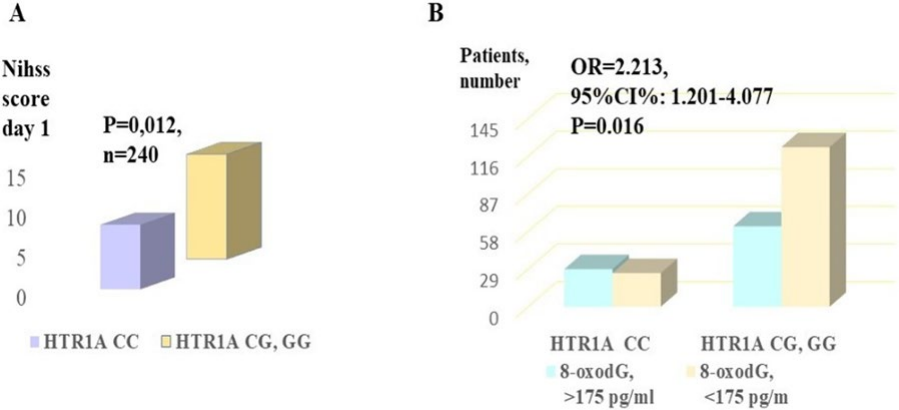
**Fig. 1** **(abstract P098)** HTR1A polymorphism impacts NIHSS scores and circulating oxidized DNA in plasma

## P099

### PET-pathomorphological study of skeletal muscles in patients with chronic disorders of consciousness and critical illness polyneuromyopathies

#### S. Kondratev^1^, E. Skiteva^2^, Y. U. Zabrodskaya^2^, D. Ryzkova^3^, E. Kondratyeva^4^, A. Kondratyev^5^

##### ^1^Intensive Care Department, Russian Neurosurgical Institute named after Polenov, St-Petersburg, Russian Federation; ^2^Pathomorphology, Russian Neurosurgical Institute named after Polenov, St-Petersburg, Russian Federation; ^3^Radioisotope Diagnostics, Almazov National Medical Research Centre, St-Petersburg, Russian Federation; ^4^Chief Investigator, Russian Neurosurgical Institute named after Polenov, St-Petersburg, Russian Federation; ^5^Head Of ICU, Russian Neurosurgical Institute named after Polenov, St-Petersburg, Russian Federation

*Critical Care* 2021, **25**(**Suppl 1**): P099

**Introduction**: A comprehensive study of 23 patients with chronic disoders of consciousness (unresponsive wakefulness syndrome, minimally conscious state), treated in the ICU department of Russian Polenov Neurosurgical Institute, was conducted in order to clarify the mechanisms of impaired functions and the possibility of regeneration of striated muscles in patients with severe damage of the central nervous system.

**Methods**: All patients underwent PET-CT with 18F-fluorodeoxyglucose (18F-FDG) brain, skeletal muscles of the shoulder girdle, to identify the asymmetry of muscle metabolism comparison with the scale of muscle tone. 18F-FDG was administered intravenously in a dose125-250 MBq. For pathomorphological examination, muscles tissue was taken by surgeon under local anesthesia with a 1% solution of novocaine, the size of the muscle biopsies was 1 × 1 cm histological examination included fixation of the biopsies in buffered 10% neutral formalin, conducting alcohol wiring and filling in paraffin.Immunohistochemical (IHC) reactions were performed on paraffin sections according to the standard protocol, with the antigen unmasking in a water bath. Primary antibodies were used: dystrophin (ab85302), beclin-1 (ab62557), myosin (Fast, DBS), desmin (DBS).

**Results**: Structural changes in skeletal muscles in DOC patients with CIP have a progressive nonspecific degenerative-atrophic character with more pronounced manifestations on the side of paresis. Intracellular and metabolic changes indicate that, despite the deep-seated process, changes are usually reversible and the regenerative potential of muscle fibers is preserved. Analysis of 18F-FDG metabolism of the shoulder girdle muscles in patients with CHF showed its decrease regardless of the side of paresis.

**Conclusions**: A clear correlation of the metabolism level and changes in muscle tone, deep reflexes was not found, but a certain priority was found in the skeletal muscles innervated by the dominant hemisphere.

**Acknowledgement**: Supported by RFBR grant 19–29-01,066.

## P100

### Machine learning and heart rate variability to detect cerebral ischemia

#### R. Vithal^1^, A. El-Mehri^2^, H. Odenstedt Hergès^2^, M. Staron^3^, L. Block^2^

##### ^1^Institute of Clinical Science, Sahlgrenska Academy, University of Gothenburg, Department of Anesthesiology and Intensive Care, Gothenburg, Sweden; ^2^Institute of Clinical Science, Sahlgrenska Academy, University of Gothenburg, Gothenburg, Sweden; ^3^Department of Computer Science and Engineering, IT Faculty, Chalmers, University of Gothenburg, Gothenburg, Sweden

*Critical Care* 2021, **25**(**Suppl 1**): P100

**Introduction**: Detecting delayed cerebral ischemia (DCI) in patients with aneurysmal subarachnoid hemorrhage (aSAH) is challenging for the clinician. The lack of precise monitoring in these patients plays a major role in the delayed identification of DCI. The main objective of this study is to analyze heart rate variability (HRV) data from SAH patients for further analysis, using machine-learning to aid us in identifying imminent DCI. We hypothesize that machine learning, may detect incipient DCI using HRV trends.

**Methods**: HRV data from a previous study underwent comprehensive analysis. The HRV material was collected prospectively from a cohort of 64 patients with aSAH admitted to the neurointensive care unit at Sahlgrenska University Hospital, Gothenburg, Sweden, 2015–2016. HRV data was cleaned from noise, quantified and labelled in multiple steps and analyzed using the Random Forest supervised machine learning algorithm.

**Results**: HRV data was obtained in 55 patients. After excluding 19 patients due to low quality data, a total of 36 patients remained, 12 of which developed DCI. The machine learning algorithm was able to identify 71% of patients with DCI. Nevertheless, DCI was also detected in non-DCI patients, demonstrating a specificity of 57%

**Conclusions**: This study implies that processing of HRV, using machine learning can be helpful in patients susceptible to DCI. The results display an adequate sensitivity; however, the specificity was low. Our data support further investigation, correlating HRV and cerebral ischemia. More studies are needed to further evolve a secure method to detect cerebral ischemia using dynamic HRV values.

## P101

### Machine learning for detection of changes in cerebral perfusion

#### A. El-Merhi^1^, R. Vithal^1^, J. Liljencrantz^1^, M. Elam^2^, M. Staron^3^, L. Block^1^, H. Odenstedt-Hergés^1^

##### ^1^Department of Anaesthesia and Intensive Care Medicine, Sahlgrenska University Hospital, Gothenburg, Sweden; ^2^Department of Clinical Neurophysiology, Sahlgrenska University Hospital, Gothenburg, Sweden, ^3^Department of Computer Science and Engineering, University of Gothenburg, Gothenburg, Sweden

*Critical Care* 2021, **25**(**Suppl 1**): P101

**Introduction**: Detection of cerebral ischemia in the unconscious patient is challenging for clinicians. Patients treated in the ICU or operating room (OR) are continuously monitored with several parameters, yielding large amounts of physiological data. We hypothesize that a machine learning (ML) algorithm can detect events with cerebral hypoperfusion and ischemia in patients in the ICU or OR.

**Methods**: In a first of a three-phase observational study with the aim of training a ML-algorithm to predict and detect cerebral ischemia using data collected from bedside monitors, we studied patients undergoing carotid endarterectomy. Patients were monitored with SpO_2_, continuous ABP, ECG, near-infrared spectrometry (NIRS), and electroencephalography. Data was exported to a computer, cleaned, and labelled. The perioperative period was divided into 7 events. Feature extraction of the data was done in Python programming environment. Heart rate variability parameters were extracted from the ECG. We quantified all variables per minute. This provided us with a large matrix of all features for each minute (data point). The data points were then labelled with the event name. The feature matrix was used to train a random forest algorithm.

**Results**: Nine patients were included in a pilot study. Recognition of the clinical events was achieved with an accuracy of 82–98%, precision (specificity) 86–98%, and recall (sensitivity) 87–98%. Accuracy was not affected when NIRS-values were excluded from analysis. Using decision tree or support vector machine algorithms yielded similar results.

**Conclusions**: It is possible for a ML-algorithm to differ between clinical events, including clamping of the carotid arteries, in a controlled environment. Artificial intelligence and ML can help utilize big data generated in the ICU and OR. We will continue our research to help develop models able to aid clinicians detect cerebral ischemia in real time, using data generated from bedside monitors.

## P102

### Association between ECG changes and prognosis in subarachnoid hemorrhage

#### M. Rabaey, H. Schaubroeck, E. Hoste, K. Colpaert

##### Intensive Care, Ghent University Hospital, Ghent, Belgium

*Critical Care* 2021, **25**(**Suppl 1**): P102

**Introduction**: Cardiac complications are frequently seen in patients with subarachnoid hemorrhage (SAH). Previous studies that examined the relation between electrocardiographic and echocardiographic (echo) changes in SAH patients and patient outcomes were conflicting [1, 2].

**Methods**: In this single-center retrospective cohort study, data was collected from ICU patients with an aneurysmal SAH between 01/06/2017 and 31/12/2020. We reviewed electrocardiogram (ECG) on admission and defined an abnormal ECG as either a significant ST-elevation or depression, a significant T-wave inversion or an abnormal QTc (> 440 ms for men, > 460 ms for women). We collected echo findings (left ventricular ejection fraction, EF) when available. Univariate regression analysis or Chi-square testing was used wherever applicable.

**Results**: A total of 150 SAH patients were identified (patient characteristics in Table 1). 74 patients had a normal ECG (group 1), in 71 patients ECG changes were found (group 2), 5 patients had no ECG on admission and were excluded. A prolonged QTc was found in 66%, ST-T changes in 12%, both in 21%. A total of 7 patients received an echo in group 1 (9%) compared to 18 in group 2 (25%). Overall, 10 echoes showed reduced EF (0% in group 1 vs 55% in group 2). Mean ICU length of stay (LOS) was lower in group 1 compared to group 2 (7.7 days vs 12.2 days resp, p = 0.003). Hospital mortality was lower in group 1 (6.7% vs 20.0%, p = 0.021). Reduced EF was not associated with higher mortality.

**Conclusions**: ECG changes in SAH patients are associated with longer ICU LOS and higher hospital mortality. Echocardiography was performed only in 25% of patients with ECG changes. Since more than 50% of echoes in patients with SAH having an abnormal ECG showed reduced left ventricular function, an abnormal ECG on admission in patients with SAH should trigger to perform an echocardiography to detect cardiac complications.

**References**

1. Sakr et al. Int J Cardiol 96: 369 – 373, 2004.
2. Zhang et al. J. Stroke Cerebrovasc Dis 25: 2653–2659, 2016.

**Table:** **Table 1** **(abstract P102)** Baseline characteristics

	**Normal ECG (n = 74)**	**Abnormal ECG (n = 71)**	**p value**
Age (median, years)	54 [IQR 20]	57 [IQR 20]	0.159
Female gender	59%	62%	0.757
Active smoking	36%	38%	0.848
Arterial hypertension	15%	30%	0.033
Cardiovascular disease (stroke, PAD, CAD)	5%	13%	0.126

PAD = peripheral artery disease, CAD = coronary artery disease

## P103

### Effect of lithium chloride on mortality in ischemic stroke in rats

#### R. A. Cherpakov^1^, O. A. Grebenchikov^1^, A. V. Ershov^1^, A. K. Shabanov^2^, V. V. Antonova^1^, A. N. Kuzovlev^1^

##### ^1^Federal Research and Clinical Center of Intensive Care Medicine and Rehabilitology, Moscow, Russian Federation; ^2^The Moscow Department of Health N.V. Sklifosovsky Federal Research Institute of Emergency Medicine, Moscow, Russian Federation

*Critical Care* 2021, **25**(**Suppl 1**): P103

**Introduction**: The aim of the study was to evaluate the effect of lithium chloride in various dosages on mortality in ischemic stroke in rats.

**Methods**: The study used male rats weighing 312 ± 12.5 g. The model of Long's focal ischemia was used as a basis. The animals were split into 5 groups: false-operated, control (model of ischemic stroke with the introduction of 0.9% NaCl) and three groups in which the simulation of ischemic stroke was combined with the introduction of lithium chloride in various concentrations (4.2 mg/kg, 21 mg/kg and 63 mg/kg, respectively). The drug was administered daily for 14 days. Daily, cumulative, and final mortality rates were evaluated.

**Results**: According to the results of the experiment, the following data on mortality were obtained: false – operated rats – 0 out of 8, control group – 13 out of 22 (lethality 59%), group III (LiCl 4.2 mg/kg) – 8 out of 14 (lethality 57%), p > 0.05 relative to the control, group IV (LiCl 21 mg/kg) - 6 out of 15 (lethality 40%) p > 0.05 relative to the control and in group V (LiCl 63 mg/kg), 4 out of 15 animals died (lethality 27%) p = 0.0317 relative to the control. The highest mortality rate in the control group was observed on day 1 (22.7%). In groups IV and V, the daily mortality rate was evenly distributed, in group III the largest number of animals died on day 3 (3 out of 14; 21.42%). The cumulative mortality rate in group III significantly differed from the control one only on day 2 (p < 0.05). In group IV, cumulative mortality was significantly lower up to 7 days (p < 0.05). In group V, cumulative mortality was significantly lower (p < 0.05) until 14 days after euthanasia.

**Conclusions**: Long-term administration of lithium chloride at a dose of 63 mg/kg significantly reduced the mortality of laboratory animals after a stroke. The effect of the drug at a dose of 21 mg/kg was less pronounced. The dose of 4.2 mg/kg showed no neuroprotective effect.

## P104

### Effect of lithium chloride on the volume of ischemic stroke in rats

#### R. A. Cherpakov^1^, O. A. Grebenchikov^1^, A. V. Ershov^1^, A. K. Shabanov^2^, V. V. Antonova^1^, A. N. Kuzovlev^1^

##### ^1^Federal Research and Clinical Center of Intensive Care Medicine and Rehabilitology, Moscow, Russian Federation; ^2^The Moscow Department of Health N.V. Sklifosovsky Federal Research Institute of Emergency Medicine, Moscow, Russian Federation

*Critical Care* 2021, **25**(**Suppl 1**): P104

**Introduction**: The aim of the study was to evaluate the effect of lithium chloride in various dosages on the volume of brain damage in ischemic stroke in rats.

**Methods**: The study used male rats weighing 315 ± 13.5 g. The long focal ischemia model was used. The animals were split into five groups: false-operated (median incision in the projection of the carotid artery without ischemia), control (model of ischemic stroke with the introduction of 0.9% NaCl) and three groups with the introduction of lithium chloride at concentrations of 4.2 mg/kg, 21 mg/kg and 63 mg/kg, respectively. Lithium chloride was administered immediately after the termination of the occlusion of the middle cerebral artery and then every 24 h until euthanasia. To assess the degree of brain damage, the animals underwent magnetic resonance imaging (MRI) on day 2, and brain sections stained with 2,3,5-triphenyltetrazolium chloride were evaluated on day 7 after euthanasia.

**Results**: According to MRI data, lithium chloride at a dose of 4.2 mg/kg did not significantly affect the volume of ischemic stroke and perifocal edema in relation to the control group on day 2 (p = 0.9). When using a dose of 21 mg/kg, the volume of stroke (p = 0.04) and perifocal edema was significantly lower (p = 0.03) than in the control group (by 25% and 18%, respectively). Lithium chloride at a dose of 63 mg/kg significantly reduced the volume of stroke (45%, p = 0.004) and perifocal edema (35%, p = 0.007). When determining the volume of the lesion on day 7, the data were comparable with the results obtained on day 2. When using a dose of 21 mg/kg, the stroke volume was 20% lower (p = 0.04) than in the control group. Lithium chloride at a dose of 63 mg/kg reduced stroke volume by 40% (p = 0.0037).

**Conclusions**: Lithium chloride at dosages of 21 mg/kg and 63 mg/kg significantly reduced the volume of ischemic stroke and perifocal edema of the brain, but when using a concentration of 63 mg/kg, the effect was more pronounced.

## P105

### Influence of patient characteristics on quality indicators of stroke treatment: a retrospective single center analysis over four years

#### A. Lesenne^1^, L. Ernon^2^, K. Bekelaar^2^, L. Stockx^3^, T. De Beule^3^, P. Vanelderen^4^, M. Vander Laenen^4^, D. Mesotten^4^, S. Ordies^4^

##### ^1^Department of Anesthesiology and Perioperative Medicine, Ghent University Hospital, Ghent, Belgium; ^2^Department of Neurology, Ziekenhuis Oost-Limburg Genk, Genk, Belgium; ^3^Department of Medical Imaging, Ziekenhuis Oost-Limburg Genk, Genk, Belgium; ^4^Department of Critical Care Services, Ziekenhuis Oost-Limburg Genk, Genk, Belgium

*Critical Care* 2021, **25**(**Suppl 1**): P105

**Introduction**: Worldwide, ischemic stroke is a major cause of death and morbidity. Important target times to improve patient outcome are door-to-CT time, door-to-needle time (DNT) and door-to-groin time (DGT). Since 2017 stroke data was prospectively collected in the framework of stroke center accreditation in ZOL Genk, Belgium. We hypothesized that this would improve target times and enlarge the proportion of treated patients.

**Methods**: All patients admitted for stroke or TIA at ZOL stroke center, Genk, Belgium, between 2017 and 2020 were included. Quality indicators were proportion of treated patients, door-to-CT time, DNT and DGT. A structured data collection and a stroke working flow template were created to enhance and harmonize stroke diagnostics and treatment in 2019. As of 2020, intravenous thrombolysis was administered immediately at the CT scan.

**Results**: In total 1255 patients were included. The number of patients registered with stroke and TIA increased by 80% over 4 years (p < 0.0001). Median age differed over time: 78 years in 2017 vs. 74 years in 2020 (p = 0.03). NIHSS at admission decreased over the last two years (p < 0.0001). In addition, onset-to-door time, door-to-CT time and DNT were different (p < 0.0001, p = 0.0005, p < 0.0001). DGT was similar between consecutive years (p = 0.54). The proportion of conservatively treated patients increased over time by 15% (p = 0.0001) (Table 1).

**Conclusions**: Patients were younger and presented with milder strokes in comparison with earlier years. Increased detection of stroke patients was probably due to underreporting of milder strokes in the past. The higher proportion of conservatively treated patients hereby may have affected target times such as a prolonged door-to-CT time. The sudden drop of DNT in 2020 can be explained by administration of thrombolysis already at the CT scan. Increased awareness and training among staff probably led to increased diagnoses of acute cerebrovascular accidents in our stroke center and may help to further reduce target times.

**Table:** **Table 1** **(abstract P105)** Patient demographics and treatment per year

	**2017 (n = 241)**	**2018 (n = 274)**	**2019 (n = 305)**	**2020 (n = 435)**	**p value**
Age, years, median (IQR)	78 (68 – 84)	74 (63 – 83)	76 (66 – 83)	74 (64 – 82)	0.03
NIHSS, median (IQR) (n)	6 (3 – 15) (227)	6 (3 – 14) (269)	3 (1 – 10) (305)	3 (1 – 7) (434)	< 0.0001
Onset to door time, hours, median (IQR) (n)	1.87 (0.89 – 3.66) (164)	1.62 (0.94 – 3.79) (185)	2.93 (1.13 – 9.82) (270)	7.55 (1.63 – 19.16) (422)	< 0.0001
Door to CT time, minutes, median (IQR) (n)	27 (16 – 52) (235)	34 (20 – 71) (250)	39 (22 – 63) (289)	37 (21 – 68) (371)	0.0005
Door to needle time, minutes, median (IQR) (n)	35 (27 – 58) (31)	47 (34 – 70) (61)	48 (36 – 69) (51)	29 (20 – 49) (75)	< 0.0001
Door to groin time, minutes, median (IQR) (n)	70 (39 – 102) (54)	72 (53 – 94) (51)	82 (47 – 104) (46)	81 (52 – 106) (55)	0.54
Proportion conservative treatment, n (%)	144 (60)	168 (61)	219 (72)	328 (75)	0.0001

## P106

### Outcome predictors of acute stroke patients admitted to intensive care unit

#### P. Aries^1^, P. Bailly^2^, T. Baudic^2^, M. Consigny^2^, S. Timsit^2^, O. Huet^2^

##### ^1^Departement of Anaesthesia and Surgical Intensive Care, CHRU Brest, Brest, France; ^2^CHRU Brest, Brest, France

*Critical Care* 2021, **25**(**Suppl 1**): P106

**Introduction**: An increasing number of acute stroke patients are being admitted to an ICU. Those patients have high hospital mortality and poor functional outcomes [1]. However, limited data regarding the impact of organ support therapies are available. Moreover little is known about the use and the impact of withholding life sustaining treatment (LST).

**Methods**: We used the Brest Stroke Registry, a population-based prospective registry (INSERM) to identify all stroke patients (ischemic and hemorrhagic) admitted to an ICU in between 2008 and 2017 with at least one organ support therapy. This registry regroups all confirmed cases of stroke over the region of Brest city [2]. We retrospectively collected specific data from ICU stay. ICU mortality, 90-days mortality and functional status at hospital discharge were analyzed.

**Results**: A total of 215 patients were included, 61.4% were men, mean age was 65.66 ± 12.39 years. Ischemic stroke was diagnosed in 109/215 (50.1%); hemorrhagic stroke occurred in 106/215 (49.3%). Mean NIHSS score at admission was 22.53 ± 14.74, and mean GCS score was 9.39 ± 4.78. 112/215 (52%) patients died during ICU stay, 120/199 (60%) patients died before 90-days. A total of 158 out of 178 (88%) had poor outcomes (modified Rankin Score 4–6) at hospital discharge. Seventy-nine (36.9%) patients had care limitations. Age, mechanical ventilation at admission, and intracranial hypertension were independently associated with hospital mortality and 90-day mortality. Need of vasopressors was associated with hospital mortality. NIHSS score at admission and mechanical ventilation were independent predictors of poor functional outcome. Adjusting on decision to withhold LST did not modify these associations.

**Conclusions**: Our study showed that survival and functional outcomes remain poor in stroke patients admitted in ICU. Both neurological severity and organ support therapy were independent predictors of poor outcomes.

**References**

1. de Montmollin E et al. Ann Intensive Care 10:53, 2020
2. Timsit S et al. Neuroepidemiology 42:186–95, 2014

## P107

### In patients post-endovascular coiling for subarachnoid hemorrhage an increase in systolic arterial pressure is associated with increased risk of symptomatic cerebral vasospasm

#### A. B. Binabdi^1^, S. A. Al Khalaf^2^, H. A. Alzayer^3^, I. H. Hayes^4^

##### ^1^School of Medicine, University College Cork, Cork, Ireland; ^2^School of Public Health,University College Cork, Cork, Ireland; ^3^Department of Renal Medicine,Cork University Hospital, Cork, Ireland; ^4^Department of Anaesthesia and Intensive Therapy Unit, Cork University Hospital, Cork, Ireland

*Critical Care* 2021, **25**(**Suppl 1**): P107

**Introduction**: Cerebral vasospasm is the classic cause of neurological deterioration after aneurysmal subarachnoid hemorrhage (SAH) and contributes to morbidity and mortality. Predicting the onset of cerebral vasospasm remains a challenge. Recent research suggests an association between vasospasm and arterial hypertension, thus rendering changes in blood pressure (BP) a candidate for predicting increased risk of cerebral vasospasm. We investigated for a correlation between arterial hypertension following SAH and the onset of symptomatic cerebral vasospasm.

**Methods**: This was a retrospective cohort study of coiled aneurysmal SAH patients between January 2017 and June 2020 in Cork University Hospital. Post-coiling 4-hourly BP readings were collected up to day 9 or discharge. Symptomatic cerebral vasospasm was defined as a focal neurologic deficit or decrease of > 1 point on the Glasgow Coma Scale lasting > 1 h, non-attributable to other causes. Logistic regression analysis was performed for all the BP readings with adjustment for age, sex, presence of hydrocephalus and history of hypertension.

**Results**: A total of 56 patients were included in the analysis, 21 developed vasospasm (37.5%). Mean day of vasospasm onset was day 6 (± 2) post-coiling. A 10 mmHg increase in systolic BP was associated with an increased risk of cerebral vasospasm [Odds Ratio (OR) = 1.63; 95% confidence interval (CI) 1.04–2.6; p value < 0.05] (Fig. 1). The association was stronger in patients with pre-existing hypertension [OR = 2.29; 95% CI 1.1–4.77; p value < 0.05]. Cubic spline analysis showed a positive linear relationship between systolic BP and predicting the risk of vasospasm. No significance was found in diastolic or mean arterial pressure.

**Conclusions**: An increase in systolic BP post-coiling for SAH correlated with increased risk for cerebral vasospasm. Systolic BP may be of use in anticipating patients at increased risk for vasospasm, thereby prompting closer monitoring and earlier therapeutic intervention.

**Figure:**
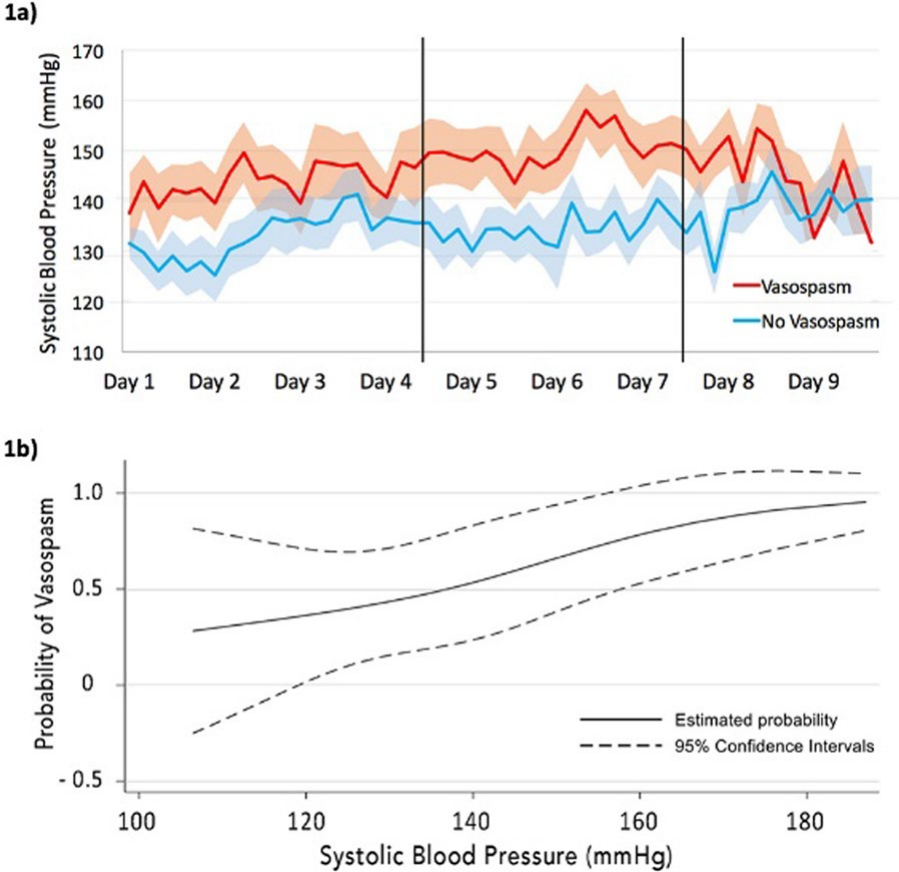
**Fig. 1** **(abstract P107)** **a** Systolic blood pressure readings across study period in vasospasm (red) versus non-vasospasm (blue) patients with line shading representing 95% confidence interval. Pronounced significant difference seen in days 5 to 7. **b**. Cubic spline graph with 3 knots representing the adjusted probability of vasospasm at different systolic blood pressure values. This model suggests a linear relationship between the probability of vasospasm and systolic blood pressure

## P108

### Concentration of the 4-hydroxyphenyllactic acid in the cerebrospinal fluid as a possible predictor of the bacterial meningitis

#### N. Beloborodova^1^, A. Pautova^1^, E. Chernevskaya^1^, I. Alexandrova^2^

##### ^1^Federal Research and Clinical Center of Intensive Care Medicine and Rehabilitology, Moscow, Russian Federation; ^2^National Medical Research Center for Neurosurgery named after Academician N.N. Burdenko, Moscow, Russian Federation

*Critical Care* 2021, **25**(**Suppl 1**): P108

**Introduction**: Several phenyl-containing acids which are metabolites of tyrosine and phenylalanine have been found in the cerebrospinal fluid (CSF) of neurosurgical patients with various intracranial diseases or injuries [1]. The goal of this study was to determine if the content of one of these acids (4-hydroxyphenyllactic acid, p-HPhLA) was specific for the neurosurgical patients with the suspected bacterial meningitis.

**Methods**: The residues of CSF samples from neurosurgical patients (n = 84) were obtained after diagnostic lumbar puncture, including patients with the suspected bacterial meningitis (n = 9). Concentration of p-HPhLA was measured by gas chromatography–mass spectrometry [1]. Nonparametric the Mann–Whitney U-test and ROC analysis with the AUROC parameter (area under the ROC curve) was used.

**Results**: The median values (interquartile range 25–75%) of the p-HPhLA for the patients without and with the suspected bacterial meningitis were 0.5 [0.3–1.1] and 1.1 [0.7–1.9] µmol/l, respectively. The concentration of the p-HPhLA was statistically higher in patients with the suspected bacterial meningitis (p = 0.025). The results of ROC analysis: AUROC – 0.730; standard error – 0.085, p = 0.025, two-sided asymptotic confidence interval (95%) – 0.563–0.898. The cut-off value was 0.7 µmol/l. Sensitivity – 77.8% (95% CI: 44.3%; 94.7%), specificity – 62.7% (95% CI: 51.3%; 72.8%). The patients with the concentration of the p-HPhLA equal or higher than 0.7 µmol/l had 5 times higher risk of the developing meningitis (unadjusted RR: 4.900 [95% CI: 1.082; 22.191]).

**Conclusions**: The results of statistical analysis indicate that the concentration of the p-HPhLA can be a one-parameter criterion for the diagnosis of the bacterial meningitis in neurosurgical patients.

**Acknowledgements**: Supported by the grant of President of the Russian Federation [No. MK-627.2020.7].

**Reference**

1. Pautova A et al. Biomedical Chromotraphy. 35:e4969, 2021.

**Figure:**
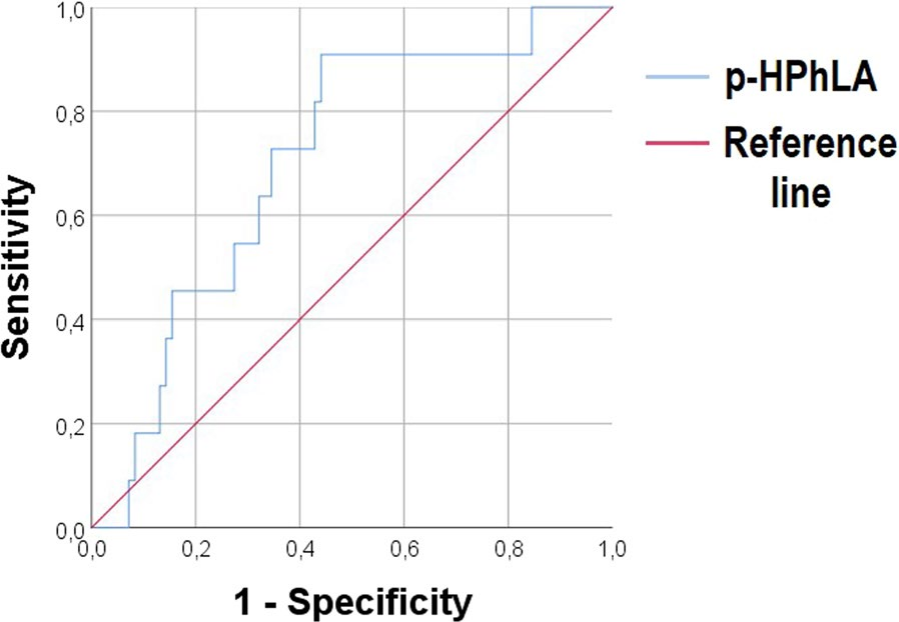
**Fig. 1** **(abstract P108)** The ROC-curve of the p-HPhLA as the predictor of the bacterial meningitis developing

## P109

### Prolonged mechanical ventilation in subarachnoid hemorrhage - the RAISE score

#### B. A. Ianosi^1,2^, V. Rass^2^, M. Lindlbauer^2^, A. Lindner^2^, M. Kofler^2^, A. Schiefecker^2^, B. Pfausler^2^, R. Beer^2^, R. Helbok^2^

##### ^1^Asklepios Fachklinikum Lübben, Neurology, Lübben (Spreewald), Germany; ^2^Department of Neurology, Medical University of Innsbruck, Innsbruck, Austria

*Critical Care* 2021, **25**(**Suppl 1**): P109

**Introduction**: Mechanical ventilation (MV) is frequently required for patients suffering from spontaneous subarachnoid hemorrhage (SAH). In a prospective cohort study analyzing retrospectively 297 consecutive non-traumatic SAH patients admitted to the ICU in a tertiary academic medical center, we aimed to identify factors associated with prolonged MV and to create a predictive score for prolonged MV.

**Methods**: Using multivariable generalized linear models, we identified factors associated with MV > 48 h, > 7 days, and > 14 days. Patients who were mechanically ventilated but died before 48 h, 7 days or 14 days and those never ventilated were excluded from the analysis. We incorporated those factors into a new prognostic score (the RAISE score: pRolonged ventilAtion In Subarachnoid hEmorrhage patients) to predict prolonged MV > 7 days. The score was developed by arbitrarily choosing 60% SAH patients in a training dataset which was further internally validated.

**Results**: The median age of patients was 57 (IQR 47–68) years and median admission Hunt&Hess grade (H&H) was 3 (IQR 1–5). The median duration of MV was 9 (IQR 2–20) days in 242 (82%) patients who required MV. Associations were found between a higher Acute Physiology Score (APS) and MV > 48 h, > 7 days and > 14 days, as well as between a higher H&H and MV > 7 days and > 14 days. Early neuroimaging findings (hydrocephalus; high-grade SEBES, Subarachnoid Hemorrhage Early Brain Edema Score) were associated with MV > 48 h. High-grade SEBES and co-occurrence of intraparenchymal bleeding were associated with MV > 7 days. Neuroimaging was, however, not associated with MV > 14 days. The RAISE score included age, APS, H&H, SEBES, and the presence of ICH stratifying the risk of MV > 7 days (See Fig. 1).

**Conclusions**: Disease severity and neuroimaging findings detected within 24 h of ICU admission are associated with the need for prolonged MV in patients with SAH. These results may be helpful to better anticipate the course of therapy.

**Figure:**
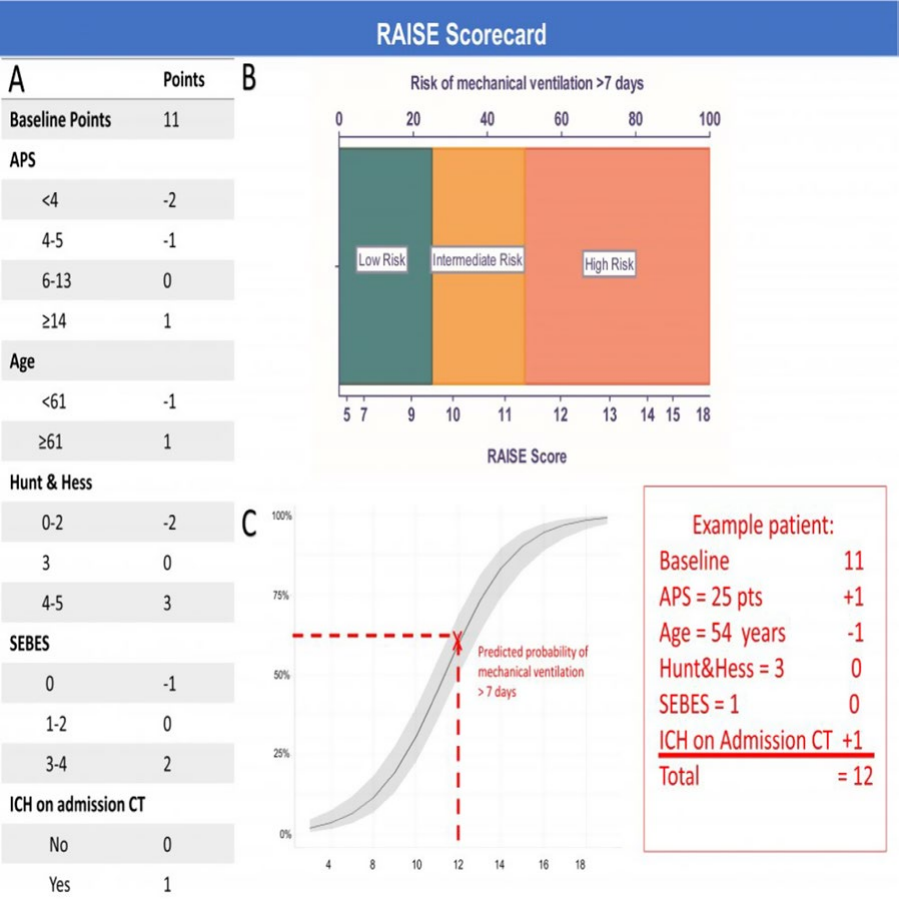
**Fig. 1** **(abstract P109)** The scorecard of the RAISE score (pRolonged ventilAtion In Subarachnoid hEmorrhage patients) shows (A) the assignment of points to each patient, (B) risk (%) of MV > 7 days at each assigned point sum, and (C) the curve showing the predicted probability of mechanical ventilation > 7 days based on assigned points with an example patient. APS - Acute Physiology Score, SEBES - Subarachnoid Hemorrhage Early Brain Edema Score

## P110

### The effect of the volemic and cardiac status on brain oxygenation in SAH patients

#### V. Rass^1^, E. Bogossian^2^, B. A. Ianosi^3^, L. Peluso^2^, A. Lindner^3^, A. Schiefecker^3^, B. Pfausler^3^, F. S. Taccone^2^, R. Helbok^3^

##### ^1^Neurology, Neurological Intensive Care Unit, Medical University of Innsbruck, Innsbruck, Austria; ^2^Department of Intensive Care, Erasme Hospital, Université Libre de Bruxelles, Brussels, Belgium; ^3^Neurology, Medical University of Innsbruck, Innsbruck, Austria

*Critical Care* 2021, **25**(**Suppl 1**): P110

**Introduction**: Optimal fluid management in patients after subarachnoid hemorrhage (SAH) aims at optimization of cerebral flood flow and brain oxygenation. Here, we investigated the effects of fluid management on brain oxygenation by integrating advanced hemodynamic monitoring (PiCCO) and invasive neuromonitoring.

**Methods**: This observational cohort bi-center study included data of consecutive poor-grade SAH patients, who underwent PiCCO monitoring and invasive neuromonitoring. Fluid management was guided by the transpulmonary thermodilution system and aimed at euvolemia (cardiac index, CI ≥ 3.0 l/min/m^2^; global end-diastolic index, GEDI 680–800 ml/m^2^; stroke volume variation, SVV < 10%). Patients were managed using a PbtO_2_ targeted protocol to prevent brain tissue hypoxia (BTH, PbtO_2_ < 20 mmHg). To assess the association between CI and PbtO_2_ and the effect of fluid challenges on CI and PbtO_2_ we used generalized estimating equations.

**Results**: On a total of 60 included patients (median age 56 [IQRs 47–65] years), BTH occurred in 23% of the monitoring time during the first 10 days since admission. Overall, mean CI was within normal ranges (ranging from 3.1 ± 1.3 l/min/m^2^ on day 0 to 4.1 ± 1.1 l/min/m^2^ on day 4). Higher CI values were associated with higher PbtO_2_ levels (Wald = 14.2; p < 0.001). Neither daily fluid input nor fluid balance were associated with absolute PbtO_2_ levels (p = 0.94 and p = 0.85, resp.) or the occurrence of BTH (p = 0.68 and p = 0.71, resp). PbtO_2_ levels were similar during hypovolemia and euvolemia. PbtO_2_ increased as a response to fluid boluses only if BTH was present at baseline (from 13 ± 6 to 16 ± 11 mmHg, adjusted OR = 15.2 [95% CI 2.3–99.6]; p = 0.005), but not in all interventions (p = 0.89).

**Conclusions**: In this study, a moderate association between increased cardiac output and brain oxygenation was observed. Fluid challenges may improve PbtO_2_ only in the presence of BTH at baseline. Individualized hemodynamic management requires advanced cardiac and brain monitoring in critically ill SAH patients.

**Figure:**
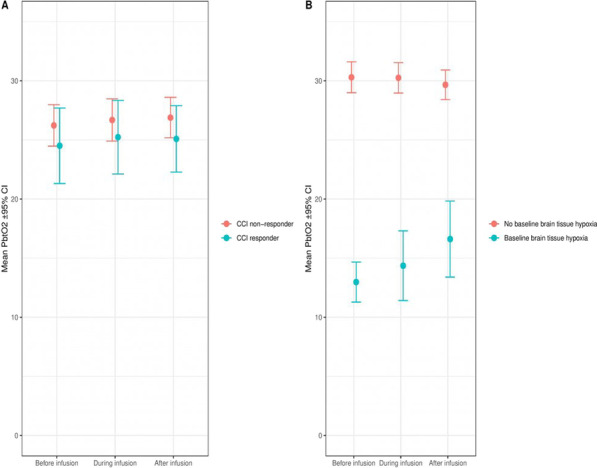
**Fig. 1** **(abstract P110)** (A) PbtO_2_ did not change (p = 0.89) as a response to fluid boluses both in CI responders and non-responders (p = 0.92, p = 1.00). (B) PbtO_2_ increased as a response to fluid boluses in the setting of BTH at baseline (p = 0.005)

## P111

### Long-term cognition and neuroinflammation in patients with coronavirus disease (COVID-19)

#### H. B. Duindam^1^, D. Mengel^2^, B. Van den Borst^3^, M. Kox^1^, M. Synofzik^2^, P. Pickkers^1^, W. Abdo^1^

##### ^1^Department of Intensive Care Medicine, RadboudUMC, Nijmegen, Netherlands; ^2^Department of Neurodegenerative Diseases, Center for Neurology and Hertie Institute for Clinical Brain Research, Tübingen, Germany; ^3^Department of Pulmonary diseases, RadboudUMC, Nijmegen, Netherlands

*Critical Care* 2021, **25**(**Suppl 1**): P111

**Introduction**: Cognitive complaints occur in a high percentage of patients suffering from long-COVID and can have lasting negative impact on rehabilitation, daily functioning and quality of life. COVID-19-induced neuroinflammation may account for this: innate immune cells of the brain become activated, due to systemic inflammation and a compromised blood–brain barrier, causing a neuroinflammatory state of the brain resulting in neuronal damage.

**Methods**: This prospective single center observational study serially assessed plasma inflammation and brain damage markers during hospital admission in both ICU and ward patients. Samples were collected every 5–7 days during hospital stay and analyzed using Simoa Neurology 4-plex for neuronal/neuroinflammatory biomarkers and Luminex Milliplex assay for cytokine determination. After six to eight months, included patients were asked to join additional cognitive examination, consisting of the Trail Making Test A and B, Letter Digit Substitution Test, Montreal Cognitive Assessment. Cognitive test results were compared to available normative data, adjusted for age and level of education.

**Results**: We included 123 patients with COVID-19 for plasma sampling and performed follow-up cognitive testing in 48 patients. Clinically significant cognitive impairment was measured in 38% to 50% of patients in at least one cognitive domain, depending on the applied cut-off value, with executive function most often affected. Momentarily, the plasma samples are being analyzed and we expect our results within the next few weeks.

**Conclusions**: This study is the first to correlate serial plasma inflammatory and neurological damage markers to long-term cognition in COVID-19 patients. The findings will demonstrate whether or not the plasma inflammatory and neurological damage markers exert accuracy to predict post-COVID-19 cognitive dysfunction, thereby possibly facilitating development of future (therapeutic or preventative) interventions.

## P112

### Dose the D-dimer test have a role in the diagnosis of cerebral vein thrombosis? A systematic review

#### A. A. Kassem^1^, T. S. Kumar^2^, M. A. Seaif^1^

##### ^1^Emergency Medicine, Hamad Medical Corporation, Doha, Qatar; ^2^Hamad Medical Corporation, Doha, Qatar

*Critical Care* 2021, **25**(**Suppl 1**): P112

**Introduction**: The D-dimer test has a diagnostic role in pulmonary embolism (PE) and deep vein thrombosis (DVT). In low-risk patients with negative D-dimer, PE, or DVT can be safely ruled out. We aim to know whether the D-dimer has a similar role in cerebral vein thrombosis (CVT) diagnosis.

**Methods**: A literature review in PubMed, Cochrane, Google scholar, from 1996 to July 2021. The AMASTER tool used for studies quality assessment.

**Results**: Out of 76 relevant titles and abstracts, two systematic reviews & meta-analyses, and one cohort study related to our clinical question [1–3]. The studies are tabled with details of author, publication date, population details, and results as in the table. Most of the studies show D–dimer has a high sensitivity, around 93 to 97%, in CVT diagnosis. It was found that more CVT extension and earlier presentation (< 2 weeks) were correlated with higher D-dimer levels. Unfortunately, most of the studies are not high-quality studies, with variable designs, population, and reference standard tests. The studies showed that D dimer could help predict CVT in combination with risk factors and clinical presentation.

**Conclusions**: Normal D-dimer only should not be used to exclude CVT. There is a probability of using D-dimer in CVT risk scoring and pre-imaging negotiation, and for that purpose, larger and higher-quality studies are needed.

**References**

1. Dentali F et al. J Thromb Haemost 10:582–9, 2012
2. Alons IM et al. BMC Neurol 15:118, 2015
3. Thammishetti V et al. J Clin Diagn Res 10:OC07-10, 2016

**Table:** **Table 1** **(abstract P112)** Summary of relevant studies

Study	Study type	Patient group	Outcomes	Weakness/comments
Dentali et al., Italy, 2012 (1)	MA	1134 suspected/diagnosed CVT, CT or MRV/D-dimer	Sensitivity 93.8%, & specificity 89.7%	low quality studies included, variable designs
Alons et al., Netherlands 2015 (2)	MA	636 isolated headache, CT or MRV/D-dimer	Sensitivity: 97.8%. Specificity: 84.9%	High risk group was excluded
Thammishetti V. India, 2016 (3)	Prospective Cohort Study	80 CVT/non CVT patient, CT or MRV/D-dimer	Sensitivity 80.62% & specificity 80%	Small control groups, low quality D-dimer assay

## P113

Withdrawn

## P114

### Treatment of upper limb spasticity in patients with chronic disorders of consciousness using continuous brachial plexus block. Case series

#### N. Lesteva^1^, D. Vasilyev^2^, E. Kondratyeva^2^, S. Kondratyev^2^, A. Kondratyev^2^

##### ^1^Anesthesiology and Intensive Care, Almazov National Medical Research Centre, Sankt-Petersburg, Russian Federation; ^2^Almazov National Medical Research Centre, Sankt-Petersburg, Russian Federation

*Critical Care* 2021, **25**(**Suppl 1**): P114

**Introduction**: Spasticity in disorders of consciousness (DOC) is mainly severe, and causes increasing of nociception, impairs physical therapy and care as well as rehabilitation prognosis in general [1]. It is especially likely to happen in patients who suffer spasticity in the upper limbs. Managing spasticity could positively impact neurological status. A regional blockade of the upper limb causes anesthesia and muscular relaxation in relevant zones. That potentially can be beneficial during spasticity treatment, especially physical therapy, which can cause pain and increasing of muscular hypertonicity itself.

**Methods**: Four patients with chronic DOC after local ethics committee approval received treatment of spasticity added by continuous brachial plexus block combined with the upper limb periodical immobilization via plaster splint in as far as possible extended position. All the patients had been assessed initially, then during treatment and at the discharge with Coma Recovery Scale-Revised (CRS-R), Modified Ashworth Scale (MAS), Nociception Coma Scale-Revised (NCS-R), as well as passive range of movements in elbow joints were measured.

**Results**: There was among all the patients increased passive range of movements in elbow joints (10-40o), decreased intensity of spasticity (2 MAS comparing with 3–4 before treatment), nobody had pain level above 1 NCSR during physical therapy (initially they had 3–4). One patient’s neurological status improved after treatment (assessed using CRS-R). There were no complications.

**Conclusions**: Method can be beneficial while treating spasticity in DOC. Further studies are needed.

**Reference**

1. Thibaut A et al. Brain Injury 27:1093–105, 2013

## P115

### Correlation between changes in sleep structure according to polysomnography, melatonin levels, MRI and FDG PET in patients with chronic disorders of consciousness

#### E. Kondratyeva^1^, L. Korostovceva^2^, M. Frolova^3^, E. Potemkina^4^, D. Ryzkova^4^, S. Kondratyev^5^

##### ^1^Minimally Conscious Research Group, Almazov National Medical Research Centre, St Petersburg, Russian Federation; ^2^Somnology Research Group, Almazov National Medical Research Centre, St Petersburg, Russian Federation; ^3^Laboratory, The Nikiforov Russian Centre of Emergency and Radiation Medicine, Saint-Petersburg, Russian Federation; ^4^Almazov National Medical Research Centre, St Petersburg, Russian Federation; ^5^Intensive Care, Almazov National Medical Research Centre, St Petersburg, Russian Federation

*Critical Care* 2021, **25**(**1**): P115

**Introduction**: Disoders of consciousness (DOC) patients often show severe sleep fragmentation, which is likely caused by structural changes in the brain areas responsible for sleep maintenance.

**Methods**: The study was based on the results of examination and treatment of 43 DOC patients. The average duration of DOC - 4.4 ± 0.7 months. The 1 group - patients with a total score CRS-R of 0–5 points, 2 group of 5–8, and 3 group - > 9. All patients underwent polysomnography (PSG) for 2 days, melatonin level was measured 6 times a day: at 8 am, 15 pm, 18 pm, 21 pm, 24 pm and 3 am, daily urine collection to determine 6-sulfatoxymelatonin in the daily and night portions of urine. MRI (3 Tesla) with a detailed assessment of structural changes in the brainstem, hypothalamus and thalamus according to the "MRI atlas of the human hypothalamus" by M. Baroncini. Also, PSG data were compared with PET CT of the brain.

**Results**: 41.7% of patients of group 1, there were no sleep cycles. The NREM/REM ratio in patients of the 1 group was almost twice as high as in patients of the 2 and 3 groups. In patients of 3 group total sleep time was higher, and sleep cycles were recorded in 58.3% of patients, the average value of the N2/total sleep time ratio was higher, but the average value of the N1/total sleep time ratio in patients of the third group was lower than in other groups. Melatonin level was maintained in patients of all groups. Relationship of PSG changes in the FDG PET data was not found. When analyzing macroscopic changes of the structure morphology (thalamus, hypothalamus, midbrain, corpus callosum), relationship with the assignment to the group and the PSG data was not identified.

**Conclusions**: There are no clearly defined PSG patterns that reliably define each of the reviewed DOC states. We have received evidence indicating that sleep in DOC is under normal homeostatic control – level and rhythm of melatonin secretion were preserved in all groups.

**Acknowledgement**: Study was supported by RFBR grant 19–29-01–066.

## P116

### Exposure to non-lung protective mechanical ventilation is associated with outcome in critically ill patients with acute brain injury

#### N. Sangana^1^, H. Kim^2^, R. Stevens^2^

##### ^1^Department of Mechanical Engineering, Johns Hopkins University, Baltimore, USA; ^2^Department of Anesthesiology and Critical Care Medicine, Johns Hopkins School of Medicine, Baltimore, USA

*Critical Care* 2021, **25**(**Suppl 1**): P116

**Introduction**: Patients with acute brain injury (ABI) commonly receive mechanical ventilation (MV) which can be harmful due to lung injury and adverse physiological effects on the brain. Our aim was to explore relationships between MV and outcome of patients with ABI. We hypothesize that MV variables are important determinants of short-term ABI outcomes.

**Methods**: Adult patients with traumatic brain injury (TBI) or stroke who received MV as part of their care in the ICU were selected from large multicenter database (eICU). A set of features was crafted based on current recommendations for lung-protective MV and included time “out-of-range” (OOR) for: tidal volume per ideal body weight (> 8 ml/kg), plateau pressure (> 30 cmH_2_O), PEEP (< 5 cmH_2_O). Outcomes were defined as “Unfavorable” for patients who died or whose discharge motor Glasgow Coma Score (mGCS) was < 5, and “Favorable” for patients who were alive and had a mGCS ≥ 5 at discharge. Two models were created: (1) A clinical model which uses only the first value recorded for each feature; and (2) A combined model which included predefined OOR MV variables.

**Results**: Data were analyzed on 839 and 1,221 ICU stays for TBI and stroke, respectively. Clinical and combined models had an AUROC of 0.67 ± 0.07 and 0.79 ± 0.13 for Stroke and an AUROC of 0.70 ± 0.07 and 0.81 ± 0.07 respectively for TBI. A number of OOR MV variables and respiratory physiologic variables were found to be significant (Fig. 1). Longer durations of exposure to OOR tidal volume, PEEP and plateau pressure were identified as top contributors with longer OOR durations predictive of unfavorable outcome.

**Conclusions**: These results indicate that exposure to non-lung protective MV may contribute to clinical and neurological outcomes in stroke and TBI patients. They support the need for clinical trials evaluating optimal MV strategies in this population.

**Figure:**
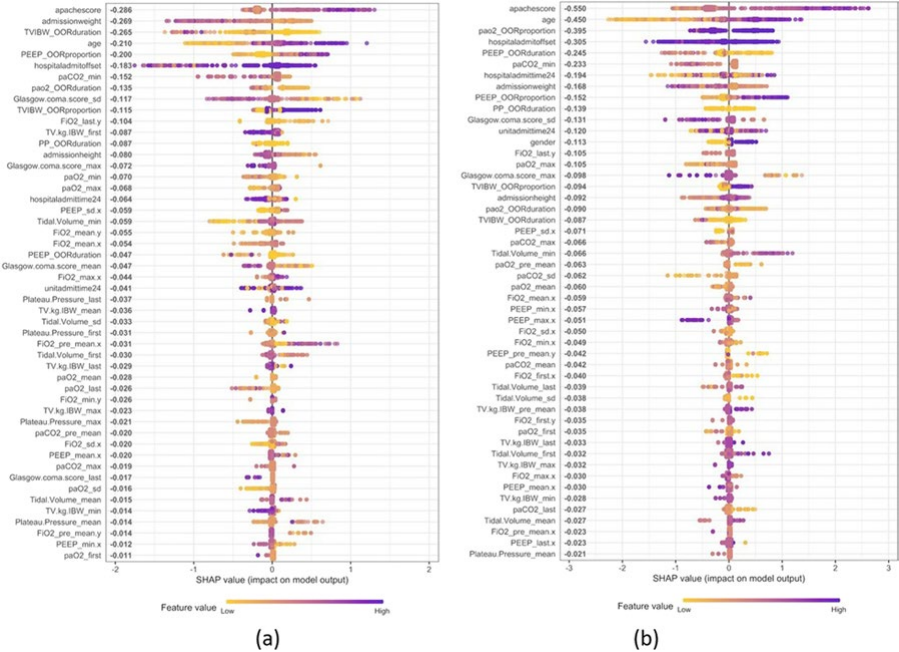
**Fig. 1** **(abstract P116)** Feature ranking of top 50 features for TBI combined model (left) and stoke combined model (right). Negative SHAP value association with favorable outcomes and positive SHAP associated with unfavorable outcomes

## P117

### Timing of invasive intracranial pressure monitoring between neurosurgeons and intensive care physicians (TIMING-ICP)

#### L. Mariani^1^, N. Latronico^2^, D. Filippi^3^, G. Chiarini^3^, F. A. Rasulo^2^

##### ^1^Department of Clinical and Experimental Sciences, University of Brescia, Brescia, Italy; ^2^Department of Medical and Surgical Specialties, Radiological Sciences and Public Health, University of Brescia, Brescia, Italy; ^3^University Division of Anesthesiology and Critical Care Medicine, ASST Spedali Civili, Brescia, Italy

*Critical Care* 2021, **25**(**Suppl 1**): P117

**Introduction**: Prolonged intracranial hypertension (ICH) has been related to poor outcome [1]. Placement of an intracerebral catheter represents the gold standard technique for intracranial pressure (ICP) invasive monitoring. This maneuver has usually been performed by neurosurgeons, but recently this procedure has more often been carried out by intensivists, at the bedside [2]. Preliminary retrospective data suggest that ICP catheter placement performed by trained intensivists can be a safe procedure which can be carried out faster than the one performed by neurosurgeons, with a similar incidence of complications. The aim of this observational, prospective and multicentric study is to compare timing of invasive ICP monitoring performed by intensivist and neurosurgeons and to detect differences in the incidence of complications.

**Methods**: We are including all adults with acute cerebral pathology and urgent indication to invasive ICP monitoring in 16 different centers in Italy. Exclusion criteria are represented by significative coagulation disorders, not urgent request, need for intracranial catheter placement other than ICP monitoring. Timing of ICP probe positioning will be analyzed as follows: T0 represents time in which ICH development is suspected, T1 represents time when indication to ICP monitoring is stated and T2 is the time in which skin incision is performed. Incidence of catheter-related intracranial bleedings, meningitis related to procedure and wrong positioning of catheter and/or its malfunction will be also registered.

**Results**: This study was registered on ClinicalTrial.gov. Centers are enrolling patients.

**Conclusions**: Despite all efforts, intracranial hypertension is still a worldwide medical issue. TIMING-ICP may allow us to assess if ICP monitoring placement by intensivists can be a time-saving procedure compared to neurosurgical one and equally safe.

**References**

1. Vik A et al. J Neurosurg 109:678–84, 2008
2. Ehtisham A et al. Neurocrit Care 10:241–7, 2009

## P118

### A scoping review of interventions to reduce diagnostic blood loss, anemia and transfusion in hospitalized patients

#### T. Francois^1^, J. Charlier^1^, S. Balandier^1^, M. Tucci^1^, A. Pincivy^2^, J. Lacroix^1^, G. Du Pont-Thibodeau^1^

##### ^1^Pediatric Critical Care Unit, Department of Pediatrics, Centre Hospitalier Universitaire Sainte-Justine, Université de Montréal, Montreal, Canada; ^2^Scientific Library, Centre Hospitalier Universitaire Sainte-Justine, Université de Montréal, Montreal, Canada

*Critical Care* 2021, **25**(**Suppl 1**): P118

**Introduction**: Blood sampling is a recognized and modifiable contributor to iatrogenic anemia in pediatric critical care units (PICU).

**Methods**: We aimed to bundle and evaluate current evidence of interventions to lower the incidence of iatrogenic anemia and transfusion by lowering diagnostic blood loss in hospitalized patients. We performed a systematic search in PubMed, Ovid MEDLINE, All EBM Reviews (Cochrane) and Embase until May 2021. Trial eligibility, data extraction and Risk of Bias (ROB Cochrane Collaboration tool) assessment were independently done by 2 or 3 reviewers.

**Results**: Thirty-nine trials were retained after screening 16,132 papers. ROB was high in all studies, but one. Many studies evaluated multiple interventions, of which most done in adults. All 7 adult studies evaluating small blood tubes observed a significant reduction in blood loss, only 1 observed a positive effect on hemoglobin (Hb) / transfusion. 8 adult studies evaluated closed blood sampling: all showed a significant reduction in blood discarded with varying effects on Hb. 1 neonatal study showed that returning blood discarded was effective in lowering transfusion volume. Point-of-care testing was found effective in reducing transfusion in 1 adult study; 5 neonatal studies reported divergent effects. Another study showed adults being significantly sampled more with arterial line vs. without. The remaining 16 trials that implemented bundled interventions showed mixed results: only 3/10 adult studies observed a significant lower transfusion rate, the 6 pediatric trials observed a lower transfusion rate when bundles included education/protocols.

**Conclusions**: Current evidence on interventions to reduce diagnostic blood volume and associated complications is highly heterogeneous. Using smaller tubes and closed-loop sampling may be effective in adults, while bundled interventions with protocols/teaching seem promising in the pediatric population.

## P119

### Application of population pharmacokinetics of nadroparin for thromboprophylaxis in critically ill COVID-19 patients: accurate prediction of anti-Xa levels and assessment of dosing regimens

#### L. Romano^1^, N. Hunfeld^2^, M. Kruip^1^, H. Endeman^3^, T. Preijers^4^

##### ^1^Department of Hematology, Erasmus MC, Erasmus University Medical Center Rotterdam, Rotterdam, Netherlands; ^2^Department of Adult Intensive Care Medicine, Department of Hospital Pharmacy - Clinical Pharmacology, Erasmus MC, Erasmus University Medical Center Rotterdam, Rotterdam, Netherlands; ^3^Department of Adult Intensive Care Medicine, Erasmus MC, Erasmus University Medical Center Rotterdam, Rotterdam, Netherlands; ^4^Department of Hospital Pharmacy - Clinical Pharmacology, Erasmus MC, Erasmus University Medical Center Rotterdam, Rotterdam, Netherlands

*Critical Care* 2021, **25**(**Suppl 1**): P119

**Introduction**: COVID-19 ICU patients require intermediate dosing of low molecular weight heparin (LMWH) (e.g. nadroparin 5700 IE b.i.d.). However, the optimal dose regimen (reaching anti-Xa target levels) is unknown. Respecting stringent sample timing (t = 4 h) can also be challenging. This study aimed to assess which dose is adequate for critically ill COVID-19 patients and whether a population pharmacokinetic (PK) model can predict anti-Xa levels accurately irrespective of sample timing.

**Methods**: Retrospective data of 65 ICU patients with ≥ 1 positive SARS-CoV-2 PCR were included. At ICU admission, patients started with b.i.d. 5700 IU nadroparin. Using anti-Xa level vs. time, a population PK model was constructed with NONMEM v7.4.1. Monte Carlo simulations (10,000 patients) provided individual anti-Xa versus time curves to allow evaluation of the accuracy of sample timing with Bayesian analysis. Accuracy was described by the relative mean prediction error (rMPE) for bias (< 15%) and relative root mean squared error (rRMSE) for imprecision (< 25%).

**Results**: Anti-Xa levels versus time profiles were adequately described using a 2-compartment model with following covariates associated to clearance (CL): CRP, D-dimer, CKD-EPI-eGFR, corticosteroid and vasopressor use. Target level (0.3 IU - 0.7 IU/ml) achievement was most optimal for b.i.d. 5700 IU (Fig. 1). Using 1 sample, t = 3 h allowed to estimate the anti-Xa level (rMPE: 1.8%; rRMSE: 19.1%) and CL of nadroparin (rMPE: 1.9%; rRMSE: 22.4%) most optimally. Using 2 samples, a first t = 4 h and second after a subsequent dose between t = 2 h to t = 10 h allowed to adequately predict anti-Xa levels (range rMPE: 0.94%-3.36%; rRMSE: 15.1%-23.7%) and CL (range rMPE: 0.79%-1.49%; rRMSE: 16.6%-18.2%), respectively.

**Conclusions**: Anti-Xa levels were most optimal within target using an intermediate dose (b.i.d. 5700 IU). An evaluation of limited sampling strategies demonstrated the accuracy of less stringent sample timing.

**Figure:**
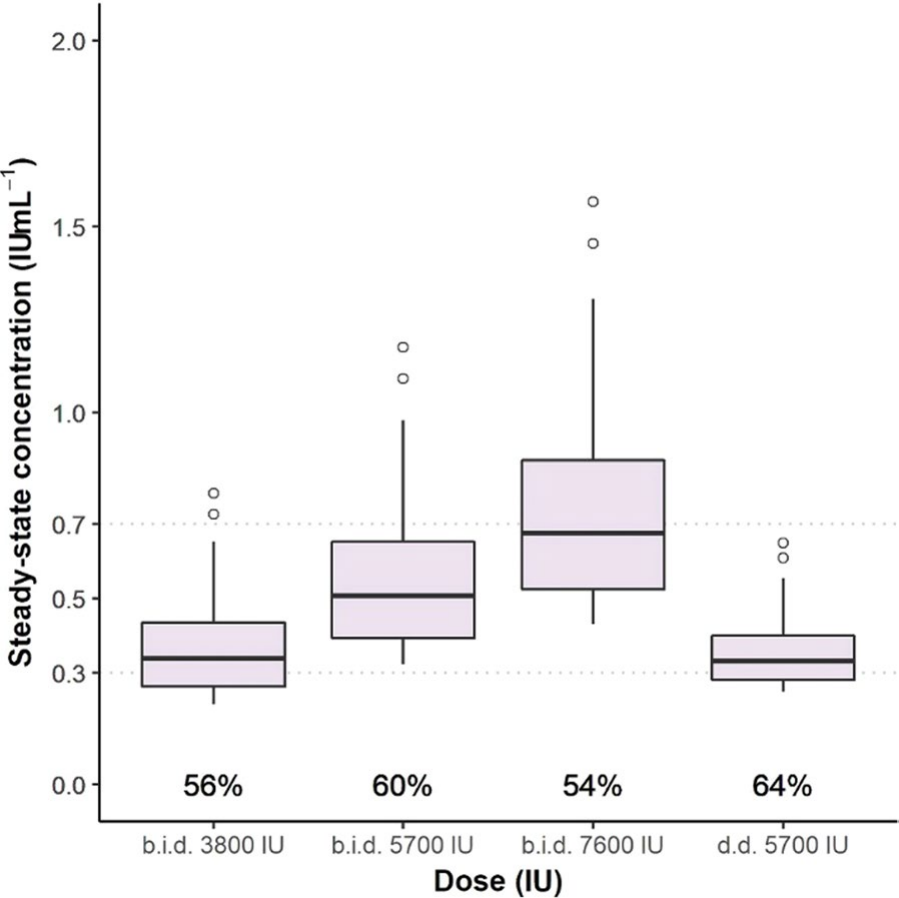
**Fig. 1** **(abstract P119)** Boxplots of estimated anti-Xa target levels at t = 4 h after respective nadroparin dose regimen in the modeling (n = 65) cohort. The amount of patients on target (anti-Xa 0.3 - 0.7 IU/ml) is specified with the percentage below the boxplot

## P120

### Biochemical quality of OctaplasLG frozen and freeze-dried products

#### A. Heger^1^, A. C. Hinz^2^, G. Gruber^3^

##### ^1^Research & Development, Octapharma PPGmbH, Vienna, Austria; ^2^Octapharma AB, Stockholm, Sweden; ^3^Octapharma PPGmbH, Vienna, Austria

*Critical Care* 2021, **25**(**Suppl 1**): P120

**Introduction**: OctaplasLG® is a solvent/detergent-treated, coagulation active plasma product for treating complex coagulation factor deficiencies, such as coagulopathy due to severe hepatic failure or massive transfusion, and as a substitution therapy in coagulation factor deficiencies in situations where specific factor concentrates are not available, or in emergency situations where precise laboratory diagnosis is not possible. OctaplasLG® is supplied frozen; the thawed product is stable during refrigerated storage for up to 5 days. Recently, a new freeze-dried form was developed (OctaplasLG® Lyo) to further increase ease of logistics and utilization.

**Methods**: Three OctaplasLG® Lyo batches for process performance qualification were manufactured at Octapharma AB, freeze-dried and reconstituted with sterilized water. Twelve batches of frozen OctaplasLG® were used for comparison; batches were assessed directly after thawing, as well as after storage of the thawed product at 2–8 °C for ≤ 6 days. All batches were assessed for global coagulation parameters, important coagulation factors and protease inhibitors, and activation markers of coagulation and fibrinolysis.

**Results**: Frozen OctaplasLG® and freeze-dried OctaplasLG® Lyo demonstrate identical quality profiles upon thawing and reconstitution, respectively; all parameters were in line with levels mandated by the European Pharmacopoeia. In addition, OctaplasLG® Lyo exhibited comparable/higher quality for temperature-sensitive parameters, such as levels of factor V, factor VIII, and protein S, compared to thawed OctaplasLG® after 6 days refrigerated storage (Fig. 1).

**Conclusions**: OctaplasLG® frozen and freeze-dried products have equally high biochemical quality. The key features of the new freeze-dried version (OctaplasLG® Lyo) are the fast reconstitution time and flexibility of storage conditions (refrigerated/room temperature), which has advantages in emergency situations and in ex-hospital settings.

**Figure:**
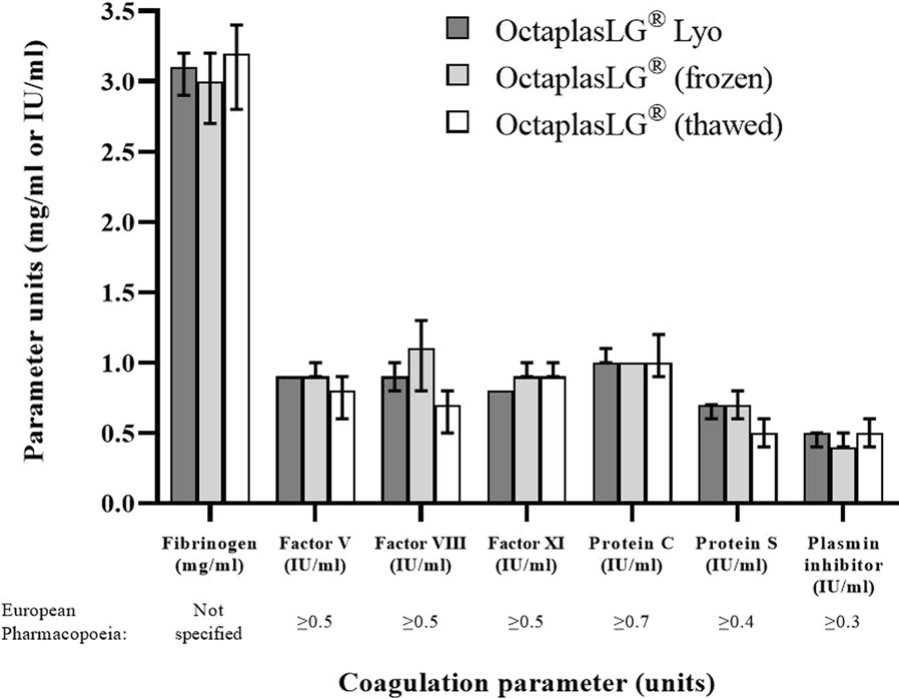
**Fig. 1** **(abstract P120)** Quality of OctaplasLG® and OctaplasLG® Lyo products

## P121

### The FiiRST-2 prospective, randomized study of clotting factor concentrates versus standard massive hemorrhage protocol in severely bleeding trauma patients

#### L. Da Luz^1^, J. Callum^2^, A. Beckett^3^, H. Peng^4^, P. Engels^5^, N. Parry^6^, H. Tien^7^, A. Nathens^7^, B. Schwartz^8^, K. Karkouti^9^

##### ^1^Department of Surgery, Sunnybrook Health Sciences Centre, Toronto, Canada; ^2^Kingston Health Sciences Centre, Kingston, Canada; ^3^Saint Michael´s Hospital, Toronto, Canada; ^4^Defence Research and Development Canada, Toronto, Canada; ^5^Hamilton General Hospital, Hamilton, Canada; ^6^London Health Sciences Centre, London, Canada; ^7^Sunnybrook Health Sciences Centre, Toronto, Canada; ^8^Octapharma, Paramus, USA; ^9^University Health Network, Sinai Health System, and Women’s College Hospital, Toronto, Canada

*Critical Care* 2021, **25**(**Suppl 1**): P121

**Introduction**: Bleeding plus acute trauma coagulopathy (ATC) is a leading cause of in-hospital mortality in trauma. Patients with ATC are up to 8 times more likely to die ≤ 24 h after injury than those without coagulopathy. Acquired fibrinogen deficiency and impaired thrombin generation are major drivers of ATC. Prompt and targeted coagulation factor replacement with fibrinogen concentrate (FC) and prothrombin complex concentrate (PCC) may be superior to current standard of care with ratio-based plasma resuscitation via a massive hemorrhage protocol (MHP). FiiRST-2 is investigating whether FC + PCC given ≤ 1 h after hospital arrival is superior to standard of care.

**Methods**: FiiRST-2 is a randomized, parallel-control, superiority trial with an adaptive two-stage design, performed in eight Canadian Level One Trauma Centers. Bleeding trauma patients > 16 years old (N = 350) receive FC + PCC or a minimum 2:1 red blood cells (RBCs):plasma transfusion plus platelets, until the second MHP pack has been given, MHP is terminated, or 24 h has elapsed from admission (Fig. 1). Exclusion criteria include receipt of > 2 units RBCs before randomization, > 3 h elapsed from injury, catastrophic brain injury, or known congenital or acquired bleeding disorders. The primary endpoint is superiority in the number of composite allogeneic blood product units transfused ≤ 24 h after admission. Secondary endpoints include RBC units transfused ≤ 24 h after admission, ventilator-free days, and 28 day mortality. Adverse and serious adverse events, including thromboembolic complications, will be assessed through 28 days.

**Results**: FiiRST-2 has enrolled 3 patients at 1 site and is expected to complete in Q1 2023.

**Conclusions**: This study could have a major impact on clinical practice and improve the management and outcomes of bleeding trauma patients. FiiRST-2 will determine if early use of factor concentrates (FC + PCC) is superior to standard of care (ratio-based plasma resuscitation) in these high-risk patients.

**Figure:**
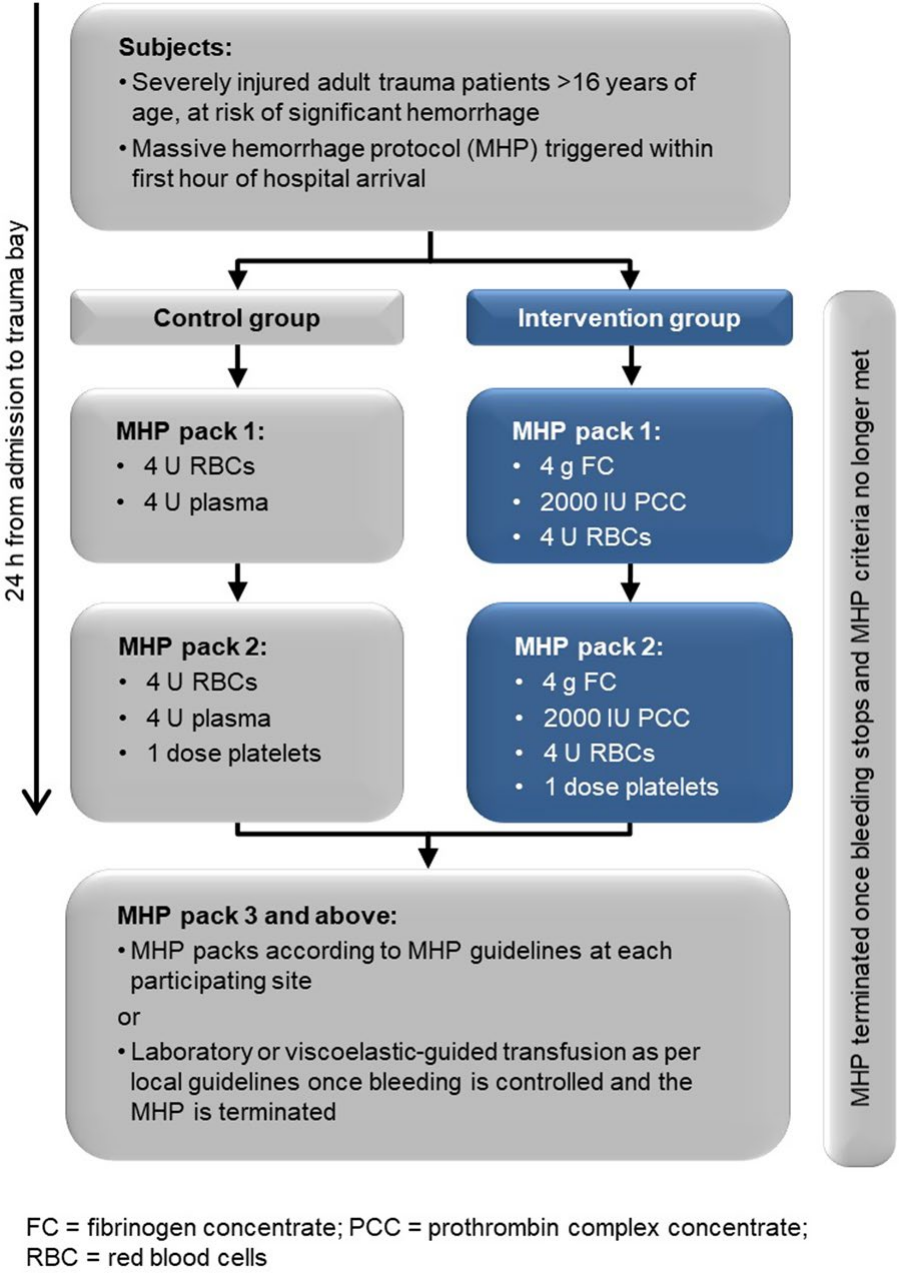
**Fig. 1** **(abstract P121)** Study treatment plan

## P122

### Sorption of sepsis-associated metabolites on the mesoporous carbon sorbent modified with 3-phenylpropanoic acid

#### N. Beloborodova^1^, M. Getsina^1^, L. P’yanova^2^

##### ^1^Lab. Metabolisms in Critical State, Federal Research and Clinical Center of Intensive Care Medicine and Rehabilitology, Moscow, Russian Federation; ^2^Center of New Chemical Technologies of the Federal Research Center Boreskov Institute of Catalysis of Siberian Branch of the Russian Academy of Sciences, Omsk, Russian Federation

*Critical Care* 2021, **25**(**Suppl 1**): P122

**Introduction**: We studied the mesoporous carbon sorbent VNIITU-2, modified with 3-phenylpropanoic acid (PhPA), because this metabolite of normal gut microbiota has a number of positive properties. Previously, we showed that PhPA is always present in blood of healthy humans, but is absent in patients with sepsis. The aim of the work is to evaluate the ability of the modified sorbent to perform targeted sorption of metabolites associated with sepsis and desorption of the modifier.

**Methods**: In an experiment in vitro, created model solutions in blood plasma with sepsis-associated metabolites such as 3-phenyllactic (PhLA), 2-(4-hydroxyphenyl)acetic (p-HPhAA), 3- (4-hydroxyphenyl)lactic (p-HPhLA) acids close to the known concentration of these metabolites in the blood serum of patients with A) renal failure, B) sepsis, C) septic shock (Table 1). The sorbent without a modifier and three types of sorbents with different content of the modifier PhPA were studied. A 1 ml aliquot of model solutions was added to each 1.5 ml Eppendorf tube with the sorbent for the sorption experiments, n = 3 for min, middle and max concentration. The weight of the sorbent was 0.053 ± 0.001 g. The mixture was shaken for 30 min, the supernatant was taken for analysis by liquid–liquid extraction and gas chromatography-mass spectrometry.

**Results**: The degrees of the sorption of all sepsis-associated metabolites for the minimum and middle concentrations of the model solutions were more than 90%. Only at the maximum concentration of PhLA, p-HPhAA, p-HPhLA in solution and the maximum concentration of the PhPA modifier on the sorbent, sorption decreased slightly to 50–80%. In all modified sorbents, there was an easy and fast desorption of PhPA from the surface of the sorbent into the solution.

**Conclusions**: The studied modified sorbent demonstrated in vitro ability to perform the function of purifying the blood from toxic sepsis-associated compounds, and simultaneously it can serve as a carrier for the delivery of missing metabolites to the body.

**Table:** **Table 1** **(abstract P122)** Concentrations of sepsis-associated metabolites in model solutions, µmol/l

Model solutions	Correspond to the pathological conditions (conc.)	PhLA	p-HPhAA	p-HPhLA
A	renal failure (min)	7	8	7
B	sepsis (middle)	22	24	20
C	septic shock (max)	72	79	66

## P123

### Impact of cefiderocol treatment on iron homeostasis and anemia in critically ill patients with carbapenem-resistant infections in the CREDIBLE-CR study

#### C. Longshaw^1^, G. Weiss^2^, E. P. Skaar^3^, M. Zeitlinger^4^, T. Baba^5^, Y. Matsunaga^6^, S. Portsmouth^6^, R. Echols^7^

##### ^1^Medical Affairs, Shionogi B.V., London, UK; ^2^Department of Internal Medicine II, Medical University, Innsbruck, Austria; ^3^Infectious Diseases, and Immunology, Department of Pathology, Vanderbilt University Medical Center, Nashville, TN, USA; ^4^Department of Clinical Pharmacology, Medical University, Vienna, Austria; ^5^Shionogi & Co., Ltd., Osaka, Japan; ^6^Shionogi Inc., Florham Park, NJ, USA; ^7^Infectious Disease Drug Development Consulting, LLC., Easton, CT, USA

*Critical Care* 2021, **25**(**Suppl 1**): P123

**Introduction**: We studied whether cefiderocol (CFDC) treatment had an adverse effect on iron homeostasis or led to anemia in critically ill patients enrolled into the open-label, pathogen-focused, Phase 3 CREDIBLE-CR study [1]. CFDC is an iron-chelator, siderophore cephalosporin developed for the treatment of infections caused by carbapenem-resistant (CR) Gram-negative bacteria, including Enterobacterales and non-fermenter species, in adult patients with limited treatment options.

**Methods**: Patients with serious CR infections were randomized 2:1 to receive intravenous CFDC, 2 g, q8h, or best available therapy (BAT; ≤ 3 antibiotics with Gram-negative activity) for 7–14 days [1]. Hemoglobin levels were measured in protocol-defined routine laboratory analyses. Specific iron homeostasis-related laboratory tests were performed at randomization and test of cure [TOC; end of treatment + 7 days) to determine serum levels of total iron, hepcidin, total iron binding capacity (TIBC) and transferrin saturation (TS%). Normal iron level ranges were defined as 59–178 µg/mL (males) and 37–173 µg/mL (females).

**Results**: Of 150 randomized patients (CFDC 101, BAT 49), 71 (47.3%) had anemia in their medical history (CFDC 44.6% [45/101], BAT 53.1% [26/49]) and 134 (CFDC 91, BAT 43) had serum iron data available at baseline. Most patients had iron levels below the lower limit of normal (CFDC 76.2% [77/101], BAT 57.1% [28/49]), and fewer patients had iron levels in the normal range (CFDC 13.9% [14/101], BAT 30.6% [15/49]). Between baseline and TOC, mean hemoglobin level increased from 9.6 to 9.9 g/dL in the CFDC arm and from 9.0 to 9.5 g/dL in the BAT arm (Fig. 1). Changes in all four specific iron homeostasis parameters between baseline and TOC were similar between treatment arms (Fig. 1).

**Conclusions**: CFDC therapy did not lead to increased anemia or reduced serum iron level in patients with serious CR infections.

**Reference**

1. Bassetti M et al. Lancet Infect Dis 21:226–240, 2021.

**Figure:**
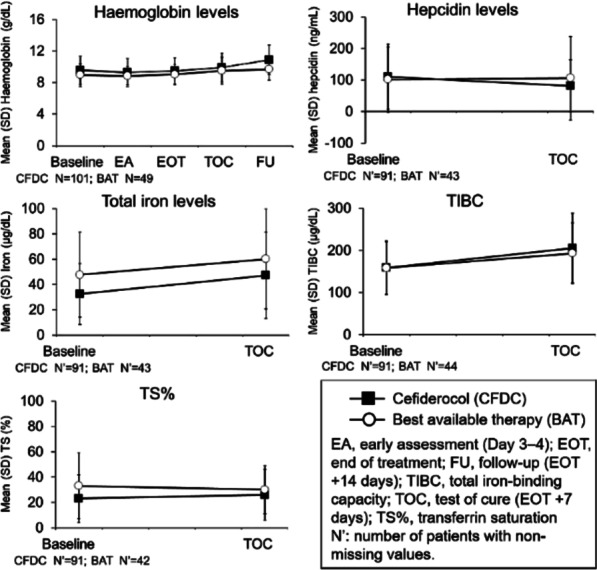
**Fig. 1** **(abstract P123)** Changes in iron homeostasis parameters in the CREDIBLE-CR study

## P124

### Effects of iron supplementation on the efficacy of cefiderocol in patients with carbapenem-resistant infections in the pathogen-focused CREDIBLE-CR study

#### C. Longshaw^1^, G. Weiss^2^, E. P. Skaar^3^, M. Zeitlinger^4^, T. Baba^5^, Y. Matsunaga^6^, S. Portsmouth^6^, R. Echols^7^

##### ^1^Medical Affairs, Shionogi B.V., London, UK; ^2^Department of Internal Medicine II, Medical University, Innsbruck, Austria; ^3^Department of Pathology, Infectious Diseases, and Immunology, Vanderbilt University Medical Center, Nashville, TN, USA; ^4^Department of Clinical Pharmacology, Medical University, Vienna, Austria; ^5^Shionogi & Co., Ltd., Osaka, Japan; ^6^Shionogi Inc., Florham Park, NJ, USA; ^7^Infectious Disease Drug Development Consulting, LLC., Easton, CT, USA

*Critical Care* 2021, **25**(**Suppl 1**): P124

Introduction: We assessed whether the efficacy of cefiderocol (CFDC) was reduced when patients with serious carbapenem-resistant (CR) infections received iron supplementation. CFDC is the first siderophore, iron-chelator cephalosporin developed for the treatment of adult patients with serious CR Gram-negative infections with limited treatment options. Critically ill patients with anemia eventually receive iron supplementation or blood transfusions.

**Methods**: CREDIBLE-CR, an open-label randomized 2:1, Phase 3 study, investigated the efficacy of CFDC 2 g (or renal function-adjusted dose), q8h, or best available therapy (BAT; ≤ 3 non-siderophore antibiotics) for 7–14 days in hospitalised critically ill patients with serious CR infections [1]. Clinical cure and microbiological eradication were evaluated at end of treatment (EOT), test of cure (TOC) and follow-up (FU). Day 28 all-cause mortality (ACM) was compared for patients with or without iron supplementation given at any time up to EOT.

**Results**: Of 118 patients with confirmed CR pathogens at baseline, 42.5% (34/80) in the CFDC arm and 36.8% (14/38) in the BAT arm received blood transfusions and/or iron preparations up to EOT. Clinical cure rates were similar between CFDC and BAT at TOC with (41.2% [14/34] and 35.7% [5/14], respectively) and without (60.9% [28/46] and 58.3% [14/24], respectively) iron supplementation or transfusion; microbiological eradication showed a similar pattern (Fig. 1). In the safety population, Day 28 ACM with CFDC was 23.8% (10/42) for patients with and 25.4% (15/59) for those without iron supplementation and/or transfusion. In the BAT arm, Day 28 ACM was 27.8% (5/18) and 12.9% (4/31), respectively.

**Conclusions**: CFDC was as efficacious as BAT in the treatment of patients receiving blood transfusion and/or iron preparation. This suggests that iron supplementation and/or transfusion does not interfere with the antibacterial activity of CFDC.

**Reference**

1. Bassetti M et al. Lancet Infect Dis 21:226–240, 2021.

**Figure:**
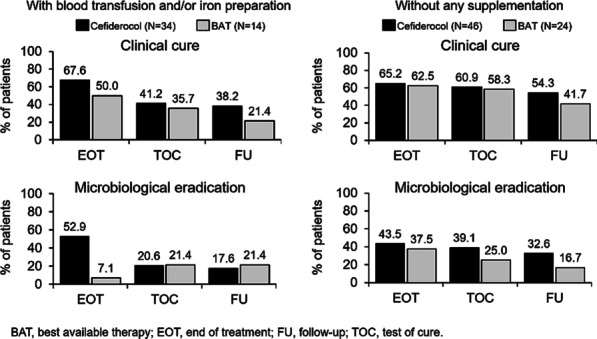
**Fig. 1** **(abstract P124)** Clinical cure and microbiological eradication rates in patients with or without iron supplementation in the CREDIBLE-CR study

## P125

### Microcirculatory dysfunction in sepsis-associated acute kidney injury is not equivalent to impaired tissue oxygenation

#### C. L. Manrique-Caballero^1^, J. Toro^1^, C. Baty^2^, M. Oberbanscheidt^3^, G. Del Rio-Pertuz^1^, A. Frank^1^, M. R. Pinsky^1^, S. Vinogradov^4^, J. A. Kellum^1^, H. Gomez^1^

##### ^1^Center for Critical Care Nephrology - Department of Critical Care Medicine, University of Pittsburgh, Pittsburgh, USA; ^2^Renal-Electrolyte Division - Department of Medicine, University of Pittsburgh, Pittsburgh, USA; ^3^Department of Surgery, University of Pittsburgh, Pittsburgh, USA; ^4^Department of Biochemistry and Biophysics and Department of Chemistry, University of Pennsylvania, Philadelphia, USA

*Critical Care* 2021, **25**(**Suppl 1**): P125

**Introduction**: The mechanisms by which sepsis causes organ dysfunction are not well understood. Microcirculatory dysfunction has been proposed to cause sepsis-associated acute kidney injury (S-AKI). However, whether it induces S-AKI through tissue hypoperfusion or increased local inflammation remains unclear. We hypothesize that microcirculatory dysfunction can induce S-AKI in the absence of impaired renal cortical oxygenation.

**Methods**: C57Bl/6 mice (n = 5–8/group) were randomly assigned to cecal ligation and puncture (CLP)-induced sepsis or sham surgery. We measured the following outcomes at 24 h: renal injury using serum creatinine (sCr), systemic inflammation using IL-6, renal cortical oxygenation and mortality. Renal cortical oxygenation was measured by the phosphorescence quenching method using an established phosphorescent probe, Oxyphor PdG4, injected systemically, and an OxyLED fiber-optic phosphorometer. Partial pressures of oxygen (PO_2_) were measured in four locations of the kidney during 1 min each. Renal cortex microcirculatory flow was assessed using 2-photon intravital microscopy and quantified using the microvascular flow index (MFI).

**Results**: CLP resulted in higher levels of sCr (CLP 1.66 vs Sham 0.98; p < 0.001) (Fig. 1.1), Il-6 (CLP 7759.28 vs Sham 79.1; p = 0.01) (Fig. 1.2), microcirculatory dysfunction evidenced by an MFI < 2.5 (CLP 1.88 vs Sham 2.59; p = 0.06) (Fig. 1.3), and higher mortality (CLP 64.2% vs Sham 0%; p < 0.01) (Fig. 1.4). Importantly, the renal cortex oxygenation was higher in the CLP group (PO_2_: CLP: 53.7 mmHg vs Sham 41.2 mmHg; p < 0.001) (Fig. 1.5).

**Conclusions**: Sepsis induced a decrease in renal cortical microcirculatory blood flow concomitant with an increase in cortical partial pressure of oxygen. This suggesting that local regulation of microcirculatory blood flow relative to tissue demand was impaired and that this mechanism may contribute to the development of S-AKI. Further studies will need to address if this is secondary to a mechanism akin to tissue ‘hibernation’ vs. mitochondrial dysfunction.

**Figure:**
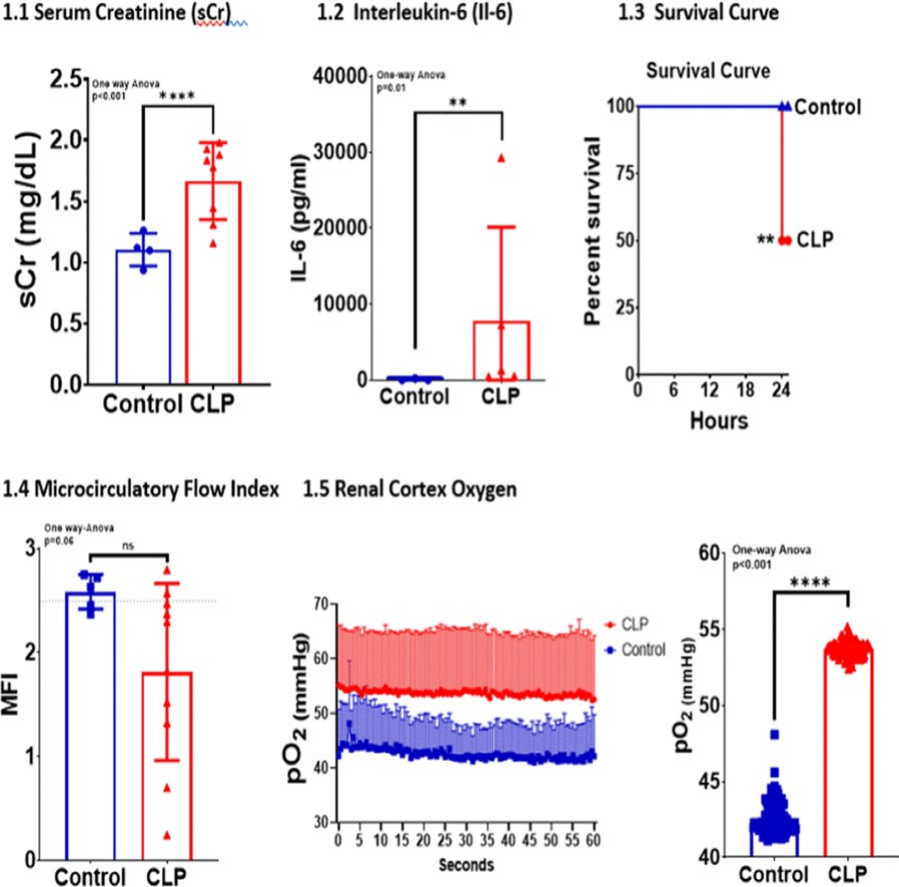
**Fig. 1** **(abstract P125)** Results

## P126

### Oliguria in critically ill patients: impact on AKI classification and outcomes prediction

#### N. Bianchi^1^, L. Stavart^2^, L. Stavart^2^, M. Altarelli^1^, T. Kelevina^1^, M. Faouzi^3^, A. Schneider^1^

##### ^1^Adult Intensive Care Unit, Centre Hospitalier Universitaire Vaudois, Lausanne, Switzerland; ^2^Faculty of Biology and Medicine, University of Lausanne, Lausanne, Switzerland; ^3^Division of Biostatistics, Center for Primary Care and Public Health (Unisanté), University of Lausanne, Lausanne, Switzerland

*Critical Care* 2021, **25**(**Suppl 1**): P126

**Introduction**: The relevance of KDIGO oliguria-based criteria for acute kidney injury (AKI) is disputed. We aimed to determine their impact on AKI diagnosis, severity assessment and mortality prediction.

**Methods**: We conducted a cohort study including all adult patients admitted to our unit between 2010 and 2020. Daily serum creatinine (sCr) and hourly urinary output (UO) measurements along with socio-demographic characteristics and severity scores were extracted. Long-term mortality was assessed by cross-referencing our database with the Swiss national death registry. We determined the onset and severity of AKI according to KDIGO classification using UO and sCr criteria separately and assessed their agreement. Using a multivariable model accounting for baseline characteristics, severity scores and sCr stages, we evaluated the relative influence of UO criteria on 90-day mortality. Sensitivity analyses were conducted to assess the impact of missing sCr, body weight and UO values.

**Results**: Among the 15,620 patients included [10′330 (66.1%) males, median age 65.0 years (IQR 53.0–75.0), median SAPS score 40.0 (IQR 30.0–53.0), median follow-up 67.0 months (IQR 34.0–100.0)], 12,143 (77.7%) fulfilled AKI criteria. SCr and UO criteria had poor agreement on AKI diagnosis and staging (Cohen’s weighted kappa = 0.36, 95% CI 0.34–0.37, p < 0.001). Compared to the isolated use of sCr criteria, consideration of UO criteria enabled to identify AKI in 5,630 (36.0%) patients. Those patients had a higher 90-day mortality than no-AKI patients (respectively 12.9% and 8.3%, p < 0.001). On multivariable analysis accounting for sCr stage, comorbidities and illness severity, UO stage 2 and 3 were associated with a higher 90-day mortality [OR 2.4 (1.6–3.8), p < 0.001, and 6.2 (3.7–10.5), p < 0.001, respectively] (Fig. 1). These results remained significant in all sensitivity analyses.

**Conclusions**: Oliguria lasting more than 12 h (KDIGO stage 2 or 3) has major diagnostic and prognostic implications, irrespective of sCr elevations.

**Figure:**
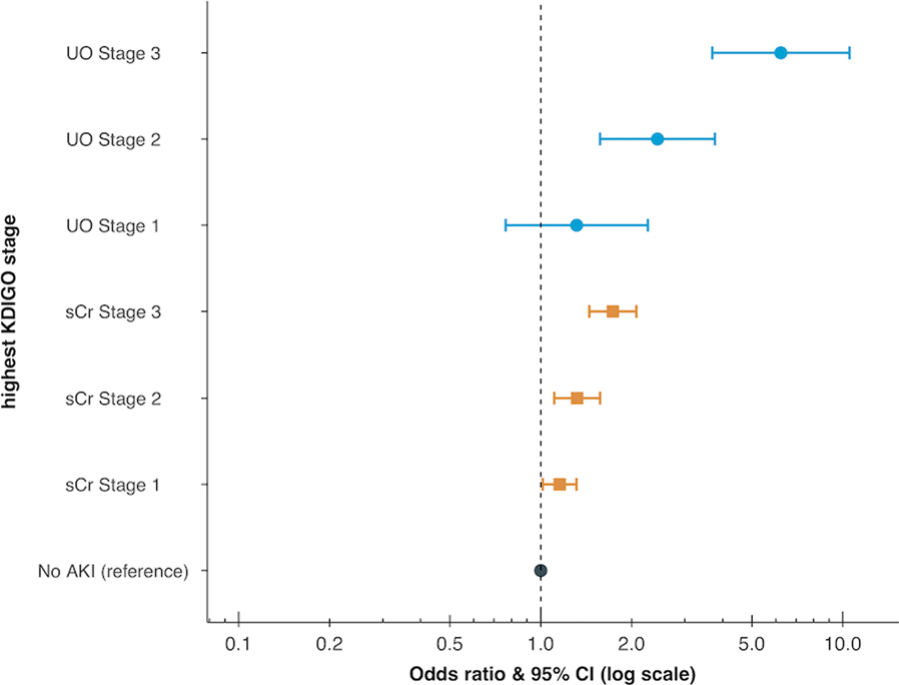
**Fig. 1** **(abstract P126)** Adjusted odds ratio for 90-day mortality per AKI severity stage according to KDIGO sCr or UO criteria. The relation between AKI severity and 90-day mortality was explored in a multivariate logistic regression model. Variables included in the model were age at ICU admission, baseline sCr, SAPS II score, Charlson score, and main ICU diagnosis. As no collinearity was found between KDIGO sCr and UO criteria both were included together in the model. Goodness of fit assessed by the Hosmer–Lemeshow test, χ2 = 430.47, df = 498, p = 0.99. Discrimination power assessed by the area under the receiver operating characteristic curve for the model for 90-day mortality = 0.87 (95% CI 0.86 – 0.87), p = 0.014. Patients included in the analysis, n = 14,852/15,551 (95.5%)

## P127

### Risk factors of acute kidney injury after lung transplant recipients in early postoperative period

#### P. Nadziakiewicz^1^, M. Wajda-Pokrontka^1^, A. Krauchuk^2^, M. Ochman^3^, P. Przybytowski^4^

##### ^1^Cardiac Anesthesia and Intensive Therapy, Silesian Centre for Heart Diseases, Zabrze, Poland; ^2^Szkola Doktorska, Medical University of Silesia, Katowice, Poland; ^3^Department of Lung Transplantation, Silesian Centre for Heart Diseases, Zabrze, Poland; ^4^Department of Cardiac Surgery, Heart Transplantation and Mechanical Circulatory Support, Silesian Centre for Heart Diseases, Zabrze, Poland

*Critical Care* 2021, **25**(**Suppl 1**): P127

**Introduction**: Acute kidney injury (AKI) after lung transplant (LTx) is the one of the most serious complications, connected to increased mortality and morbidity. Identification of predisposing factors can improve standards of care and outcome.

**Methods**: Retrospective analysis of 27 consecutive LTx recipients in Silesian Center for Heart Diseases in Zabrze, Poland operated in 2015 and 2016. The level of AKI according to KDIGO guidelines was noted in 7 postoperative days period. Patients were divided according to KDIGO level 1 and 2 – (group A – 9 patients) and 3 (group C – 14 patients). One patient died and was excluded from analysis.

**Results**: AKI was developed by 23 of 26 analyzed subjects (88.46%). Level 1 was noted in 3 cases (11.54%) and Level 2 in 6 (23.08%). Serious AKI - 14 patients (53.85%) and 4 of them needed renal replacement therapy. We did not find differences in preoperative creatinine levels. Patients did not differ according do age sex and BMI between groups. Group C subjects more often suffer from pulmonary hypertension (9 vs 3 p = 0.146) and diabetes (5 vs 1 p = 0.123), but it does not reach statistical significance. In group C additionally operation tends to last longer, be more often performed with the use of cardiopulmonary bypass and patients need more transfusions.

**Conclusions**: It seems to be probable that pulmonary hypertension and diabetes could be significant risk factor of high-grade acute kidney injury development after lung transplantation. Identification of factors modifying renal insufficiency development in lung transplant recipients needs further investigations.

## P128

### Persistent severe acute kidney injury (PS-AKI) is associated with higher health resource utilization (HRU) and costs

#### J. Textoris^1^, N. Rosenthal^2^, L. Carabuena^2^, J. P. Kampf^3^, T. Rodriguez^3^, A. Sanghani^3^, J. Echeverri^4^, P. McPherson^3^, M. Blackowicz^4^, R. Mackey^2^

##### ^1^Service d´Anesthésie et de Réanimation, bioMerieux, Inc., Lyon Cedex 03, France; ^2^Premier, Inc., Premier Applied Sciences, Charlotte, NC, USA; ^3^Global Medical Affairs, bioMerieux, Inc., Durham, NC, USA; ^4^Global Medical Affairs, Baxter Healthcare, Deerfield, IL, USA

*Critical Care* 2021, **25**(**Suppl 1**): P128

**Introduction**: We aimed to compare HRU and costs for patients with persistent severe AKI (PS-AKI) to those for patients with non-persistent severe AKI (NPS-AKI).

**Methods**: We conducted a retrospective observational study of hospitalized US adults using the Premier Healthcare Database from January 1, 2017 to December 31, 2019, with 30-day (d) follow-up for outcomes and 12-month look-back period (LBP) for baseline serum creatine (SCr) level and comorbidities, including the Charlson Comorbidity Index (CCI). “Index” admission was the first during the study period that met inclusion criteria (age ≥ 18 yr, hospital length of stay (LOS) ≥ 3 d with ≥ 3 SCr measures, and KDIGO stage 2 or 3 AKI [SCr criteria] and exclusion criteria (≥ 2 dialysis visits during LBP; or ECMO, stage 5 CKD, eGFR < 15 ml/min/1.73 m^2^, baseline SCr ≥ 4.0 mg/dl, or renal transplant during index hospitalization or LBP). PS-AKI was defined as 1) AKI stage 3 lasting ≥ 3d or with death within 3d, without intervening lower AKI stage, or 2) AKI stage 2/3 with dialysis within 3d. A second definition, PS-AKI by SCr only (PS-AKISCrO), excluded PS-AKI defined by dialysis or death. Regression models adjusted for age, sex, race-ethnicity, CCI, hospital characteristics (number of beds, teaching status, region, urban/rural), admission type and point of origin, medical vs surgical MS-DRG, primary payer, presence of CKD or sepsis, and ICU stay.

**Results**: Of 126,528 AKI stage 2/3 patients, 75.6% had NPS-AKI, 24.4% had PS-AKI and 15.3% had PS-AKISCrO. Compared to NPS-AKI, PS-AKISCrO had ≥ 38% longer mean index LOS (4.8 d) and ICU LOS (2.8 d), and 11% longer readmission LOS (1.0 d). PS-AKISCrO also had ≥ 39% higher index and ICU costs, 17% higher readmission costs and 8% higher outpatient costs over 30 days (Fig. 1). Results were similar or greater for PS-AKI. P < 0.01 for all comparisons (PS-AKI and PS-AKISCrO vs NPS-AKI) in adjusted models.

**Conclusions**: Persistent severe AKI is independently associated with longer LOS and higher costs during index hospitalization and 30-day follow-up.

**Figure:**
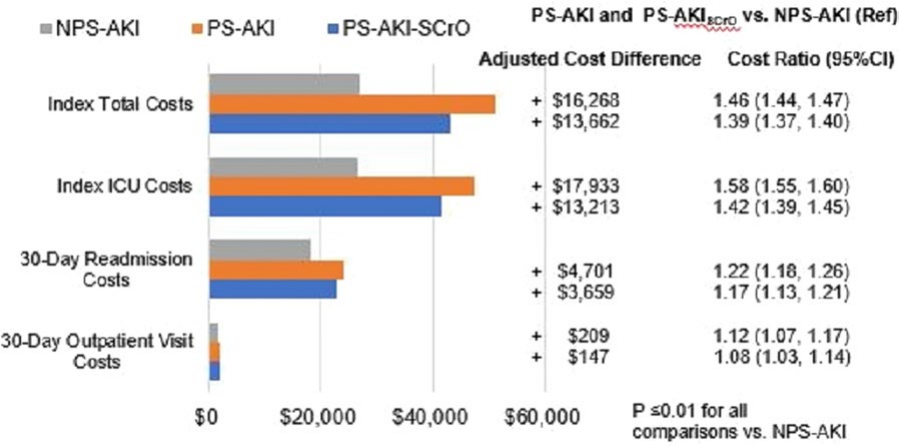
**Fig. 1** **(abstract P128)** Unadjusted mean costs, adjusted mean difference and ratio (95%CI) for PS-AKI and PS-AKISCrO vs. NPS-AKI (ref)

## P129

### Acute kidney injury, renal replacement therapy, and long-term outcomes in patients receiving veno-venous extracorporeal membrane oxygenation

#### N. Lumlertgul, R. Wright, G. Hudson, J. Kusic, G. Vlachopanos, K. Lee, L. Camporota, N. A. Barrett, M. Ostermann

##### Critical Care, Guy´s & St Thomas´ NHS Foundation Trust, London, UK

*Critical Care* 2021, **25**(**Suppl 1**): P129

**Introduction**: Acute kidney injury (AKI) is a frequent complication in patients with severe respiratory failure receiving extracorporeal membrane oxygenation (ECMO). However, long-term renal function is rarely monitored in survivors. This study aimed to assess the long-term mortality and renal outcomes in patients receiving ECMO.

**Methods**: This was a single-center retrospective observational study of adult patients (≥ 18 years old) who were treated with veno-venous ECMO at Guy’s & St Thomas’ Hospital between January 1, 2010 and December 31st, 2017. We excluded patients with end-stage renal disease (ESRD), kidney transplant recipients, and patients who received veno-arterial ECMO. The primary outcome was 1-year mortality. The secondary outcomes were 1-year incidence of ESRD and chronic kidney disease (CKD) in patients with and without AKI.

**Results**: A total of 300 patients (57% male; median age 44.5 (IQR, 34–54) were included in the final analysis. Past medical histories included diabetes (12%), hypertension (17%), and CKD (2.3%). The main indication for ECMO was respiratory infection (72%). AKI occurred in 230 patients (76.7%) [AKI stage 1 (79.4%), stage 2 (7.4%), stage 3 (13.2%)]. RRT was commenced in 59.5% of all patients. Patients with AKI were more likely to have longer mechanical ventilation duration, in-hospital mortality, RRT dependence, and higher creatinine at discharge. One-year mortality was 27.8% in AKI vs. 21.4% in non-AKI patients (p = 0.18). ESRD occurred in 3 patients in the AKI group. At 1-year, only 95/237 (40.1%) survivors had creatinine results available. Among these, CKD was found in 27% of AKI patients compared with 4% in non-AKI patients (p = 0.02).

**Conclusions**: In vv-ECMO patients, one-year mortality is non-significantly higher in patients with AKI than in those without AKI. Follow-up of renal function post-ECMO was infrequent. In patients with follow-up creatinine measurements, the CKD incidence was extremely high at 1 year. More awareness about this serious complication is required.

## P130

### COVID-19: impact on circuit lifetime and performance during continuous renal replacement therapy

#### L. Whiting, N. Bianchi, S. Abed-Maillard, A. Schneider

##### Intensive Care, Centre Hospitalier Universitaire Vaudois, Lausanne, Switzerland

*Critical Care* 2021, **25**(**Suppl 1**): P130

**Introduction**: The impact of SARS-CoV-2 associated coagulopathy on continuous renal replacement therapy (CRRT) circuit lifespan and performance remains unknown.

**Methods**: In this prospective observational study, we enrolled all consecutive patients who received CRRT in our intensive care unit September and December 2020. We collected patients’ baseline characteristics, laboratory results, CRRT circuit lifespan as well as plasma and effluent samples at 12 (T1), 24 (T2), 48 (T3) and 72 h (T4) of CRRT circuit initiation. At each study time point, we computed urea, creatinine and β2-microglobulin clearance. Results obtained in patients with COVID-19 (C19 group) were compared to those without COVID-19 (control group). Circuits’ lifespan was assessed using Kaplan–Meier estimates and compared using log-rank test. Filter clearances at each study time point were compared using Student’s T-test.

**Results**: We included 35 patients, 26 (74%) males (median age 68 [IQR 57– 71] years). Of those 16 (45%) were COVID-19 positive. We analyzed 150 CRRT circuits: 77 (51.3%) in the C19 group and 73 (48.7%) in the control group. Compared to patients in the control group, those in the C19 group had a significantly shorter median circuit lifespan (52 [37.0–69.5] versus 66 [49–71.5] hours, p = 0.024) (Fig. 1). They had a lower median urea (T1 27.5 vs 31.4; T2 26.8 vs 32.6; T3 27.3 vs 30.4 and T4 26.4 vs 30.7 ml/kg/h, all p < 0.05) and creatinine (T1 22.7 vs 24.7; T2 23.0 vs 24.5; T3 20.4 vs 22.9 and T4 21.3 vs 22.8 ml/kg/h, respective p values: 0.03, 0.02, 0.06 and 0.08) clearance at all study time points. However, there was no difference in β2-microglobulin’s clearance between the two groups (respective p values: 0.6, 1.0, 0.8 and 0.7).

**Conclusions**: Patients with COVID-19 disease had a shorter CRRT circuit lifetime and a lower urea and creatinine clearance. The magnitude of this difference was, however, limited and further studies are required to explore clinical implications of such differences.

**Figure:**
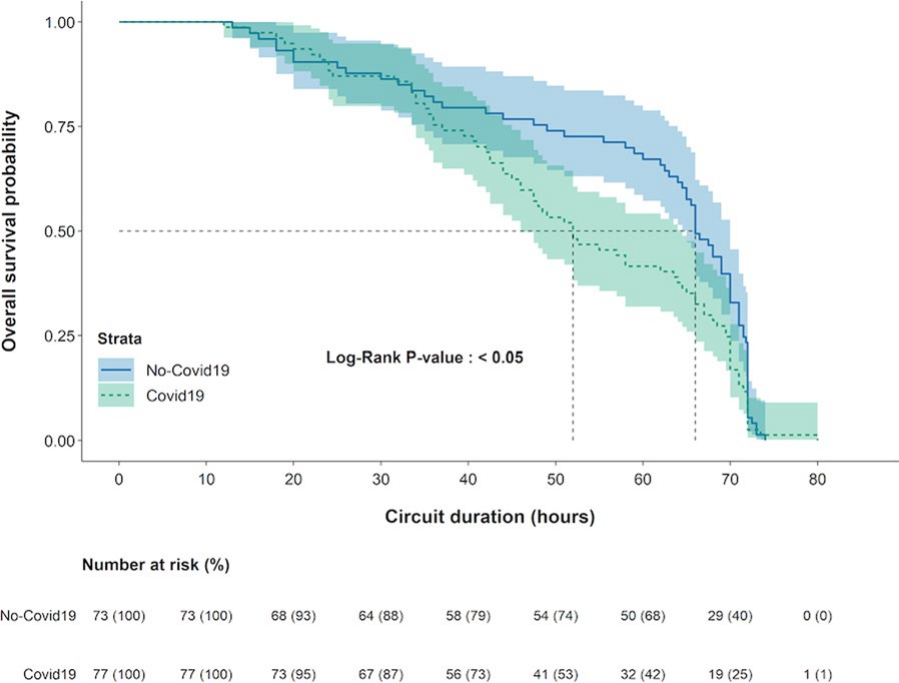
**Fig. 1** **(abstract P130)** Kaplan–Meier analysis presenting circuit lifespan in COVID-19 group versus control group. Median circuit lifespan was 52 [37.0–69.5] hours in the COVID-19 group versus 66 [49.0–71.5] hours in the control group. p value = 0.024 using log-rank test comparison

## P131

### Influence of continuous renal replacement therapy on fosfomycin serum levels in critically ill patients

#### T. H. Hüppe^1^, A. Fernandes^1^, F. Maurer^1^, A. Meiser^1^, K. Götz^2^, S. Kreuer^1^

##### ^1^Intensive Care Medicine and Pain Therapy, Saarland University Medical Center and Department of Anaesthesiology, Saarland University Faculty of Medicine, Homburg, Germany; ^2^Saarmetrics GmbH, Saarland University, Department of Anaesthesiology, Saarbrücken, Germany

*Critical Care* 2021, **25**(**Suppl 1**): P131

**Introduction**: Fosfomycin plays an important role in the therapy of multi-resistant organisms in critically ill patients. To date, no data are available on pharmacokinetics of fosfomycin in patients who require continuous renal replacement therapy. In order to achieve adequate serum levels and avoid overdosing, this knowledge is essential. We hypothesize that dialysis leads to a significant decrease in serum levels and a shortening of elimination half-life.

**Methods**: We determined 300 serum fosfomycin levels in 15 critically ill patients using high performance liquid chromatography. Samples were taken before and after 15, 30, 60, 90, 120, 180, 240, 300 and 360 min after intravenous administration of 5 g fosfomycin during multi-filtration dialysis and withdrawal of renal replacement therapy, each in the same patient. Using E max model for time-concentration curves we determined peak concentration, time to half-maximum concentration and gamma, each for rise and fall in concentration, elimination half-life as well as area under the curve (AUC) of elimination.

**Results**: 15 patients had a mean age of 60 (± 8 SD) years, mean weight of 88.5 (± 19.8 SD) kg and mean height of 175.6 (± 19 SD) cm. 13 patients were male. Dialysate flow was 2.4 (± 0.5 SD) l/h, blood flow 110 (± 27 SD) ml/min and ultrafiltration 73 (± 57 SD) ml/h. Fosfomycin peak concentration was significant lower during renal replacement therapy (117 µg/ml; ± 59.9 SD) compared to discontinuation of multi-filtration (143 µg/ml; ± 54.7; p < 0.05). Elimination half-life was 114 min (25th-75th percentiles 103.5–156.5) for dialysis compared to 570 min (25th-75th percentiles 196–1172; p < 0.05). Renal replacement therapy resulted in a 59% (± 37.2 SD) reduction of AUC of fosfomycin elimination.

**Conclusions**: Continuous renal replacement therapy results in significant lower fosfomycin serum levels and a significantly shorter elimination half-life, which should considered in dosing fosfomycin during renal replacement therapy.

**Acknowledgement**: The study was financially supported by Infectopharm GmbH, Heppenheim, Germany.

## P132

### Exploring population pharmacokinetic models in patients treated with vancomycin during continuous venovenous hemodiafiltration (CVVHDF) on different anticoagulant modalities

#### Y. Kelly^1^, M. Coyle^2^, E. Deasy^2^, P. Lavin^3^, M. Donnelly^1^, D. D’Arcy^4^

##### ^1^Intensive Care, Tallaght University Hospital, Dublin, Ireland; ^2^Clinical Pharmacy, Tallaght University Hospital, Dublin, Ireland; ^3^Nephrology, Tallaght University Hospital, Dublin, Ireland; ^4^School of Pharmacy and Pharmaceutical Sciences, Trinity College Dublin, Dublin, Ireland

*Critical Care* 2021, **25**(**Suppl 1**): P132

**Introduction**: Achievement of target concentrations for antibiotics using therapeutic dose monitoring (TDM) is particularly challenging in septic patients requiring renal replacement therapy.

**Methods**: We conducted an exploratory population pharmacokinetic (PK) analysis in our single center tertiary level intensive care unit (ICU) on PK of vancomycin following intermittent infusion in critically ill patients receiving continuous venovenous hemodiafiltration (CVVHDF). This retrospective study extracted clinical, laboratory and dialysis data from the electronic healthcare record (EHR), using strict inclusion criteria. A population PK analysis was conducted with a one compartment model using the PMetrics population PK modelling package. A base structural model was developed, with further analyses using clinical and dialysis related data to see if model prediction could be improved through covariate inclusion. The final selected model was used to simulate patient concentrations to investigate the probability of different dosing regimens achieving target therapeutic concentrations.

**Results**: 107 vancomycin dosing intervals (155 levels) in 24 patients were examined. An acceptable base model was produced (Plots of observed vs. population predicted concentrations (Obs-Pred) R2 = 0.78). No continuous covariates used resulted in a clear improvement over the base model. Use of anticoagulation modality and vasopressor use as categorical covariates resulted in similar PK parameter estimates, with a trend towards lower parameter estimate variability when using RCA or without vasopressor use. Simulations using PTA plots suggested that a 2 g loading dose followed by 1.5 g in 24 h as maintenance dose, commencing 12 h after loading, is required to achieve adequate early target trough concentrations of at least 15 mg/l.

**Conclusions**: Simulations based on PTA plots showed we could achieve acceptable vancomycin trough concentrations early in treatment with a 2 g loading dose and maintenance dose of 750 mg 12 hourly in CVVHDF.

## P133

### Population pharmacokinetics of multiple dose fosfomycin in critically ill patients during continuous veno-venous hemodialysis

#### K. Götz^1^, S. Kreuer^1^, T. Hüppe^2^, T. Lehr^1^

##### ^1^Saarmetrics GmbH, Saarbruecken, Germany; ^2^Saarland University Medical Center and Department of Anaesthesiology, Intensive Care and Pain Therapy, Saarland University Faculty of Medicine, Homburg, Germany

*Critical Care* 2021, **25**(**Suppl 1**): P133

**Introduction**: To investigate the pharmacokinetics (PK) of intravenous fosfomycin in critically ill patients with and without continuous veno-venous hemodialysis (CVVHD), a population pharmacokinetic model was developed.

**Methods**: Critically ill patients with acute renal failure and need of CVVHD were included. Patients received 5 g fosfomycin over 120 min every 6 h intravenously. PK was measured two times for each patient, once with CVVHD and once without. Model development and simulation was performed using non-linear mixed effects modelling (NONMEM® V.7.4.3).

**Results**: Two of 15 patients were female. Median (range) age was 57 (49–80) years. Six patients were anuric. Creatinine clearance (CRCL) ranged from 5.89 to 210 ml/min for patients retaining urine production. 300 blood samples were collected in total. A two-compartment model with zero-order input and inter-individual variability on intrinsic clearance (CLRENAL) and volume of the central compartment (Vc) was used. An additional dialysis clearance (CLCVVHD) was incorporated. CRCL accounted for the variability on CLRENAL. VC increased linearly with time after first dose. Population estimates were 0.34 l/h for CLRENAL, 18.1 l for VC at first dose, 4.89 l/h for intercompartmental clearance and 19.7 l for volume of the peripheral compartment. Simulations on basis of the model showed that approved daily doses of 12 to 24 g intravenous fosfomycin in two or three daily doses do not result in critical accumulations within five days after the first dose in anuric patients with and without CVVHD. For patients with CRCL > 50 ml/min and CVVHD, dosage should be increased to at least 15 g fosfomycin in three daily doses.

**Conclusions**: Model based simulations reveal a safe and effective use of the approved fosfomycin dosing regimens against bacteria with minimum inhibitory concentrations up to 64 mg/l in critically ill patients with and without CVVHD.

**Acknowledgements**: The project was financially supported by Infectopharm GmbH, Heppenheim, Germany.

## P134

### Effects of regional citrate anticoagulation on thrombin generation, fibrinolysis and platelet function in critically ill patients on CRRT

#### R. Fisher^1^, G. Moore^2^, M. Mitchell^2^, L. Dai^2^, S. Crichton^3^, N. Lumlertgul^4^, M. Ostermann^5^

##### ^1^Department of Critical Care, King´s College Hospital, London, UK; ^2^Department of Haemostasis & Thrombosis, Guy´s & St Thomas´ Hospital, London, UK; ^3^MRC Clinical Trials Unit, University College London, London, UK; ^4^Departments of Critical Care and of Haemostasis & Thrombosis, Guy´s & St Thomas´ Hospital, London, UK; ^5^Departments of Critical Care & Nephrology, Guy´s & St Thomas´ Hospital, London, UK

*Critical Care* 2021, **25**(**Suppl 1**): P134

**Introduction**: Regional citrate anticoagulation (RCA) is recommended as first line anticoagulation for continuous renal replacement therapy (CRRT). Studies report variable filter life despite optimal citrate protocols. The aims of this study were i) to investigate whether citrate affects thrombin generation, fibrinolysis and platelet function, and ii) to explore whether systemic blood samples are representative of intra-circuit clotting parameters.

**Methods**: We screened critically ill patients who were prescribed CRRT for acute kidney injury (AKI). Patients with known thrombotic or bleeding tendencies and patients who had been prescribed blood products or other anticoagulants were expluded. In eligible patients, we measured coagulation parameters at baseline (pre-CRRT), followed by serial measurement of thrombin generation, D-dimer and platelet function during CRRT for up to 72 h. We also compared samples taken from the arterial line with paired samples taken directly from the circuit.

**Results**: A total of 11 patients were recruited (mean age 62.4, 82% male). At baseline, all patients had Factor VIII and von Willebrand Factor concentrations above reference range and also significantly increased peak thrombin generation. During CRRT, there was no significant variation in systemic maximum peak thrombin generation, time to peak thrombin generation, fibrinolysis and platelet function analysis. There was no significant difference between paired samples taken from the arterial line and the circuit.

**Conclusions**: Critically ill patients with AKI requiring CRRT are hypercoagulable. RCA during CRRT does not affect thrombin generation, fibrinolysis or platelet function.

## P135

### Successful treatment of severe valproic acid intoxication with Cytosorb® hemoadsorption

#### J. Willems^1^, N. Hunfeld^2^, B. Van der Hoven^2^, H. De Geus^2^

##### ^1^Intensive Care, Franciscus Gasthuis & Vlietland, Rotterdam, Netherlands; ^2^Department of Intensive Care, Erasmus Medical Center, Rotterdam, Netherlands

*Critical Care* 2021, **25**(**Suppl 1**): P135

**Introduction**: Valproic acid (VPA) is moderately dialyzable in case of overdosing. In vitro data show effective removal with a hemadsorbent system [1]. We report the first successfully treated clinical case of a lethal VPA intoxication with CytoSorb® (CS) hemadsorption after an intermittent hemodialysis (IHD) session.

**Methods**: A 54-year-old male was admitted to the emergency department following auto intoxication with VPA, lorazepam and olanzapine. He was intubated because of lowered mental status (GCS 4). First measured VPA plasma concentration was 1320 mg/l with a free fraction of 1012 mg/l. IHD was commenced for 4 h. As VPA remained at toxic levels, a citrate-anticoagulated continuous veno-venous hemodialysis (CVVHD) circuit was initiated with a CS cartridge installed in pre-hemofilter position. Blood samples were collected at the adsorber in-and outlet to evaluate VPA plasma levels.

**Results**: At the start of CVVHD and CS therapy, VPA bound and free fraction plasma levels where 525 mg/ml and 335 mg/ml respectively. Combined treatment resulted in a decline to 50 mg/ml and 0 mg/ml over the course of 30 h (Fig. 1). A total of 3 adsorbers were used. CS showed saturation in approximately 3 h. On day 3 our patient was awake and extubated successfully. He was discharged to the psychiatric ward for further treatment.

**Conclusions**: Combined CVVHD and CS hemadsorption therapy is an effective add-on treatment for life-threatening VPA intoxication.

Patient written informed consent was obtained for publication.

**Reference**

1. Reiter K et al. Blood Purif 20:380–388, 2002

**Figure:**
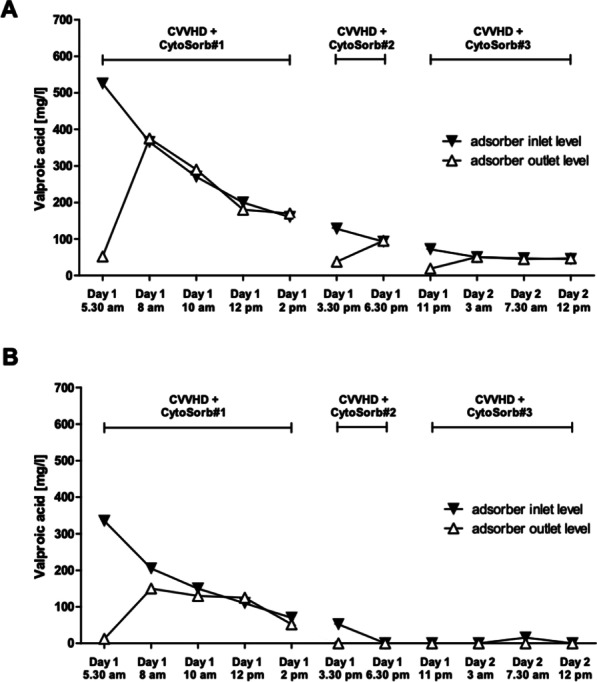
**Fig. 1** **(abstract P135)** Plasma VPA levels during CVVHD + CS treatment: A) Bound fraction B) Free fraction

## P136

### Reducing vasopressor requirements with hemoadsorption in the critically ill: a systematic review

#### C. Rao^1^, F. Hawchar^2^, A. Akil^3^, C. Rugg^4^, Y. Mehta^5^, J. Scheier^1^, H. Adamson^1^, D. Adam^1^, E. Deliargyris^6^, Z. Molnar^7^

##### ^1^Clinical, Medical and Health Economics Department, Cytosorbents Europe GmbH, Berlin, Germany; ^2^University of Szeged, Szeged, Hungary; ^3^Klinikum Ibbenbueren, Ibbenbueren, Germany; ^4^Medical University of Innsbruck, Innsbruck, Austria; ^5^Medanta the Medicity, Gurugram, India; ^6^Cytosorbents Corporation, Monmouth Junction, NJ, USA; ^7^University of Pécs, Pécs, Hungary

*Critical Care* 2021, **25**(**Suppl 1**): P136

**Introduction**: Hemoadsorption with CytoSorb therapy (CS) could lead to rapid shock reversal in patients with vasoplegic shock. This systematic review summarizes evidence on the impact of CS on vasopressor requirements.

**Methods**: A systematic search was conducted (last update: May 8, 2021) and studies reporting norepinephrine (NE) doses before and after CS were selected for a descriptive analysis. Four studies in patients with hyperinflammation-induced vasoplegia that included control cohorts were used for a comparative analysis. Effect size was expressed as reduction in NE dose from baseline to 24 h.

**Results**: Thirty-six studies totaling 447 patients reported NE dose before and after CS. Significant NE dose reduction was noted with CS (before CS: 0.55 [0.39–0.86] vs. after CS 0.1 [0.00–0.25] μg/kg/min, p < 0.001). In the comparative analysis, a statistically significant benefit after 24 h of CS was observed (Hedge's g: 1.59, 95% CI 0.48- 2.70); however, the I^2^ statistic suggested a high heterogeneity between studies [1–4] (Fig. 1).

**Conclusions**: Despite the limited number of studies, this analysis indicates that CS may reduce NE needs in vasoplegic shock patients. Hemodynamic stability could be a novel endpoint to assess hemoadsorption efficacy in future trials.

**References**

1. Hawchar F et al. J Crit Care 49:172–8, 2019
2. Akil A et al. Thorac Cardiovasc Surg 69:246–51, 2021
3. Mehta Y et al. World J Crit Care Med 9:1–12, 2020
4. Rugg C et al. Biomedicines 8, 2020

**Figure:**
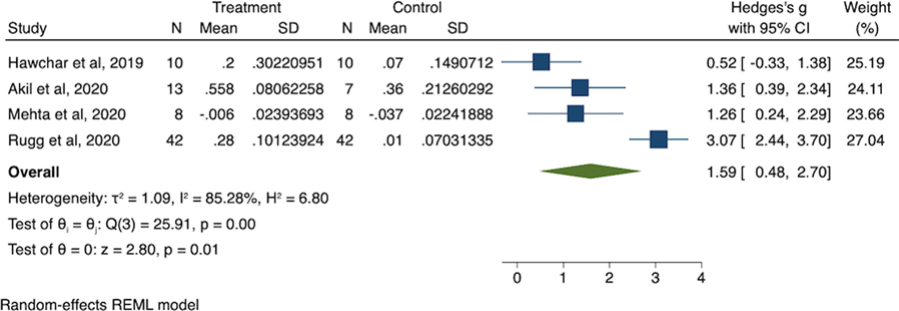
**Fig. 1** **(abstract P136)** Forest plot for efficacy of CS-therapy to reduce NE requirements at 24 h

## P137

### RRT with the oXiris membrane decrease PAI-1: effect on TEG parameters

#### F. Turani^1^, G. Barettin^1^, P. Zulli^2^, S. Busatti^1^, M. Maisto^2^, F. Candidi^2^, V. Cotticelli^2^, M. De Paola^2^

##### ^1^Anesthesia and Intensive Care, Aurelia and European Hospital, Rome, Italy; ^2^Aurelia And European Hospital, Rome, Italy

*Critical Care* 2021, **25**(**Suppl 1**): P137

**Introduction**: RRT with adsorbing membranes is used in septic shock with AKI, but the anti coagulation may be problematic when a pro-inflammatory and anti-fibrinolytic response is present. Aim of this study is: 1- to evaluate the changes of plasminogen activator inhibitor 1 (PAI-1), a key suppressor of fibrinolysis, during RRT with an adsorbing membrane; 2 - the time course of the thromboelastographic parameters.

**Methods**: Thirty patients with sepsis and AKI were submitted to RRT with the adsorbing membrane oXiris (Baxter, USA) in CVVHDF mode integrated into the Prismaflex platform (Baxter, USA). Pre-filter citrate was used as loco–regional anticoagulation. In all the patients arterial and post-filter TEG was performed at basal time (T0) and at 24 h of treatment (T1) and an arterial sample was obtained to calculate plasmatic PAI-1 (Elisa method) at the same times. All data are expressed as mean and SD or median and IQ. For comparison beween data T test student or non parametric test were used. The significance level was p < 0.05.

**Results**: All the treatments were completed without major complications. Arterial TEG parameters did not change from T0 to T1 and were in the normal range, whereas post-filter TEG parameters parameters had longer R time and reduced MA amplitude. Plasmatic PAI-1 decreased from 46 ± 30 to 17 ± 10 ng/ml (p < 0.05).

**Conclusions**: PAI-1, a key suppressor of fibrinolysis, may decrease durng CRRT with the membrane oXiris. This may improve the anti-coagulant response of the filter without negatively affect the patient coagulation.

## P138

### PMX hemoperfusion restores COVID-19-induced lymphopenia by removal of NETs-related nuclear proteins

#### N. Takeyama^1^, H. Mori^1^, T. Terajima^1^, M. Hattori^1^, S. Tanabe^1^, M. Tsuda^1^, S. Ito^2^, T. Irahara^1^

##### ^1^Department of Emergency and Critical Care Medicine, Aichi Medical University, Aichi, Japan; ^2^Department of Respiratory Medicine and Allergology, Aichi Medical University, Aichi, Japan

*Critical Care* 2021, **25**(**Suppl 1**): P138

**Introduction**: Previous studies demonstrated neutrophil extracellular traps (NETs) formation augmented during COVID-19 and the levels of circulating NETs-derived molecules correlated to the severity of COVID-19. We hypothesized that removal of circulating NETs-derived molecules from the bloodstream could be an effective strategy to reduce dysregulated inflammation in COVID-19. In this study, we examined whether treatment with direct hemoperfusion with polymyxin B (PMX)-immobilized fiber column (Toray Industries, Japan) can limit the progression of COVID-19.

**Methods**: We studied, 30 COVID-19 ARDS patients, who required admission to the ICU and mechanical ventilation from August 2020 to June 2021. Thirteen patients received PMX hemoperfusion within 12 h after ICU admission and blood samples were taken from them before and immediately after hemoperfusion. Plasma levels of NETs-related products including myeloperoxidase-DNA, neutrophil elastase-DNA, and cell free-DNA were measured.

**Results**: Plasma levels of NETs-related products collected at ICU admission increased in COVID-19. This result suggests that NETs would be accelerated by COVID-19. When plasma levels of NETs-related products and cytokines were measured before and after PMX, the former significantly decreased after PMX, whereas the latter did not change. Time-course change in lymphocytes count without PMX significantly decreased at 5–7 days when compared with 0–1 days. On the other hand, patients who were treated with PMX lymphocytes counts significantly increased at 5–7 days compared with patients without PMX.

**Conclusions**: We demonstrated that PMX membrane can effectively capture various nucleus-derived molecules. Furthermore, scavenging nucleus-derived molecules by treatment with PMX inhibited the progression of lymphopenia. Removing systemic nucleus-derived molecules from circulation by PMX may ameliorate irrelevant inflammatory and thrombotic complications in patients with COVID-19.

## P139

### CytoSorb therapy in COVID-19 (CTC) patients requiring extracorporeal membrane oxygenation: a multicenter, retrospective registry

#### T. Song^1^, J. Hayanga^2^, L. Durham^3^, L. Garrison^4^, A. Supady^5^, M. Jaros^6^, P. Nelson^7^, E. Deliargyris^7^, N. Moazami^8^

##### ^1^University of Chicago Medicine, Chicago, IL, USA; ^2^West Virginia School of Medicine, Morgantown, WV, USA; ^3^Medical College of Wisconsin, Milwaukee, WI, USA; ^4^Franciscan Health Indianapolis, Indianapolis, IN, USA; ^5^University of Freiburg, Freiburg, Germany; ^6^Summit Analytical LLC, Denver, CO, USA; ^7^CytoSorbents Corporation, Monmouth Junction, NJ, USA; ^8^New York University School of Medicine, New York, NY, USA

*Critical Care* 2021, **25**(**Suppl 1**): P139

**Introduction**: CytoSorb is a cytokine adsorption device that received FDA Emergency Use Authorization (EUA) for use in critically ill COVID-19 patients. The CTC Registry was established to collect patient-level data from U.S. centers using CytoSorb under the EUA.

**Methods**: Consecutive patients on ECMO treated with CytoSorb were included. Retrospective data collection included demographics, comorbidities, COVID-19 medications, inflammatory biomarkers, and details on ECMO and CytoSorb use. Study follow-up was to hospital death or discharge. Primary outcome was ICU mortality. Comparisons between survivors and non-survivors were performed to evaluate predictors of mortality. Up-to-date information from the international Extracorporeal Life Support Organization (ELSO) ECMO COVID-19 Registry was considered to help contextualize the results.

**Results**: Fifty-two ECMO patients treated under EUA from April 2020 to April 2021 were enrolled from 5 U.S. centers. Baseline characteristics were comparable to the ELSO Registry except for higher rates of obesity in the CTC cohort. ICU mortality rates in the CTC cohort were 17.3% at 30 days, 26.9% at 90 days, and 30.8% overall. Gender, age, baseline SOFA, baseline D-dimer levels, and CytoSorb use between survivors and non-survivors are shown in the Table. Regression analyses suggested a borderline association between baseline D-dimer levels and mortality, with 32% increase in the risk of death per 1 µg/ml increase (p = 0.055). CytoSorb was generally well tolerated without any unanticipated device-related adverse events reported.

**Conclusions**: Combined use of ECMO and CytoSorb in critically ill COVID-19 patients was associated with mortality rates that compared favorably to international benchmarks. Elevated baseline D-dimer levels appeared to be associated with increased risk of mortality.

**Table:** **Table 1** **(abstract P139)** Comparison of survivors and non-survivors in the CTC ECMO cohort

Survivors (S) vs. Non-Survivors (NS)	S (n = 36)	NS (n = 16)	p value
Male	67% (24/36)	63% (10/16)	0.764
Age (years)	47.4 ± 9.54	51.2 ± 7.38	0.161
SOFA score at start of CytoSorb therapy	6.3 ± 3.64	8.3 ± 4.13	0.104
Baseline D-dimer levels (µg/ml)	2.0 ± 1.3	22.1 ± 36.7	0.056
Duration of CytoSorb therapy (hours)	79.4 ± 27.72	88.3 ± 35.11	0.326
Time to therapy after ECMO start (days)	2.3 ± 6.63	2.8 ± 4.54	0.781
Time to therapy after ICU admission (days)	6.1 ± 7.40	9.1 ± 5.60	0.168

## P140

### The SCRAM bag: a comparison between current practice versus a novel standardized approach for in-hospital pediatric emergency airway management

#### M. Wylie^1^, E. Waters^2^, J. McCormack^3^, P. Swinton^4^

##### ^1^Royal Infirmary Edinburgh, Anaesthetics and Critical Care, Edinburgh, UK; ^2^Ninewells Hospital, Anaesthetics and Critical Care, Dundee, UK; ^3^Royal Hospital for Children and Young People, Anaesthetics, Edinburgh, UK; ^4^Scottish Air Ambulance Service, Scottish Ambulance Service, Air Ambulance Division, Glasgow, UK

*Critical Care* 2021, **25**(**Suppl 1**): P140

**Introduction**: Emergency pediatric airway management and intubation require the completion of multiple individual tasks, under time pressure, with a high cognitive load, prone to error. The Pediatric Structured CRitical Airway Management (SCRAM) bag has been designed to rationalise and standardize the approach to pediatric airway management. We hypothesized that the use of the SCRAM bag in hospital on first exposure, with no prior training would perform at least as well as standard practice.

**Methods**: Twelve participants, comprising a combination of anesthetic registrars, operating department practitioners and pediatric emergency department nurses, were randomized into two groups and asked to prepare a ‘kit dump’ for a simulated pediatric emergency using either a standard resuscitation trolley or the SCRAM bag. Following at least a 2-week wash out period, each participant completed a second simulation using the alternative equipment set. The primary outcome measured was time taken to kit dump completion. Secondary outcome measures were the number of errors and self-reported cognitive load.

**Results**: Use of the SCRAM bag resulted in a shorter time to kit dump completion (95% confidence interval: 44.5 + 35.6; 8.9 to 80.1 s). This is an average reduction of 11.5%. 20% fewer errors and an average of 9.8% reduction in cognitive load were observed in the SCRAM group.

**Conclusions**: This study demonstrated that the SCRAM bag performed as well as established emergency airway preparation systems in the hospital setting. The SCRAM bag did not increase time to readiness nor increase errors, despite this being the first exposure with no prior training. This highlights ease of use which provides the user with the advantages of equipment standardization, portability and cognitive aids integrated within the SCRAM system.

## P141

### Do-not-intubate order and prolonged high flow nasal cannula therapy in patients with hypoxemic respiratory failure related to COVID-19

#### A. Van Hoorn^1^, M. Moretti^2^, M. Mekeirele^1^, J. Poelaert^1^

##### ^1^Department of Critical Care, UZ Brussel, Jette, Belgium; ^2^Department of Internal Medicine, UZ Brussel, Jette, Belgium

*Critical Care* 2021, **25**(**Suppl 1**): P141

**Introduction**: High-flow nasal cannula (HFNC) therapy can reduce the need for mechanical ventilation and seems to lower mortality rates in patients affected by Coronavirus disease 2019 (COVID-19) with hypoxemic respiratory failure (HRF). However, the effectiveness of HFNC therapy on mortality in these patients with a Do-Not-Intubate (DNI) order is unclear. Purpose: Assess mortality in patients affected by HRF due to COVID-19 with a DNI order treated with HFNC. Identify predictive factors for mortality in this cohort. Compare the use of HFNC and mortality between a first and second pandemic wave of COVID-19.

**Methods**: We retrospectively analyzed the medical records of all COVID-19 patients with a DNI order that received HFNC in UZ Brussel between March 6, 2020, and the January 1, 2021. Besides 30-day mortality, we focused on APACHE III score and P/F ratio at the start of HFNC. Furthermore, to evaluate an evolution in HFNC use, a comparative analysis was made between patients treated during the first and the second COVID-19 wave.

**Results**: A total of 57 patients treated with HFNC had DNI orders with a 30-day mortality of 84%. Higher APACHE III and lower P/F ratios at the start of HFNC were predictors for mortality. APACHE III scores higher than 47 had a positive predictive value for mortality of 97%. Survivors had a median APACHE III score of 45 (IQR 40–48). In the first wave, the duration of HFNC therapy was 3 (IQR2-5) vs. 6 (IQR3-10) days in the second, p = 0.005. Mortality was comparable 85% vs. 84%.

**Conclusions**: COVID-19-patients with HRF and DNI orders treated with HFNC have high mortality. Patients in the second wave received significantly longer HFNC therapy compared to the beginning of the COVID-19 pandemic, without improved survival. APACHE III at the start of HFNC is an early predictor for mortality with a high positive predictive value. In high APACHE scores with DNI orders, palliative care should be considered, as prolonged HFNC therapy does not improve the outcome.

**Figure:**
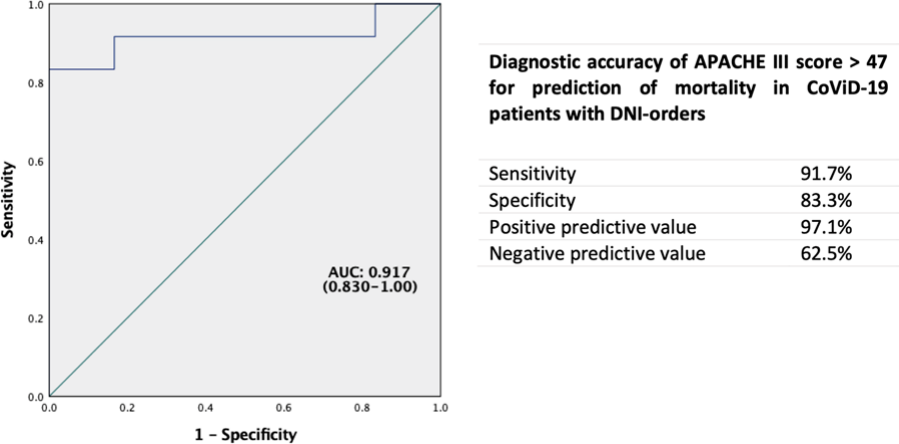
**Fig. 1** **(abstract P141)** ROC curve and diagnostic accuracy of APACHE III for prediction of mortality, applied to COVID-19 patients with DNI orders; x-axis: 1-specificity; y-axis: sensitivity; AUC: area under the curve; 95% CI: 95% confidence interval

## P142

### Safety for health workers of bronchoscopy in critically ill patients with SARS-CoV-2 pneumonia

#### A. Estella

##### Intensive Care Unit University Hospital of Jerez, INiBICA Medicine Department Cadiz University, Intensive & Critical Care, Cadiz, Spain

*Critical Care* 2021, **25**(**Suppl 1**): P142

**Introduction**: Aerosols are only generated by specific medical interventions like bronchoscopy. Initial recommendation in the SARS COV 2 pandemic was to avoid invasive procedures such as bronchoscopy by the risk posed to the operator [1, 2], especially in the first days of ICU admission, that the viral load is postulated is greater.

**Objective**: The aim of the present study is to analyze the risk to the healthcare provider of perform fibrobroncoscopy in mechanically ventilated patients with SARS-CoV-2 pneumonia.

**Methods**: Observational, single-center study. Fiberoptic bronchoscopy was performed in pressure controlled ventilation mode maintaining the optimal PEEP previously established. All broncoscopies were performed by an intensivist, a nurse and a nurse assistant, all of them equipped with individual protective equipment. Time of study was from March 2020 to October 2020. Clinical followup of the healthcare providers was conducted exploring the onset of symptoms such as fever, headache, anosmia. Testing were available widely to symptomatic health-care workers and auxiliary acute health-care staff. In case of clinical suspicion of infection RT-PCR for SARS-CoV-2 were performed.

**Results**: A total of 119 bronchoscopies were performed in 81 mechanically ventilated patients during the time of study. Mean duration of procedure with the airway occupied by the bronchoscope was 2 min and 18 s, performed by a professional with proven experience in this field. Only four healthcare workers developed non specific symptoms in first 48 h after procedure. RT-PCR for SARS-CoV-2 resulted negative in all of them.

**Conclusions**: Exposure to any aerosol-generating procedures with adequate individual protective equipment and minimizing procedure times turned out to be a safe procedure for healthcare workers in mechanically ventilated patients.

**References**

1. Wahidi MM et al. Chest 158:1268–1281, 2020.
2. Gildea TR et al. Cleve Clin J Med. https://doi.org/10.3949/ccjm.87a.ccc054.

## P143

### Awake videolaryngoscopic intubation: a case series

#### F. B. Bongiovanni, E. C. Cagnazzi, N. V. Varanini, S. B. Bonetta, F. R. Rasulo, N. L. Latronico

##### ASST Spedali Civili di Brescia, Anestesia e Rianimazione 2, Brescia, Italy

*Critical Care* 2021, **25**(**Suppl 1**): P143

**Introduction**: Awake videolaryngoscopic intubation (A-VDL) was introduced in our Department at Brescia's Spedali Civili University Hospital in 2019. The aim of this retrospective case series analysis was to further improve and standardize our protocol.

**Methods**: We retrospectively evaluated 24 patients (from January 2019 to June 2021). The Indications for A-VDL were neck/facial anatomical abnormalities, super-obesity or El Ganzouri Risk Index > 6. We collected data about the timing of sedation, drugs used and dosage, level of sedation, procedure success/failure, complications, patient’s explicit memory, and comfort.

**Results**: A-VDL was successful in all the patients: 23 were allocated to A-VDL intubation and 1 to A- videobronchoschopic intubation due to an A-VDL Cormack-Lehane score of 4. We identified 4 procedural times (T0-T3). At T0, all patients received dexmedetomidine (see Table 1) and local anesthesia (LA) followed by a deep pharynx exploration as “topicalization effectiveness test”. At T1, 22 patients had fentanyl, and 14 a subanesthetic dose of ketamine; at T2 local anesthesia was also nebulized directly on the vocal cords/trachea in A-VDL in all patients. At T3, 9 patients had a subanesthetic dose of propofol (before tube passing). The median Ramsey and RASS score were 3 and -1 respectively. The mean time from T0 to intubation was 22 min. When asked, 4 out of 13 patients recalled intubation and 1 out of 14 reported pain, while 3 out of 14 complained of mild discomfort. None required atropine. One patient had a transient oxygen desaturation after successful A-VDL. Patients receiving ketamine became less cooperative.

**Conclusions**: In this case series, A-VDL was successful and well tolerated. Dexmedetomidine at a higher dosage than previously reported (mean 1.48 mcg/kg), together with a low dose of fentanyl, safely granted in this study the desired level of sedation while maintaining spontaneous breathing and cooperation.

**Table:** **Table 1** **(abstract P143)** Results

1	EGRI (median/IQR)	5	3–6
2	ASA (median/IQR)	3	2–3
3	Lidocaine (mean/SD, mg)	245	111
4	Dexmedetomidine (mean/SD, mcg/kg)	1–3	0.5
5	RASS (median/IQR)	− 1	(− 1) − (1 −)
6	Successful laryngoscopy	24	100%
7	Successful IOT	23	95.8%
8	Procedure duration (mean/SD, minutes)	31.9	21.5

## P144

### A quality improvement project to increase the confidence of non-airway trained (NAT) doctors in an intensive care unit (ICU) to manage tracheostomy emergencies through simulation training

#### E. Quek^1^, S. Mandal^2^, M. Carrington^3^

##### ^1^Royal Free Hospital, London, UK; ^2^Thoracic Medicine, Royal Free Hospital, London, UK; ^3^Department of Anaesthesia and Intensive Care, Royal Free Hospital, London, UK

*Critical Care* 2021, **25**(**Suppl 1**): P144

**Introduction**: Airway compromise involving the formation of a tracheostomy is a significant cause of mortality in the ICU [1]. The COVID-19 pandemic saw an increase in NAT doctors re-deployed to ICU, bringing its own challenges. This quality improvement project aimed to assess the impact of simulation training on the confidence of NAT doctors to manage tracheostomy emergencies.

**Methods**: A self-assessment survey and multiple-choice questionnaire (MCQ) were distributed to NAT doctors in a tertiary center ICU to assess their confidence and knowledge in managing a tracheostomy emergency. They were subsequently invited to participate in simulation sessions based around the National Tracheostomy Safety Project algorithm [2]. Management of a tracheostomy emergency up to the point of primary oxygenation was demonstrated on a simulation dummy. Participants then ran through scenarios including blocked and displaced tracheostomy tubes. Trainees completed the same MCQ and survey post-intervention. The aims were to train a minimum of 80% of our NAT doctors in the ICU and increase the average confidence score to manage an emergency to ≥ 3/5 (on a Likert scale 1–5: 1 = not at all confident/familiar, 5 = very confident/familiar).

**Results**: Twenty-one out of 26 NAT doctors participated in a simulation (80.1%). Twenty-two doctors completed the pre-simulation survey, and 16 completed a post-simulation survey. Mean score for familiarity with the algorithm improved from 2.41 (SD = 0.73) pre-simulation to 4.06 (SD = 0.57) post-simulation, p < 0.001. Average confidence scores for managing an emergency improved from 1.86 (SD = 0.47) to 3.81 (SD = 0.75), p < 0.001. MCQ test scores improved post-simulation, from an average score 61.4% (SD = 10.3%) to 87.7 (SD = 8.3%), p < 0.001.

**Conclusions**: These data demonstrate that use of a simulation teaching programme provided an effective learning interface to equip doctors with the skills to manage a tracheostomy emergency.

**References**

1. Thomas AN et al. Anaesthesia 64:358–365, 2009
2. McGrath BA et al. Anaesthesia 67:1025–1041, 2012

## P145

### Non-invasive respiratory support in COVID-19 patients with acute respiratory failure

#### S. Khedher

##### AFH, ICU, Wadi Al-Dawassir, Saudi Arabia

*Critical Care* 2021, **25**(**Suppl 1**): P145

**Introduction**: Non-invasive ventilation (NIV) is currently enumerated as alternative approach in the management of acute respiratory failure in COVID-19 patients. Herein, we report our experience with the use of NIV.

**Methods**: This was a prospective observational study was conducted between April 01, 2020 and April 31, 2021 in our ICU. Patients were monitored clinically and with serial arterial blood gas analysis. NIV was started with initial inspiratory positive airway pressure (IPAP) of 6–8 cm of H2O and was gradually increased to achieve clinical response. The success of NIV, duration of NIV use, hospital mortality, and improvement in clinical parameters were assessed. The failure of success of NIV was defined as a subsequent requirement of invasive ventilation.

**Results**: A total of 64 patients were included. The mean age of the study population was 62 years. 34 were men. Community-acquired pneumonia (83%) and pulmonary edema (17%) were the most common causes of respiratory distress. The ARDS criteria were found in 45 patients. NIV was used in 54 patients (84.3%), NIV with prone in 47.2% and NIV with High Flow Nasal Cannula (HFNC) in 38.9%. The use of NIV was failed in 25 subjects, while 32 subjects required intubation with an average delay of 4 days. The mortality rate was 31% with positive correlation with NIV failure. Pao2/Fio2 ≤ 100 (p = 0.02), comorbidity (p = 0.01) and ICU delay admission ≥ 7 days (p = 0.038) and acute renal injury (0.01), are significantly associated with NIV failure. The use of prone position and HFNC combined with NIV decrease significantly endotracheal intubation.

**Conclusions**: In COVID-19 patients with acute hypoxemic respiratory failure, NIV is feasible can decrease the rate of tracheal intubation and mortality particularly if combined with prone and HNFC.

## P146

### Development of an interactive intubation checklist for use on mobile devices

#### J. M. Dudziak^1^, P. B. Sherren^2^

##### ^1^Anaesthetics, Guy´s & St Thomas´ NHS Foundation Trust, London, UK; ^2^Critical Care Unit, Guy´s & St Thomas´ NHS Foundation Trust, London, UK

*Critical Care* 2021, **25**(**Suppl 1**): P146

**Introduction**: Tracheal intubation in critical care is well recognized as a high-risk procedure, meticulous preparation and effective team working are determinants of good outcome. Checklists aid structured preparation and execution of procedures. We aimed to adapt our unit intubation checklist [1] for use on mobile devices to ensure a consistent safety standard for intubations both on the unit and elsewhere in the hospital.

**Methods**: The unit intubation checklist covers different aspects of pre-procedure preparation, each aspect was assigned one page. Using Affinity Publisher (Serif Europe Ltd., West Bridgford, UK), the different components of each aspect were laid out and supplemented by pictograms, arrows and formatting to aid visual reception. Interactive elements were then added using Adobe Acrobat X Pro (Adobe Inc., San Jose, California, US). We sought feedback from multiple clinicians at different training levels and improved the checklist iteratively in response to their input.

**Results**: The resulting PDF document contains eight pages in a 16:9 format suitable for most modern smartphones (Fig. 1). Viewing is intended in the Acrobat app (Adobe Inc., San Jose, California, US), with one-page display and sideways swiping allowing a view of each page in its entirety and quick progress through the checklist. Each page uses a characteristic background color, with large page numbers in the upper right corner. Seven of the pages contain interactive elements - checkboxes to aid completion of all checklist steps and radio buttons on the airway assessment page to aid identification of high-risk features, and a link to the published guideline on the title page.

**Conclusions**: This interactive mobile checklist has been described as useful and easily adopted in clinical practice from initial feedback. After rolling out this checklist broadly, the impact on checklist uptake and rate of clinical incidents will be the subject of subsequent work.

**Reference**

1. Sherren PB et al. Scand J Trauma Resusc Emerg Med 22:41, 2014.

**Figure:**
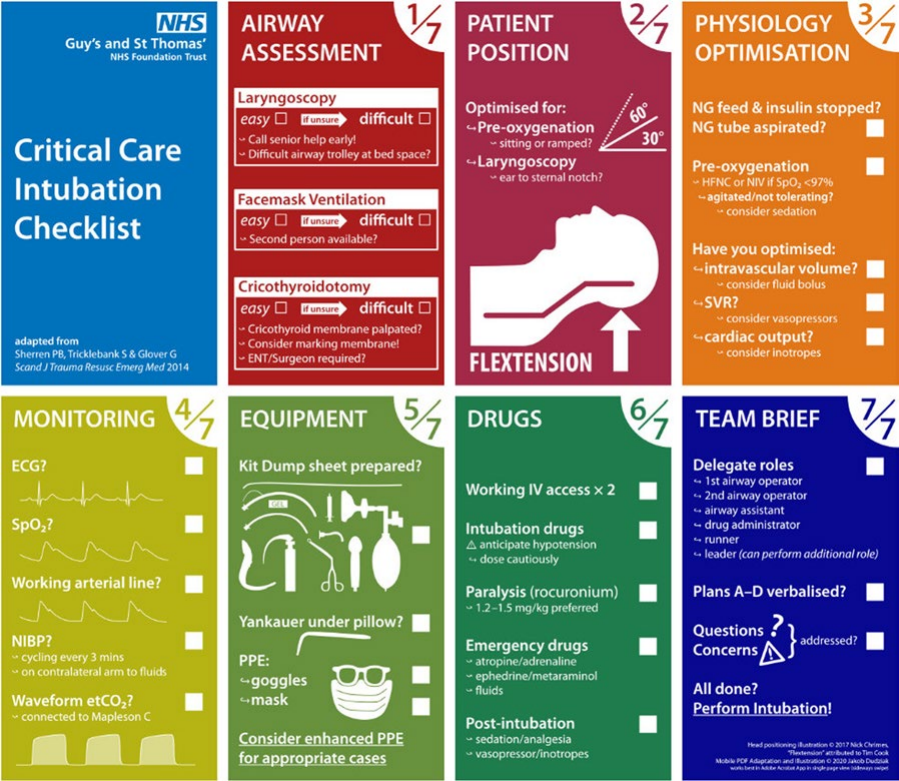
**Fig. 1** **(abstract P146)** Overview of 8-page interactive PDF document optimized for smartphone displays. Each page has a characteristic background color and a large page number in the upper right corner. Most pages contain interactive elements to ensure no aspects of the checklist are missed

## P147

### Evaluation of extubation practice and risk perception at a pediatric cardiorespiratory intensive care unit

#### S. S. Chieng, S. Akunuri, A. Anand, M. Grant, A. Narayanan, A. Chan-Dominy

##### Paediatric Intensive Care Unit, Royal Brompton Hospital, London, UK

*Critical Care* 2021, **25**(**Suppl 1**): P147

**Introduction**: Extubation requires disciplined practice to sustain patient safety during de-escalation from artificial support. We aim to evaluate extubation practice and risk perception around extubation to plan our implementation of extubation care bundle.

**Methods**: Retrospective analysis of electronic notes for procedural documentation and post-extubation complications from two 3-month periods in 2019–2020 was conducted. Pediatric intensive care staff survey on extubation practice was reviewed.

**Results**: Of 114 extubation events for 106 children (median age 6 months, range 1 day-16 years), procedural documentation present in 75% (n = 85) medical and 96% (n = 109) nursing notes. 68% (n = 77) extubations occurred daytime (09:00 h-19:00 h) and 13% (n = 15) night-time (22:00 h-07:00 h). Unplanned extubation rates were 1.7 and 0.9/100 endotracheal tube days for 2 periods, with 2 events of resuscitation for emergent re-intubation. Incidence of post-extubation stridor requiring treatment was 31% (n = 36), and 12% (n = 14) for desaturation over 10% from baseline. 38 extubations transitioned to non-invasive ventilation, of which 4 (11%) escalated to invasive ventilation by 12 h. 48-h freedom from re-intubation was 94% (n = 107). 36 (47% nursing, 53% medical) staff completed questionnaires. Stridor and desaturation were perceived as “often-to-always” events by 16% and 8% staff respectively. Staff rated unorganized or chaotic experience as “often-to-always” related to resource factors (readiness of airway management equipment,71%, or verbalizing failure plan, 37%) or team factors (communication, 74%, role clarity, 69%, or night-time extubation, 26%).

**Conclusions**: Post-extubation complications appeared less infrequent than perceived and risk perceptions are a critical determinant of change in clinical practice. Hence, we are targeting interventions that will engage and change risk perceptions to produce subsequent positive changes in extubation practice.

## P148

### Airway shield: a novel barrier mouthpiece to reduce the risk of aerosol and droplet exposure during endotracheal intubation

#### J. Alonso

##### Intensive Care, The Prince Charles Hospital, Brisbane, Australia

*Critical Care* 2021, **25**(**Suppl 1**): P148

**Introduction**: Endotracheal intubation (ETI) is an aerosol-generating procedure with high risk of clinician exposure and infection by SARS-CoV-2. Protective barrier enclosures have been found to complicate ETI and prolong intubation times, posing a potential threat to patient safety. We designed a novel barrier mouthpiece (Airway Shield) for use during ETI, to reduce risk of clinician exposure to airborne pathogens without complicating the procedure. Our study aims to demonstrate Airway Shield's efficacy in reducing airborne particle exposure in a preclinical model.

**Methods**: We performed a preclinical simulation trial of ETI using a C-Mac videolaryngoscope in manikin models to evaluate laryngoscopist exposure to airborne particles (droplets (> 5 microns) and aerosols (< 5 microns)) with and without 3-D printed prototypes of Airway Shield (Fig. 1). A self-contained clinical simulation lab and a manikin (AirSim, TruCorp®) with droplet and aerosol-generating equipment using normal saline were used for simulation. Airborne particle generation during intubation was measured in 2 clinical scenarios (CPR and high-flow nasal oxygen (HFNO)) during induction, initial laryngoscopy, and endotracheal tube placement. ImageJ software was used to estimate airborne particle dispersion using automatic pixel quantification applied to manually selected polygonal areas. Student's T-test was used to compare averages. A p value of < 0.05 denoted statistical significance.

**Results**: The highest risk of exposure to airborne particles for all scenarios was observed during induction. Overall, Airway Shield demonstrated significant average reduction of airborne particles compared with standard intubation (6108 vs 122,027; p = 0.0149). The greatest reduction in aerosol counts was seen during induction in the CPR scenario (3047 vs 24,001) and during induction in the HFNO scenario (78 vs 37,142) for droplet counts.

**Conclusions**: In a preclinical model, Airway Shield proves effective at reducing the risk of clinician exposure to droplets and aerosols during ETI.

**Figure:**
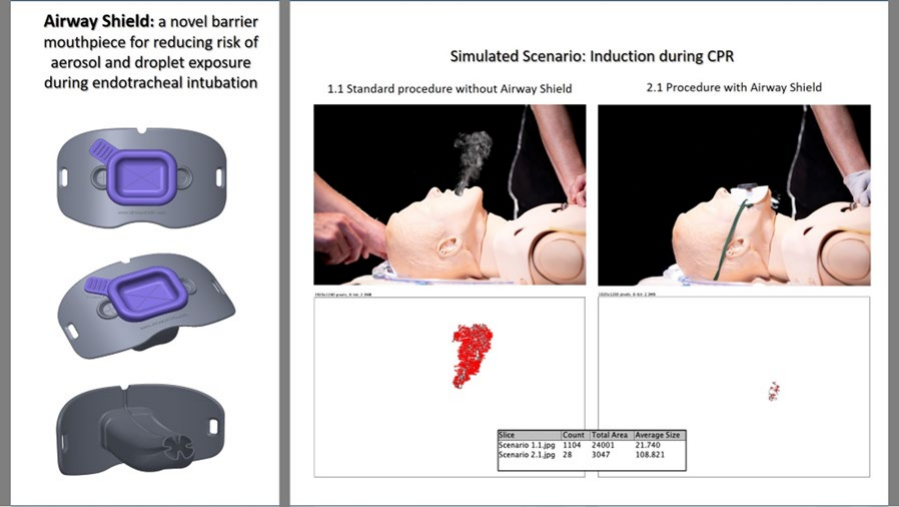
**Fig. 1** **(abstract P148)** Airway Shield: a novel barrier mouthpiece to reduce airborne particle exposure during endotracheal intubation

## P149

### Nebulizer type influences positive end expiratory pressure during high flow nasal therapy in a simulated adult model

#### S. Lally^1^, E. Fernández Fernández^2^, G. Bennett^2^, O. O´Sullivan^2^, L. Reilly^1^, B. Murphy^1^, A. O´Sullivan^1^, M. Joyce^1^, R. MacLoughlin^1^

##### ^1^R&D Science and Emerging Technologies, Aerogen Ltd., Dangan, Galway, Ireland; ^2^Medical Affairs, Aerogen Ltd., Dangan, Galway, Ireland

*Critical Care* 2021, **25**(**Suppl 1**): P149

**Introduction**: High flow nasal therapy (HFNT) is used for oxygen supplementation and the application of positive end expiratory pressure (PEEP) in spontaneous breathing patients. Jet nebulizers (JN), require a driving gas flow to operate and disconnection from the high flow circuit for drug refill. For vibrating mesh nebulizers (VMN), drug is refilled without breaking the circuit and does not require a driving gas flow. The objective of this in-vitro study was to evaluate the impact of nebulizer drug refill on PEEP during HFNT in a simulated adult model.

**Methods**: A VMN (Aerogen Solo, Aerogen, Ireland) or a JN (Cirrus 2, Intersurgical, UK) was connected to a high flow system (O2FLO, Inspired, HK) at 50LPM, with nasal cannula placed on an anatomically correct head model. The head model was connected to a breathing simulator (ASL 5000, Ingmar Medical, USA) set to simulate an adult breathing pattern (Vt 500 ml, 15BPM, and I:E 1:1). The JN was driven with compressed air at 8LPM. This additional gas flow was accounted for in the total high flow gas rates applied. PEEP was recorded using a pressure sensor (Citrex H5, IMT, Switzerland), placed at the level of the lung.

**Results**: During the JN drug refill process, the applied PEEP in the lung drops to near zero (p < 0.0001). This might be explained by the need to break the circuit in order to add drug to the medication cup. On the other hand, the VMN was seen to have no significant effect on PEEP (Table 1) as it remains closed during drug refill (p = 0.84). This could be explained by the VMN design wherein the ventilatory circuit remains intact and drug refill is completed by opening the silicon cap on the medication cup.

**Conclusions**: This study provides key information related to the impact of nebulizer type on PEEP during HFNC in a simulated adult model. VMN technology showed no influence on PEEP when performing drug refill during HFNT.

**Table:** **Table 1** **(abstract P149)** PEEP (cmH_2_O) during HFNT in a simulated adult model

Nebulizer type	Test Scenario	PEEP (cmH_2_O) Average ± SD
VMN	50 LPM HFNT + VMN	4.15 ± 0.05
VMN	During VMN drug refill	4.14 ± 0.03
JN	50 LPM HFNT + JN	4.13 ± 0.04
JN	During JN drug refill	0.01 ± 0.01

## P150

### Success rate in improving oxygenation of COVID-19 patients outside intensive care unit (ICU) using non-invasive ventilation (NIV) in KFAFH, Jeddah

#### F. Almutairi

##### Respiratory Care, King Fahd Armed Forces Hospital, Jeddah, Saudi Arabia

*Critical Care* 2021, **25**(**Suppl 1**): P150

**Introduction**: Non-invasive ventilation (NIV) including continuous positive airway pressure (CPAP) and bi-level positive airway pressure (BiPAP) are the modes of ventilation that applied positive pressure to keep the airways continuously open by using a mask or interface. It is widely use in managing COVID-19 patients in COVID-19 wards and in the intensive care unit (ICU). The main challenges in managing the COVID-19 patients is how to improve oxygenation and to avoid deterioration leading to ICU admission, intubation and sometimes death.

**Methods**: The data were collected precisely using Non-invasive Ventilation Flowsheet and Introduction-Situation-Background-Assessment-Recommendation (ISBAR) handover tool from the month of May 2020 to October 2020. Data collected includes the type of mask use, number of patients with improved oxygenation, number of patients without improvement, diagnosis of patients, comfortability of mask by applying skin barrier and arterial blood gas results.

**Results**: Based on the patient’s data collected for the month of May up to October 2020, 82% of COVID-19 patients who used CPAP therapy outside ICU improved their oxygenation, while about 18% of them showed no signs of improvement then later intubated. On the first three months of the study, success rate in improving oxygenation of patients after using CPAP were between 91–95% (Fig. 1). The success rate eventually drops significantly on month of August and September due to surprising increase of COVID-19 cases which overwhelms the number of RT staff assigned and also the supply of proper mask sizes became scarce. Not long enough, additional RT staff were trained and assigned to COVID-19 patients and arrival of mask supplies late-month of September that causes rebound in success rate to 75% on month of October.

**Conclusions**: Improvement of oxygenation in COVID-19 patients using non-invasive ventilation is critical prior to patient deterioration and requires proper management of trained healthcare personnel and availability of resources.

**Figure:**
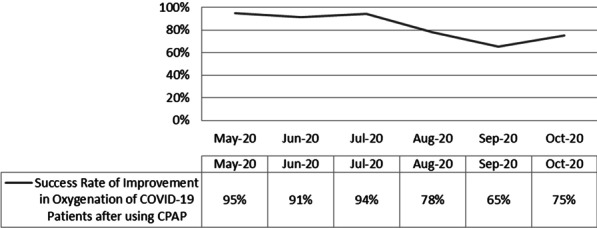
**Fig. 1** **(abstract P150)** Results

## P151

### Percutaneous tracheostomy in patients with COVID-19 supported by ECMO

#### A. L. Shehatta^1^, H. Elmelliti^2^, D. Mutkule^1^, M. Imran^1^, N. Shallik^3^, A. A. Hssain^1^

##### ^1^MICU, Hamad General Hospital, Doha, Qatar; ^2^Emergency Department, Hamad General Hospital, Doha, Qatar; ^3^Anaesthesia, Hamad General Hospital, Doha, Qatar

*Critical Care* 2021, **25**(**Suppl 1**): P151

**Introduction**: Bleeding after percutaneous dilatational tracheostomy (PDT) in patients on ECMO is reported at 40% [1–3]. There is little literature regarding the safety of PDT for patients with COVID-19 on ECMO. The primary objective is to assess the safety of PDT in COVID-19 patients on ECMO. Secondary objectives are complications within 48 h, healthcare workers (HCWs) cross infection.

**Methods**: A single center retrospective review of electronic ICU data of adult patients with confirmed COVID-19 infection supported by ECMO at Hamad General Hospital, Doha-Qatar from 01/03/20 to 01/01/21. The study is approved and individual consent was waived (MRC-01–21-037).

**Results**: A total of 34 patients received ECMO for severe COVID-19.17/34 underwent tracheostomy. One of 17underwent surgical tracheostomy is excluded from final analysis. In 14/16 patients, the median duration of anticoagulant infusion hold prior to PDT was 7 h (IQR 7–15.8). No major complications were observed within 48-h. 12/16 experienced none to mild bleeding; 19% (3/16) experienced moderate bleeding (obvious bleed with up to1 g/dl drop of hemoglobin and/or require at least 1 unit of blood product transfusion); and 6% (1/16) experienced severe bleeding within 48 h of PDT (a drop of 2 + g/dl of hemoglobin; needed 2 or > transfusion units or surgical intervention). 4/16 of the COVID-19 patients had a positive PCR test result on the day of PDT. Each PDT involved 3 HCWs: an operator, a respiratory therapist and an ECMO nurse specialist. None of the HCWs involved tested + ve for the virus within 2 weeks post-PDT.

**Conclusions**: 25% of patients suffered moderate to severe non-life-threatening bleeding. None of ICU involved HCWs suffered COVID-19 transmission. Experienced operator, minimal trauma and strict adherence to PPE are essential. Extended research including larger sample size may further support the safety of PDT in COVID-19 patient ECMO.

**References**

1. Braune S et al. Intensive Care Med 39:1792–9, 2013
2. Kruit N et al. J Cardiothorac Vasc Anesth 32:1162–6, 2018
3. Salna M et al. ASAIO J 66:652–6, 2020

## P152

### Evaluating and comparing tracheostomy practice during the first wave of the COVID-19 pandemic with pre-pandemic practice, in the South East of Scotland

#### A. J. Thomson^1^, T. H. Craven^2^, M. J. Blackstock^1^

##### ^1^Critical Care Department, Western General Hospital, Edinburgh, UK; ^2^Critical Care Department, Royal Infirmary of Edinburgh, Edinburgh, UK

*Critical Care* 2021, **25**(**Suppl 1**): P152

**Introduction**: Performing a tracheostomy during the COVID-19 pandemic raised a number of concerns, and guidance was limited and conflicting from the onset [1]. This study aims to investigate whether significant change in our tracheostomy practice occurred during the first wave of the COVID-19 pandemic.

**Methods**: We undertook a service evaluation from March—May 2020, assessing tracheostomy practice during the COVID-19 pandemic within five adult intensive care units (ICUs) across the South East of Scotland. We included patients with confirmed COVID-19 infection, receiving mechanical ventilatory support that required tracheostomy insertion. We compared this to a cohort treated within one of the ICUs prior to the pandemic, from 2015–19. Patients were included if treated for ARDS secondary to bacterial, viral or aspiration pneumonitis, requiring mechanical ventilation and tracheostomy insertion.

**Results**: Tracheostomy insertion was performed in 25 (28.1%) of 89 mechanically ventilated COVID-19 positive patients. Within the pre-COVID-19 cohort 19 patients, who met the inclusion criteria, required a tracheostomy insertion. Result of the comparison data for both cohorts is shown in Fig. 1.

**Conclusions**: Overall, despite the limited and conflicting guidance, we were reassured that our practice had not deviated from standard care due to fears around aerosolisation and risk of staff transmission. The results show a decreased age and a higher positive end-expiratory pressure (PEEP) at the time of tracheostomy in the COVID-19 cohort, suggesting that tracheostomies can be safely performed at PEEP values higher than 5.

**Reference**

1. Queen Elizabeth Hospital Birmingham COVID-19 airway team. Br J Anaesth 125: 872–79, 2020

**Figure:**
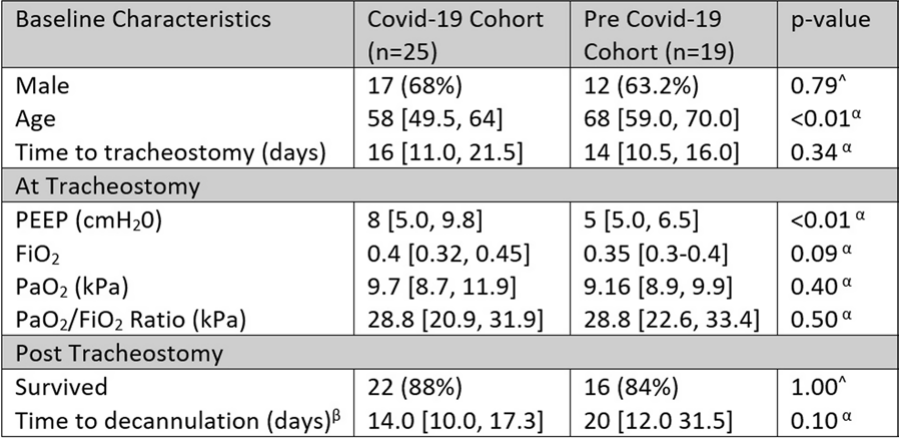
**Fig. 1** **(abstract P152)** Tracheostomy practice in a COVID-19 cohort in comparison to a pre-COVID-19 cohort. All values are median [IQR] unless otherwise stated. Fishers exact(^), Mann–Whitney(α), Survivors only(β)

## P153

### Clinical characteristics and outcome of tracheostomized patients with COVID-19 supported by ECMO

#### A. L. Shehatta^1^, D. Mutkule^1^, M. Imran^1^, H. Elmelliti^2^, N. Shallik^3^, A. A. Hssain^1^

##### ^1^MICU, Hamad General Hospital, Doha, Qatar; ^2^Emergency Department, Hamad General Hospital, Doha, Qatar; ^3^Anesthesia, Hamad General Hospital, Doha, Qatar

*Critical Care* 2021, **25**(**Suppl 1**): P153

**Introduction**: To describe the demographics, clinical characteristics, severity of illness and outcome of confirmed severe COVID-19 infection requiring tracheostomy whilst on venovenous ECMO support. Percutaneous dilatational tracheostomy (PDT) is performed to aid sedation and mechanical ventilation management and to allow mobilization and rehabilitation. There is no specific or agreed recommendations on the timing of PDT [1, 2]. Outcome of ECMO-supported COVID-19 tracheostomized patients is not adequately reported.

**Methods**: A single center retrospective review of electronic ICU data of adult patients with confirmed COVID-19 infection supported by VVECMO at Hamad General Hospital in Doha, Qatar from 01/03/2020 to 01/01/2021. The study is approved by institutional research board and individual consent was waived (MRC-01–21-037).

**Results**: Thirty-four patients received ECMO for severe COVID-19. 17/34 (50%), all supported by VVECMO, underwent tracheostomy. The majority 16/17 were performed bedside by percutaneous technique and one (1/17) underwent surgical tracheostomy and is excluded from final analysis. Table 1 presents demographics and severity of illness. The prevalence of hypertension, diabetes, coronary artery disease was 19%, 19% and 13% respectively. None of the patients was known to have heart failure, stroke or chronic kidney disease. The 30- and 90-day survival was 69% and 50%, respectively, which is similar to international findings [3].

**Conclusions**: Adult patients with confirmand COVID-19 undergoing percutaneous tracheostomy on ECMO were younger and had fewer comorbidities. They had high severity of illness score on admission and only half of them survived to 90 days. A larger sample size is needed to reach firm conclusions.

**References**

1. Kruit N et al. J Cardiothorac Vasc Anesth 32:1162–6, 2018
2. Salna M et al. ASAIO J 66:652–6, 2020
3. https://www.elso.org/Registry/FullCOVID19RegistryDashboard.aspx Accessed 31/5/21

**Table:** **Table 1** **(abstract P153)** Demographics, duration of intubation, days on ECMO before tracheostomy and severity of illness scores of all patients (n = 16)

	**Median**	**25th centile**	**75th centile**
Age (years)	48	42.8	57.8
BMI	25.7	24.2	26.8
Duration of intubation (days)	25	21	32
ECMO-to-tracheostomy (days)	21.5	17.8	26.3
APACHE2	26.5	21	30
SOFA on admission	12	11	12
SOFA on PDT day	9	6.75	10.5

## P154

### Hyperbaric oxygen therapy in patients with COVID-19

#### A. Evseev^1^, O. Levina^1^, A. Shabanov^2^, I. Goroncharovskaya^1^, A. Kuzovlev^2^, V. Kulabukhov^1^, S. Petrikov^1^

##### ^1^ N.V. Sklifosofsky Research Institute of Emergency Medicine, Moscow, Russian Federation; ^2^Federal Research and Clinical Center of Intensive Care Medicine and Rehabilitology, Moscow, Russian Federation

*Critical Care* 2021, **25**(**Suppl 1**): P154

**Introduction**: The research in COVID-19 is focused on finding methods aimed not only at eliminating hypoxia - one of the main complications of COVID-19, but also capable of reducing the risk of transferring a patient to mechanical ventilation. A combination of these characteristics is possessed by hyperbaric oxygenation (HBO) [1–3].

**Methods**: We examined 60 patients diagnosed with a new coronavirus infection caused by the SARS-CoV-2 virus. The control group consisted of 30 patients (13 men, 17 women, 64.5 ± 12.7 y.o.), study group - 30 patients undergoing HBO (14 men, 16 women, 61.5 ± 14.5 y.o.). HBO sessions were carried out in a Sechrist 2800 machine (USA) in the 1.4–1.6 ATA mode for 40–60 min. In total patients received 153 HBO sessions (5.1 ± 2.5 sessions per patient).

**Results**: Depending on the severity of the condition SpO_2_ values could reach 95% after 1–2 sessions in moderately severe patients and after 5–6 sessions in patients in severe condition. This circumstance made it possible in most cases to refuse additional oxygen therapy during the HBO course or within 1–2 days after its completion (Fig. 1). In no cases it was necessary to put patient to mechanical ventilation. In addition, against the background of HBO, the normalization of the redox state (malonic dialdehyde concentration, blood serum total antioxidant activity, platinum electrode open circuit potential in blood serum) was noted [4]. There was a significant difference in the NEWS2 scale: 4.7 ± 2.3 and 4.0 ± 2.3 points on days 4 and 10 in the control group, 4.3 ± 2.2 and 1.2 ± 1.7 points before and after the HBO course in the study group.

**Conclusions**: HBO can be an important addition to the complex treatment in patients with COVID-19 by eliminating hypoxia, reducing the risk of switching to mechanical ventilation and significantly improving patient condition.

**References**

1. Guo D et al. Undersea Hyperb Med 47:181–187, 2020
2. Thibodeaux K et al. J Wound Care. 29:S4–S8, 2020
3. Gorenstein SA et al. Undersea Hyperb Med 47:405–413, 2020
4. Petrikov SS et al. General Reanimatology 16:4–18, 2020

**Figure:**
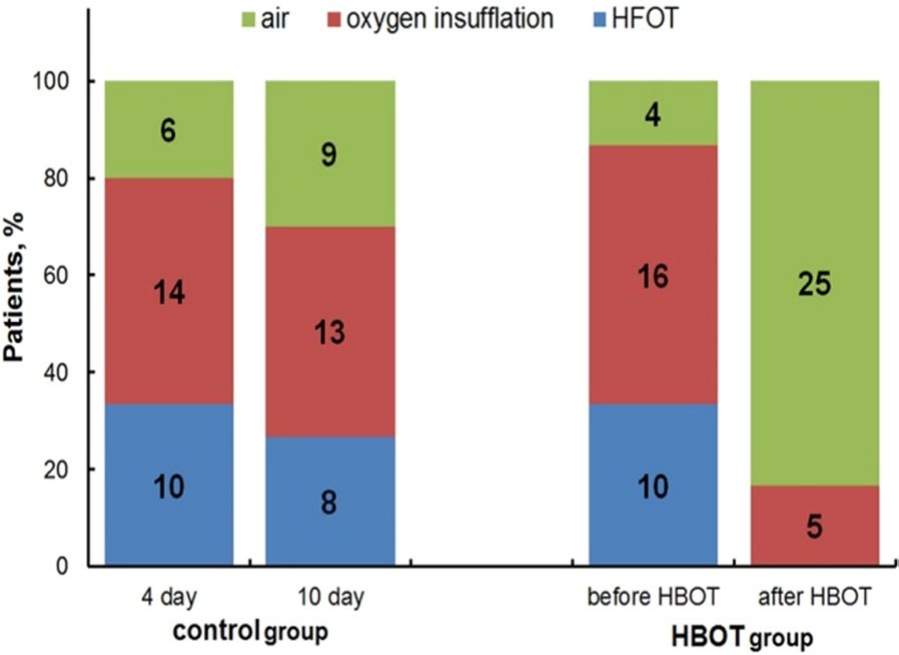
**Fig. 1** **(abstract P154)** Need for oxygen support in patients with COVID-19. Oxygen insufflation - 3–6 l/min, HFOT (high flow oxygen therapy) - up to 60 l/min

## P155

### Prolongation of high flow nasal cannula therapy in COVID-19, an observational cohort study

#### J. Volcke^1^, A. Van Hoorn^2^, M. Moretti^3^, M. Mekeirele^1^, S. De Bontridder^4^, J. Jonckheer^5^, J. Poelaert^2^

##### ^1^Intensive Care, University Hospital Brussels (UZB), Jette, Belgium; ^2^Intensive Care - Anesthesiology and Perioperative Medicine, University Hospital Brussels (UZB), Jette, Belgium; ^3^Internal Medicine and Infectious Disease, University Hospital Brussels (UZB), Jette, Belgium; ^4^Respiratory Medicine, University Hospital Brussels (UZB), Jette, Belgium; ^5^Intensive Care - Respiratory Medicine, University Hospital Brussels (UZB), Jette, Belgium

*Critical Care* 2021, **25**(**Suppl 1**): P155

**Introduction**: Optimal timing of invasive mechanical ventilation (MV) in severe acute respiratory distress syndrome (ARDS) due to coronavirus disease 2019 (CoViD-19) is unclear. The primary objective of this study was to explore the role of prolonged high flow nasal cannula (HFNC) oxygen therapy on mechanical ventilation and mortality in patients with ARDS caused by CoViD-19. Furthermore, predictors for the need for mechanical ventilation and mortality were investigated.

**Methods**: A retrospective observational study was conducted at a mixed tertiary ICU in Brussels. Medical records were reviewed of all consecutive CoViD-19 patients presenting with acute hypoxic respiratory failure treated with HFNC oxygen therapy between March 6th, 2020, and January 1st, 2021. Delayed intubation or prolongation of HFNC therapy was defined as the number of days of HFNC from the point when a respiratory rate oxygen index (ROXi, defined as the ratio of SpO2/FiO2 to respiratory rate) < 3.85 was reported.

**Results**: One hundred and twenty patients received HFNC therapy, and fifty-four (45%) were successfully weaned. Thirty-six (66%) of them reached the HFNC prolongation criteria. Seventy-five patients underwent MV with 40% mortality. Higher Apache III and SAPS 2 scores were predictive factors for mortality, while prolonged HFNC treatment was not. Prolongation of HFNC therapy was a negative predictor for MV after adjustment for age, sex, and comorbidities.

**Conclusions**: In the current cohort, prolongation of HFNC therapy and subsequently delayed intubation resulted in a lower rate of MV and was not associated with higher mortality. APACHE III and SAPS2 at admission were early predictors for MV and mortality.

## P156

### Risk factors associated with mortality during the first prone cycle in patients with ARDS secondary to SARS-CoV-2 pneumonia

#### M. C. Gonzalez^1^, G. Musso^1^, J. M. Dominguez^1^, S. Calabrono^1^, A. Lopipi^1^, G. Appendino^1^, M. Manago^1^, M. Manago^1^, E. Estenssoro^2^, C. Lovesio^1^

##### ^1^Intensive Care, Sanatorio Parque, Rosario, Argentina; ^2^Intensive Care, Hospital Interzonal de Agudos San Martin de La Plata, La Plata, Argentina

*Critical Care* 2021, **25**(**Suppl 1**): P156

**Introduction**: Our objective was to identify factors associated with mortality after the first 24 h of prone position in patients on mechanical ventilation due to moderate and severe ARDS caused by SARS- CoV-2.

**Methods**: This was a retrospective cohort study. We included all patients with ARDS causedby SARS-CoV-2 pneumonia and required prone. We registered sex, age, APACHE II, SOFA, number of prone sessions—which lasted 24 h-, hospital-acquiredinfections and hospital mortality. PaO_2_/FiO_2_, plateau pressure and driving pressurewere recorded and compared before and after the first prone session.

**Results**: A total of 126 patients were included. Mean age was 60 ± 15 years, 25% were women. APACHE II was 12 ± 2. Baseline PaO2/FiO2 112 ± 26 mmHg with no differences between survivors and non-survivors (p = 0.92) and after prone it was 186 ± 59 (p < 0.000). Survivors had 220 ± 48 mmHg versus 169 ± 57 in non-survivors (p < 0.000).One prone session was required by 67 patients, 2 sessions by 26 patients, 3 by 23 patients and 4 by 10 patients. Mortality was 55%, 69%, 75% and 100% respectively; p < 0.000). 117 patients increased PaO_2_/FiO_2_. In 21 patients (16%) it was less than 25%; 28 patients (22%) showed a rise between 25- 50%; and in 68 patients (54%) more than 50%. Mortality was 90%, 68% and 53% respectively (p = 0.012). 52 patients had suspected intra-hospital infection. 10 survived (20%) and 42 died (80%) (p < 0.001). After multivariate analysis, factors independently associated with mortality were % of increase in PaO_2_/FiO_2_ after 24 h in prone (OR 0.99; CI 95% 0.98–0.99; p = 0.01) the number of prone cycles (OR 1.84; CI 95% 1.08–3.12; p = 0.02) and presence of in-hospital infection (OR 5.8; CI 95% 1.78–2; p = 0.001).

**Conclusions**: In this cohort of patients with ARDS secondary to SARS-CoV-2 pneumonia, the increase in PaO_2_/FiO_2_ ratio after 24 h of prone position was independently associated with decreased mortality; while number of prone cycles, and presence of hospital-acquired infection were associated with increased mortality.

**Figure:**
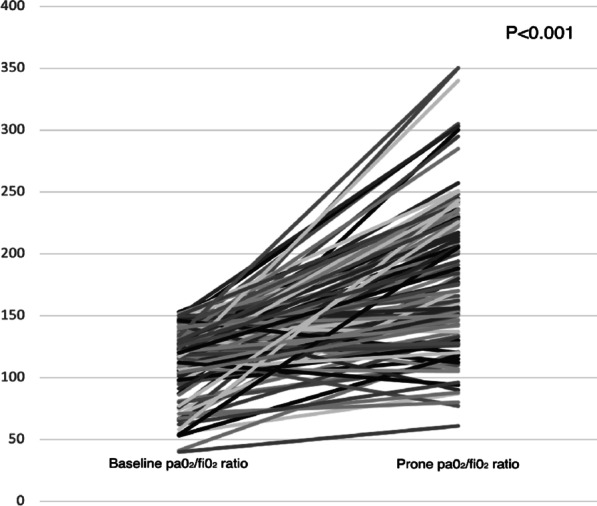
**Fig. 1** **(abstract P156)** Before and after prone PaO_2_/FiO_2_

## P157

### HFNC in patients with severe respiratory infection due to SARS-CoV2: usefulness of the ROX index

#### J. F. Martínez carmona, E. Lopez Luque, F. A. Hijano Muñoz, M. J. Delgado Amaya, J. M. Mora Ordoñez

##### Intensive Care Unit, HRU Málaga, Málaga, Spain

*Critical Care* 2021, **25**(**Suppl 1**): P157

**Introduction**: One of the devices most used in patients with severe respiratory infection caused by SARS-Cov2 has been HFNC (high flow nasal cannula), with the aim of preventing progression to IMV. It is important to have scores that allow predicting the success or failure of this therapy, avoiding unnecessary delays in the initiation of IMV.

**Methods**: To assess the prognostic capacity of the ROX Index in patients with severe respiratory infection due to SARS-Cov2 with HFNC. We evaluated 20 patients admitted to the ICU for severe respiratory infection with CRP + SARS-CoV-2 who required respiratory support on admission with HFNC. The following variables were collected: demographic data, ROX index at admission, 12 h, 24 h, 36, 48, 60 and 72 h, duration of NFNC, need for invasive mechanical ventilation, stay in ICU, mortality in ICU.

**Results**: The mean age was 63.9 years ± 11.5. 85% males. The median time of HFNC was 4.5 days. The mean stay in the ICU was 12 days ± 6.9. 65% required invasive mechanical ventilation. Mortality was 50%. Patients who required invasive mechanical ventilation had lower ROX index values in the first 48 h compared to the group that did not require MV, however, we did not find a statistically significant association (95% CI p > 0.05). The patients who required mechanical ventilation presented higher mortality with significant differences (95% CI p = 0.029). We did not observe differences in mortality associated with the duration of previous HFNC in patients who required IMV.

**Conclusions**: We observed an adequate correlation between the ROX index and HFNC failure in patients with severe respiratory infection due to SARS-CoV-2.

## P158

### Dose-dependent effect of diaphragm neurostimulation on GFAP serum concentrations in pigs mechanically ventilated for 50 h

#### T. Bassi^1^, E. Rohrs^2^, K. Fernandez^2^, M. Nicholas^2^, M. Ornowska^2^, M. Gani^3^, D Evans^3^, S. Reynolds^2^

##### ^1^Physiology, Simon Fraser University, Burnaby, Canada; ^2^Simon Fraser University, Burnaby, Canada; ^3^Lungpacer Medical Inc., Exton, USA

*Critical Care* 2021, **25**(**Suppl 1**): P158

**Introduction**: Preclinical studies have demonstrated that mechanical ventilation (MV) is associated with brain injury. Glial fibrillary acid protein (GFAP) is an accepted serological marker for brain injury. We evaluated whether diaphragm neurostimulation in synchrony with lung-protective MV for 50 h, in a normal-lung porcine model, would affect GFAP serum concentrations in comparison with never-ventilated and mechanically ventilated pigs.

**Methods**: Twenty-eight healthy juvenile pigs with non-injured lungs were divided into four groups: MV only (MV group, n = 8), MV in association with diaphragm neurostimulation delivered every other breath (TTDN50% + MV group, n = 7), MV in association with diaphragm neurostimulation delivered every breath (TTDN100% + MV group, n = 7) and never ventilated (NV group, n = 6). A central line catheter with embedded electrodes was inserted into the left subclavian vein for the TTDN50% + MV and TTDN100% + MV subjects, and the diaphragm was activated by temporary transvenous diaphragm neurostimulation (TTDN), targeting a reduction in pressure–time product between 15 and 20%. Volume control MV was set to achieve and maintain a tidal volume of 8 ml/kg with a PEEP of 5 cmH_2_O. Blood samples were collected at the end of the experiment, to analyze GFAP serum concentrations. Data are expressed as median and interquartile ranges. The Kruskal–Wallis test and Dunn’s multiple comparison test were used for statistical analysis. P values < 0.05 were considered statistically significant.

**Results**: GFAP serum concentrations found were: 0.40 ng/ml (0.28–0.57) for the MV group, 0.29 ng/ml (0.25–0.32) for the TTDN50% + MV group, 0.04 ng/ml (0.02–0.06) for the TTDN100% + MV group, and 0.15 ng/ml (0.07–0.23) for the NV group, with statistically significant differences between groups, as determined by the Kruskal–Wallis test, p < 0.0001.

**Conclusions**: In a porcine model, TTDN + MV for 50 h resulted in lower GFAP serum concentrations in comparison with the MV group, with a dose-dependent effect.

**Figure:**
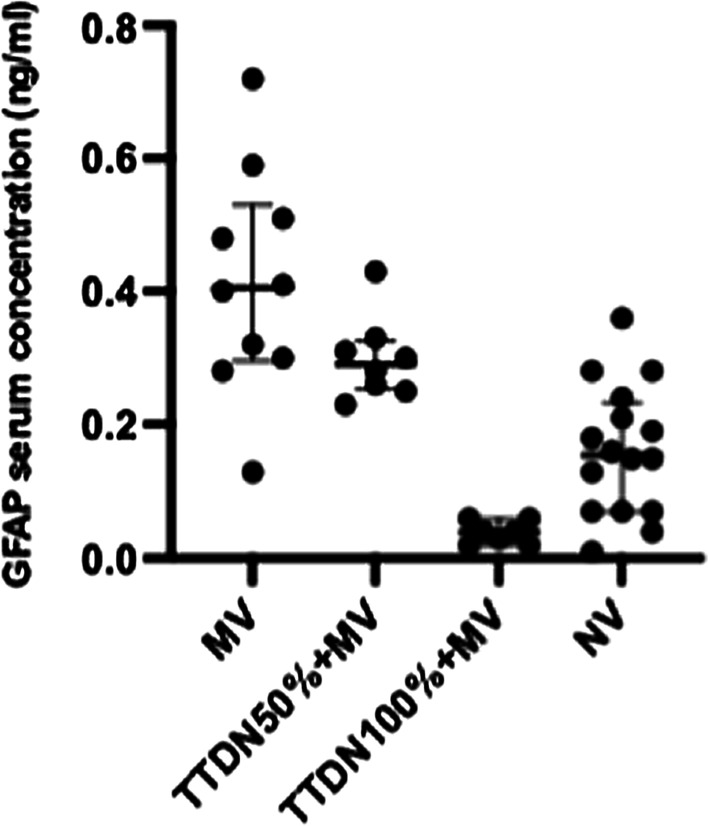
**Fig. 1** **(abstract P158)** Dot plot showing the GFAP concentrations found in the serum: 0.40 ng/ml (0.28–0.57) for the MV group, 0.29 ng/ml (0.25–0.32) for the TTDN50% + MV group, 0.04 ng/ml (0.02–0.06) for the TTDN100% + MV group, and 0.15 ng/ml (0.07–0.23) for the NV group, with statistically significant differences between groups, as determined by the Kruskal–Wallis test, p < 0.0001. Post-hoc analysis using Dunn’s multiple comparison test showed considerable statistical differences in serum GFAP concentrations between the MV and TTDN100% + MV groups (0.40 vs. 0.04, p < 0.0001), the MV and NV groups (0.40 vs. 0.15, p = 0.0170), and the TTDN50% + MV and TTDN100% + MV groups (0.29 vs. 0.04, p = 0.0008)

## P159

### Predictors of poor outcome in patients admitted to intensive care following failed high-flow nasal oxygen therapy

#### R. R. Best, R. Docking

##### Critical Care, Queen Elizabeth University Hospital, Glasgow, UK

*Critical Care* 2021, **25**(**Suppl 1**): P159

**Introduction**: We investigated whether there were any predictors of poor outcome amongst patients who were admitted to the intensive care unit (ICU) following treatment with high-flow nasal oxygen (HFNO) in Medical HDU (MHDU) at a tertiary hospital in Glasgow. Delayed intubation as a result of initial treatment with HFNO has been shown to worsen ICU outcomes, including mortality, extubation success and length of ventilation [1]. Identification of factors that predict poor ICU outcome during HFNO therapy could aid decision-making regarding escalation and timing of intubation to avoid delayed intubation.

**Methods**: Between January 2016 and January 2019, there were 106 patients admitted to MHDU who were treated with HFNO, deteriorated and were admitted to ICU. Data were collected from the patient record, including demographics and physiological parameters. Severity of respiratory failure was calculated using the ROX index [2], measured after one hour of HNFO therapy and immediately prior to ICU admission. Chi-square tests were conducted to assess whether demographics, length of HFNO therapy or ROX index were associated with poor ICU outcome.

**Results**: The majority (62%) of patients were male and median age was 59 years. Median MHDU length of stay was 0.79 days (IQR 0.34–1.91) and patients received a median of 12 h HFNO (IQR 5–29.5 h) prior to admission to ICU. Once admitted to ICU, 85.8% received invasive ventilation. Hospital mortality was 43.4%. In a regression analysis, only age and quartile of ROX index at ICU admission were significantly associated with ICU outcome, with p values of 0.009 and 0.03 respectively.

**Conclusions**: Age and low ROX index on admission to ICU are predictors of poor outcome in patients admitted to MHDU receiving HFNO.

**References**

1. Kang BJ et al. Intensive Care Med 41:623–632, 2015.
2. Roca O et al. J Crit Care 35:200–205, 2016.

## P160

### Qualitative evaluation of different mechanical lung ventilation methods

#### J. Krauklyte, E. Januskeviciute, S. Vosylius

##### Institute of Clinical Medicine, Department of Anesthesiology and Intensive Care, Vilnius University Faculty of Medicine, Vilnius, Lithuania

*Critical Care* 2021, **25**(**Suppl 1**): P160

**Introduction**: It is known that ventilator related mechanical power (MP) can cause ventilator-induced lung injury (VILI), especially if chosen method and settings of ventilation are inadequate. The aim of this study was to perform qualitative analysis of various mechanical lung ventilation modes by composing and applying a methodology for processing and evaluating large data sets.

**Methods**: Data collected during one of the phases of the research conducted at the Republican Vilnius University Hospital in the period of 2020–2022 were analyzed. Mechanical lung ventilation data were obtained from Hamilton S1 ventilator database. Criteria for rejecting false ventilation cycles were defined. Assessment of MP, driving pressure and inspiratory pressure was performed for qualitative evaluation of different ventilation modes. Calculation of MP was carried out according to formulas described in the literature.

**Results**: Mechanical lung ventilation data of 8 patients were collected during the study period (6 were ventilated with adaptive support ventilation mode, 2 – pressure controlled ventilation mode). A total of 241,143 ventilation cycles were analyzed, 9309 (3.86%) of which met false criteria. Average MP and driving pressure values ranged from 8.69 J/min to 29.34 J/min and from 4.69 cmH_2_O to 14.69 cmH_2_O accordingly. Proportion of detected ventilation cycles in which driving pressure exceeded 15 cmH_2_O ranged from 0% to 24.89%. Estimated inspiratory pressure values varied from 5 cmH_2_O to 15 cmH_2_O.

**Conclusions**: Criteria reflecting false mechanical lung ventilation cycles were developed. Calculated MP generated using different mechanical lung ventilation modes raged from 8.69 J/min to 29.34 J/min. Episodes of ventilation cycles that exceeded limits of safe mechanical lung ventilation strategy, observed in majority of patients, were not associated with increased risk of VILI.

## P161

### Preventing accidental heat and moisture exchanger obstruction with the humidicare

#### J. Richardson, H. Altemimi, M. Blunt, P. Young

##### ITU, Queen Elizabeth Hospital, King´s Lynn, UK

*Critical Care* 2021, **25**(**Suppl 1**): P161

**Introduction**: The accidental use of a heat and moisture exchanger (HME) in a heated and humidified ventilatory circuit causes sudden and catastrophic obstruction of the HME in hours. Water saturates the filter and blockage is usually sudden with average time to occlusion between twelve and twenty four hours [1]. An NHS safety alert (2015) identified 76 reported incidents in England [2]. The Humidicare (Medovate, Cambridge, UK) warns of an impending problem by reacting to the heated humidifier (HH) with a color change and caution message. Removal of the Humidicare is prompted before waterlogging occurs. We evaluated device ability to deliver. Warning within the relevant timeframe and to ensure immunity to heat and humidity from a simulated patient.

**Methods**: We performed a randomized study in a simulated ICU environment using a Maquet ventilator and mechanical lung. Time to Humidicare warning delivery in a HH circuit (n = 10) was compared to behaviour in a control circuit without HH (n = 10). Fisher & Paykel HHs were used. A secondary HH on the lung side emulated patient behaviour to demonstrate no confounding factors.

**Results**: All HMEs activated in HH arm. Color change started after a mean of 31.4 s ± 2.76 s (95% CI). The warning message was fully developed after a mean of 93.6 s ± 8.09 s (95% CI). No HMEs were triggered in the control arm after five minutes.

**Conclusions**: The Humidicare HME seems to reliably indicate the problem in a timely manner. It does not register false positive with the heat and humidity coming off a patient equivalent. Occasional errors are hard to eliminate with traditional information dissemination techniques and training; improved response is achieved with engineered solutions [3]. The Humidicare protects staff and patients by helping comply with NHS safety standards to prevent errors.

**COI**: Young: share of patent ownership.

**References**

1. Doyle A et al. J Crit Care 30:863, 2015
2. (NHS/PSA/W/2015/012)
3. Patel V et al. J Crit Care 47:159–163, 2018

## P162

### Silent hypoxemia in critical COVID-19 patients: prevalence, risk factors and importance of prolonged disease course before ICU admission

#### V. Vicka^1^, E. Januskeviciute^1^, J. Krauklyte^1^, J. Krauklyte^1^, A. Aleknaviciene^1^, G. Patapaviciute^1^, D. Ringaitiene^1^, L. Jancoriene^2^, J. Sipylaite^1^

##### ^1^Department of Anesthesiology and Intensive Care, Vilnius University, Vilnius, Lithuania; ^2^Department of Infectious Diseases, Vilnius University, Vilnius, Lithuania

*Critical Care* 2021, **25**(**Suppl 1**): P162

**Introduction**: Silent hypoxemia (SH) is defined as severe hypoxemia without an increase in respiratory effort. This phenomenon is reported as highly prevalent in COVID-19 patients. The aim of this study was to determine the prevalence and the risk factors of SH in COVID-19 ICU population.

**Methods**: This was a retrospective analysis of institutional database of COVID-19 patients treated in the ICU during the year of 2020. The SH was defined as a respiratory rate below 20 bpm and by two groups of PaO_2_/FiO_2_ ratio: ≤ 300 to 200 (indicating mild ARDS) and ≤ 200 (indicating moderate to severe ARDS) upon admission to the ICU; the latter was selected for further analysis. Demographic data, co-morbidities and other variables were entered into the regression analysis of SH risk factors. Statistical analysis was carried out with SPSS IBM v24.

**Results**: A total of 228 patients were included in the study. 62.3% were women (n = 142), mean age of 61.52 (SD = 13.5), and APACHE II score of 13.42 (SD = 7.21). The prevalence of SH was 18.9% (n = 43) in mild ARDS group and 11.8% (n = 27) in severe to moderate ARDS group. Univariate regression analysis revealed count of days before admission to ICU (Exp(B) = 1.09 CI 95: 1.02–1.17, p = 0.013) and lymphocyte count (Exp(B) = 1.29 CI95: 1.01–1.67, p = 0.046) as risk factors of SH. Multivariate regression model of these determinants revealed count of days before admission to ICU (Exp(B) = 1.09 CI95: 1.01–1.17, p = 0.025) as an independent prognostic factor of SH. Further analysis of days before admission to ICU showed median of 12 [7.8–14.3] for SH group and median of 7.5 [4–11] for non-SH group (p = 0.003), overall group – 8 [4–11].

**Conclusions**: SH is prevalent in COVID-19 patients and is dependent on the definition criteria. Prolonged disease course before ICU admission is an independent risk factor of SH.

## P163

### Performance of automatic endotracheal cuff management device

#### U. Borg, K. Bull

##### Medtronic, Boulder, USA

*Critical Care* 2021, **25**(**Suppl 1**): P163

**Introduction**: Effective control of endotracheal tube (ETT) cuff pressure (CP) is vital to airway management in intubated patients. CP must be kept within a therapeutic range to ensure adequate cuff seal and delivery of mechanical ventilation, while preventing against complications such as ischemia, aspiration, and stenosis [1]. The purpose of this study was to test the CP management device PBACC (Puritan Bennett Automatic Cuff Controller, Medtronic, Boulder, USA) and evaluate maintenance of cuff seal in an animal model during mechanical ventilation.

**Methods**: Eight animals with tracheal sizes suitable for 5.0 to 8.0 mm inner diameter ETTs were intubated. The cuff was inflated to a pressure of 25–30 cmH2O, as recommended in instructions for use. Contrast media was applied above the cuff. CP was maintained using the PBACC and compared to a reference pressure transducer. Animals were in supine position for 60 min, followed by lateral position for 60 min. Supine position was assumed again for 30 min, followed by opposite lateral position for 60 min. Fluoroscopy was obtained five minutes after position change and at the end of each position period. Images were examined for dye in the trachea below the cuff.

**Results**: There were no statistical differences between the PBACC pressures and the reference. No cuff leaks were detected on fluoroscopy.

**Conclusions**: CP may change due to change in position, slow cuff leaks and other causes, making routine monitoring of CP necessary to maintain correct pressure. Automated cuff management devices have been suggested to remedy this situation. The PBACC maintained CP during all position changes resulting in no breach of cuff seal. These results may indicate that the PBACC device is suitable for continuous CP management. Longer-term clinical studies are needed to demonstrate its clinical utility.

**Reference**

1. Sole M et al. Am J Crit Care 18:133–143, 2009.

**Table:** **Table 1** **(abstract P163)** Cuff pressures versus reference pressures

	**5 mm tube**		**6 mm tube**		**7 mm tube**		**8 mm tube**	
Animal number	1	2	3	4	5	6	7	8
Mean PBACC pressure (cmH_2_O)	24.9	25.1	24.9	25.1	25.0	25.4	25.1	25.2
STD	0.62	0.86	0.80	0.56	0.68	0.74	0.68	0.67
Mean reference pressure (cmH_2_O)	24.9	27.3	26.7	26.2	27.5	27.6	26.8	27.6
STD	0.89	1.14	1.13	1.78	1.22	1.19	1.69	1.11
Mean difference (cmH_2_O)	0.0	-2.2	-1.8	-1.1	-2.5	-2.2	-1.6	-2.4
STD	0.73	0.96	0.99	1.68	1.10	1.03	1.53	0.83

## P164

### Investigation of the correlation between perioperative oxygen reserve index and peripheral oxygen saturation and partial arterial oxygen pressure values in patients undergoing hypotensive anesthesia

#### H. Sarizeybek^1^, C. Balci^2^

##### ^1^Anesthesiology, Zonguldak Public Hospital, Zonguldak, Turkey; ^2^Anesthesiology and Reanimation, Kutahya Healty Science Un., Kutahya, Turkey

*Critical Care* 2021, **25**(**Suppl 1**): P164

**Introduction**: Excessive oxygen administration is known to cause absorption atelectasis during general anesthesia. There are concerns in using excessive oxygen during general anesthesia, the optimal fraction of inspired oxygen (FiO_2_) for general anesthesia is not studied. Oxygen Reserve Index (ORI) is a new parameter for noninvasive monitoring [1]. In our study, we evaluate the correlation between ORI and the SpO_2_ in underwent hypotensive anesthesia adults.

**Methods**: Our study is between the ages of 20–60; Twenty-four patients who will undergo elective tympanoplasty-mastoidectomy by applying hypotensive anesthesia are included in Group 1, and 9 patients who will undergo laryngectomy-neck dissection without hypotensive anesthesia are included as Group 2.

**Results**: There are 24 patients who have peroperative hyperoxic period in Group 1 and 5 of them were detected as hyperoxic with ORI (20.83%). In group 2, there are 8 patients who have hyperoxic period and 6 of them were detected as hyperoxic with ORI (75%).In our study, the value of ORI = 0 for the 150 mmHg value that we accept the safe PaO_2_ limit has high specificity and sensitivity.

**Conclusions**: In our study, although there was a significant relationship between PaO_2_ and ORI in both groups. In order to benefit from the mentioned advantages in terms of clinical use of ORI, we think that larger clinical studies may be beneficial in patients receiving hypotensive anesthesia.

**Reference**

1. Applegate R et al. Anesth Analg. 123:626–633, 2016

## P165

### Diaphragmatic ultrasound in difficult weaning: monitoring of SBT

#### J. F. Martínez Carmona, A. Navarro Cruz, R. M. Barraso González, J. M. Mora Ordoñez, M. J. Delgado Amaya

##### HRU Málaga, Intensive Care Unit, Málaga, Spain

*Critical Care* 2021, **25**(**Suppl 1**): P165

**Introduction**: Faced with a patient with difficult weaning, we must evaluate all the factors involved. Diaphragmatic dysfunction, due to injury or atrophy, plays a key role in these patients, so it is important to carry out an adequate assessment, for this we can use diaphragmatic ultrasound, which is a useful, non-invasive tool available at the bedside.

**Methods**: We evaluated a 62-year-old patient with a history of hypertension, DM2 and obesity (BMI > 30) who was admitted to the ICU due to severe acute respiratory failure due to SARS-Cov2 requiring ECMO support with good evolution, currently in the weaning phase of respiratory support Percutaneous tracheostomy was performed due to difficult weaning. She is stable at the hemodynamic and respiratory levels, with a good level of consciousness (RASS 0), adequate cough, but with significant paresis due to prolonged mechanical ventilation. We monitor diaphragmatic mobility through ultrasound during the weaning process. Diaphragmatic ultrasound is performed at the level of the right hemithorax, at the 7-8th rib in the mid-axillary line. We monitor diaphragmatic mobility through diaphragmatic excursion.

**Results**: We started a SBT in CPAP 8 cmH_2_O for 60 min, measurements were made after 30 min showing a diaphragmatic excursion between 22-26 mm. Subsequently, we removed the ventilator and switched to a humidifier with 50% FiO_2_, we performed a new ultrasound control after 30 min, presenting a diaphragmatic excursion between 18–22 mm. After 4 h, the patient begins to be fatigued, with use of accessory muscles, although he maintains SpO_2_ 98%. We repeat ultrasound, presenting a 16–17 mm diaphragmatic excursion. We again connected in PS 10 cmH_2_O over 6 cmH_2_O PEEP, FiO_2_ 40%, after 60 min we repeated the ultrasound control again, presenting a diaphragmatic excursion between 20-24 mm.

**Conclusions**: Monitoring of diaphragmatic mobility by ultrasound is useful and simple, it is a tool to consider in patients with difficult weaning.

**Figure:**
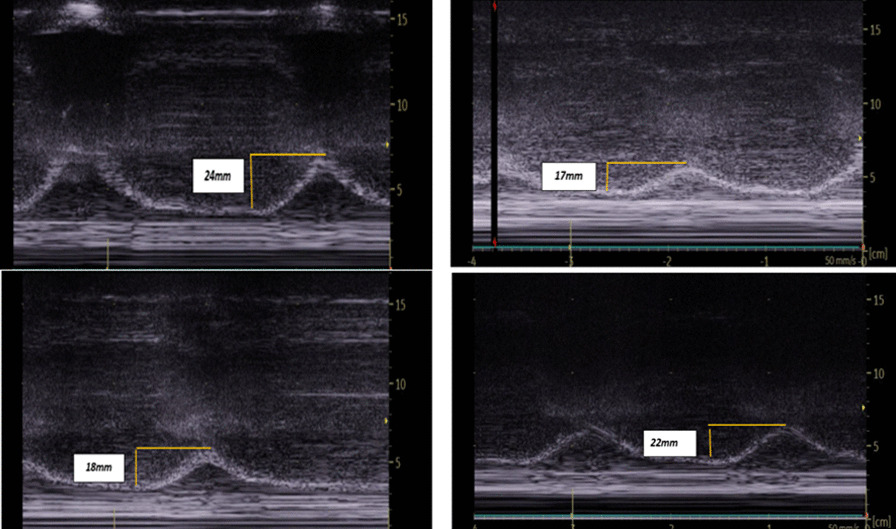
**Fig. 1** **(abstract P165)** A) CPAP 8 cmH_2_O, FiO_2_ 40% 30 min, B) tracheal mask oxygen FiO_2_ 50% 30 min, C) tracheal mask oxygen FiO_2_ 50% 4 h, D) support pressure 10/6 PEEP FiO_2_ 40% 30 min

## P166

### Monitoring the diaphragmatic electrical activity in critically ill patients with COVID-19 favors an early detection of high neuro-ventilatory drive

#### R. Di Mussi^1^, F. M. Murgolo^2^, R. I. Iannuzziello^2^, S. S. Spadaro^3^, I. M. Magnesa^2^, A. M. Miccolis^2^, A. C. Civita^2^, M. S. Stufano^2^, L. D. Dalfino^2^, S. G. Grasso^2^

##### ^1^Department of Emergencies and Organ Transplant, Bari University "A. Moro", Bari, Italy; ^2^Department of Emergencies and Organ Transplant, Bari, Italy; ^3^Ferrara University, Ferrara, Italy

*Critical Care* 2021, **25**(**Suppl 1**): P166

**Introduction**: COVID-19 may cause acute respiratory failure requiring mechanical ventilation. Assisted ventilation may prevent diaphragm atrophy, facilitating weaning. Nevertheless, a high drive could increase transpulmonary pressure causing patient self-inflicted lung injury (P-SILI) [1]. The diaphragmatic electrical activity (EAdi) can be continuously monitored at the bedside and is a reliable surrogate of the neuro-ventilatory drive. Objectives: To evaluate neuroventilatory drive, in terms of Eadi, during pressure support ventilation (PSV) in critically ill patients with COVID-19.

**Methods**: Eadi was continuously recorded for 30 min during PSV in 8 patients. PSV was set on clinical basis. For each mechanical breath, Eadi peak, VT, RR were measured. The Eadi peak was classified as LOW: between 1–5 μV; NORMAL: between 5–15 μV; HIGH: over 15 μV.

**Results**: The figure shows the distribution of LOW, NORMAL and HIGH Eadi during the 30 min. There was no difference in terms of VT and RR in the three different Eadi classes (VT: 0.43 ± 0.04 l, 0.43 ± 0.10 l 0.47 ± 0.05 l for LOW, NORMAL and HIGH Eadi respectively and RR: 25.52 ± 3.06 b/min, 22.97 ± 5.10 b/min, 21.02 ± 2.52 b/min for LOW, NORMAL and HIGH Eadi).

**Conclusions**: The incidence of high neuro-ventilatory drive during PSV in our cohort of COVID-19 patients was 25.83%. The fact that VT and RR were similar among the Eadi classes suggests that the clinical detection of P-SILI may be difficult. Further studies are needed to evaluate whether the incidence of high respiratory drive may influence the duration of mechanical ventilation and other clinically meaningful outcome parameters.

**Reference**

1. Esnault P et al. Am J Respir Crit Care Med 202:1173–1178, 2020

**Figure:**
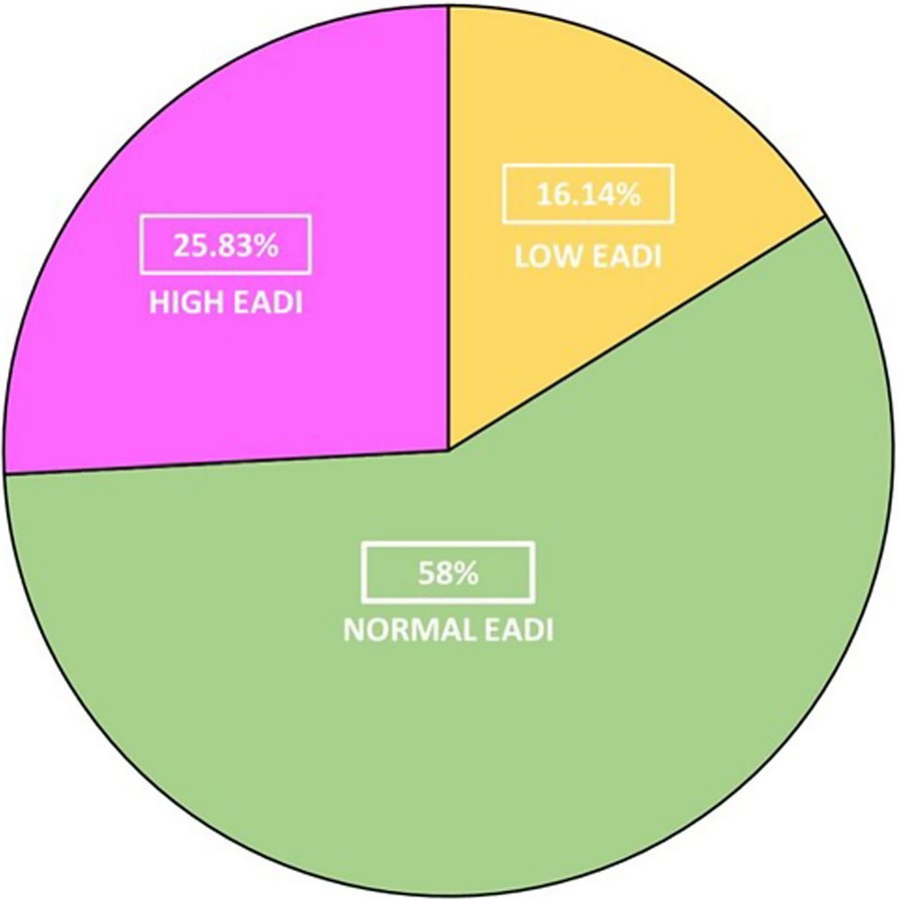
**Fig. 1** **(abstract P166)** % of Eadi distribution in the study period

## P167

### The utility of transthoracic echocardiographic measures in prediction of postoperative pulmonary edema development

#### M. K. Karaman ilić^1^, L. Š. Štefančić^1^, A. Š. Šimunić Forić^1^, M. L. Lekić^2^

##### ^1^Radiochirurgia, Faculty of Dental Medicine and Health, JJ Strosmmayer University of Osijek, Anesthesiology and Intensive Care, Sveta Nedelja, Croatia; ^2^Radiochirurgia, Faculty of Dental Medicine and Health,JJ Strosmmayer University of Osijek, Onkology and Radiotherapy, Sveta Nedelja, Croatia

*Critical Care* 2021, **25**(**Suppl 1**): P167

**Introduction**: Transthoracic ultrasound has been successfully used in intensive care units for many years to observe global cardiac function, valvular apparatus, and pericardial space [1]. This study aimed to assess changes in the tricuspid annular plane systolic excursion (TAPSE) and inferior vena cava (IVC) diameter and IVC collapsibility index (IVCcl) as a predictor for the development of postoperative pulmonary edema.

**Methods**: In the period from October 2020 to May 2021, one hundred patients scheduled for the major noncardiac surgical procedures were included in this observational study. TAPSE and IVCcl were measured the day before and the day after the procedure. Development of pulmonary parenchymal opacity, as a sign of pulmonary edema, was monitored by lung ultrasound.

**Results**: Changes in preoperative vs postoperative TAPSE measurements were seen: TAPSE 25 mm 2 vs 0 patients, TAPSE 24 mm 22 vs 8 patients, TAPSE 23 mm 21 vs 6 patients, TAPSE 22 mm 28 vs 15 patients, TAPSE 20 mm 20 vs 28 patients, TAPSE 19 mm 7 vs 37 patients and TAPSE 18 mm 0 vs 6 patients. Preoperative measurements showed that 73 patients had IVC diameter ≤ 2 cm with IVCcl > 50% and 27 patients had IVC diameter ≥ 2 cm with IVCcl > 50%. In the postoperative period, 47 had IVC diameter ≤ 2 cm of which 25 patients had IVCcl > 50% and 22 IVCcl 35- 50% while 43 patients had IVC diameter ≥ 2 cm of which 31 patient with IVCcl 35–50% and 12 patients with IVCcl < 35%. Patients with a decline in TAPSE also had reduction in IVCcl and B profile on the lung ultrasound.

**Conclusions**: A combination of TAPSE decline and IVCcl reduction has a strong predictive value for developing postoperative pulmonary edema due to fluid overload.

**Reference**

1. McLean A et al. Critical Care Ultrasound Manual, Elsevier, Australia, pp 64–75, 2012.

## P168

### Oxygenation parameters that affect the outcome in ICU patients with critical COVID-19 infection

#### A. Vakalos, I. Tsioulis

##### ICU, Xanthi General Hospital, Xanthi, Greece

*Critical Care* 2021, **25**(**Suppl 1**): P168

**Introduction**: COVID-19 pandemic continues to affect millions worldwide, while the critical form of the disease requires ICU hospitalization to manage mainly respiratory failure. Our retrospective observational study aimed to test the hypothesis that there is a difference in mean values of oxygenation parameters on ICU admission day, like oxygenation index (PaO_2_/FiO_2_) and oxygenation index over respiratory rate, among patients with confirmed critical COVID-19 infection who died and patients who survived ICU.

**Methods**: During late 2020 to 2021, 69 patients indicated with the diagnosis of critical COVID-19 disease admitted to ICU served to our community hospital. Mean age 66.14 years, length of stay 10.75 days, days under mechanical ventilation 9.97. The patients separated into two groups. Group A involved all patients who survived ICU (17 pts) and group B all patients who died in ICU (52 pts). We looked for statistically significant differences between the medians values of two groups according to oxygenation parameters on the ICU admission day, performing unpaired t - test or Mann–Whitney Test according equal S.D.s assumption.

**Results**: Results are shown in the Table.

**Conclusions**: According to our data, there was a strong statistically significant difference detected between the mean values of two groups according to oxygenation parameters we measured, while the oxygenation index over respiratory rate proved to be the stronger. Our data suggest that the oxygenation disorder was not only the main impact of COVID-19 infection on the ICU admission day, but was so important that affected the ICU outcome as well.

**Table:** **Table 1** **(abstract P168)** Results

Group A/B	Mean	Max	Min	p value
PaO_2_ / FiO_2_	111.5 / 76.4	299 / 189	44 / 33	0.0068
PaO_2_ / FiO_2_/RR	5.3 / 3.7	13.5 / 9.4	2.2 / 1.2	0.0047

## P169

### Effect of prone position regarding respiratory work and gas exchange during spontaneous breathing in patients with acute hypoxemic respiratory failure due to COVID-19 pneumonia

#### E. Chiodaroli^1^, P. D. Wendel Garcia^2^, S. Cappio Borlino^3^, M. Pitimada^3^, C. Granata^3^, M. Bonifazi^4^, P. Formenti^4^, L. Bolgiaghi^4^, S. Coppola^4^, D. Chiumello^5^

##### ^1^Anesthesiology and Intensive Care, ASST Santi Paolo e Carlo, San Paolo University Hospital, Milan, Italy; ^2^Institute of Intensive Care Medicine, University Hospital of Zurich, Zurich, Switzerland; ^3^Department of Pathophysiology and Transplantation, University of Milan, Milan, Italy; ^4^Department of Anesthesia and Intensive Care, ASST Santi Paolo e Carlo, San Paolo University Hospital, Milan, Italy; ^5^Department of Health Sciences, University of Milan, Milan, Italy

*Critical Care* 2021, **25**(**Suppl 1**): P169

**Introduction**: Aim of the present study was to investigate the effect of prone position regarding respiratory mechanics and gas exchange during spontaneous breathing in patients with acute hypoxemic respiratory failure (AHRF) due to COVID-19 requiring helmet CPAP.

**Methods**: Prospective enrolment of adults (> 18 years) with AHRF due to, radiologically and laboratory confirmed, COVID-19 pneumonia. Inclusion criteria: PaO_2_/FiO_2_ ratio < 300 mmHg and/or respiratory distress, requiring helmet CPAP. Exclusion criteria: unstable hemodynamics and Glasgow coma scale < 15. Blood gas analyses, respiratory mechanics and oesophageal pressures were collected during supine position and 3 h after the initiation of prone position. For statistical analysis, a fully scripted data management pathway was created within the R environment for statistical computing, version 3.6.1.

**Results**: The results are shown in Table 1. In particular, we observed that prone positioning was associated with a PaO_2_/ FiO_2_ ratio increase from 166 [136, 224] mmHg to 314 [232, 398] mmHg (p < 0.001). Respiratory work, estimated as the product of esophageal pressure, tidal volume and respiratory rate, decreased from supine (65 [46, 88] cmH_2_O*l/min) to prone position (51 [34, 67] cmH_2_O*l/min) (p < 0.001). The reduction in respiratory work was due to a reduction in respiratory rate from supine (20 [17, 24] breaths per minute (bpm)) to prone position (17 [15, 19] bpm) and corresponded to an improvement in dyspnea assessed both subjectively by the patient (Borg dyspnea scale) (p 0.005) and objectively by the physician (Work of Breathing (WOB) score) (p 0.001).

**Conclusions**: In this prospective cohort of spontaneously breathing patients affected by AHRF due to COVID-19 pneumonia, prone positioning was associated with an improvement in both gas exchange and respiratory mechanics. Widespread implementation of this easy to perform intervention could prove essential in improving critical care of non-invasively ventilated patients.

**Table:** **Table 1** **(abstract P169)** Results

	**Supine (N = 40)**	**Prone (N = 40)**	**p**
PaO_2_/ FiO_2_ ratio, mmHg	166 [136, 224]	314 [232, 398]	< 0.001
Respiratory rate, breaths/min	20 [17, 24]	17 [15, 19]	< 0.001
Esophageal pressure, cmH_2_O	-7 [-9, -5]	-6 [-9, -5]	0.306
Pes * respiratory rate, cmH_2_O/min	152 [104, 197]	118 [90, 150]	< 0.001
Pes*tidal volume*respiratory rate, cmH_2_O * l/min	65 [46, 88]	51 [34, 67]	< 0.001
Tidal volume, ml/kg	6.9 [6.0, 7.9]	6.9 [5.7, 7.9]	0.517
Stress, cmH_2_O	17 [14, 19]	16 [14, 18]	0.34

## P170

### Minimally invasive extracorporeal CO_2_ removal with PRISMA lung and PRISMA lung + : effect on CO_2_ clearance

#### F. Turani^1^, G. Barettin^2^, P. Zulli^2^, S. Busatti^2^, F. Gargano^2^, F. Vannicola^3^, L. Zamidei^4^, G. Consales^4^

##### ^1^Anesthesia and Intensive Care, Aurelia and European Hospital, Rome, Italy; ^2^Aurelia and European Hospital, Rome, Italy; ^3^Tor Vergata University, University of Rome Tor Vegata, Roma, Italy; ^4^Intensive Care Unit, Santo Stefano Hospital, ICU S Stefano Hospital Prato Italy, Prato, Italy

*Critical Care* 2021, **25**(**Suppl 1**): P170

**Introduction**: ECCO_2_ removal may be useful in acute decompensated chronic obstructive disease and in ARDS to improve mechanical ventilation. ECCO_2_ removal may be also combined with renal replacement therapy when AKI and respiratory failure coexist. Aim of this study is to quantify membrane lung CO_2_ removal (VCO_2_ ML) of two membrane lung oxygenators inserted in a RRT paltform.

**Methods**: Fifteen patients were submitted to RRT combined with ECCO_2_R integrated into the Prismaflex platform (Baxter, USA). In 10 patients a 0.35 m^2^ surface area polymetilpentene heparine coated hollow fiber membrane oxygenator (PrismaLung, Baxter, USA) was used. In five patients, a 0.8 m^2^ surface area polimetylpentene and phosforilcoline coated membrane oxygenator was used (PrismaLung + , Baxter, USA). The RRT was CVVHDF mode and pump flow was gradually increased from 200 ml/min to 400 ml/min. Heparin was used for anti-coagulation. Arterial, pre- and post-oxygenator blood gas were obtained to calculated VCO_2_ ML [1].

**Results**: All the treatments were completed without major complications. Pump flow was 350 ± 80 ml/min in all patients. Patients treated with PrismaLung and PrismaLung + had 10 and 7 VCO_2_ ML measurements respectively. VCO_2_ ML was 64 ± 24 ml/min in the PrismaLung patients and 104 ± 13 ml min in the PrismaLung + patients (p < 0.01).

**Conclusions**: ECCO2R combined with RRT may be used safety in patients with AKI and respiratory failure. PrismaLung + seems to remove CO_2_ better then PrismaLung, as reported from experimental data. Larger studies are warranted.

**Reference**

1. Allardet-Servent J et al. Crit Care Med 43:2570–2581, 2015

## P171

### Combined ACE and aminopeptidase inhibition reduces inflammation and maintains normal blood pressure in ventilation induced lung injury

#### M. Xinjun^1^, V. Tretter^2^, Y. Zhu^2^, F. Kraft^2^, L. Paulis^3^, M. Poglitsch^4^, B. Vigl^3^, R. Ullrich^2^

##### ^1^Department of Anaesthesia, Critical Care and Pain Medicine, Medical University of Vienna, Vienna, Austria; ^2^Medical University of Vienna, Vienna, Austria; ^3^Alterras Therapeutics GmbH, Vienna, Austria; ^4^Attoquant Diagnostics GmbH, Vienna, Austria

*Critical Care* 2021, **25**(**Suppl 1**): P171

**Introduction**: Patients in need of mechanical ventilation may suffer additional damage to the lungs from ventilator-induced lung injury (VILI) [1]. ACE inhibition was found to be beneficial, but its hypotonic effect impedes clinical application [2]. Here we tested, whether additional manipulation of the renin-angiotensin-system (RAS) by blocking aminopeptidases can alleviate this undesired effect.

**Methods**: Anesthetized mice (C57/BL6, 18–28 g, 8–10 weeks) were mechanically ventilated with low or high tidal Volume (LVT, 6 ml/kg; HVT, 30 ml/kg) for 4 h, and treated with the ACE inhibitor Lisinopril (0.15 ug/kg/min) with or without aminopeptidase inhibitor (ALT00) at 2.7, 10 or 100 ug/kg/min. BALF was analyzed for inflammatory cytokine levels (IL-6, KC, MIP-2 and IL-1β) by ELISA. Mean arterial pressure (MAP) was measured continuously in the right carotid artery. Equilibrium concentrations of RAS metabolites in plasma were measured by LC tandem MS.

**Results**: ACE inhibition decreased ventilation-induced inflammatory cytokines and MAP. Co-treatment with ALT00 reduced inflammation and increased dose dependently levels of Ang 1–7, an anti-inflammatory and anti-fibrotic RAS metabolite. Various doses had mixed effects of normalizing MAP and reducing cytokine secretion.

**Conclusions**: Combined manipulation of the RAS system might represent a promising new treatment strategy for VILI requiring further mechanistic exploration.

**References**

1. Baumgardner JE et al. Am J Respir Crit Care Med 166: 1556–1562, 2002;
2. Jiang JS et al. J Appl Physiol 102: 2098–2103, 2007.

## P172

### Back to basics: Improving consistency of 6 ml/kg PBW using the Model for Improvement

#### V. Varley, R. Starba, A. Revill

##### Intensive Care Unit, Torbay Hospital, Torquay, UK

*Critical Care* 2021, **25**(**Suppl 1**): P172

**Introduction**: ICU staff are aware that hypoxic patients with ARDs should be ventilated at 6 ml/kg. Hitting this target is difficult to achieve, even in protocolised research trials [1]. We have been monitoring the total time within the target 6 ml/kg PBW for patients with a P/F ratio < 200 for 18 months using this as an outcome measure to monitor iterative changes to improve adherence to this target. Here we report the results of a project determining whether heights are correctly measured and inputted into the ventilators.

**Methods**: All patients who required ventilation for > 12 h were selected for this study during a five-week period in an ICU of a DGH. Data collected for each patient included: PBW, height on IntelliSpace Critical Care and Anaesthesia (ICCA), height in ventilator, measured height, tidal volume (ml/kg), P/F ratio and mode of ventilation.

**Results**: Data were collected for 21 patients. 1 had no PBW inputted on the ventilator. 19.1% of patients (4/21,95% CI 5.5–41.9%) had PBW inputted incorrectly. 14.3% had ventilation heights significantly different to their actual height on ICCA (2/14, 95% CI 1.8–42.8%). 36.8% (7/19, 95% CI 16.3–61.6%) had a time delay of > 1 h between ventilation and recording their height on the ventilator. 23.8% (6/21, 95% CI 11.3–52.2%) had a tidal volume of > 6.0 ml/kg of PBW.

**Conclusions**: The time we hit the 6 ml/kg target varies weekly with performance worst in weeks with patients on ventilators with a P/F < 200. This demonstrated that we are not consistently measuring patients correctly or inputting measurements promptly in the ventilator. This provides a great starting point for an education programme, the success of which can be monitored using the weekly outcome measure of % time < 6 ml/kg reported on the run chart above (Fig. 1).

**Reference**

1. Young D et al. N Engl J Med 368:806–813, 2013

**Figure:**
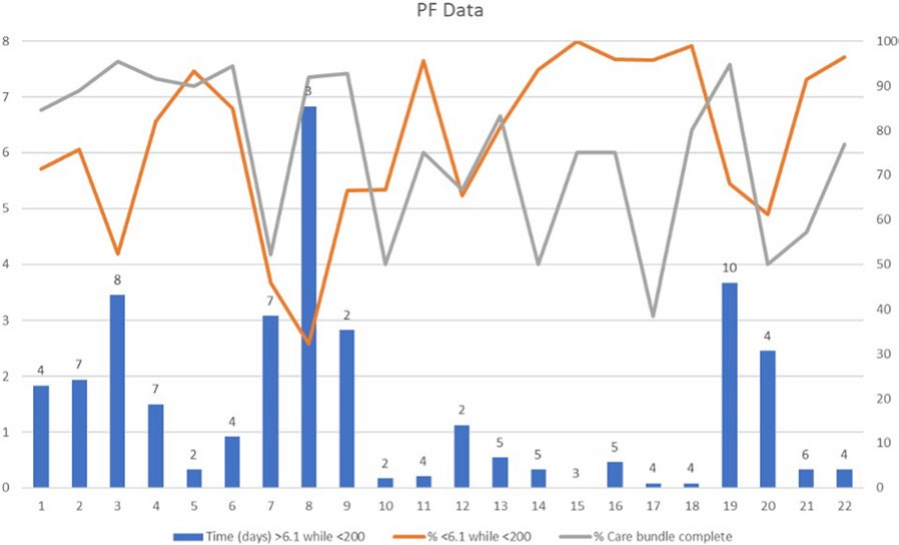
**Fig. 1** **(abstract P172)** Run chart of % of patients with a P/F < 200 who are ventilated at or below 6.1 ml/kg. Plotted with the total time in days when patients had a P/F < 200

## P173

### Mechanical power better determinant of mortality in ARDS patients due to COVID-19 under mechanical ventilation

#### L. A. Cortes-Puentes, D. Osorio, M. Gomez, A. Valencia, L. Sarmiento, V. Nieto, D. Molano, C. Lotta

##### Critical Care Units, Loscobos Medical Center, Bogota, Colombia

*Critical Care* 2021, **25**(**Suppl 1**): P173

**Introduction**: Protective ventilation parameters have been associated with better outcomes in patients with ARDS, however, ventilator power could be associated whit better outcomes. In this study, we analyzed the trend of the ventilatory parameters including the respiratory work and ventilatory power during the first four days of mechanical support in patients with ARDS due to COVID-19.

**Methods**: Observational analytical retrospective study. We included ARDS patients due to COVID-19 with need of invasive mechanical ventilation support for at least four days, that were admitted in the intensive care units between March 2020 and 2021. We describe the trend of ventilatory parameters in the first four days and evaluate the association of these parameters trends with mortality.

**Results**: Forty-nine patients meeting the inclusion criteria were analyzed, no significant difference was found in the ventilatory parameters of the surviving and non-surviving patients on the first day of mechanical ventilation support, meeting criteria for protective ventilation in both groups. The progression of ventilatory parameters during the first four days evidenced a significant increase in the energy delivered to the respiratory system in the no survivors patients compared with survivors patients, especially in the ventilator inspiratory power (4.82 vs 0.2 J/min) p: 0.04. There is a trend of increase of adjusted ventilator inspiratory power (8 vs 0.2 J/min/kg ideal body weight*10) ventilator work (0.18 vs -0.03 J) and adjusted ventilator work (0.3 vs 0 J/kg ideal body weight*100). No significant difference was found in the trend of driving pressure over the first four days of support.

**Conclusions**: More than the absolute value in the ventilatory parameters at the beginning of mechanical ventilation support, the trend to increase in the ventilator inspiratory power over the first days of mechanical support, could be better associated with mortality in ARDS patients due to COVID-19.

## P174

### Performance of Access IL-6 assay in predicting risk for mechanical ventilation in COVID-19 patients

#### N. Robert^1^, C. Morales^2^, A. Mendoza^1^, G. Rocamora^1^, C. Garcia^2^, M. D. Quesada^3^, I. Castro^4^, D. Careaga^4^, R. Magari^5^, L. Tejidor^4^

##### ^1^Emergency Department, Hospital Universitari Germans Trías i Pujol, Badalona, Spain; ^2^Laboratory Medicine Department, Hospital Universitari Germans Trías i Pujol, Badalona, Spain; ^3^Microbiology Department, Hospital Universitari Germans Trías i Pujol, Badalona, Spain; ^4^Clinical Affairs, Beckman Coulter Inc., Miami, USA; ^5^Bioinformatics/Biostatistics, Beckman Coulter Inc., Miami, USA

*Critical Care* 2021, **25**(**Suppl 1**): P174

**Introduction**: The clinical study evaluated IL-6 measurements as an early indicator of disease progression and poor prognosis in patients with SARS-CoV-2 infection. Elevated IL-6 has been shown to identify patients at risk of hypoxemia and need for mechanical ventilation [1].

**Methods**: A retrospective cohort study enrolled adults presenting to the Emergency Department (ED) between March 18 and May 4, 2020 with symptoms suggestive of COVID-19 and who were RT-PCR positive for SARS-CoV-2. All patients had blood samples drawn at ED presentation and tested with the Access IL-6 assay on Unicel DxI 800 immunoanalyzer (Beckman Coulter Inc.). Results of radiological studies and respiratory treatments (non-invasive and invasive mechanical ventilation, nasal cannula) were extracted from the medical charts. An IL-6 cut-off of 35 pg/ml based on literature was utilized for the analysis [1].

**Results**: Seventy-five RT-PCR confirmed SARS-CoV-2 patients were initially enrolled. The prevalence of mechanical ventilation in this cohort was 32% with a median time from sample draw to mechanical ventilation of 4 days, and a mortality of 17%. 10 patients were excluded, as despite having severe hypoxemia, died before receiving mechanical ventilation. The median IL-6 levels were 26.21 (non-ventilated group) vs. 82.61 pg/mL in those receiving mechanical ventilation. ROC analysis of these 65 patients yielded an AUC of 0.800 (95% CI 0.695 – 0.905) for baseline IL-6 levels. At a cut-off of 35 pg/ml, IL-6 effectively differentiated COVID-19 patients who received mechanical ventilation, with a sensitivity of 95.2% (95% CI 77.3 – 99.2), specificity of 56.8% (95% CI 42.2 – 70.3), PPV of 51.3% (95% CI 36.2 – 66.1) and NPV of 96.2% (95% CI 81.1 – 99.3).

**Conclusions**: The Access IL-6 assay is a highly sensitive marker to aid in determining the risk of intubation by mechanical ventilation in confirmed COVID-19 patients. This assay received Emergency Use Authorization from the US Food and Drug Administration.

**Reference**

1. Herold T. J Allergy Clin Immunol. 146:128–136, 2020

## P175

Withdrawn

## P176

### Mechanical power and protective ventilation as mortality determinant in ARDS associated to COVID-19

#### L. A. Cortes-Puentes, D. O. Osorio-Perdonmo, A. V. Valencia, V. N. Nieto, M. G. Gomez

##### Critical Care Units, Loscobos Medical Center, Bogota Colombia, Colombia

*Critical Care* 2021, **25**(**Suppl 1**): P176

**Introduction**: The main cause of admission to intensive care of patients with COVID-19 is hypoxemic respiratory failure with a requirement for ventilatory support; the best results have been associated with protective ventilation and the energy disipated over the lung as mechanical power aplicate in the first days of mechanical ventilation. The scope of our paper is find the relation of mortalidad and the ventilation setting.

**Methods**: Observational analytical retrospective cohort of ARDS patients managment with mechanical ventilation in a critical care unit in Bogota Colombia. 101 adult patients were admitted to LosCobos intensive care between March 2020 and 2021 with hypoxemic respiratory failure secondary to COVID-19, with indication for mechanical ventilation.

**Results**: We analyzed the first four days of mechanical ventilation. On the first of starting mechanical ventilation, no significant difference was found in the ventilatory parameters of the surviving and non-surviving patients. on the fourth day, the variables under study were quantitatively higher in the group that died: FiO_2_ 73.93% ± 9.3 vs 42.91 ± 12.3 (MD: 30.71 95% CI - 6.87–68.30), tidal volume 434.07 ± 58.982 vs 415.71 ± 43.45 (MD: 18.36 95% CI - 14.63–51.35), respiratory rate 27 ± 2.5 vs 14 ± 2.68 (MD: -0.07 95% CI - 1.75–1.79), power 31.058 ± 9.28 vs 26.95 ± 7.25 (MD: 4.41 95% CI - 1.35–10.18). The trend of power, adjusted power, work and adjusted work is to increase between day 1 to day 4 in the group of those who died: 3.66 ± 7.85, 0.05 ± 0.12, 0.13 ± 0.50 and 0.01 ± 0.01, respectively. Unlike those who did not die, the trend of the variables is to decrease.

**Conclusions**: The energy delivered to the respiratory system during mechanical ventilation was associated with mortality in our description, where despite initial protective parameters, ventilatory power and work increased during the first four days of ventilation in the deceased patients.

## P177

### Outcomes of patients with COVID-19 acute respiratory distress syndrome requiring invasive mechanical ventilation admitted to an intensive care unit in South Africa

#### C. Arnold-Day^1^, R. Van Zyl-Smit^2^, I. Joubert^1^, D. Thomson^1^, D. Fredericks^1^, M. Miller^1^, W. Michell^1^, P. Semple^3^, J. Piercy^1^

##### ^1^Division of Critical Care, Department of Anaesthesia and Perioperative Medicine, University of Cape Town and Groote Schuur Hospital, Cape Town, Western Cape, South Africa; ^2^Division of Pulmonology, Department of Medicine, University of Cape Town and Groote Schuur Hospital, Cape Town, Western Cape, South Africa; ^3^Department of Neurosurgery, University of Cape Town and Groote Schuur Hospital, Cape Town, Western Cape, South Africa

*Critical Care* 2021, **25**(**Suppl 1**): P177

**Introduction**: Up to 32% of patients with COVID-19 pneumonia may require ICU admission or mechanical ventilation [1, 2]. Data from low- and middle-income countries for COVID-19 ARDS are limited. Groote Schuur Hospital in Cape Town, South Africa expanded its ICU service to support patients with COVID-19 ARDS requiring invasive mechanical ventilation (IMV). We report on patients' characteristics and outcomes from two pandemic waves.

**Methods**: All adult patients with COVID-19 ARDS admitted to ICU for IMV were included in this prospective cohort study. Data were collected from 5th April 2020 to 5th April 2021. Ethical approval was granted (HREC: 362/2020). Consent was obtained for survivors and waived for patients who died.

**Results**: Over the 12-month study period, 461 patients were admitted to the designated COVID-19 ICU. Three-hundred-and-eighty patients met study criteria and 377 had confirmed hospital discharge outcomes. The median age of patients was 51 years (range 17–71), 50.5% were female and the median BMI was 32 kg/m2 (IQR 28–38). The median P/F ratio was 97 (IQR 71.5–127.5) after IMV was initiated. Co-morbidities included diabetes (47.6%), hypertension (46.3%) and HIV infection (10.5%). Of the patients admitted, 30.8% survived to hospital discharge with a median ICU length of stay of 19.5 days (IQR 9–36). Predictors of mortality after multivariate analysis were: male (OR: 1.79), increasing age (OR: 1.04), higher SOFA score (OR: 1.29).

**Conclusions**: In a resource limited environment, escalation of ICU IMV support achieved a 30.8% hospital survival in patients with COVID-19 ARDS. The ability to predict survival remains difficult given this complex disease.

**References**

1. Huang C et al. Lancet 395:497–506, 2020.
2. Guan WJ et al. N Engl J Med 382:1708–1720, 2020.

## P178

### Diaphragm neurostimulation with mechanical ventilation mitigates neuroinflammation and results in microglia cellular characteristics similar to never-ventilated subjects

#### T. Bassi^1^, E. Rohrs^2^, K. Fernandez^2^, M. Nicholas^2^, M. Ornowska^2^, M. Gani^3^, D. Evans^3^, S. Reynolds^2^

##### ^1^Physiology, Simon Fraser University, Burnaby, Canada; ^2^Simon Fraser University, Burnaby, Canada; ^3^Lungpacer Medical Inc, Exton, USA

*Critical Care* 2021, **25**(**Suppl 1**): P178

**Introduction**: Mechanical ventilation (MV) has been associated with neuroinflammation. Microglia are key cells that initiate and control inflammation in the brain, behaving either as pro-inflammatory or anti-inflammatory cells. We investigated whether transvenous temporary diaphragm neurostimulation (TTDN) in synchrony with MV, a hybrid ventilation strategy, mitigates neuroinflammation.

**Methods**: Thirty-one healthy juvenile pigs (4–5 months, 48–66 kg) with non-injured lungs were divided into four groups: MV only (MV group, n = 10), MV in association with diaphragm neurostimulation delivered either every other breath (TTDN50% + MV group, n = 8) or every breath (TTDN100% + MV group, n = 7) and never ventilated (NV group, n = 6). TTDN targeted a reduction in pressure–time product between 15 and 20%. Volume control MV was set to achieve and maintain a tidal volume of 8 ml/kg with a PEEP of 5 cmH2O. Hippocampus samples were harvested, and IBA-1 assay was used to stain microglia. Machine learning software classified microglia cells as either pro-inflammatory or anti-inflammatory based on cellular biometric characteristics. Data are expressed as median and interquartile range. The Kruskal–Wallis test and Dunn’s multiple comparison test were used for statistical analysis. P values £0.05 were considered statistically significant.

**Results**: Microglia percentages were dependent on TTDN dose. Differences in percentages of IBA-1-positive hippocampal cells were statistically significant between groups, p < 0.0001 (Fig. 1). Microglia from the TTDN100% + MV group had cellular characteristics statistically indistinguishable from the NV group.

**Conclusions**: TTDN mitigates neuroinflammation after 50 h of MV. TTDN every breath results in microglia cellular characteristics similar to the NV group.

**Figure:**
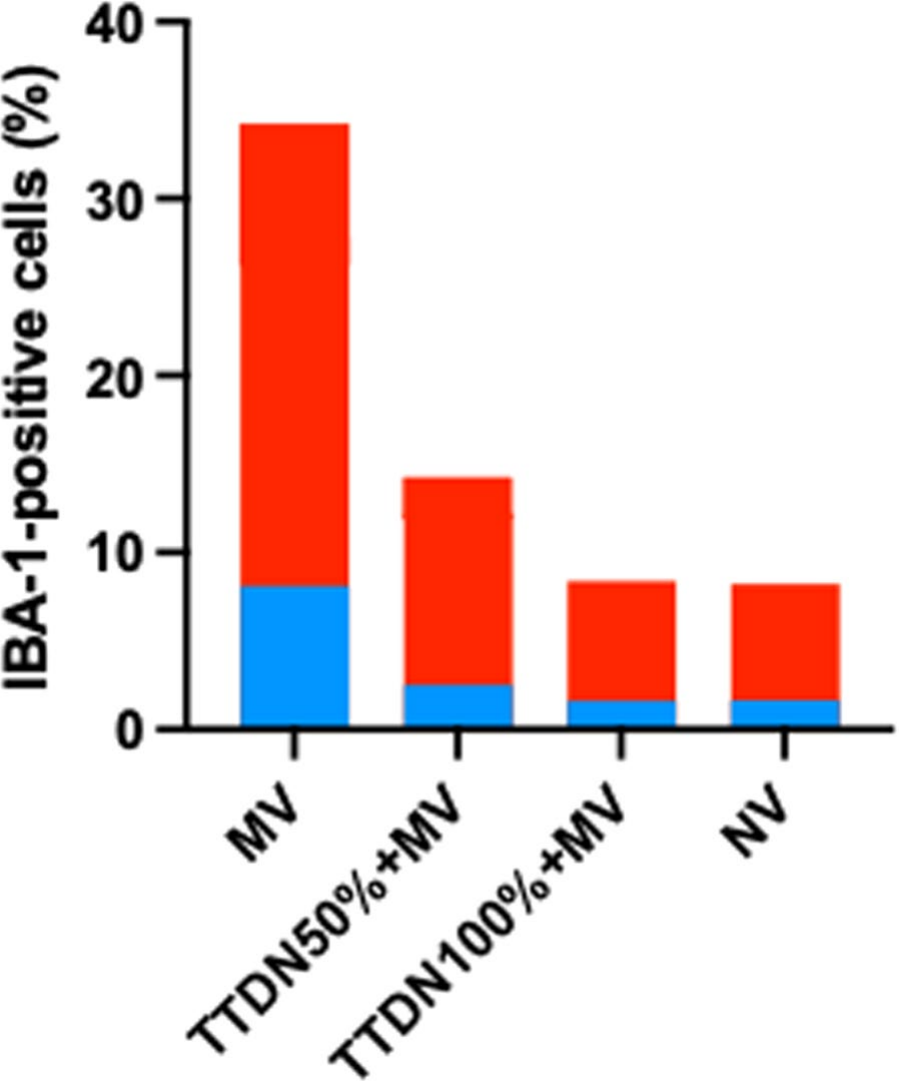
**Fig. 1** **(abstract P178)** IBA-1-positive cell percentages for all groups; pro-inflammatory in blue and anti-inflammatory in red. The MV group had 36.17% (30.71–48.27) IBA-1-positive cells, consisting of 8.11% (6.74–9.69) with pro-inflammatory and 27.82% (23.63–32.00) with anti-inflammatory characteristics. The TTDN50% + MV group had 16.70% (10.82–22.42) IBA-1-positive cells, consisting of 2.50% (2.02–2.88) with pro-inflammatory and 12.23% (7.36–19.54) with anti-inflammatory characteristics. The TTDN100% + MV group had 9.80% (7.86–11.19) IBA-1-positive cells, consisting of 1.60% (1.10–1.77) with pro-inflammatory and 8.37% (6.86–9.41) with anti-inflammatory characteristics. The NV group had 10.12% (8.93–10.65) IBA-1-positive cells, consisting of 1.63% (1.32–2.06) with pro-inflammatory and 8.20% (7.68–8.50) with anti-inflammatory characteristics

## P179

### Prone position (PP) on patients (pt) supported with veno-venous extracorporeal membrane oxygenation (VV-ECMO): a risk worth taking?

#### V. Templeman, F. Caetano, G. Georgovasilis, T. Topciu, S. Ledot

##### Adult Intensive Care, The Royal Brompton and Harefield Hospitals - part of GSTT NHS Foundation trust, London, UK

*Critical Care* 2021, **25**(**Suppl 1**): P179

**Introduction**: PROSEVA proved that PP in severe ARDS reduces mortality without an increase in adverse outcomes. However, complications such as cannula displacement could be life-threatening, and the benefits of PP whilst on ECMO are yet to be proved. Our study aimed to assess safety of proning patients on VV-ECMO and try to identify signs of potential benefit of this therapy.

**Methods**: Retrospective data collection from electronic Pt notes including: demographics; SARF aetiology; CT findings; indication; number of PP sessions; duration of ECMO and complications. Pre- and post-P/F ratio, ventilator and ECMO settings were compared. The data were analyzed using statistical software SPSS®

**Results**: Fifty-two ECMO Pts (71% male; 46 ± 14 years-old; BMI 29 ± 6 kg/m2) were PP from 2016–2021. 73% survived to ECMO decannulation and were discharged from our center. ECMO duration was 26 ± 18 days. The most frequent etiologies of SARF were community acquired pneumonia (38.4%) and COVID-19 pneumonitis (23.1%). CT chest prior to PP documented consolidation in 94.2% and bilateral infiltrates in 84.6% A high PEEP CT was performed in 16 Pts, showing recruitability in 81%. Most patients were proned for recruitment (32.7%) or due to refractory hypoxia (30.8%). Ventilator and ECMO support decreased with PP (FiO_2_ on the ventilator 0.5 ± 0.2 vs 0.4 ± 0.2; p = 0.001; ECMO blood flow (4.4 ± 0.8 vs 4.2 ± 0.8 l/min; p = 0.006). A significant improvement in the following variables was seen after PP: lung compliance (19 ± 17 vs 25 ± 20 ml/cmH_2_0; p = 0.014), P/F ratio (137 ± 102 vs 180 ± 126; p = 0.001) and 100% test (17 ± 10 Kpa vs 31 ± 16 Kpa, p < 0.001). Two Pts required emergency deproning due to oropharyngeal bleeding and one due to sudden drop in ECMO flow. Otherwise, no major adverse events (ECMO cannula removal, accidental extubation or central vascular access removal) were documented.

**Conclusions**: PP in VVECMO support therapy seems to be safe and is associated with improvement in lung mechanics and oxygenation. Further research is warranted to assess its effect on ECMO duration.

## P180

### ECMO- MADRID. Implementation of the veno-venous ExtraCorporeal Membrane Oxygenation (ECMO) Retrieval program in the region of Madrid (Spain). Experience of Hospital Universitario 12 de Octubre ECMO referral center

#### A. Delgado-Téllez, E. Renes, J. L. Pérez-Vela, J. A. Rubio Mateo-Sidrón, A. Lesmes, T. Grau, J. C. Montejo González

##### Intensive Care Medicine, Hospital Universitario 12 de Octubre, Madrid, Spain

*Critical Care* 2021, **25**(**Suppl 1**): P180

**Introduction**: The objectives of this study are: first to describe the cases transferred to an ECMO referral center (Hospital Universitario 12 de Octubre), second to investigate characteristics before ECMO and while the patient was on ECMO, third to analyse the presence or not of complications secondary to transfer and cannulation and finally to analyse the ICU outcome.

**Methods**: This is a prospective descriptive study of ECMO retrieval program done from November 1, 2020 to May 15, 2021. We analyzed baseline descriptive variables, severity Scores, variables before and during ECMO, prone position on ECMO, complications during cannulation and transfer. Finally ICU survival was examined.

**Results**: Seventeen cases were accepted on the ECMO Retrieval program from November 1, 2020, to May 15, 2021. Thirteen (76%) of a total of 17 cases were male. Fifteen cases were accepted for emergency ECMO cannulation in the hospital of origin and transfer and 2 were accepted for conventional transfer and cannulation was done on the ECMO referral center. Basic characteristics of the cases were: median age of 48 (IQ 18) and IMC 31.2 (IQR 9.8). The etiology of SDRA was predominantly secondary to SARS-CoV-2 (COVID-19) infection, 16 cases of a total of 17 cases (94%). Lenght of non invasive ventilation and invasive ventilation before ECMO were 7.5 days (10.3 IQR) and 2 days (2.5 IQR) respectively and length of ICU admission before ECMO was 3 days (IQR 5). Severity scores were APACHE 8 (IQR 4), SOFA 4 (IQR 2.5), ELSO 3 (IQR 3). The cases where on ECMO Prone position was done on 9 cases (53%). Median days on ECMO were 13 days (IRQ 10). Succesfull weaning from ECMO were achieved on 9 cases (53%). No complications were seen on transfer or cannulation. ICU Survivors were 6 (35%).

**Conclusions**: This is the first pilot study for ECMO retrieval in the region of Madrid, Spain. Despites high mortality in the context of COVID-19 pandemia, ECMO-MADRID retrieval program assures ECMO availability, and it allows to rescue severe ARDS cases in the whole region.

## P181

### Detection of patient-ventilator asynchrony by machine learning based on synthetic data

#### T. Bakkes, A. Van Diepen, M. Mischi, P. Woerlee, S. Turco

##### Electrical Engineering, Eindhoven University of Technology, Eindhoven, Netherlands

*Critical Care* 2021, **25**(**Suppl 1**): P181

**Introduction**: Patient-ventilator asynchrony (PVA) is difficult to detect consistently since it relies on visual inspection of the waveforms. However, the development of automatic detection methods is hampered by the lack of properly labelled ventilation data. One solution would be to utilize artificially generated data. In this study, we compared the performance of a detection method based on clinical data to a detection method based on simulated data.

**Methods**: The detection method was based on a neural network that was trained to detect the start and end of the patient breathing efforts, as described in [1]. In our work, two different datasets were utilized to train the network. The first dataset consisted of clinical data and the second consisted of simulated data obtained from the model in [2]. The detection method was trained and tested in four different settings. As a baseline, only the clinical data was utilized. In the second to the fourth setting, the training data was extended with simulated data. Three different ratios of clinical to simulated data were utilized in each setting. To prevent overfitting the model was cross-validated (CV) with each patient being onefold of the cross-validation.

**Results**: The quality of the detection for each of the trained networks was evaluated by comparing the detections to the ground truth, and the subsequent results are found in Table I.

**Conclusions**: The results from Table I show that the inclusion of simulated data during the training resulted in a marginal improvement of detection quality. A maximum improvement of roughly 1% in both precision and recall was achieved with a mix-ratio of 1:2. In future research, further improvements in the simulations, e.g. including higher variability and more realistic triggering and cycling, can possibly lead to improved detection performance.

**References**

1. Bakkes T et al. EMBC 2020 150–153, 2021
2. van Diepen A et al. arVix, 2021

**Table:** **Table 1** **(abstract P181)** Detection results of the four different settings. The ratios denote the number of simulated data points to clinical data points. Results are given in positive predictive value (PPV) and true positive rate (TPR)

Trained on	Clinical	Clinical & Sim (1:1)	Clinical & Sim (1:2)	Clinical & Sim (1:3)
Tested on	Clinical (CV)	Clinical (CV)	Clinical (CV)	Clinical (CV)
PPV	96.0%	96.0%	96.9%	96.6%
TPR	95.4%	95.7%	96.5%	95.9%

## P182

### Effect of hospital case volume on clinical outcomes of patients requiring extracorporeal membrane oxygenation

#### P. Y. Ng^1^, S. Fang^2^, A. Ip^2^, J. C. Lin^2^, C. W. Ngai^3^, W. C. Sin^2^, W. M. Chan^3^

##### ^1^Department of Medicine, The University of Hong Kong, Hong Kong, Hong Kong, SAR China; ^2^The University of Hong Kong, Hong Kong, Hong Kong, SAR China; ^3^Queen Mary Hospital, Hong Kong, Hong Kong, SAR China

*Critical Care* 2021, **25**(**Suppl 1**): P182

**Introduction**: The utilization of extracorporeal membrane oxygenation (ECMO) has increased rapidly around the world. Being an overall low-volume high-cost form of therapy, the effectiveness of having care delivered in segregated units across a geographical locality is debatable. Longitudinal trends in outcomes of ECMO patients managed in high- and low-case volume centers are not well described.

**Methods**: This was a retrospective observational study including all adult ECMO cases admitted to public hospitals in Hong Kong between 2010 and 2019. With reference to an international guideline, “high-volume” centers were those with > 20 ECMO cases annually, while “low-volume” centers were those with ≤ 20. Clinical outcomes such as hospital mortality of patients who received ECMO care in high-volume centers were compared with those in low-volume centers.

**Results**: A total of 911 patients received ECMO – 297 (32.6%) veno-arterial ECMO, 450 (49.4%) veno-venous ECMO, and 164 (18.0%) ECMO-cardiopulmonary resuscitation. The median age was 54 years (IQR 42–62), and 583 (64.0%) were male. The average number of ECMO cases managed in high- and low-volume centers was 25 and 12 per center per year, respectively. Management in a high-volume center was associated with decreased ICU mortality compared with a low-volume center (255 [50.2%] vs 201 [49.9%]; adjusted OR 0.70, 95% CI 0.52 – 0.95, p = 0.024). Over the 10-year period, the observed mortality was similar to the APACHE-IV predicted mortality in high-volume centers, but was worse than predicted in low-volume centers (Fig. 1).

**Conclusions**: We demonstrated that ECMO in high-volume centers was associated with decreased mortality that closely reflected predicted outcomes. With the drastic increase in ECMO utilization worldwide, our data supports current international guidelines that delivery of ECMO should be consolidated to high-volume dedicated centers to improve patient outcomes of this expensive form of organ support.

**Figure:**
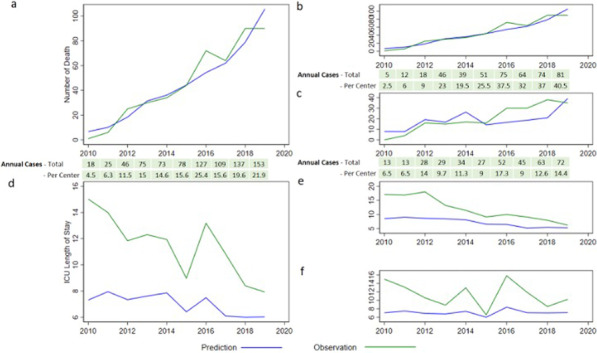
**Fig. 1** **(abstract P182)** These figures compare the observed (green lines) and APACHE IV predicted (blue lines) outcomes. The overall observed hospital mortality was similar to the predicted mortality (panel a). In high-volume centers, the observed hospital mortality was similar to the predicted mortality (panel b), while in low-volume centers, the observed hospital mortality became higher than the predicted mortality from 2015 to 2018 (panel c). The observed ICU length of stay was longer than predicted in all hospitals (panel d), high-volume centers (panel e), and low-volume centers (panel f)

## P183

### Effect of age on mortality in patients undergoing veno-venous ECMO: a systematic review and meta-analysis

#### M. B. Bordini^1^, L. M. Mascia^2^, E. M. Maietti^2^, L. B. Bambini^1^, I. C. Cavalli^1^, G. P. Pizzilli^3^, P. R. Rucci^2^, M. R. Ranieri^1^, T. T. Tonetti^1^

##### ^1^Department of Medical and Surgical Sciences, Alma Mater Studiorum University of Bologna, Bologna, Italy; ^2^Department of Biomedical and Neuromotor Sciences, Alma Mater Studiorum University of Bologna, Bologna, Italy; ^3^Department of Anesthesia and General Intensive Care Unit, Sant’Orsola-Malpighi Hospital, Bologna, Italy

*Critical Care* 2021, **25**(**Suppl 1**): P183

**Introduction**: Although use of veno-venous extracorporeal membrane oxygenation (VV-ECMO) for ARDS is increasing worldwide, criteria for eligibility are not clear and use of ECMO for older patients is still debated. The primary objective of this meta-analysis and meta-regression was to determine whether mortality on VV-ECMO is affected by patient’s age.

**Methods**: We searched Pubmed for studies published between 2015 and 2019, using the keyword ‘ECMO’. Only RCTs and observational studies on VV-ECMO with > 10 patients were included. Secondary analyses of large databases were excluded to avoid data duplication. We excluded records rated as ‘poor’ according to the NIH study quality assessment tools. Outcomes were ICU, hospital and 3-month mortality. The relationship between mean age and mortality was investigated with meta-regression [1] and results reported as regression coefficient (b) with 95% confidence interval.

**Results**: We included 63 studies (22 rated as ‘good’, 41 ‘fair’). ICU mortality was available in 22 studies, hospital-mortality in 45 studies, and 3-month mortality in six studies. We found a strong association between mean age and ICU mortality (b = 0.021, 95%CI: 0.011–0.031, p < 0.001) (Fig. 1). The sensitivity analysis (considering only ‘good’ studies) showed the same b coefficient but not a statistically significant relationship (b = 0.021, p = 0.124). Similar association was found for hospital-mortality (b = 0.014, 95%CI: 0.008–0.020, p < 0.001), confirmed by the sensitivity analysis (b = 0.020, 95%CI: 0.006 – 0.033, p = 0.008). There was a positive association (although not statistically significant b = 0.015, p = 0.204) between mean age and 3-months mortality; ‘good’ rated studies were too few to perform sensitivity analysis.

**Conclusions**: Our meta-regression shows that mortality on ECMO is independently influenced by the age of the patient. Further analyses are needed to determine whether an age cut-off exists, above which mortality increases unacceptably.

**Reference**

1. DerSimonian R et al. Controlled Clinical Trials 7:177–88, 1986.

**Figure:**
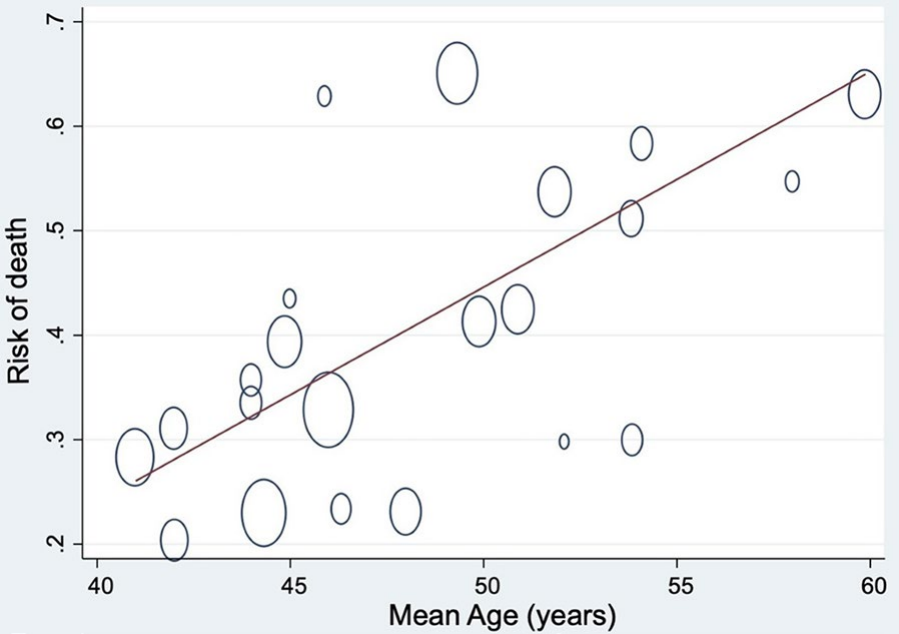
**Fig. 1** **(abstract P183)** Association between mean age and ICU mortality

## P184

### Extracorporeal membrane oxygenation in pregnant women with COVID-19: a case series

#### F. Al Shammari^1^, O. Bamasood^2^, M. Shamsah^3^, H. Alfoudri^3^, Z. Bitar^3^, J. Jagadeesan^1^, V. Pandian^1^, Y. Elkenany^1^, T. Tammam^1^, A. Alsarraf^3^

##### ^1^Intensive care and Anesthesia, Jahra Hospital, Jahra, Kuwait; ^2^Department of Anesthesia and Critical Care,Jahra Hospital, Jahra, Kuwait; ^3^Intensive care and Anesthesia, Al Adan Hospital, Mubarak Al Kabeer, Kuwait

*Critical Care* 2021, **25**(**Suppl 1**): P184

**Introduction**: Extracorporeal membrane oxygenation (ECMO) is important in the management of severe cardiac and pulmonary dysfunction when conventional methods fail. To study the outcome of pregnant women with severe COVID-19 ARDS who required ECMO.

**Methods**: All patients were pregnant, admitted with COVID-19 and received ECMO in the intensive care unit (ICU) at Jahra hospital from September 2020 to May 2021 were included in this study and followed prospectively.

**Results**: Twenty-nine pregnant women were in the ICU, 18 needed invasive ventilation. Out of those 18 patients, 10 required invasive ventilation, followed by ECMO. One patient died after abortion during ECMO and 9 patients discharged home. The median age was 32 years (IQR, 27 to 40) and a median Acute Physiology and Chronic Health Evaluation II score was 27 (IQR, 23 to 30). The gestational age at delivery was 33 weeks (range, 22 to 36 weeks). The indications for ECMO were severe COVID-19 ARDS (n = 8). One patient had massive pulmonary embolism, requiring VA-ECMO and local thrombolysis. All patients were endotracheally intubated for a median of 3 days (IQR, 2 to 5 days) before starting ECMO. Four patients delivered while on ECMO. One patient died who had reduced right ventricular function and was managed with VA-ECMO, dobutamine, and noradrenaline. One patient aborted her fetus while on ECMO, and nine patients underwent CS. The nine patients recovered and were successfully weaned from the combination of early mechanical ventilation and ECMO.

**Conclusions**: ECMO has been used successfully in pregnant women with severe COVID-19 and severe hypoxia in our ECMO center. The high maternal and fetal survival rates in this prospective observational study suggest that the benefits of managing maternal and fetal hypoxia due to COVID-19 with ECMO outweigh the potential risks.

## P185

### Long-term survival and health-related quality of life after veno-venous extracorporeal membrane oxygenation therapy in patients with severe acute respiratory distress syndrome

#### J. Rilinger^1^, K. Krötzsch^2^, X. Bemtgen^2^, M. Jäckel^2^, V. Zotzmann^2^, D. Duerschmied^2^, C. Bode^2^, D. L. Staudacher^2^, T. Wengenmayer^2^

##### ^1^Department of Cardiology and Angiology I, Heart Center Freiburg University, Freiburg, Germany; ^2^Heart Center Freiburg University, Freiburg, Germany

*Critical Care* 2021, **25**(**Suppl 1**): P185

**Introduction**: In severe acute respiratory distress syndrome (ARDS) patients veno-venous extracorporeal membrane oxygenation (VV ECMO) support is initiated when conventional measures fail. However, so far there is limited data about long time survival and especially long term quality of life (QoL) in these very sick patients.

**Methods**: We report retrospective data of a single-center registry of patients with severe ARDS requiring VV ECMO support between 10/2010 and 06/2019. Follow up of all patients that survived the index hospitalisation was performed by telephone interview. Survival and QoL (SF-36, St. Georges Respiratory Questionary (SGRQ) and Hospital Anxiety and Depression Scale (HADS)) were assessed.

**Results**: Of 289 VV ECMO patients, 129 patients (44.6%) survived the index hospitalisation. Follow up (4.3 ± 2.9 years after ECMO therapy) was successful in 94 patients, with 71 (75.5%) patients being still alive. Hospital survivors showed a high 6- and 12-month survival rate (89.4% and 85.1%, respectively, Fig. 1). QoL interviews were completed for 53 patients. Mean SF-36 score was 70.7, SGRQ score 19.3% and HADS score 7.4.

**Conclusions**: This analysis with a long follow up period showed very encouraging long term survival rates of VV ECMO therapy survivors. Moreover, SF-36, SGRQ and HADS showed a low level of remaining physically, respiratory and mentally limitations.

**Figure:**
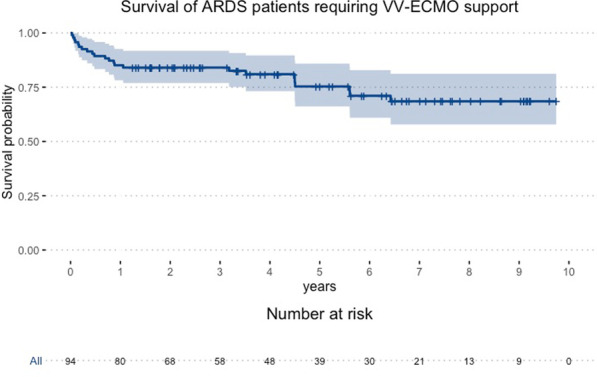
**Fig. 1** **(abstract P185)** Kaplan–Meier survival estimation for veno-venous extracorporeal membrane oxygenation therapy survivors

## P186

### Procedural characteristics of veno-venous extracorporeal membrane oxygenation patients with severe acute respiratory distress syndrome

#### J. Rilinger^1^, R. Book^2^, X. Bemtgen^2^, M. Jäckel^2^, V. Zotzmann^2^, D. Duerschmied^2^, C. Bode^2^, D. L. Staudacher^2^, T. Wengenmayer^2^

##### ^1^Heart Center Freiburg University, Department of Cardiology and Angiology I, Freiburg, Germany; ^2^Heart Center Freiburg University, Freiburg, Germany

*Critical Care* 2021, **25**(**Suppl 1**): P186

**Introduction**: Mortality remains high in patients with severe acute respiratory distress syndrome (ARDS) despite the use of veno-venous extracorporeal membrane oxygenation (VV ECMO). Understanding the differences between survivors and deceased patients is crucial for developing tomorrow’s therapy.

**Methods**: We report retrospective data of a single-center registry of patients with severe ARDS requiring VV ECMO support between 10/2010 and 04/2020. Vital parameters, laboratory values and blood gas analysis as well as the level of support of ECMO therapy, ventilator settings and catecholamine therapy of the first 10 days after ECMO cannulation were analyzed with regard to their association with survival.

**Results**: A total of 283 patients with complete medical data could be analyzed (age 53 years, 66% male). Overall ECMO weaning and hospital survival rate were 52.8% and 44.8%, respectively. Survivors showed a lower level of ECMO support after day 2 (blood flow and sweep gas flow) and day 3 (fiO2, p < 0.05, Fig. 1). Pulmonary compliance and tidal volume were higher in survivors after day 1. Moreover, survivors showed a lower noradrenaline dosage in the first 8 days and a lower fluid balance in all 10 days. Interestingly, positive end-expiratory pressure, driving pressure, respiratory rate, mean arterial pressure, lactate and bilirubin showed no or no clinical significant association to survival.

**Conclusions**: In this analysis of the first 10 days of ECMO therapy, there were major differences in the level of ECMO support, tidal volume and pulmonary compliance as well as fluid balance and required noradrenaline dosage between survivors and non survivors.

**Figure:**
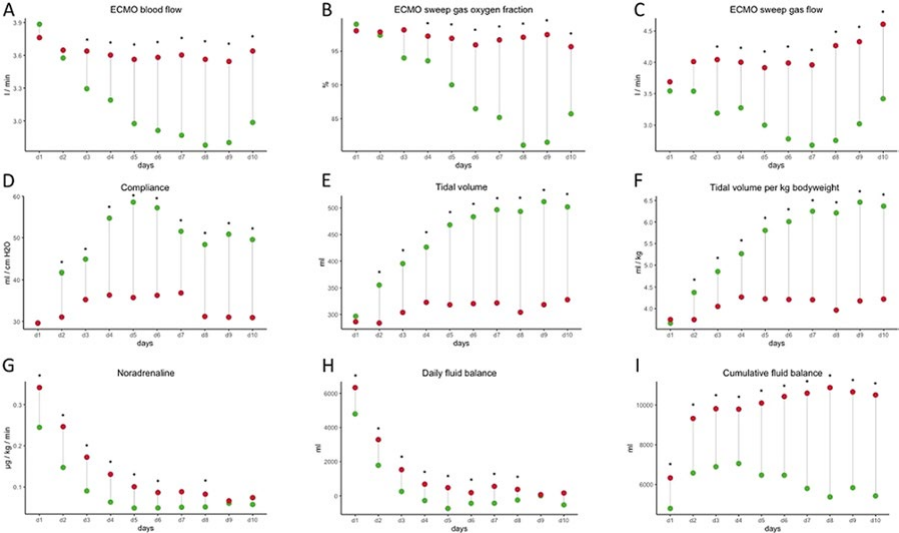
**Fig. 1** **(abstract P186)** Parameters associated with outcome in patients with ongoing VV ECMO therapy. Survivors (green) and non survivors (red) showed significant differences (*p < 0.05) in the level of ECMO support (A-C), pulmonary compliance and tidal volumes (D-F) as well as circulatory support (G-I) over the first 10 days of ECMO therapy

## P187

### Using inhalation volatile sedation for COVID-19 - easy, effective and safe

#### R. Knafelj, G. Govekar

##### Intensive Internal Medicine, University Medical Center Ljubljana, Ljubljana, Slovenia

*Critical Care* 2021, **25**(**Suppl 1**): P187

**Introduction**: Mechanical ventilation for ARDS COVID-19 is cornerstone supportive therapy. While prolonged ventilation is typically needed increasing doses of benzodiazepines, propofol, opioids and NMBAs are addmnistered. Drug clearance is decreased and prolonged awakening, weaning and delirium is seen. Also, in some countries shortage of fentanyl, midazolam and propofol has been reported [1]. Sevoflurane has active metabolites and tachyphylaxis, tolerence or dependance has not been described. It has predictable and favorable pharmacokinetics with anti-inflammatory effects demonstrated in patients with ARDS. No data is available on using sevoflurane for sedation in COVID-19. We assessed feasibility of sevoflurane sedation in COVID-19 patients requiring mechanical ventilation.

**Methods**: Thirty consecutive COVID-19 patients transferred to our ICU for advanced ventilatory support and/or rescue therapies were switched from midazolam and/or midazolam + propofol based sedation to sevofluran using Anaconda (Sedana Medical Sweden). Sedation depth was assessed using Richmond Agitation Sedation Scale (RASS) target -4 and Bispectral Index (BIS, Medtronic, US) target BIS 40–50. End expiratory sevoflurane concentration vas documented (Vamos, Dräger, Germany). All patients received continuous fentanyl and rocuronium.

**Results**: In cohort of 30 invasively ventilated patients 71% were male with BMI 29 ± 9 and APACHE 17 ± 8. 18 (60%) patients received iNO (20 ppm) (Fig. 1). Anaconda was placed in inspiratory limb in 3 (10%) patients.

**Conclusions**: Sevoflurane via Anaconda provided adequate dept of sedation in all COVID-19 patients. Dose needed for adequate dept of sedation was higher than reported in other patient populations. Less infusion pump manipulation was needed during sevoflurane only sedation. No adverse events occurred during sevoflurane administration. Further research is warranted.

**Reference**

1. Jerath A et al. Intensive Care Med 46:1563–1566, 2020

**Figure:**
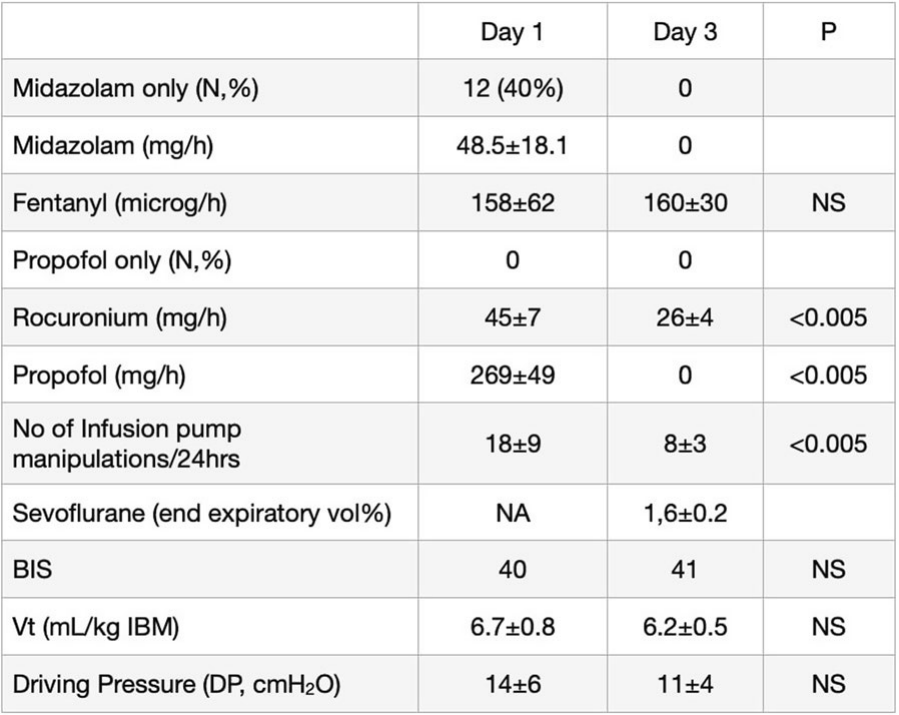
**Fig. 1** **(abstract P187)** Results. BIS: bispectral Index

## P188

### Ethical evaluation of sedation and analgesic practices during end of life care in South African intensive care units

#### R. Davids

##### Department of Anaesthesiology and Critical Care, Stellenbosch University, Cape Town, South Africa

*Critical Care* 2021, **25**(**Suppl 1**): P188

**Introduction**: Intensive care units are tasked with the mammoth responsibility of caring for the sickest patients. These patients are often in critical condition and despite best medical therapy, mortality remains high. The end of life scenario is commonplace in these units placing health care staff under immense occupational and emotional pressure. In South Africa these pressures are compounded by additional issues of financial constraints and limited resources on the background of great demand. The author explores the impact of these external factors on the ethical and moral standing of intensivists when contending with end of life decisions.

**Methods**: A cross-sectional, descriptive analysis was performed on data obtained from an anonymized questionnaire survey of healthcare workers in the intensive care unit. The questionnaire was developed and guided by similar studies and covers aspects of end of life decisions, sedation and analgesia and explores the pressures of the critical care environment. Face validation of the survey was performed. Ethical clearance obtained via Stellenbosch University REC 11,786. All intensivist responses were analyzed using SPSS version 21.

**Results**: Seventy-nine South African intensivists participated in the survey. 82% of respondents indicated severe patient burden and bed pressure. More than 90% of respondents indicated that they found it morally acceptable to administer sedation and analgesia despite the anticipated hastened death (Fig. 1). These intensivists go on to admit that bed pressure and financial constraints factor into their end-of-life care decision making.

**Conclusions**: The moral arguments regarding hastening death and euthenasia is unearthed in the South African intensive care arena, as the results of the survey uncovers the strain of severe bed pressure and financial constraints faced by intensivits. These entities are admittedly factored into end-of-life decision-making, particularly in terms of sedation and analgesic practice, in which hastening death is viewed positively by this cohort.

**Figure:**
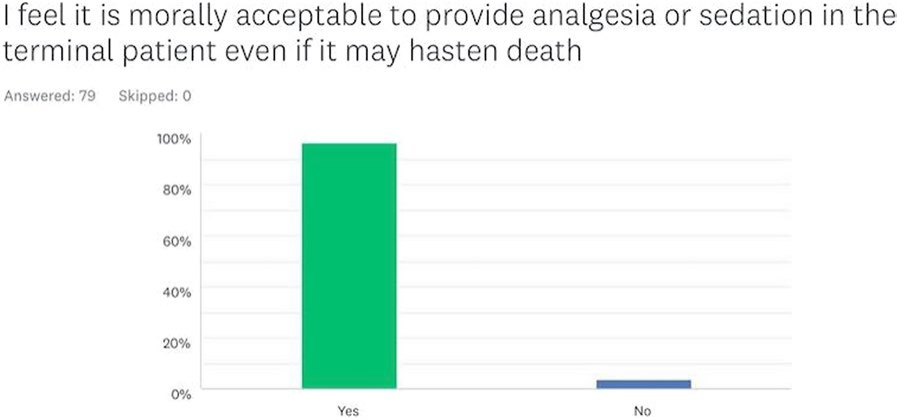
**Fig. 1** **(abstract P188)** Moral acceptance of analgesia or sedation in hastening death

## P189

### Over-sedation as a risk factor for delirium in critically-ill COVID-19 and non COVID-19 patients with ARDS: a dual center analysis

#### M. Marchesi^1^, F. Rasulo^1^, S. Calza^2^, S. Cattaneo^3^, B. Matta^4^, D. Cunningham^4^, S. Piva^5^, M. Filippini^5^, S. Beretta^5^, N. Latronico^5^

##### ^1^Department of Anesthesiology and Intensive Care, ASST Spedali Civili di Brescia, Brescia, Italy; ^2^Health Statistics, ASST Spedali Civili di Brescia, Brescia, Italy; ^3^Cardioanesthesiolgy and Cardiac ICU, ASST Spedali Civili di Brescia, Brescia, Italy; ^4^Anesthesiology and Intensive Care, Addenbrooke´s Hospital, Cambridge, UK; ^5^Anesthesiology and Intensive Care, ASST Spedali Civili di Brescia, Brescia, Italy

*Critical Care* 2021, **25**(**Suppl 1**): P189

**Introduction**: Over-sedation is an important issue for critically ill patients, and has been associated with increased mortality and morbidity, including delirium. Patients with acute respiratory distress syndrome (ARDS) frequently require deep sedation and neuromuscular blockade to optimize mechanical ventilation.

**Methods**: We assessed the incidence of over-sedation and delirium in COVID-19 and non COVID-19 patients and sought any differences between those with and without ARDS. Depth of sedation was monitored through use of continuous processed EEG. Delirium was evaluated through use of the CAM-ICU. The main outcomes were the incidence of over-sedation and delirium and the correlation between them in COVID-19 and non COVID-19 patients with and without ARDS. A total of 78 patients were included into the study, 38 of whom ad ARDS, 21 of whom had COVID-19 disease.The mean monitoring time for the COVID-19 and the non-COVID-19 patients was respectively 43 and 50 h. Thirty-eight (49%) of the total 78 patients fulfilled the criteria for over-sedation.

**Results**: There was a statistically significant association between the incidence of delirium and over-sedation (OR 11.4, p < 0.001) and age (OR 1.04, p = 0.017) (Fig. 1). When adjusting for age, over-sedation was still significantly associated to delirium (OR 8.35, p = 0.002). COVID-19 patients showed a non-significant higher percentage of delirium (92.3% vs 63.4%, p = 0.076).Although non-significant, there was a higher incidence of over-sedation (60.5% vs 37.5%, p = 0.069), and delirium (84% vs 58.6%, p = 0.07) in ARDS patients.

**Conclusions**: This study shows that, besides age, over-sedation represents an important risk factor for delirium in mechanically ventilated patients, and that over-sedation and delirium were more common in ARDS vs non ARDS patients. It supports the use of continuous EEG-based monitoring systems for the quantification of sedation depth and highlights the necessity for larger, randomized trials to verify whether monitoring the depth of sedation can improve outcome.

**Figure:**
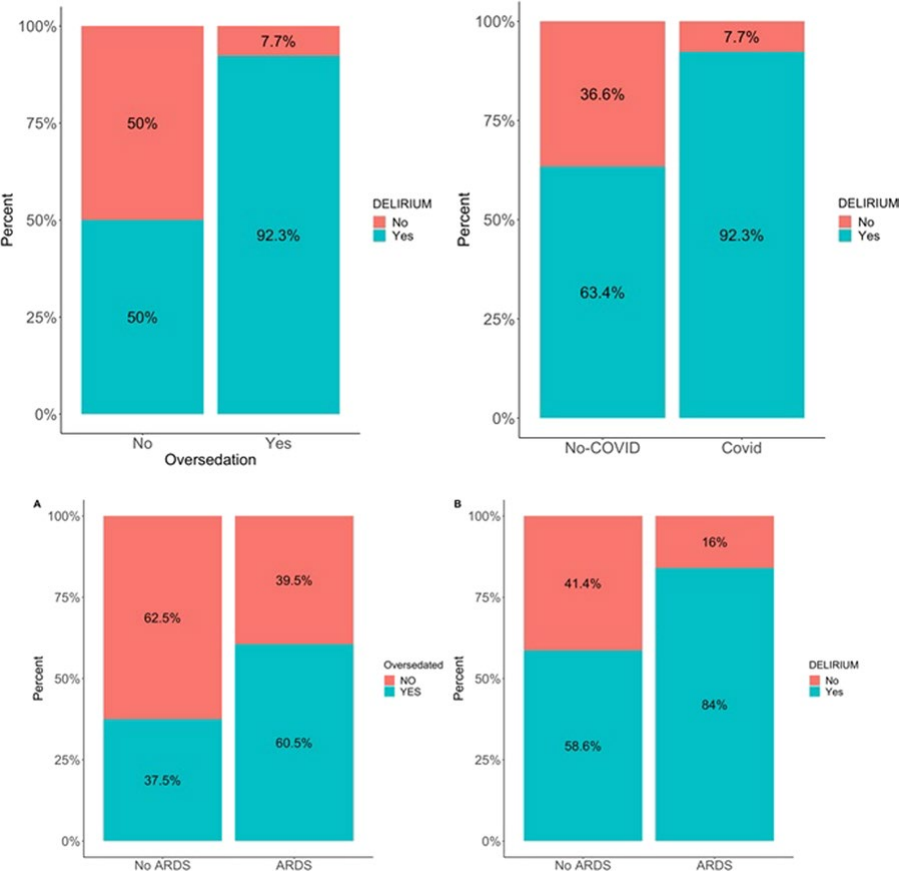
**Fig. 1** **(abstract P189)** Incidence of oversedation and delirium in COVID-19 vs non-COVID-19and ARDS vs non-ARDS patients

## P190

### Endogenous glucose production and gluconeogenesis: are they predictable? One more small step toward personalized medicine

#### I. U. Udin^1^, M. H. Habisreutinger^2^, L. T. Tappy^3^, A. S. Schneider^1^, M. B. Berger^1^

##### ^1^Intensive Care Unit, CHUV, Lausanne, Switzerland; ^2^Computational Fluid Dynamics, EPFL, Lausanne, Switzerland; ^3^Institute of Physiology, University, Lausanne, Switzerland

*Critical Care* 2021, **25**(**Suppl 1**): P190

**Introduction**: Feeding a patient the closer to his/her needs is a challenge in the intensive care unit (ICU), avoiding both under- or over-feeding and their associated complications and mortality [1–3]. But ICU patients have an unrepressed endogenous glucose production (EGP) [4, 5], an energy source not considered by classical energy balance. This study aims at identifying clinically available variables predictive of EGP and gluconeogenesis (GNG), which otherwise need isotopic tracers.

**Methods**: This exploratory study is based on the data from the Supplemental Parenteral Nutrition study 2 (SPN2) [5], which measured EGP and GNG at days 4 and 10 in 23 ICU patients. The correlations between EGP and GNG and 83 potential clinical indicators were explored, using single-stage and multivariate analysis.

**Results**: On single-stage analysis, the strongest correlations were norepinephrine dose at day 4 with GNG (R = 0.71; p = 0.0004), and at day 10, VO_2_ with GNG (R = 0.59, P = 0.04), and VCO_2_ with EGP (R = 0.85, p = 0.00003). Cumulated insulin dose between days 5 and 9 was correlated to EGP at day 10 (R = 0.55, p = 0.03). Our multivariate model could predict EGP at day 4 with an error coefficient (EC) between 7.8% and 23.4% (minimal and maximal error), and GNG at day with an EC of 18.5% and 29.9%. GNG at day 4 and EGP at day 10 could not be predicted with an EC < 40%.

**Conclusions**: This preliminary study shows that GNG and EGP have different predictors on days 4 and 10; EGP is more correlated with the metabolic level, while GNG is dependent on external factors. Some variables could be identified to assess the magnitude of both values. Our results suggest that a robust model might be built, but require a prospective study including a larger number of patients.

**References**

1. Zusman O et al. Crit Care 20:367, 2016
2. Dissanaike S et al. Crit Care 11:R114, 2007
3. Yeh DD et al., JPEN 40:37–44, 2016
4. Tappy L et al., Crit Care Med 26:860–7, 1998
5. Berger MM, Clin Nutr 38:2408–2416, 2019

## P191

### Using machine learning to compare gastric residual volume thresholds as predictors of clinical outcomes in critically ill patients

#### O. Raphaeli^1^, C. Hajaj^2^, I. Bendavid^1^, A. Goldstein^2^, E. Chen^2^, P. Singer^1^

##### ^1^Intensive Care Unit, Beilinson hospital, Petah Tikva, Israel; ^2^Industrial Engineering and Management, Ariel University, Ariel, Israel

*Critical Care* 2021, **25**(**Suppl 1**): P191

**Introduction**: The aim of the study is to examine the association between feeding intolerance (FI), defined by different gastric residual volume (GRV) thresholds, and clinical outcomes in enterally fed critically ill patients. “Large” GRV is a key criterion used in most FI definitions, yet due to conflicting evidence, the determination of the optimal GRV threshold remained an unresolved issue.

**Methods**: We included adult patients (2012–2018) admitted at Beilinson hospital ICU for more than 48 h. FI definition is based on the occurrence of “large” gastric volumes (thresholds of 150, 250 and 500 ml), GI symptoms and “inadequate” delivery of enteral nutrition [1]. Admission conditions and FI occurrences, along 72 h, were analyzed by machine learning classification algorithms predicting mortality and morbidity. Prediction performance was assessed by the area under the curve (AUROC) of ten-fold cross-validation and validation sets for 3 GRV thresholds.

**Results**: The dataset comprised of 1782 enterally fed patients. The median (IQR) age was 62 (48–72) years, BMI 26.5 (23–31). Main admission conditions: surgical (47%), trauma (27%) and medical (25%). Five algorithms were trained and tested (Python software). The best performing algorithm was Random Forest classifier. Models with GRV threshold of 250 ml achieved the best results (AUC = 0.82–0.87, depending on outcome metric), followed by models using GRV threshold of 150 ml (AUC = 0.81–0.86) and models using GRV threshold of 500 ml (AUC = 0.76–0.85). Valuable predictors in the models were GRV > 250 ml or GRV > 150 ml in 72 h.

**Conclusions**: FI occurrences along 72 h of ICU admission, using GRV threshold of more than 250 ml, have the best performance in predicting clinical outcomes in enterally fed critically ill patients.

**Acknowledgments**: This study was funded by Fresenius-Kabi.

**Reference**

1. Reintam-Blaser et al., Nutr Clin Pract 36:1, 2021

## P192

### A handheld device to detect CO_2_ after insertion of nasogastric feeding tubes

#### J. Turnbull, K. Williams, V. Waugh, J. Fernandez Roman, D. Shaw, I. Welters

##### Royal Liverpool intensive Care Unit, Liverpool University Hospitals NHS Foundation Trust, Liverpool, UK

*Critical Care* 2021, **25**(**Suppl 1**): P192

**Introduction**: Erroneous nasogastric tube (NGT) placement and subsequent feeding can be fatal. Current techniques to identify NGT position focus on pH measurement of gastric aspirate. A device combining pH testing and CO_2_ detection could reduce the need for chest radiographs (CXR), improve patient safety and save resources. Here we report first results of a handheld point of care device to demonstrate usability of CO_2_ detection.

**Methods**: NGTs were inserted in 30 patients in the Royal Liverpool University Hospital intensive care unit. Data collection included: conventional pH measurement of aspirate (if obtained) and CO_2_ detection using the DoubleCHEKTM device (https://enteralaccesstech.com). NGT location on CXR was also recorded. Users were asked to complete an evaluation form containing device experience questions and allowed free-text comments.

**Results**: Evaluation of the device was completed in 29/30 (96.67%) of cases. Aspirates were obtained in 14/30 (46.67%) cases with 7/14 (50%), resulting in a pH of < 5.5 as measured by conventional pH strips. Of the 16/30 (53.33%) insertions yielding no aspirate, 3/16 (18.75%) were clinically located in the lungs. In these insertions, CO_2_ was detected, the NGT was removed, and a second insertion attempt was successful as indicated by CXR or pH < 5.5. Of the 7/30 (23.33%) cases in which aspirates were obtained, but pH reading was > 5.5, six of seven (85.71%) CXRs showed that NGTs were in the stomach, and no CO_2_ was detected with the DoubleCHEKTM device. Only 2/29 (6.8%) users would object to using the device in daily practice.

**Conclusions**: Simultaneous pH and CO_2_ detection is a promising method to avoid misplacement of NGTs. Difficulties in obtaining aspirates and high pH are common problems in NGT placement [1]. Further clinical trials are warranted to determine sensitivity and specifity of the technique compared to standard protocols and to evaluate the cost-saving potential.

**Reference**

1. Pailsey et al. Crit Care 21:P69, 2017

## P193

### Comparative effects of intermittent vs continuous enteral feeding and time of day on glycemic variability in critically ill patients

#### E. Termote^1^, L. S. Chapple^2^, M. Horowitz^3^, M. J. Chapman^1^, I. W. Kouw^2^

##### ^1^Intensive Care Unit, Royal Adelaide Hospital, Adelaide, Australia; ^2^Discipline of Acute Care Medicine, The University of Adelaide, Adelaide, Australia; ^3^Centre of Research Excellence in Translating Nutritional Science to Good Health, The University of Adelaide, Adelaide, Australia

*Critical Care* 2021, **25**(**Suppl 1**): P193

**Introduction**: In critical illness, increased glycaemic variability is associated with greater mortality and morbidity [1] and the relationship with the mode and timing of enteral nutrition (EN) delivery are unknown [2]. We assessed the effect of intermittent vs continuous EN delivery and delivery time on glycemic variability.

**Methods**: Preliminary data of an active parallel, single-center, single-blinded, trial in non-diabetic ICU patients (age ≥ 18 y; receiving or eligible to receive nasogastric EN), who were randomized to 24 h of isocaloric EN delivered intermittently (INT: 6-hourly bolus, T1 = 8:00 h, T2 = 14:00 h, T3 = 20:00 h, T4 = 2:00 h) or continuously (CONT) were analyzed. Subcutaneous continuous glucose monitoring (DEXCOM G6) was used to assess glycemic variability (standard deviation; SD, coefficient of variation; CV, and mean absolute glucose concentrations; MAG) and 6 h postprandial blood glucose levels (area under curve; AUC) were calculated. Variables were compared between EN mode and over time. Data are expressed as mean ± SD.

**Results**: Groups were not different at baseline (INT vs CONT: n = 11 vs 9; age: 49 ± 16 vs 55 ± 11 y; BMI: 31 ± 11 vs 32 ± 6 kg/m^2^; APACHE II: 19 ± 9 vs 16 ± 5). Glycemic variability over 24 h (SD: 1.3 ± 0.6 vs 1.0 ± 0.5 mmol/l; CV: 17.8 ± 10.5 vs 14.2 ± 3.9%; MAG: 1.7 ± 0.8 vs 1.3 ± 0.3 mmol/l) did not differ between INT and CONT, respectively (Fig. 1; all p > 0.05). In INT, postprandial peak glucose levels were higher at T4 (10.7 ± 2.3 mmol/L) than T1 (9.8 ± 2.2 mmol/l, p = 0.02) and T2 (9.5 ± 2.6 mmol/l, p = 0.01), while the postprandial rise in glucose did not differ between bolus times (all p > 0.05). Postprandial glucose AUC was higher in both T3 and T4 when compared to T1 and T2 (all p < 0.05).

**Conclusions**: In critical illness, intermittent delivery of EN is likely not associated with increased glycaemic variability compared to continuous feeding and the glycemic response to intermittent EN appears to be increased at night.

**References**

1. Eslami S et al. Intensive Care Med 37:583–93, 2011.
2. McNelly AS et al. Chest 158:183–94, 2020.

**Figure:**
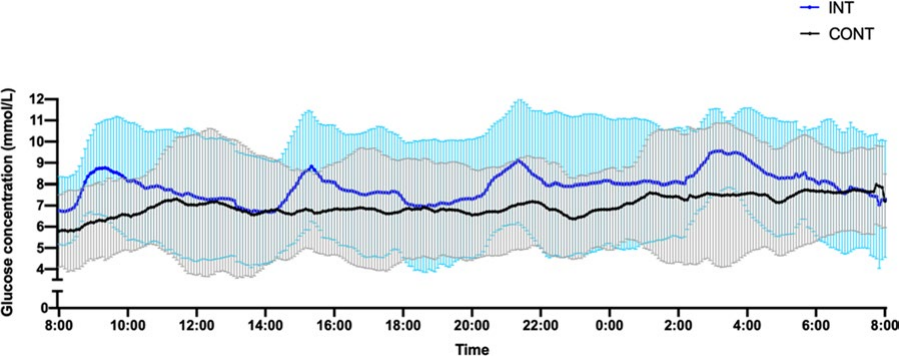
**Fig. 1** **(abstract P193)** Interstitial glucose concentrations during 24 h of intermittent (INT) or continuous (CONT) isocaloric enteral nutrition in 20 critically ill patients

## P194

### Previous ICU stay increases the probability to receive enteral or parenteral nutrition in the ward: a multivariate nutritionDay analysis of 191,886 adult patients

#### M. Hiesmayr^1^, B. Zapletal^2^, A. Lassnigg^3^, S. Tarantino^4^, E. Pestana^5^, A. Roberts^5^, A. Laviano^6^, C. Veraar^3^, A. Fischer^3^, P. Singer^7^

##### ^1^Dept of Cardiothoracic Anesthesia & Critical Care, Medical University Vienna, Vienna, Austria; ^2^Medical University Vienna, Vienna, Austria; ^3^Division Cardiac Thoracic Vascular Anaesthesia and Intensive Care, Medical University Vienna, Vienna, Austria; ^4^CEMSIIS, Medical University Vienna, Vienna, Austria; ^5^Market Access & Education Business Unit Enteral Nutrition - Pharmaceuticals Division Fresenius-Kabi, Scientific Affairs, Bad Homburg, Germany; ^6^Department of Clinical Medicine,Universita La Sapienza, Rome, Italy; ^7^General Intensive Care, Rabin Medical Center, Tel Aviv University, Tel Aviv, Israel

*Critical Care* 2021, **25**(**Suppl 1**): P194

**Introduction**: Continuity of care has become an important concern in patients after an intensive care stay. We analyzed in the adult nutritionDay cohort 2006–2019 whether a previous ICU stay is a risk factor for receiving enteral (EN) or parenteral nutrition (PN) in the ward.

**Methods**: The nutritionDay cohorts 2006–2015 (n = 153,470) and 2016–2019 (n = 38,416) were analyzed separately with uni- and multivariate GLM logistic regression with length bias correction for cross-sectional sampling and units as random factors (STATA 15.1). Included risk factors are sex, age, surgical status, duration of hospital stay before nutritionDay, BMI, self-rated health, fluid status, weight change within the last 3 month, mobility and amount eaten on nutritionDay. Data are reported as OR with 95% confidence intervals.

**Results**: In 191,886 adults patients from 67 countries, a previous ICU stay was documented in 16,636 patients (10.8%) and in 4127 patients (10.7%) in cohort 1 & 2. A previous ICU stay was most prevalent for postoperative patients 21.3% & 19% and lowest in non-surgical patients 6.8% & 7.7%. Patients with a previous ICU stay were more often men 56% vs 49%, less often older > 80a 18% vs 23% or younger < 40a 10% vs 12%, but similar in BMI and self-rated health. Only eating all was slightly lower by 5% in patients with an ICU stay whereas eating half, quarter or nothing was similar. Patients with a previous ICU stay had more than twice as often EN 13 vs 7 and 13 vs 4% in cohort 1 & 2 (p < 0.001) and PN 8 vs 4% in both cohorts. The OR for EN and PN was strongly affected by BMI, self-rated health and actual eating (Fig. 1 Cohort 2). PN was not driven by a previous ICU stay in cohort 2. Patients with a previous ICU stay and eating nothing received EN or PN in 55% and 60% compared with 30% and 31% in those without ICU stay (p < 0.001).

**Conclusions**: After an ICU stay, artificial nutrition is used twice as often than in patients without an ICU stay but patient appearance (BMI and subjective health) appears to be a strong independent factor.

**Figure:**
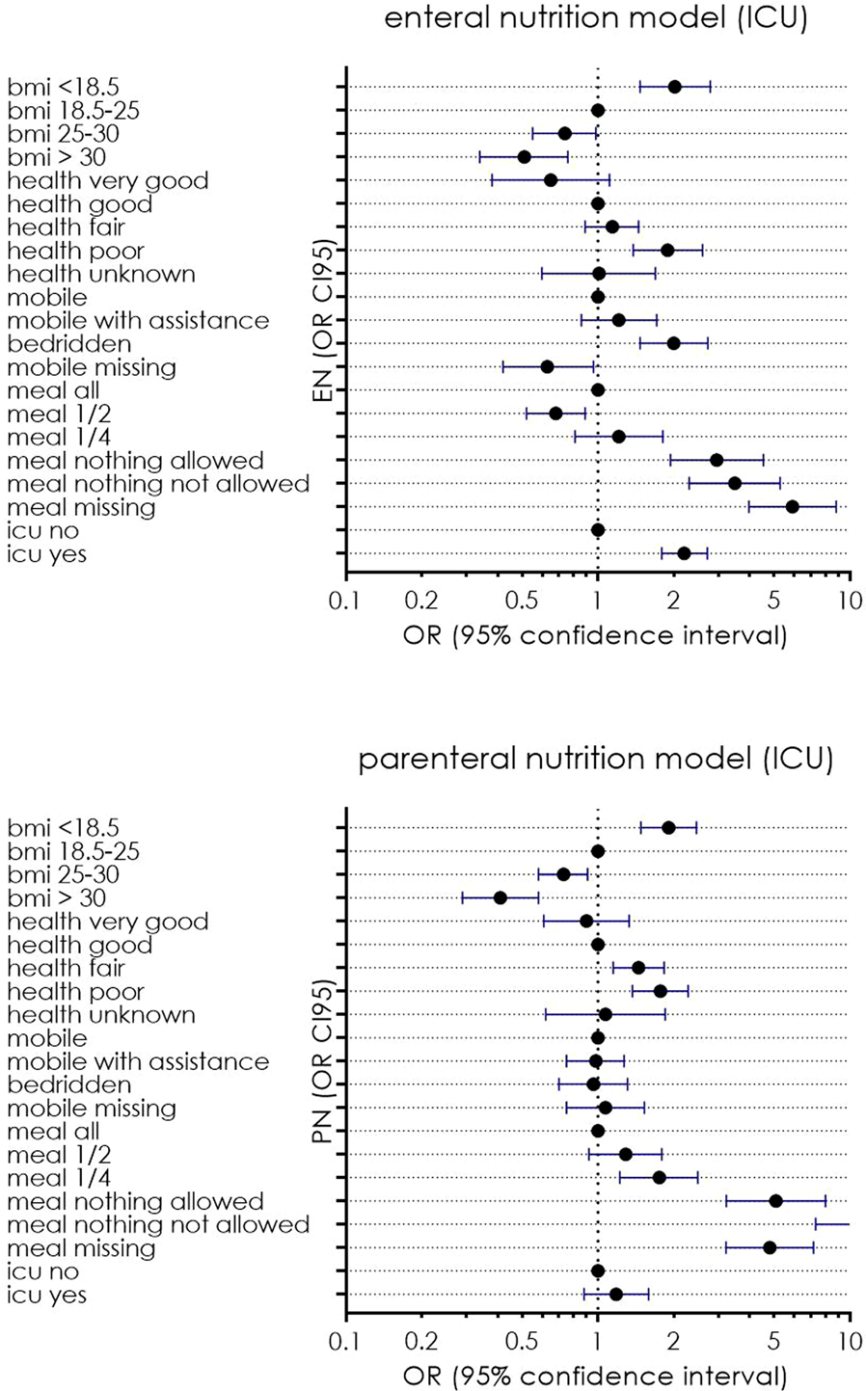
**Fig. 1** **(abstract P195)** Enteral and parenteral nutrition use multivariate analysis of patients after a previous ICU stay in the nutritionDay cohort 2 (n = 38,416)

## P195

### Preferential use of enteral or parenteral nutrition in the world regions: a multivariate nutritionDay ICU analysis in 16,032 patients

#### M. Hiesmayr^1^, S. Tarantino^2^, I. Sulz^2^, C. Veraar^3^, A. Fischer^3^, E. Pestana^4^, A. Roberts^4^, A. Lassnigg^3^, A. Laviano^5^, P. Singer^6^

##### ^1^Dept of Cardiothoracic Anesthesia & Critical Care, Medical University Vienna, Vienna, Austria; ^2^CEMSIIS, Medical University Vienna, Vienna, Austria; ^3^Division Cardiac Thoracic Vascular Anaesthesia and Intensive Care, Medical University Vienna, Vienna, Austria; ^4^Scientific Affairs, Market Access & Education Business Unit Enteral Nutrition - Pharmaceuticals Division, Fresenius-Kabi, Bad Homburg, Germany; ^5^Department of Clinical Medicine,Universita La Sapienza, Rome, Italy; ^6^General Intensive Care, Rabin Medical Center, Tel Aviv University, Tel Aviv, Israel

*Critical Care* 2021, **25**(**Suppl 1**): P195

**Introduction**: Controversy exists between ESPEN and ASPEN guidelines on the indication for enteral (EN) versus parenteral (PN) nutrition. The aim of the study is the comparison between world regions in preference of enteral over parenteral nutrition after adjustment for patient characteristics.

**Methods**: All 16,032 patients from the nutritionDay project 2007–2018 are included in the analysis. World region are defined according to WHO definition: EUR A (Central & Western Europe), EUR B (Eastern Europe), AMR A (North America), AMR B (South America), Eastern Mediterranean (UA) and Asia. Adjustment risk factors: age, sex, BMI, SAPS2, SOFA, renal replacement therapy, ventilation, duration of ICU stay before nutritionday and reason for ICU dependency. Preference for either EN or PN was analyzed with logistic regression with ICUs as clusters. Statistical analysis was done in R 3.3.1.

**Results**: The majority of patients were adults (98.5%), male (63%) with a median age of 64 IQR [51–74], BMI 25.5 [22.6–29.3], SAPS2 38 [27–52), SOFA 4 [1–4]. Observed mortality in hospital was 24% within 60 days. In Europe 39% of patients receive EN either alone or in combination with PN and 27% PN. One out of four patients are on oral nutrition whereas one out of seven receive EN or PN in addition to oral nutrition. Outside Europe more patients were on EN, PN was used about half as frequently and oral nutrition was more prevalent. The preference for EN was independent of age, BMI and sex. EN became more prevalent with duration of ICU stay. Reason for ICU admission neurological and cardiac increased the preference for EN and abdominal reasons for PN. Americas and Asia preferred EN compared with both European regions. EN was more prevalent with increasing severity of illness at admission. More PN if on CRRT and more EN if ventilated (Fig. 1).

**Conclusions**: Preference for EN over PN is strongly associated with world regions after adjustment for multiple patient factors.

**Figure:**
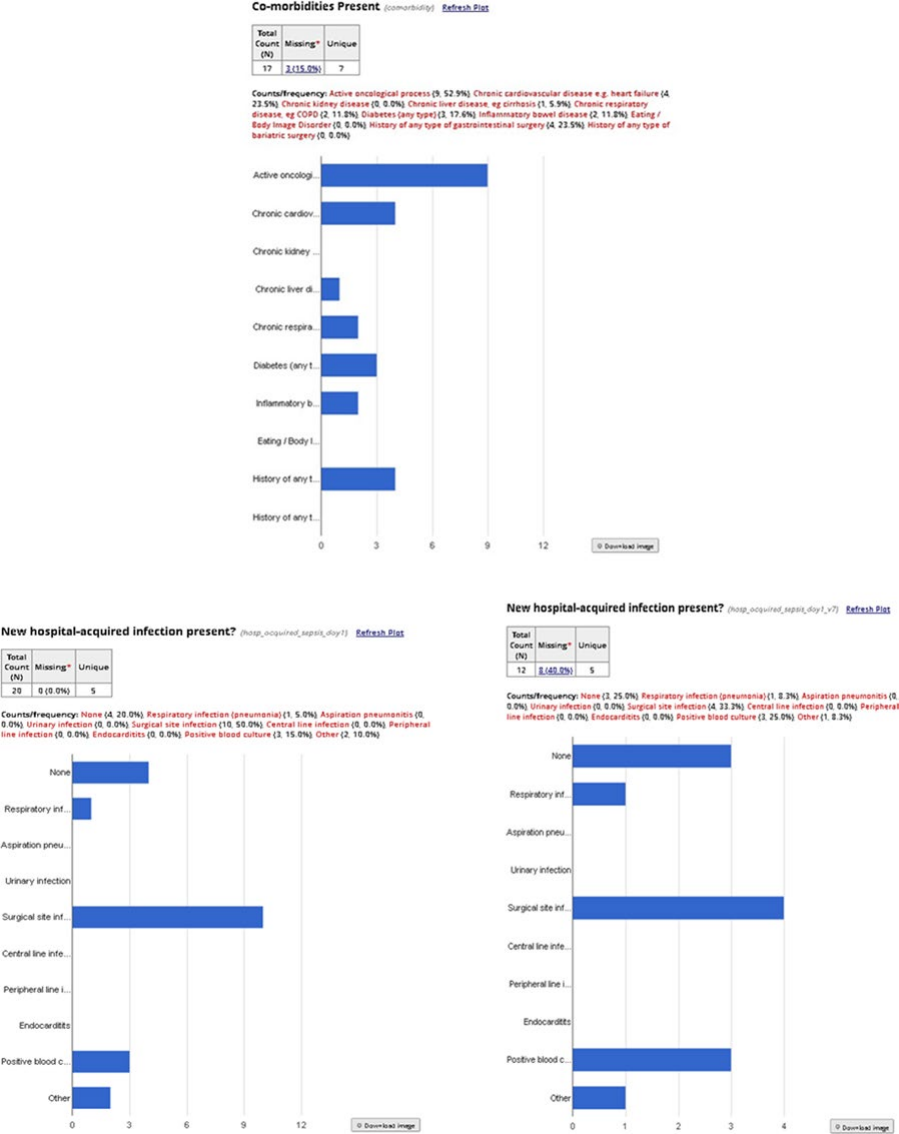
**Fig. 1** **(abstract P196)** Preferential use of enteral (OR < 1) or parenteral (OR > 1) nutrition in the nutritionDay cohort 2007–2018 (n = 16,032)

## P196

### Evaluating the parenteral nutrition team service at a major tertiary-teaching hospital

#### A. S. Shahsavand^1^, G. D. Duke^2^, S. H. Hunter^2^, L. B. Breik^2^

##### ^1^Pharmacy, Eastern Health - Boxhill Hospital, Boxhill, Australia; ^2^Intensive Care Unit, Eastern Health - Boxhill Hospital, Boxhill, Australia

*Critical Care* 2021, **25**(**Suppl 1**): P196

**Introduction**: Patients that receive parenteral nutrition (PN) are at higher risk of infections, metabolic and liver derangement and having an effective interdisciplinary parenteral nutrition team (PNT) is crucial to optimal nutrition care for this vulnerable population group. This research was undertaken in a 430-bed university-affiliated metropolitan public hospital with 25,000 overnight (and 55,000 day-case) admissions per annum, and the Millenium™ electronic medical records system.This PNT consists of an intensive care unit (ICU) nurse liaison, an intensivist and a surgical dietician.

**Methods**: Patient data were interrogated from day one until day seven of PN therapy. Process measures included reasons for PN initiation, PN delivery data, and reasons for cessation or changes of PN.

**Results**: During the 1-month study period 20 records were audited and 19 patients met the indications for PN initiation. Mean age was 66 ± years and 55% were male and 89% had serious comorbidities (Fig. 1). Median PN duration was 5 days and 86 total PN bed-days. 79% were receiving antibiotic treatment for acute infection on Day-1. Mild hepatic dysfunction was observed in 59%with GGT > 71 mmol/l; 46% with ALP > 110 mmol/l; 23% with ALT > 40 mmol/l. Caloric requirements were estimated at 30–35 kcal/kg/day and 90% met recommended daily energy requirements from PN alone. Electrolyte disorders included hypokalemia in 9%, 9% with hyponatremia 6.5% episodes of hypernatremia. Outcome of fluid overload throughout the audit is lacking as fluid balance for 45% of participants were not measured.

**Conclusions**: This audit highlights that blood glucose management, triglyceride checking and strict fluid balances are required for PN patients. The parenteral nutrition team displayed consistent commencement practices but strategies are needed to minimize metabolic complications arising from PN administration. The results of this study have been shared with PNT team with ongoing strategic plans to improve the PN service at this center.

**Figure:**
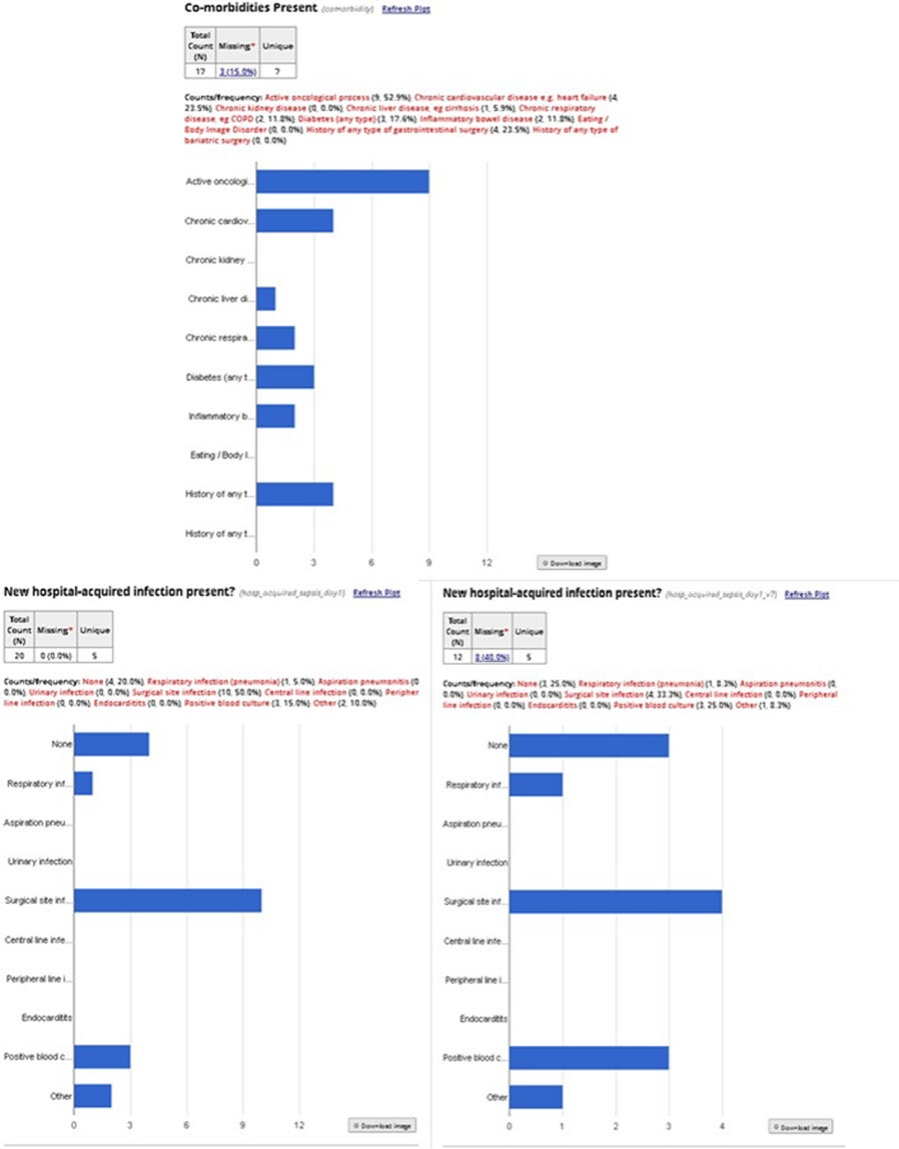
**Fig. 1** **(abstract P197)** Results

## P197

### Clinical manifestation and diagnosis of patients with thyroid storm: a retrospective study

#### M. Akatsuka^1^, S. Yamamoto^2^

##### ^1^Department of Anesthesiology, Obihiro Kosei Hospital, Sapporo, Japan; ^2^Obihiro Kosei Hospital, Sapporo, Japan

*Critical Care* 2021, **25**(**Suppl 1**): P197

**Introduction**: Thyroid storm (TS) is an endocrine emergency, which is a rare and life-threatening condition. It occurs suddenly leading to a high mortality rate if not recognized immediately and treated aggressively. However, due to its rare incidence, there is a lack of information about the manifestations of TS. The present study aimed to evaluate important aspects of epidemiology, diagnosis, and prognosis of TS.

**Methods**: We conducted a retrospective observational study on patients diagnosed with thyroid storms in our hospital between January 2011 and December 2020. The definition of TS was used by the Japanese Thyroid Association criteria [1]. We obtained information on the patients’ clinical characteristics and outcome from medical records.

**Results**: Six patients were included in this study. The median and the interquartile range (IQR) of age were 45 [41–52] years old. The SOFA score on ICU admission was 3.5 [3.0–4.8] and the Burch-Wartofsky Point Scale was 65 [60–81]. The thyroid function test revealed free triiodothyronine (fT3) level of 13.58 [9.78–20.00] pg/mL (normal range: 2.3–4.0), free thyroxine (fT4) level of 6.00 [4.41–6.53] ng/dL (normal range: 0.9–1.7), and thyroid-stimulating hormone (TSH) < 0.003 μIU/mL (normal range: 0.5–5.0). All patients exhibited abnormal tachycardia and altered mentation. The symptoms of the patients mostly included fever, nausea, and vomiting. The most common cause (83.3%) of the TS was self-interruption of the anti-thyroid drug which is used to treat hyperthyroidism. One case exhibited diabetic ketoacidosis with TS. The 28-day mortality of all patients was found to be 33.3%.

**Conclusions**: TS needs an early diagnosis and an aggressive treatment with the appearance of its symptoms to prevent worsening of the condition. Additionally, efforts should be made to maximize patient compliance to anti-thyroid agents used for the treatment of such patients.

**Reference**

1. Akamizu T. Thyroid 28:32–40, 2018

## P198

### Critical and alert value with pH lower than 7.2

#### M. De la Torre-Prados^1^, A. García-de la Torre^2^, J. Diaz-Ojeda^3^, M. C. Navarrete-Ortiz^4^, C. Ortiz-García^2^, P. Trillo-López^5^

##### ^1^Facultad de Medicina, Universidad de Málaga, Departamento de Medicina, Málaga, Spain; ^2^Laboratorio de Análisis Clínico y Bioquímica, Hospital Universitario Virgen de la Victoria, Málaga, Spain; ^3^Servicio de Tecnología de la Información y Comunicaciones, Hospital Universitario Regional, Málaga, Spain; ^4^Instituto de Investigación Biomédica de Málaga (IBIMA), Fundación Pública Andaluza para la Investigación de Málaga en Biomedicina y Salud (FIMABIS), Málaga, Spain; ^5^Estrategia Seguridad Paciente Sistema Sanitario Público Andaluz, Hospital Universitario Regional, Málaga, Spain

*Critical Care* 2021, **25**(**Suppl 1**): P198

**Introduction**: The generation of an automated alert [1] in Laboratory Information System (LIS) addressed to the applicant after validation critical pH value (CpHV) < 7.2 would ease optimal clinical performance.

**Methods**: Intervention study with Phase 1 or historical control from 1 / 2018 to 12 / 2018, including ward area and emergency patients > 14 years and CpHV < 7.2. Demographic, clinical and time variables were recorded in different clinical actions. The program used for data processing, descriptive and comparative statistical analysis was carried out with SPSS version 20.0, according to the nature of variables and study objectives. Project approved by the Ethics and Research Committee.

**Results**: Of 46,141 values performed in the emergency laboratory, 872 (4.37%) had CpHV < 7.2; 165 samples were randomly. 58.2% were male and 68.9 years ± 15.27 years. The location with the 1st CpHV < 7.2 was Emergencies 59.4%, (n = 98), Critical Area 29.1% (n = 48) and ward area 11.5% (n = 19). The pathology (P) related to CpHV < 7.2 was Cardiac P (28.5%, n = 47), sepsis (14.5%, n = 24), Respiratory P (14%, n = 23), Digestive (12%, n = 20) and other P (31%). Mortality was 45.5% (n = 75), it had differences with 1st CpHV healthcare area: Critical Area 74% (n = 23) vs Emergency 40% (n = 39), Chi2 = 23.3, p = 0.001; age: 74.6 years vs. 64 years, 95% CI 6.01–14.9, T-t = 4.6, p < 0.001; Charlson: 5.3 vs 3.8, 95% CI 0.78–2.1, T-t = 4.2, p < 0.001; SOFA: 9.4 vs. 5.5 95% CI 2.84–4.88, T-t = 7.4, p < 0.001; number of dysfunctional organs: 3.6 vs. 2.4, 95% CI 0.81–1.56, T-t = 7; p < 0.001; pH repetition delay: 7.3 vs 6.7 h, p = ns; delay first clinical performance: 2.6 vs. 1.5 h, p = ns.

**Conclusions**: It can be stated that pH < 7.2 should be included as a critical value parameter in the LIS in order to improve the different therapeutic actions and the survival of these patients, mainly in time-dependent pathologies.

**Reference**

1. Escobar GL et al. N Engl J Med 2020; 383:1951–1960.

## P199

### Intensive care essentials: reflections on 3 years of a one-day course for introduction to critical care

#### E. Collins^1^, S. Kwok^1^, D. Melia^2^

##### ^1^Whipps Cross University Hospital, London, UK; ^2^Dept of Anaesthesia and Critical Care, Whipps Cross University Hospital, London, UK

*Critical Care* 2021, **25**(**Suppl 1**): P199

**Introduction**: In 2018 a teaching program for doctors starting their first rotation in critical care was created at Whipps Cross Hospital; Intensive Care Essentials (ICE), covering basic knowledge and practical skills [1]. Here, we reflect on its evolution, evaluate feedback and assess whether it is still fit for purpose.

**Methods**: The program is delivered as a mixture of lectures and practical workshops. It aims to introduce core critical care topics, helping to alleviate anxiety felt by new starters. Initially the attendees were new doctors, but it has since been expanded to include new nursing staff. The themes covered remain unchanged: mechanical ventilation, non-invasive ventilation, cardiac output monitoring, inotropes and vasopressors, sedation and delirium, renal replacement therapy, and airway management, but the content within each theme has been modified according to feedback from previous courses. This year, we have also had to make adjustments to enable smaller groups in line with covid social distancing advice. Attendees were sent a pre- and post-course questionnaire, asking them how strongly they agreed with certain statements using a Likert scale (1-strongly disagree, 5-strongly agree). Unpaired t-tests were used to compare pre- and post-course results, and these were in turn compared to data from previous courses.

**Results**: Feedback from 2021 (Table 1) demonstrated that after the course, attendees felt significantly more prepared for ICU clinical work (mean pre-course score 2.47 (SD = 0.94), mean post-course score 3.03 (SD = 1.26), p < 0.01) and more knowledgeable (mean pre-course score 2.88 (SD = 0.96), mean post-course score 4.04 (SD = 0.56), p < 0.01). These findings were similar to previous courses.

**Conclusions**: Feedback was consistently positive in all domains over all courses run since 2018. The program has improved novice knowledge and confidence in the basics of critical care, and the course is now multidisciplinary.**Reference**

1. Baker C et al. Intensive Care Med Exp 7(Supp 3):55, 2019

**Table:** **Table 1** **(abstract P199)** Results from 2020/2021 feedback: mean pre- and post-course responses, all p < 0.02

Confidence with:	Pre-course (mean (SD))	Post-course (mean (SD))
Non-invasive ventilation	2.94 (0.83)	3.70 (0.97)
Mechanical ventilation	2.65 (1.17)	3.48 (0.95)
Cardiac output monitoring	2.82 (0.95)	3.78 (1.00)
Inotropes	2.76 (1.15)	3.74 (0.96)
Renal replacement therapy	2.59 (1.28)	3.52 (1.16)
Sedation and delirium	2.82 (1.07)	3.91 (0.95)
Airway management	2.82 (1.07)	3.96 (0.98)

## P200

### A review of handovers of new patients on an intensive care unit

#### E. J. Jones^1^, D. H. Hepburn^2^

##### ^1^Cardiff University, Cardiff, UK; ^2^Intensive Care Unit, Royal Gwent Hospital, Newport, UK

*Critical Care* 2021, **25**(**Suppl 1**): P200

**Introduction**: Every new admission to the intensive care unit (ICU) prompts a handover from the referring department to the ICU staff. This step in the patient pathway provides an opportunity for information to be lost and for patient care to be compromised. Mortality rates in intensive care have fallen over the last twenty years, however, 20% of patients admitted to an ICU will die during their admission [1]. Communication errors contribute to approximately two-thirds of notable clinical incidents; over half of these are related to a handover [2]. NICE have concluded that structured handovers can result in reduced mortality, reduced length of hospital stay and improvements in senior clinical staff and nurse satisfaction [3].

**Methods**: A checklist was created with doctors and nurses to review the information shared and to score each handover. This was relevant for handovers between all staff members. Information was gathered prospectively by directly observing 17 handovers on the ICU.

**Results**: There is a notable discrepancy in the quality of handovers of new patients (Fig. 1). This is true of handovers between doctors, nurses and a combination of the two. It is also true of all staff grades. Whilst a doctor may have reviewed the patient prior to their arrival, 41% (n = 7) of patients were not handed over to a doctor. The most commonly missed pieces of information were the patient’s weight (96%, n = 16), their height (100%, n = 17), whether they had previously been admitted to an ICU (78%, n = 15) and whether they had any allergies (71%, n = 12).

**Conclusions**: The handover of new patients to the ICU is often unstructured and information is missed. This is true for all staff members and grades, and for handovers from all hospital departments. Standardising handovers could improve their efficiency and effectiveness.

**References**:

1. ICS. Guidelines for the Provision of Intensive Care Services, Version 2. ICS; 2018.
2. Starmer AJS et al. Pediatrics 129:201–4, 2012
3. National Institute for Health and Care Excellence (UK). Chapter 32, Structured Patient Handovers, 2018.

**Figure:**
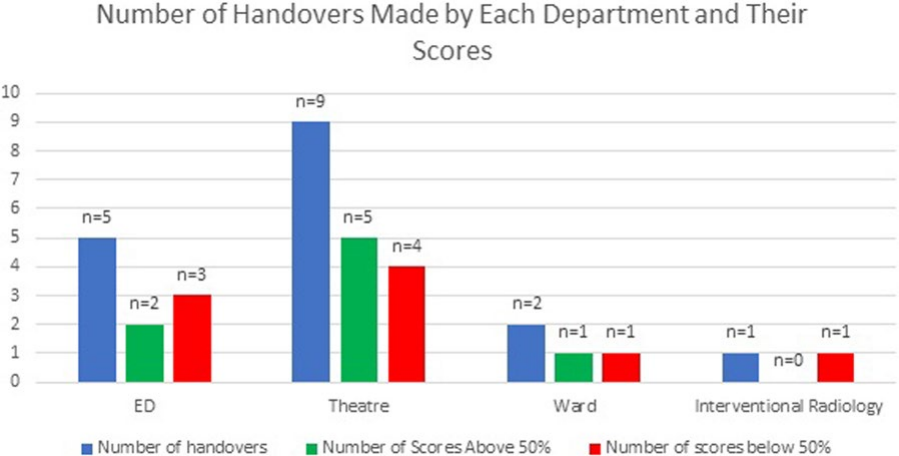
**Fig. 1** **(abstract P200)** A graph showing the score various departments achieved with their handovers

## P201

### Predictive value of the APACHE II and IV score for ICU mortality in critically ill patients with COVID-19: a retrospective cohort study

#### P. L. Piccart, S. B. Stessel, V. J. Vandenbrande, L. Paclé, C. I. Callebaut, D. J. Dubois

##### Intensieve Zorgen, Jessa Ziekenhuis, Hasselt, Belgium

*Critical Care* 2021, **25**(**Suppl 1**): P201

**Introduction**: Severity scores play a crucial role in comparing predicted versus observed outcomes, allowing evaluation of treatment and benchmarking of ICU performance. Acute physiology and chronic health evaluation (APACHE) scoring systems are designed for ICU mortality prediction. APACHE scoring systems are not validated in critically ill COVID-19 patients. This study aims to assess and compare the predictive value of APACHE II and IV for ICU mortality in critically ill COVID-19 patients.

**Methods**: All adult patients with a laboratory-confirmed diagnosis of COVID-19 pneumonia admitted to the ICU of Jessa Hospital, Hasselt, Belgium between March 13, 2020, and October 17, 2020, were included in this retrospective, cohort study. APACHE II and APACHE IV scores were calculated within 24 h after admission. The performances of the APACHE scoring systems were evaluated by means of logistic regression models with ICU mortality as the dependent variable and one of the scoring systems as an independent variable. Discrimination for mortality was assessed by receiver operating characteristic curves. tenfold cross-validation was used to obtain more conservative estimates of the discriminative abilities of the prediction tools. The Hosmer–Lemeshow goodness-of-fit (HL) test was used to assess calibration.

**Results**: In total, 116 COVID-19 patients were admitted to the ICU between March 13, 2020, and October 17, 2020. 13 COVID-19 patients were excluded for various reasons, leaving 103 patients in the statistical analysis of the overall population. Table 1 shows the results of the cross-validated discriminative abilities and the HL test.

**Conclusions**: APACHE IV on admission provided the best discrimination and calibration for ICU mortality in critically ill COVID-19 patients compared to APACHE II. Nevertheless, discriminative abilities of APACHE IV scores were only moderate (AUC 0.67) after correction for optimism.

**Table:** **Table 1** **(abstract P201)** Results of univariate logistic regression model

Severity Score	OR (95% CI)	AUC	AUC (after crossvalidation)	HL statistics
APACHE II	1.10 (1.01, 1.21)	0.67	0.63	10.98 (0.1394)
APACHE IV	1.05 (1.02, 1.08)	0.70	0.67	2.54 (0.9234)
Comparison of AUC ROC				
APACHE IV versus APACHE II				

## P202

### Development of the HIV In-hospital Mortality Prediction (HIV-IMP) risk score

#### A. Laher^1^, F. Paruk^2^, W. Venter^3^, O. Ayeni^4^, F. Motara^1^, M. Moolla^1^, G. Richards^5^

##### ^1^Emergency Medicine, University of the Witwatersrand, Parktown, Johannesburg, South Africa; ^2^Critical Care, University of Pretoria, Pretoria, South Africa; ^3^Infectious Diseases, University of the Witwatersrand, Parktown, Johannesburg, South Africa; ^4^Wits Developmental Pathways for Health Research Unit,University of the Witwatersrand, Parktown, Johannesburg, South Africa; ^5^Critical Care, University of the Witwatersrand, Parktown, Johannesburg, South Africa

*Critical Care* 2021, **25**(**Suppl 1**): P202

**Introduction**: With 690 000 HIV related deaths in 2019, HIV is a major cause of global mortality. Despite over 30 years into the HIV epidemic, there are currently no clinical scoring tools that can predict mortality in HIV-positive patients requiring hospital admission. Therefore, the aim of this study was to develop and internally validate such a score [1].

**Methods**: Consecutive HIV-positive patients presenting to the Charlotte Maxeke Johannesburg Academic Hospital Adult Emergency Department between 07 July 2017 and 18 October 2018 were prospectively enrolled. Multivariate logistic regression was used to determine parameters for inclusion in the final risk score. Discrimination and calibration were assessed by means of the area under the receiver operating curve (AUROC) and the Hosmer–Lemeshow goodness-of-fit test respectively. Internal validation was conducted using the regular bootstrap technique.

**Results**: The overall in-hospital mortality rate was 13.6% (n = 166). Eight predictors were included in the final risk score: ART non-adherence or not yet on ART, Glasgow coma scale < 15, respiratory rate > 20 breaths per minute, oxygen saturation < 90%, white cell count < 4 × 109/l, creatinine > 120 μmol/l, lactate > 2 mmol/l and albumin < 35 g/l. After internal validation, the risk score maintained good discrimination (AUROC 0.83, 95% confidence interval (CI) 0.78 – 0.88) and calibration (Hosmer–Lemeshow χ^2^ 2.26, p = 0.895).

**Conclusions**: The HIV In-hospital Mortality Prediction (HIV-IMP) risk score has overall good discrimination and calibration is relatively easy to use. Further studies should be aimed at externally validating the score in varying clinical settings.

**Reference**

1. Laher A et al. HIV Med 22:557–566, 2021

**Figure:**
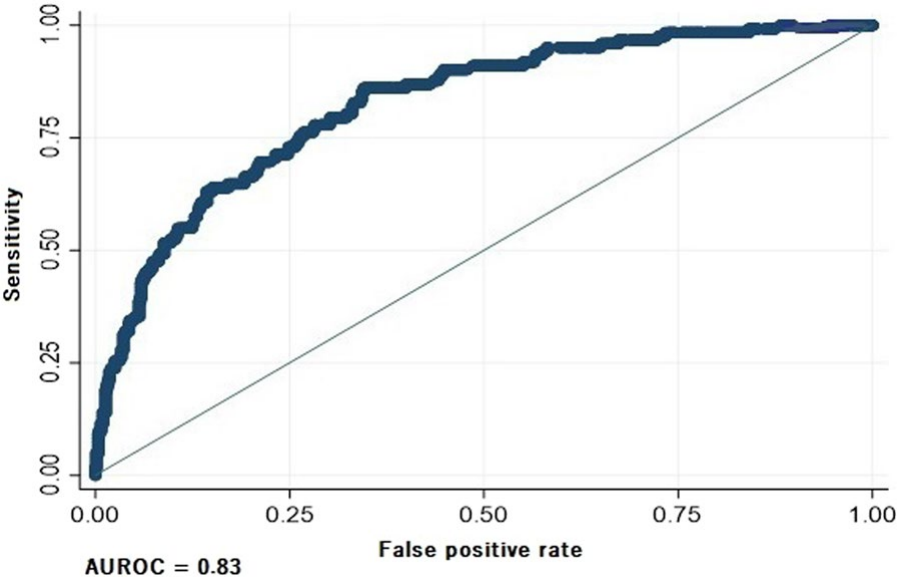
**Fig. 1** **(abstract P202)** Receiver operating characteristic (ROC) curve of the HIV-IMP risk score

## P203

### Recovery from COVID-19 critical illness: a secondary analysis comparing recovery from COVID-19 and general critical illness

#### E. Pauley^1^, T. M. Drake^2^, D. M. Griffith^3^, N. Lone^4^, E. Harrison^5^, J. K. Bailie^6^, J. T. Scott^7^, T. S. Walsh^3^, M. G. Semple^8^, A. B. Docherty^5^

##### ^1^University of Edinburgh Medical School, Edinburgh, UK; ^2^Centre for Medical Informatics, and Intensive Care Unit, Royal Infirmary Edinburgh, University of Edinburgh, Edinburgh, UK; ^3^Anaesthesia, Critical Care & Pain Medicine, University of Edinburgh, Edinburgh, UK; ^4^Usher Institute, University of Edinburgh, Edinburgh, UK; ^5^Centre for Medical Informatics, University of Edinburgh, Edinburgh, UK; ^6^Roslin Institute, University of Edinburgh, Edinburgh, UK; ^7^MRC-University of Glasgow Centre for Virus Research, University of Glasgow, Glasgow, UK; ^8^Institute of Infection, Veterinary and Ecological Sciences, University of Liverpool, Liverpool, UK

*Critical Care* 2021, **25**(**Suppl 1**): P203

**Introduction**: We aimed to compare the prevalence and severity of fatigue in survivors of COVID-19 versus non-COVID-19 critical illness, and to explore potential associations between baseline characteristics and worse recovery.

**Methods**: We conducted a secondary analysis of two prospectively collected datasets. The population included was 92 patients who received invasive mechanical ventilation (IMV) with COVID-19, and 240 patients who received IMV with non-COVID-19 illness before the pandemic. Follow-up data were collected using self-reported questionnaires. The main outcome measures were self-reported fatigue severity and the prevalence of significant fatigue at 3–12-months post-hospital discharge.

**Results**: COVID-19 IMV-patients were significantly younger with less prior comorbidity, and more males, than pre-pandemic IMV-patients. At 3-months the prevalence (87.5% [7/8] vs. 82.4% [155/188]) and severity (median 5.5/10 vs. 5.0/10) of fatigue was similar between COVID-19 and pre-pandemic populations respectively. At 6-months the prevalence (59.4% [19/32] vs. 87.3% [145/166]) and severity (median 2.0/10 vs. 5.7/10) of fatigue was less in the COVID-19 cohort. In the COVID-19 population, women under 50 experienced more severe fatigue, breathlessness, and worse overall health state compared to other COVID-19 IMV-patients (adjusted mean difference 2.58, 95%CI: -0.19 to 5.35). There were no significant sex differences in long-term outcomes in the pre-pandemic population. In the total sample included, having COVID-19 disease was significantly associated with not reporting fatigue reaching 7/10 severity (adjusted OR 0.35, 95%CI: 0.15 to 0.76).

**Conclusions**: This study has shown that survivors of both COVID-19 and non-COVID-19 critical illness experience high levels of persistent fatigue. Fatigue may be less severe after COVID-19 than after other critical illness.

## P204

### Systematic review and comparison of ICU datasets – a decision guide for clinicians and data scientists

#### C. M. Sauer^1^, T. A. Dam^1^, L. A. Celi^2^, A. R. Girbes^1^, P. W. Elbers^1^

##### ^1^Department of Intensive Care Medicine, AmsterdamUMC, Amsterdam, Netherlands; ^2^Laboratory for Computational Physiology, Institute for Medical Engineering and Science, Massachusetts Institute of Technology, Cambridge, USA

*Critical Care* 2021, **25**(**Suppl 1**): P204

**Introduction**: The publication of large, rich, single patient level data sets has significantly propelled clinical research possibilities in the intensive care unit (ICU) and beyond. After publication of MIMIC-III, additional datasets have previously been released [1–4], thus warranting a comparison of their data completeness and richness to allow scientists to choose the most appropriate dataset(s) for their clinical problem.

**Methods**: A systematic search of published and pre-print articles was performed to identify all publicly available, adult, critical care, patient level databases. Subsequently, databases were compared using a priori defined categories, such as demographics, patient characteristics and data richness.

**Results**: A total of 4 ICU databases were identified (MIMIC-IV, eICU-CRD, AmsterdamUMCdb, HiRID). Number of unique patient admissions varied significantly between datasets, with eICU-CRD being the largest (> 130 K) and AmsterdamUMCdb the smallest (> 23 k). ICU mortality and intensity of treatment also varied, with eICU-CRD 28-day mortality rates and frequency of ventilation being lowest among the datasets. Frequency of laboratory values tended to be highest in MIMIC-IV, while frequency of vital signs was highest in AmsterdamUMCdb.

**Conclusions**: Several high-quality ICU databases are currently available. The research question, and thus required sample size, presence of covariates and frequency of measurements, should inform which database to use.

**References**:

1. Johnson AEW et al. Sci Data 3:1–9, 2016
2. Pollard TJ et al. Sci Data 5:1–13, 2018
3. Thoral PJ et al. Crit Care Med 49:e563-e577, 2021
4. Faltys M et al. HiRID, a high time-resolution ICU dataset (version 1.1.1). PhysioNet, 2021

## P205

### Sex differences in treatment intensity of adult intensive care patients: a systematic review and meta-analysis

#### L. Modra^1^, A. Higgins^2^, V. Abeygunawardana^3^, R. Vithanage^4^, M. Bailey^2^, R. Bellomo^1^

##### ^1^Department of Intensive Care, Austin Health, Heidelberg, Australia; ^2^Monash University, Melbourne, Australia; ^3^Austin Health, Heidelberg, Australia; ^4^Bendigo Health, Bendigo, Australia

*Critical Care* 2021, **25**(**Suppl 1**): P205

**Introduction**: We undertook a systematic review to synthesise and evaluate the available literature on sex differences in the treatment intensity of adult intensive care unit (ICU) patients.

**Methods**: Data sources: MEDLINE, EMBASE. Study selection: Two reviewers independently screened studies to identify observational studies of adult ICU patients that explicitly examined the association between sex and treatment intensity, specifically mechanical ventilation, renal replacement therapy, and length of stay. Data extraction: We extracted data independently and in duplicate: mean age and illness severity of women and men, length of stay in ICU and hospital, use of and duration of mechanical ventilation and use of renal replacement therapy. We assessed risk of bias using the Newcastle–Ottawa scale and used a Dersimonian-Laird random effects model to calculate pooled odds ratios and mean differences between women and men.

**Results**: We identified 21 studies with a total of 545,538 participants (42.7% women). The study populations ranged from 246 to 261,255 participants (median 4420). Most studies were at high risk of bias in at least one domain, for example not adjusting treatment intensity outcomes for illness severity or other potential confounders. Women were less likely than men to receive invasive mechanical ventilation (MV pooled OR = 0.83, 95% CI 0.77–0.89, I^2^ = 90%) (Fig. 1) or renal replacement therapy (RRT pooled 0.79, 95% CI 0.70–0.90, I^2^ = 76%) and had shorter mean length of stay in ICU than men (mean difference -0.24 days, 95% CI -0.37 to -0.12, I^2^ = 90%). These findings persisted in a pre-specified sensitivity analysis excluding studies at high risk of bias. Women and men had similar mean duration of mechanical ventilation and hospital length of stay.

**Conclusions**: Compared to men, women were less likely to receive mechanical ventilation or renal replacement therapy and had shorter admissions to the ICU. There is substantial heterogeneity and risk of bias in the existing literature, therefore further research is warranted.

**Figure:**
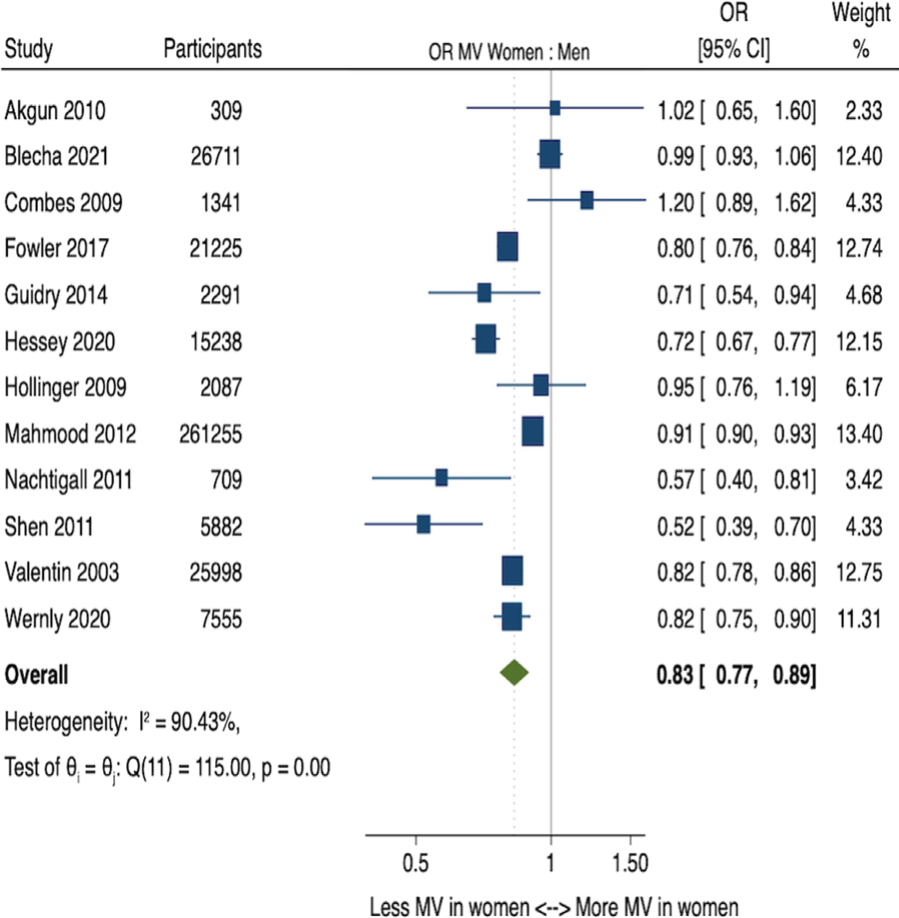
**Fig. 1** **(abstract P205)** Use of mechanical ventilation in women compared to men in the ICU

## P206

### Clinical frailty scale (CFS) indicated frailty is associated with increased in-hospital and 30-day mortality in COVID-19 patients: a systematic review and meta-analysis

#### M. Rottler^1^, K. Ocskay^2^, A. Görbe^2^, M. Virág^1^, P. Hegyi^2^, T. Molnár^3^, B. Erõss^2^, T. Leiner^4^, Z. Molnár^2^

##### ^1^Department of Anaesthesiology and Intensive Therapy, Fejér County Szent György University Teaching Hospital, Székesfehérvár, Hungary; ^2^Institute for Translational Medicine, University of Pécs, Pécs, Hungary; ^3^Department of Anaesthesiology and Intensive Therapy, University of Pécs, Pécs, Hungary; ^4^Anaesthetic Department, Hinchingbrooke Hospital, North West Anglia NHS Foundation Trust, Huntingdon, UK

*Critical Care* 2021, **25**(**Suppl 1**): P206

**Introduction**: The concept of frailty provides an age-independent, easy-to-use tool for risk stratification. We aimed to summarize the evidence regarding the use of frailty tools in COVID-19, assessing the risk of frail patients for in-hospital and 30-day mortality, intensive care unit (ICU) admission, and length of hospitalization (LOH).

**Methods**: The protocol was prospectively registered via PROSPERO (CRD42021241544). We conducted a systematic search up to 03.02.2021 with terms related to COVID-19 and frail* in MEDLINE (via PubMed), EMBASE, Scopus, CENTRAL, and Web of Science. Studies reporting on frailty in COVID-19 patients were eligible. We compared in-hospital and 30-day mortality, LOH and ICU admission in frail and non-frail COVID-19 patients. Search, selection, data extraction and risk of bias (RoB) assessmentwere conducted in duplicate by two independent authors. The QUIPS tool was used for the RoB assessment. Odds ratios (OR) and weighted mean differences (WMD) were calculated with 95% confidence intervals (CI) using a random effect model. Heterogeneity was assessed using the I2and χ^2^ tests.

**Results**: From 1693 records, 27 were included in the qualitative and 21 in the quantitative synthesis. Clinical Frailty Scale (CFS) was used in 24 studies. We found that frail patients (CFS 5–9) compared to non-frail patients (CFS 1–4) have a higher risk for both in-hospital (OR: 2.77; CI: 1.86–4.15) and 30-day mortality (OR: 1.47; CI: 1.05–2.06). Frail patients were less likely to be admitted to ICU: CFS 4–9 (OR: 0.13, CI: 0.09–0.17); CFS 5–9 (OR 0.05, CI: 0.01–0.16); (Table 1). Quantitative synthesis for LOH was not feasible. Most results showed considerable heterogeneity. Most studies carried a high risk of bias.

**Conclusions**: As determined by CFS, frailty is strongly associated with in-hospital and 30-day mortality; hence, investigating its use in deciding on ICU admission further in COVID-19 is warranted.

**Table:** **Table 1** **(abstract P206)** Summary of findings

Outcomes	CFS categorization	No. of patients (Studies)	Relative effect: OR (95% CI)	Risk of bias (QUIPS)
In-hospital mortality	CFS 1–4 vs 5–9	4023 (11)	2.77 (1.86, 4.15)	10 High risk, 3 Low risk
	CFS 1–5 vs 6–9	4537 (8)	3.14 (2.09, 3.53)	
30-day mortality	CFS 1–4 vs 5–9	1445 (4)	1.47 (1.05, 2.06)	4 High risk, 1 Low risk
	CFS 1–5 vs 6–9	1058 (3)	1.62 (0.96, 2.74)	
ICU admission	CFS 1–3 vs 4–9	2636 (4)	0.13 (0.09, 0,17)	6 High risk, 0 Low risk
	CFS 1–4 vs 5–9	1774 (4)	0.05 (0.01, 0.16)	

## P207

### Predicting emergency hospital admissions in intensive care survivors

#### C. Beattie^1^, P. Henderson^2^, M. Shaw^2^, T. Quasim^2^

##### ^1^School of Medicine, University of Glasgow, Glasgow, UK; ^2^University of Glasgow, Glasgow, UK

*Critical Care* 2021, **25**(**Suppl 1**): P207

**Introduction**: Relative to the general hospital population, ICU survivors experience high levels of morbidity following hospital discharge, leading to high rates of unplanned hospital readmission. If we can predict which patients are at highest risk of readmission, targeted outpatient interventions can be made to prevent use of costly emergency healthcare resources. Existing models to predict readmissions, designed for the general hospital population, have poor predictive performance in the ICU survivor cohort. We set out to create a parsimonious, ICU-specific model to predict emergency readmission in ICU survivors.

**Methods**: Data were collected retrospectively on patients discharged alive from hospital following ICU admission from a single Scottish ICU between April and October 2018. Patients were split into derivation and validation cohorts using an 80:20 random split. A logistic regression model was developed using the derivation cohort; performance of the model was determined using the validation cohort.

**Results**: A total of 121 of 402 patients (30.1%) experienced unplanned readmission within 90 days of hospital discharge. The model consists of eight predictors which predict 90-day emergency hospital admissions with a discriminatory c-index of 0.75 (0.70–0.80). Significant predictors in the multivariable model were: number of emergency admissions in year prior to ICU admission (OR = 1.51, 95% CI = 1.25, 1.83) and number of elective admissions in year prior to ICU admission (OR = 1.24, 95% CI = 1.09, 1.41). Table 1 illustrates the performance of the model as a screening tool.

**Conclusions**: Emergency hospital readmissions are common in this ICU survivor cohort. This simplistic model can be used as a screening tool at hospital discharge to identify patients at high risk of emergency hospital readmission. This allows risk stratification of need for outpatient support. Further consideration should be given to exploring modifiable risk factors for readmission to improve the cost-effectiveness and clinical utility of post-ICU outpatient care.

**Table:** **Table 1** **(abstract P207)** Performance of risk prediction model as a screening tool to identify patients at risk of emergency hospital readmission

Threshold of predicted risk for screening positive	Number (%) screening positive	Sensitivity (%)	Specificity (%)
0.2	70.0	95.5	39.7
0.25	53.8	77.3	55.2
0.3	42.5	68.2	67.2
0.35	28.8	63.6	84.5
0.4	22.5	54.5	89.7

## P208

### Interventions and outcomes of the very old admitted to the intensive care unit

#### A. Spataru, M. Angelova-Chee, V. Metaxa

##### Critical Care, King’s College Hospital, London, UK

*Critical Care* 2021, **25**(**Suppl 1**): P208

**Introduction**: Old adults are the fastest-growing segment of the population in many developed countries [1]. Data paucity on the post-ICU outcomes in this population is perpetuated by the reluctance of physicians to admit very old patients (VOP) to the ICU [2, 3]. The aim of this study was to assess the characteristics and the short and longer-term outcomes of critically ill VOP.

**Methods**: We included all patients aged 80 y/o and over admitted to the ICU of a large tertiary hospital in 2018. We collected patient demographics (including frailty scores) along with data on admission diagnoses, treatment escalation decisions, and mortality. Frailty scores were assigned based on admission functional status, using a 9-item clinical frailty scale (CSF) [4].

**Results**: A total of 191 patients with a median age of 84 (IQR 82–87 y/o) were included. More than half (51%) were frail and almost a quarter (23%) had a do-not-attempt-cardiopulmonary resuscitation (DNACPR) order in place before ICU admission. Most VOP were admitted emergently (93.7%), with a non-surgical diagnosis (78.5%). Details on the interventions undertaken in both groups are shown in Table 1. In our cohort, the ICU mortality was 30.4% and the 1-year fatality rate 60.2%. Higher frailty was associated with lower ICU death (16.3% vs 45.2%, p < 0.001), but similar hospital and long-term mortality. Factors associated with non-survival at 1 year were higher SOFA scores (OR 1.2, 95%CI 1.001–1.4), DNACPR status on admission (OR 2.7, 95%CI 1.1–6.5), and non-surgical pathology (OR 3.5, 95%CI 1.5–8.8).

**Conclusions**: Most of the VOP survived their ICU stay and almost 40% of them were alive at 1 year. Our results do not support ICU admission bias against this population. However, as we did not examine long-term sequelae, the burden of critical illness on functional outcomes could not be assessed.

**References**

1. Waite LJ. Popul Dev Rev 30:3–16, 2004.
2. Guidet B et al. Ann Intensive Care 8:114, 2018
3. Boumendil A et al. PLoS One 7:e34387, 2012
4. Church S. BMC Geriatr 20:393, 2020

**Table:** **Table 1** **(abstract P208)** Interventions undertaken

	**Total (n = 191)**	**ICU survivors (n = 133)**	**ICU non-survivors (n = 58)**	**p value**
Interventions in the ICU				
Invasive ventilation, n (%)*	101 (52.9%)	50 (37.6%)	51 (87.9%)	< 0.001
Non-invasive ventilation, n (%)**	22 (11.5%)	22 (16.5%)	0 (0.0%)	< 0.001
Catecholamines, n (%)	110 (57.6%)	61 (45.9%)	49 (84.5%)	< 0.001
Renal replacement therapy, n (%)	43 (22.5%)	19 (14.3%)	24 (41.4%)	< 0.001
Transfusion, n (%)	41 (21.5%)	27 (20.3%)	14 (24.1%)	< 0.001
ICU LOS (median), days (IQR)	3 (2.0- 6.5)	3 (2.0- 7.0)	2 (1.0- 6.0)	0.045

*Includes patients on NIV before invasive ventilation ** Includes patients on NIV only (never escalated to invasive ventilation)

## P209

### Impact of the box-trainer in applying ergonomic principles for minimally invasive surgery

#### W. Sellami^1^, S. Landolsi^2^, I. Ben Mrad^1^, M. Zakraoui^1^, S. Bougheriou^1^, I. Labbene^1^, M. Ferjani^1^

##### ^1^Department of Critical Care Medicine and Anesthesiology, Military Hospital of Tunis, Tunis, Tunisia; ^2^Department of Surgery, Mahmoud El Matri Hospital, Ariana, Tunisia

*Critical Care* 2021, **25**(**Suppl 1**): P209

**Introduction**: Minimally invasive surgery constituted the source for professional illnesses among surgeons due to ergonomic problems.The aim of this study was to investigate the impact of the box-trainer in applying ergonomic guidelines for minimally invasive surgery.

**Methods**: Our study was a prospective, transversal, and evaluative study realized at the department of surgery, Mahmoud El Matri hospital, during the period between January 15 and February 15, 2020. Volunteered-surgical resident trainees were recruited for this study. Low-cost box-trainer was used. Evaluative score was calculated after peg transfer task in optimal ergonomic settings then in non-optimal ergonomic settings. The principal judgment criterion was the difference between the scores.

**Results**: First- and second-year participants accounted for 57% of all participants. Ten participants (33%) had no experience with minimally invasive surgery simulation. Twenty-two participants (73%) weren’t aware about ergonomic principles. None of the participants had experienced ergonomic-dedicated simulations in minimally invasive surgery. The mean score obtained after peg transfer in optimal ergonomic settings was 107.0 (extremes: 19 and 220) versus 54.8 (extremes: 0 and 191) in non-optimal ergonomic settings. The difference was 39.6 in median (extremes: 7.3 and 70.1) with a statistically significance. Multi variant analysis retained only the resident year as an independent factor associated to the accomplished scores after comparison of the scores reached by first- and second-year residents versus those of third-, fourth-, and fifth-year residents.

**Conclusions**: The impact of the box-trainer was the awareness rise about the importance of ergonomics in minimally invasive surgery, the application of these concepts, and the work environmental enhancement allowing better security for both sanitary staff and patients.

## P210

### Models of care in geriatric intensive care (MAGIC): a scoping review on the models of care for critically ill older adults

#### T. Wissanji^1^, M. F. Forget^2^, D. Beaudin^3^, H. T. Wang^4^

##### ^1^Division of Critical Care, Department of Medicine, Maisonneuve-Rosemont Hospital, Montréal, Canada; ^2^Division of Geriatric Medicine, Centre Hospitalier de l´Université de Montréal, Montréal, Canada; ^3^Division of Geriatric Medicine, Maisonneuve-Rosemont Hospital, Montréal, Canada; ^4^Division of Critical Care, Department of Medicine, Centre hospitalier de l´Université de Montréal, Montréal, Canada

*Critical Care* 2021, **25**(**Suppl 1**): P210

**Introduction**: The geriatric population admitted to critical care units is growing with the increasing age of the North American population. Geriatric ICU patients present unique challenges in terms of management due to their physiology and comorbidities. We aim to review models of care of geriatric population in critical care settings.

**Methods**: We conducted a scoping review on critically ill older adult hospitalized in critical care settings. Medline (PubMed), Embase (OvidSP), CINAHL (Ebsco) and Web of Science (Clarivate) were searched from inception to 28 August 2019, with a subsequent review of Pubmed made in May 2020. Nineteen articles were selected for the review.

**Results**: Four models of care could be extracted from this review: dedicated geriatric beds, geriatric assessment by geriatrician, geriatric assessment without geriatrician, and other models of care including checklists, bundles of care, and incremental education strategies. Because of an absence of comparative studies, the optimal model of care could not be identified through our review.

**Conclusions**: Four models of care with varying applicability and resource requirements were identified. Additional studies will be necessary to determine the optimal model of care.

## P211

### Modeling of COVID-19 pandemic to estimate the demand of mechanical ventilators

#### Y. Yuan, C. Tian

##### Philips Research, Philips, Shanghai, China

*Critical Care* 2021, **25**(**Suppl 1**): P211

**Introduction**: The emergence of several variants is associated with COVID-19 rebound in many countries and regions with higher transmission advantage, and additional demand on medical resources including mechanical ventilators (MV). It can be challenging to estimate the demand by using infection rate of population, given the epidemiological response varies over time due to different factors such as pathogen variant, non-pharmaceutical interventions. Therefore, we modeled the outbreak with time-varying epidemiological response to estimate the demand of MV in a dynamic way.

**Methods**: Confirmed cases and fatalities in six countries (US, Italy, UK, Russia, Saudi Arabia, Brazil), were collected from COVID-19 data repository by Johns Hopkins University. We performed a Long-short term memory (LSTM) model to predict confirmed cases and fatalities at 8th day by using data for past 7 days. Data from Jan 22 to May 14, 2020 were split into training (80%) and validation (20%) dataset. Internal testing dataset was from May 16 to May 24, 2020, while Taiwan data from May9 to Jun16, 2021 were used as external testing. The root mean squared logarithmic error (RMSLE) was used to evaluate the model performance. Then daily additional demand of MV for COVID-19 can be estimated by using below equation: Daily MV demand = (daily confirmed cases × hospitalization rate × ventilation rate).

**Results**: The average RMSLE for confirmed cases and fatalities of six modeling countries were 0.08 and 0.13 respectively. For Taiwan confirmed cases was much higher than modeling countries (0.26), whereas slightly increased in fatalities (0.18). The figure also showed that our model captured the systematic variation in number of new cases per day in Taiwan and estimate additional 181 MV were needed in peak day.

**Conclusions**: Our results can be of help to indicate the time-varying trend of COVID-19 and assist management team to estimate dynamic demand of MV for different regions. The early warning to government can leave more time for public decision-making.

**Figure:**
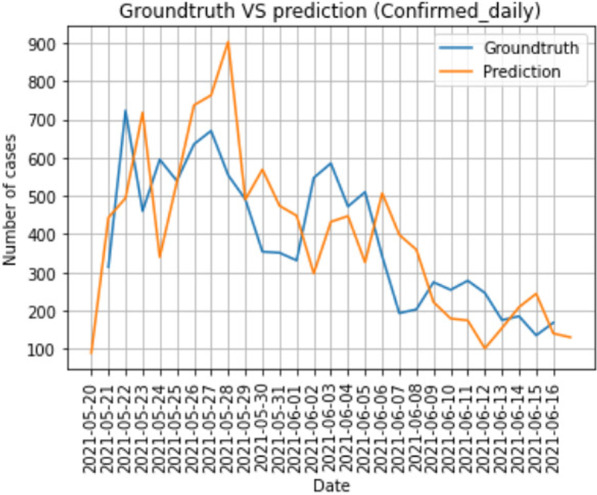
**Fig. 1** **(abstract P211)** Time series of confirmed cases per day in Taiwan by both reporting and modeling

## P212

### When the second wave comes greater than the first one

#### K. Donadello^1^, L. Gottin^2^, A. Russo^3^, P. Zanatta^4^, V. Schweiger^2^, S. Boschetti^2^, A. Olivieri^2^, M. Nizzero^2^, E. Polati^2^

##### ^1^Anaesthesia and Intensive Care B, Department of Surgery, Dentistry, Gynaecology and Paediatrics, University of Verona, School of Medicine, Verona, Italy; ^2^University of Verona, School of Medicine, Verona, Italy; ^3^Cardio-Thoracic Anesthesia and Intensive Care, AOUI_University Hospital Integrated Trust of Verona, Verona, Italy; ^4^Anesthesia and Intensive Care A, AOUI_University Hospital Integrated Trust of Verona, Verona, Italy

*Critical Care* 2021, **25**(**Suppl 1**): P212

**Introduction**: Since Feb 2020 Italy has been facing COVID-19 pandemic. After the tough but efficient first wave struggle, the Veneto Region in Italy faced a more difficult second surge. We aimed to describe the COVID-19 critically ill pts admitted to our hospital (AOUI of Verona) during the second wave.

**Methods**: Prospective observational study of all COVID-19-confirmed critically ill pts, treated at our ICUs between 1 Oct 2020 to 2 Feb, 2021. Date of final follow-up was May 1, 2021. Demographic and daily clinical data were collected, including data on organ failure, management and outcome.

**Results**: According to our treatment protocol, all pts received dexamethasone 6 mg/day,therapeutic anticoagulation with LMWH and were supplemented with Vit C and D. 212 pts were included in the analysis, the median age was 65 ± 11 years and 161 (76%) were male.164 (77%) had cardiovascular disorders,43 (20%) had diabetes, overall BMI was 28.7(26–31); 105(49.5%) pts received pre-hospital antibiotic therapy. 112 (53%) pts were admitted from an intermediate care medical ward while 18 (8.5%) were transferred from another hospital ICU. APACHE II and SOFA scores were 17 ± 8 and 9.4 ± 3.1; 171 (80.7%) pts had only respiratory failure, while 17.3% of them were on vasopressors upon ICU admission. CRP was 105 ± 43 mg/l, PCT was 1.6 ± 0.2 ng/ml; total CPK, LDH and D-dimer were 290 ± 20 U/l, 376 ± 20 U/l and 1750 ± 555, respectively; lactate level was 1.3 (1–1.7) mmol/l. P/F upon ICU admission was 121 (99–170), 177 (83.5%) pts needed tracheal intubation and 15 (7.1%) needed ECMO. 116 (54.7%) pts resulted MDR positive during their hospital stay. 28 day mortality was 21.2% and this was related to age and BMI (p < 0.05); global ICU mortality was 30.2 (p < 0.05 for age and sex) and hospital mortality was 34.4% (p < 0.05 for age, sex and BMI). ICU and hospital LOS were 18 (6–25) 31 (18–45) days; this latter was significantly related to BMI. Patients' outcome was significantly related to peak lymphocyte, lactate, fibrinogen and D-Dimer.

**Conclusions**: Our second hit was tougher with higher morbidity and mortality.

## P213

### How to protect the hospital from contamination: predictors of COVID-19 infection among emergency department consultants

#### N. E. Nouira^1^, W. Demni^2^, E. M. Ben Othmane^2^, W. Bahria^2^, D. Hamdi^2^, H. Snene^3^, N. Kaffel^4^, M. S. Mebazaa^5^

##### ^1^Emergency Department, Mongi Slim Academic Hospital, Tunis, Tunisia; ^2^Emergency Medicine, Mongi Slim Academic Hospital, Tunis, Tunisia; ^3^Pneumology Department, Mongi Slim Academic Hospital, Tunis, Tunisia; ^4^COVID-19 Committee, Mongi Slim Academic Hospital, Tunis, Tunisia; ^5^COVID-19 Committee / Anesthesia Resuscitation Department, Mongi Slim Academic Hospital, Tunis, Tunisia

*Critical Care* 2021, **25**(**Suppl 1**): P213

**Introduction**: As the COVID-19 pandemic increases, Tunisia, like other countries, is committed to reorganizing health structures, mainly emergency departments, which not only continue to provide care for non-COVID-19 patients but also manage an influx of suspected COVID-19 patients.The objective of our study was to identify predictors for COVID-19 infection and to avoid contamination of the hospital.

**Methods**: This was a prospective, descriptive study, extending over two months (from 03/27/2020 to 05/26/2020). We included all patients consulting the emergency department, regardless of the reason. The predictive factors of a COVID-19 infection, confirmed by PCR or by a typical appearance on the chest CT scan were sought.

**Results**: We included 5241 patients. The sex ratio was 0.97. The mean age was 44.6 ± 18 years. Thirty-five percent of patients were smokers. The most common medical history in our patients was hypertension (23.9%), diabetes (19.5%), asthma (7.8%), and coronary artery disease (7.7%). The reasons for consultation were dominated by cough, dyspnea, nausea or vomiting, and fever, in 9.5, 8.5, 8.8, and 6.9% of cases respectively. The concept of exposure was observed in 3.1% of patients. The concept of contact with a suspected case was observed in 1.3% of patients, while contact with health personnel was observed in 6.3% of patients. Suspicion of covid 19 infections was found in 2.3% of patients. Eighty-nine percent of patients were managed in the emergency department. Independent predictors of COVID-19 infection in multivariate analysis were: fever (adjusted OR = 21.19, p < 0.01), breathing difficulties (adjusted OR = 16.49, p < 0.01), neoplasia (adjusted OR = 15.93, p < 0.025), and contact with health care personnel (adjusted OR = 5.78, p < 0.019).

**Conclusions**: Identification of predictive factors for COVID-19 infection allows detection of suspect patients up on arrival at pre-triage and triage areas, allowing isolation while ensuring continuity of care for non-COVID-19patients.

## P214

### Validation of the COVID-19 decision support tool in the intensive care unit of a large non-university, teaching hospital; a retrospective analysis

#### J. G. Grietens, X. W. Willaert, D. M. Mesotten, T. F. Fivez

##### Anesthesiology and Intensive Care, Ziekenhuis Oost-Limburg, Genk, Belgium

*Critical Care* 2021, **25**(**Suppl 1**): P214

**Introduction**: In early spring, Belgium was struck hard by the COVID-19 pandemic, reporting one of the highest mortality rates in the world [1]. Our hospital, Ziekenhuis Oost-Limburg (ZOL), is situated in Limburg, which was the epicenter of the COVID-19 crisis in Belgium, reporting the highest infection rate of all Belgian provinces [2]. National guidelines concerning admission criteria on ICU of patients with a COVID-19 pneumonia were lacking. This is a single center retrospective analysis of patients that would be have been excluded from ICU according to the NHS COVID-19 decision support tool [3]. All patients admitted to the ICU with a confirmed COVID-19 pneumonitis from March to June 2020 were included in the analysis.

**Methods**: Categorical data were represented as numbers and percentages. The distribution of continuous data was analyzed and represented as either mean ± SD or median and IQR. No data imputation was done as for all variables the missings were less than 15%. All p values were two-sided and considered significant when < 0.05. All analyses were performed with JMPsoftware version 15.0.0 (SAS Institute, Cary, NC, USA).

**Results**: Ninety-seven patients were admitted to the ICU. Mean APACHE III was 67(n16), with a predicted ICU mortality of 30%. The ICU mortality rate was 20.6% (n20). 44% (n44) patients would not be admitted to the ICU according to the NHS guidelines. The mortality in this group was significantly higher 38% (n17).

**Conclusions**: Although mortality is significantly higher; 28% of our population (n27) would not be admitted to the ICU according to the NHS COVID-19 decision support tool; although they did survive. Follow-up of these patients is planned.

**References**:

1. How well have OECD countries responded to the coronavirus crisis? The Economist Intelligence Unit,@TheEIU, June 17, 2020
2. Epidemiological situation of the coronavirus in Belgium: daily report of national and international situation. Sciensano. Accessed June 21, 2020.
3. National Institute for Health and Care Excellence. COVID‐19 Rapid Guideline: Critical Care 2020.

## P215

### Factors associated with survival after intensive care unit (ICU) admission due to coronavirus disease. Preliminary results of a newly established ICU

#### A. Flevari^1^, M. Karagianni^2^, S. Giannakaki^2^, A. Aiginitou^2^, S. Patsilinakou^2^, E. Kourtelesi^2^, E. Bourgani^2^, C. Papadas^2^, C. Merkouri^2^, M. Daganou^2^

##### ^1^ICU, General Hospital of Thoracic Diseases "Sotiria", Athens, Greece; ^2^General Hospital of Thoracic Diseases "Sotiria", Athens, Greece

*Critical Care* 2021, **25**(**Suppl 1**): P215

**Introduction**: Newly established intensive care unit (ICU) departments in the era of COVID-19 pandemic outbreak were forced to adapt and find pace. We recorded the epidemiologic characteristics of our cohort of patients in order to determine which factors, if any, could have a positive effect on ICU survival.

**Methods**: This prospective observational study was conducted in a 13-bed capacity ICU. On admission age, gender, preexisting comorbidities (arterial hypertention, cardiovascular disease, diabetes mellitus, morbid obesity, pulmonary disease), and disease severity scores APACHE II and SOFA were recorded. Duration of stay and final outcome were also noted. A Kolmogorov–Smirnov test checked normality. An unpaired student T-test was performed for continuous variables. Analysis of variance (Anova) tested for differences in the means between categorical and continuous variables.

**Results**: Between December 24, 2020, and May 28, 2021, 64 patients (70.3% male) with median age 64.5 years were ICU admitted. 51 patients (79.6%) were under invasive mechanical ventilation and 13 patients were on HFNC. 14 patients died (21.8%). Survivors in relation to non-survivors were male (p = 0.028), younger (p = 0.000) and had lower APACHE II score (p = 0.030). We investigated whether age, number of comorbidites, obesity, duration of ICU stay, and severity scores could predict patients’ outcome. The model explained 98% of the variance and it was a significant predictor of outcome [F(6,3) = 27.99, p = 0.01]. While obesity (B = -0.43, p < 0.039), SOFA (B = -0.88, p = 0.04) and APACHE II scores (B = 1.13, p = 0.004) contributed significantly to survival, age (p = 0.09) total comorbidities (p = 0.07) and duration of ICU stay (p = 0.14) did not.

**Conclusions**: Nonobese patients and low SOFA scores had better outcome in this cohort of patients. Age per se should not be a part of the triage procedure. These results warrant further investigation and should be used cautiously to guide difficult clinical decisions.

## P216

### Six-month outcome in COVID-19 critical care patients: preliminary results from a follow-up study

#### L. Moretto^1^, V. Bellin^2^, F. Bettini^2^, V. Meroni^2^, S. Sosio^2^, M. Andreossi^2^, A. Annoni^2^, M. Villa^2^, A. Lucchini^2^, G. Foti^2^

##### ^1^Emergency Department, University of Milano-Bicocca, Milan, Italy; ^2^Emergency Department, ASST Monza-San Gerardo Hospital, Monza, Italy

*Critical Care* 2021, **25**(**Suppl 1**): P216

**Introduction**: Outcome of ICU COVID-19 patients is not completely known.

**Methods**: In our prospective observational study, we describe 6-month outcome in a cohort of COVID-19 ICU survivors. We enrolled consecutive ICU subjects with COVID-19 pneumonia, mechanically ventilated for > 72 h. Six months after hospital discharge, patients were evaluated to identify Post-intensive Care Syndrome (PICS) [1]. The diagnosis was established when at least one of the following criteria was fulfilled: one positive functional or strength test, one positive cognitive test or one positive psychological score. Respiratory impairment was defined as FEV1 or a Six-Minute-Walking-Test distance walked < 80% of predicted value, while Euroquality Five Levels Five Dimensions (HQ5D) score and Thirty-six-Item Short Form Survey (SF-36) were used to assess perceived quality of life (QOL).

**Results**: From March to December 2020, we enrolled 168 patients, 4 died after discharge and 18 were lost to follow-up. These are the results from the first 62 patients evaluated. Median age was 61 (IQR 11), 48 (77%) were males. PICS was seen in 55 cases (89%) and 50 (80%) of them experienced an impairment in only one domain; psychological symptoms were detected in 100% of patients with PICS; Fig. 1 shows impairments co-occurrence. Twenty-six patients (42%) had a respiratory disfunction, but no association was found with PICS. No difference in age, sex and pre-admission BMI and Clinical Frailty score was detected between PICS and non-PICS groups. HQ5D-VAS > 70% in 81% of patients and high percentages in almost all SF36 fields testified a good perceived QOL; neither PICS nor respiratory alterations were associated with a decrease in quality of life.

**Conclusions**: Most ICU COVID-19 survivors suffer from PICS at six months. Respiratory impairment is frequent, but not associated with PICS development. However, a good level of QOL was reported. More data are needed to define COVID-19 ICU survivors’ outcome.

**Reference**

1. Needham DM et al. Crit Care Med 40: 502–509, 2012

**Figure:**
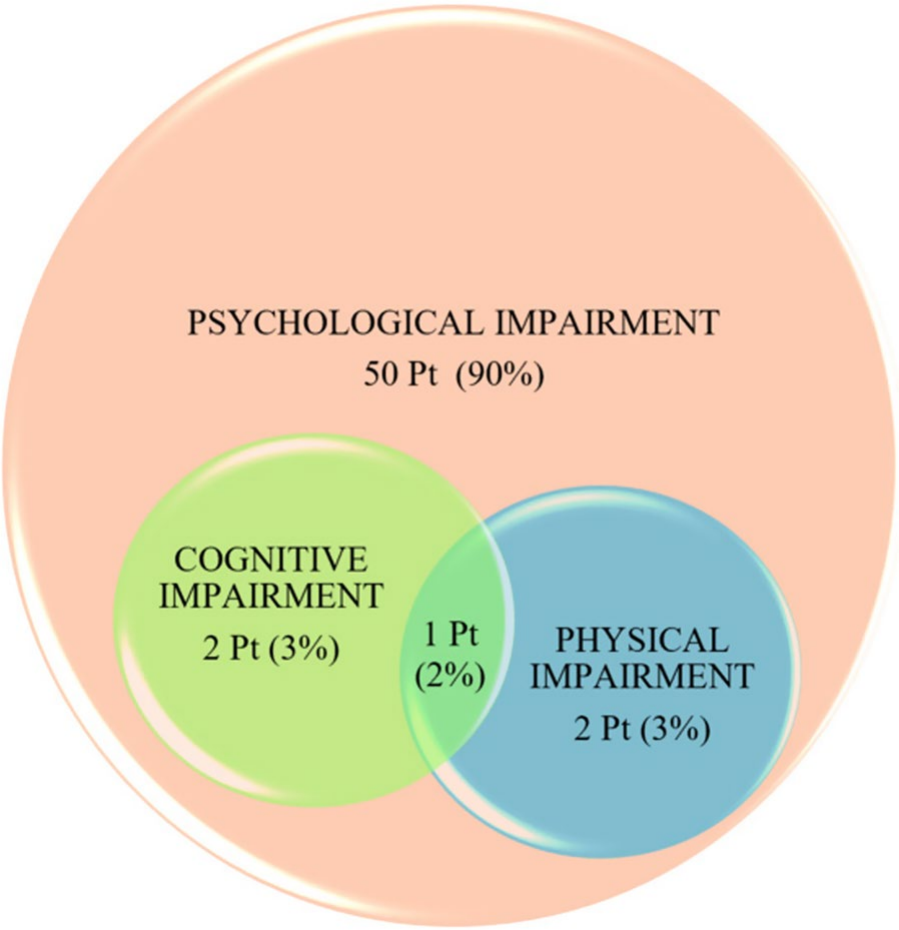
**Fig. 1** **(abstract P216)** PICS impairment co-occurrence. Pt: patients; precentages refer to frequency of each component of PICS (psychological, functional and cognitive impairment) and their concurrency in PICS patients

## P217

### Psychosocial impact of COVID-19 pandemic on ICU personnel at a tertiary care center in the USA

#### N. C. Watson^1^, S. J. Pounders^2^, L. A. Krech^3^, A. J. Chapman^4^, M. M. Lypka^5^, M. H. Armstrong^1^, C. J. Gibson^6^, K. A. Kelly Schuette^7^, G. A. Iskander^6^

##### ^1^Anesthesiology, Anesthesia Practice Consultants, Grand Rapids, USA; ^2^Trauma Research Institute & Office of Research, Spectrum Health, Grand Rapids, USA; ^3^Trauma Research Institute, Spectrum Health, Grand Rapids, USA; ^4^Trauma Research Institute & Acute Care Surgery and General Surgery, Spectrum Health, Grand Rapids, USA; ^5^Office of Research, Spectrum Health, Grand Rapids, USA; ^6^Acute Care Surgery and General Surgery, Spectrum Health, Grand Rapids, USA; ^7^General Surgery Residency, Spectrum Health, Grand Rapids, USA

*Critical Care* 2021, **25**(**Suppl 1**): P217

**Introduction**: The primary objective of this study was to assess the psychosocial impact of the COVID-19 pandemic on ICU health care workers (HCW). Previous global pandemics, such as SARS, have demonstrated increased stress on HCW leading to anxiety, depression, isolation and exhaustion.

**Methods**: A longitudinal survey design was used. From April 2020 to July 2021 ICU HCW received two scheduled anonymous surveys. The Work-Related Stress Survey (WRSS) was administered every 4 weeks and the Professional Quality of Life Measure (ProQOL) was administered initially, at peak COVID-19 patient census, and at 3 months post-peak. The WRSS was comprised of elements from published HCW stress questionnaires. The ProQOL is a validated instrument for assessing compassion satisfaction (CS), burn out (BO), and secondary traumatic stress (STS) [1]. ProQOL t-scores were grouped into low/moderate/high.

**Results**: The WRSS demonstrated that anxiety, mental exhaustion, and feelings of isolation were maximum at peak census and lower at other times. Levels of CS, BO, and STS were high across the study period (Fig. 1).

**Conclusions**: HCW demonstrated stress levels that were temporally associated with COVID-19 patient census. > 70% of ICU HCW showed high levels of BO and STS throughout the pandemic while also maintaining a high level of CS. There is an urgent need to address stress in ICU HCW.

**Reference**

1. The Center for Victims of Torture: www.ProQOL.org Accessed 31/5/21

**Figure:**
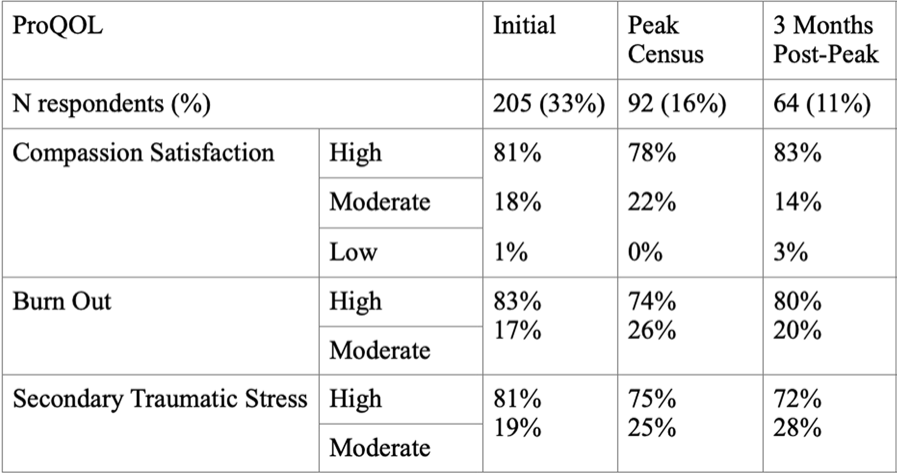
**Fig. 1** **(abstract P217)** ProQOL results (percentage of respondents) for sub-categories at study initiation, peak ICU COVID-19 patient census, and 3 months post-peak. Burn out and secondary traumatic stress had zero respondents with low scores

## P218

### Assessing adherence to COVID-19 guidelines during wave 2 at Europe’s largest single-floor intensive care unit

#### C. Irwin, D. A. Ahmed

##### College of Medical and Dental Sciences, University of Birmingham, Birmingham, UK

*Critical Care* 2021, **25**(**Suppl 1**): P218

**Introduction**: As COVID-19 was a new disease, there was a lack of formal protocol available guiding management earlier in the pandemic. This led to rapid development of guidelines by the University Hospitals Birmingham COVID-19 Working Group for the immediate management of severe respiratory disease in September 2020. We audited the adherence of the Queen Elizabeth Hospital’s (QE) intensive care units (ICUs) to these guidelines during wave 2.

**Methods**: Adherence targets were set at 90% for three core guideline aspects: 7–10 days of dexamethasone administration, lung protective ventilation (LPV), and microbiology investigations (HIV, sputum culture, urinary legionella). This was a retrospective audit, with data collected from patients admitted to QE ICUs between September to November 2020 the electronic Prescribing Information and Communications System (PICS) system. Data was analyzed using Excel.

**Results**: A total of 88 patients were included. The only investigation that met the 90% target was HIV testing (100%). Sputum culture, urinary legionella and influenza swab did not meet the target (50%, 16% and 8% respectively). 83% (n = 73) of patients received dexamethasone for 7–10 days. Overall LPV adherence was 72%, with significant variation between sub-parameters (Fig. 1).

**Conclusions**: Only a subsection of the investigations aspect of this guideline (HIV tests, bloods, imaging) met our targets. The remaining microbiology investigations, dexamethasone therapy, and LPV compliance fell short. One contributing factor we identified was the lack of staff familiarization with the environment and clinical system. We produced a set of recommendations to minimise the impact of these human factors on guideline adherence.

**Figure:**
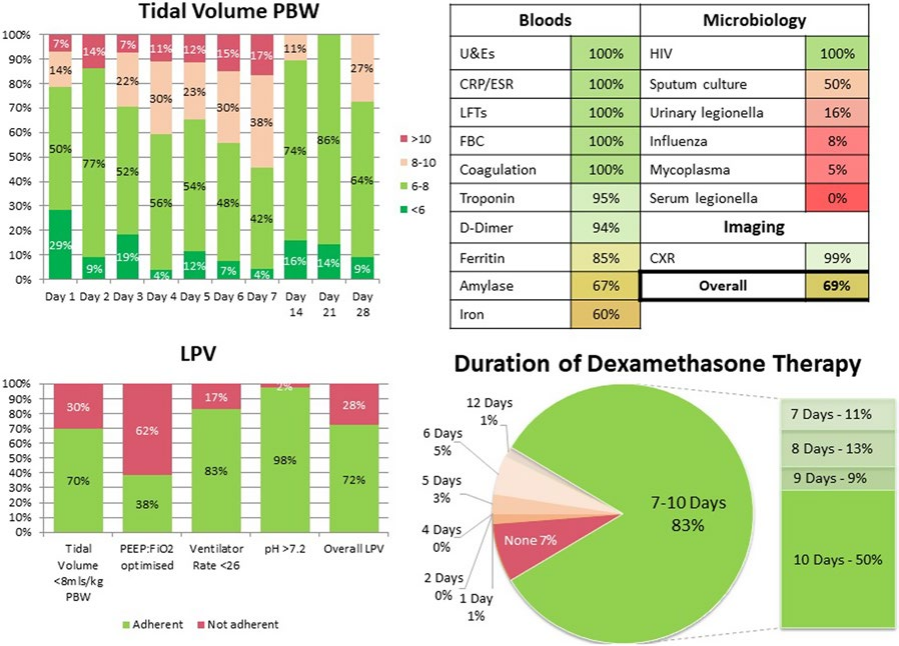
**Fig. 1** **(abstract P219)** Results from audited patients

## P219

### Peripheral nerve injuries 3 months after a critical COVID-19 pneumonia

#### C. Malengreaux^1^, P. Minguet^1^, C. Colson^1^, N. Dardenne^2^, B. Misset^1^, A. F. Rousseau^1^

##### ^1^Intensive Care, CHU de Liège, Liège, Belgium; ^2^Biostatistic Unit, CHU de Liège, Liège, Belgium

*Critical Care* 2021, **25**(**Suppl 1**): P219

**Introduction**: Peripheral nerve injuries (PNI) have been associated with prone positioning (PP) in mechanically ventilated (MV) patients with COVID-19 pneumonia. The aims of this retrospective study were to assess PNI prevalence three months (M3) after intensive care unit (ICU) discharge, and to search for some risk factors.

**Methods**: During the first and second waves of the pandemic, all COVID-19 adults who survived an ICU stay ≥ 7 days were included if they attended the M3 consultation at our follow-up clinic. PNI referred to limb weakness, pain, hypoesthesia or paresthesia. Data about demographics, medical history, ICU stay (illness severity, duration of MV, PP, neuromuscular blocking agents and insulin; corticosteroids, peak of glycemia, creatine kinase and C-reactive protein) and M3 outcomes (autonomy using Barthel index, quality of life (QOL) using EQ-5D scale) were recorded. Data are expressed as median (Q1-Q3).

**Results**: Fifyt-six patients (age 62 (55–71)y, all obese, SAPS II 35.62 (16–82)) survived an ICU stay of 20 (3–64)d for COVID-19 pneumonia and were followed at M3. From them, 75% (42/56) received MV during 17 (11–25)d, and 55.4% (31/56) patients had at least one PP session. At M3, 41.1% (23/56) declared motor and/or sensitive symptoms of PNI, located mainly at feet, thighs, hands and arms. PNI significantly impacted Barthel index: 100 (70–100) vs 100 (90–100) in survivors with and without PNI, respectively (p = 0.013). Their QOL was similar. After multivariate binary logistic regression with stepwise selection, MV duration was significantly associated with PNI (p = 0.037), independently of a wave effect. The other parameters were not associated with PNI.

**Conclusions**: In this cohort, at least 40% of critical COVID-19 survivors presented PNI 3 months after ICU discharge, associated with a significant alteration of autonomy. Only MV duration was observed as a potential independent risk factor. A larger cohort is needed to complete these preliminary results.

## P220

### Correlation of persisting post-discharge symptoms in patients hospitalized for COVID-19 pneumonia with severity of the disease

#### P. M. Mavroudis^1^, L. V. Velentza^1^, A. S. Stamati^1^, P. D. Dermatis^2^, A. G. Georgaki^3^, O. Z. Zarkotou^3^, C. S. Sidiropoulou^1^, K. T. Themeli-Digalaki^3^, E. G. Giannitsioti^1^, S. G. Gerakari^1^

##### ^1^COVID-19 Department, Tzaneio General Hospital, Piraeus, Greece; ^2^Emergency Department, Tzaneio General Hospital, Piraeus, Greece; ^3^Microbiology Department, Tzaneio General Hospital, Piraeus, Greece

*Critical Care* 2021, **25**(**Suppl 1**): P220

**Introduction**: Post-discharge sequela in patients hospitalized with COVID-19 disease are under investigation. Factors potentially associated with persistent post-COVID-19 symptoms were explored in patients hospitalized in COVID-19 medical wards and/or ICU.

**Methods**: Patients with confirmed SARS-CoV-2 disease (severe and non-severe) hospitalized and successfully discharged at home were evaluated for persisting clinical symptoms and performed lung spirometry, lung CT scan and echocardiography. Blood sampling for IgG SARS-CoV-2 detection by the LIAISON® SARS-CoV-2 S1/S2 IgG assay was done. Factors influencing persisting symptoms for more than one month after the patients’ discharge were analyzed by descriptive statistics and ROC curve for serum antibody titers and severity of the infection.

**Results**: Among 45 patients, men (n = 30, 66.7%) with mean age (± SD) 59.62 (10.25) years and mean BMI (± SD) 30.62 (4.41) kg/m^2^ presented after a mean post-discharge time of 71.11 days, COVID-19 was severe in 20% of cases. Comorbidities (62.2%) included hypertension (n = 15), dyslipidemia (n = 13), diabetes mellitus (n = 6). Echocardiography revealed mild (n = 28) or moderate (n = 2) diastolic dysfunction, being normal in 9 patients. In 35% of CT scans fibrotic lesions were detected. Lung spirometry was abnormal in 21/36 patients (58.3%) more often in those with persisting symptoms > 1 month after discharge (p = 0.05): fatigue (90.3%), dyspnea (38.7%), cough (38.7%), myalgias/arthralgias (35.5%), loss of smell/taste (35.5%) and dizziness (19.4%). Mean (± SD) IgG SARS-CoV 2 titer was 234.42 (130.10) AU/ml whilst most seriously ill patients during hospitalization present the highest titer [ROC AUC 0.787 (95%CI 0.657–0.918)].

**Conclusions**: More than 50% of patients presented with persisting symptoms -predominantly fatigue- for > 1 month post-discharge and abnormal spirometry even if only 20% of patients had severe disease during hospitalization which correlated with higher post-discharge SARS-COV-2 IgG titers.

## P221

### Impact of coronavirus 19 pandemic on children after their discharge from pediatric intensive care

#### L. B. Barcat, G. D. Du Pont-Thibodeau, C. J. Jutras, K. H. Harrington, L. D. Ducharme-Crevier

##### Pediatric Intensive Care Unit, CHU Sainte Justine, Montreal, Canada

*Critical Care* 2021, **25**(**Suppl 1**): P221

**Introduction**: The novel coronavirus disease 2019 (COVID-19) pandemic has disrupted the life of many families, especially those of children with chronic health needs [1]. Little is known about the health and well-being of critically ill children and their families after their discharge from the pediatric intensive care unit (PICU) during the COVID-19 pandemic. We are describing the repercussions of the COVID-19 lockdown on the physical and psychological wellbeing of PICU survivors and their families, their quality of life and their accessibility to medical resources and extrafamilial support.

**Methods**: We conducted a prospective cohort study of children and families followed at the Centre Hospitalier Universitaire Ste-Justine Pediatric Intensive Care Unit (PICU) follow-up clinic from October 2018 to February 2020. Patients and parents were contacted by phone by a pediatric intensivist to fill out validated questionnaires (Pediatric Quality of Life Inventory, Hospital Anxiety and Depression Scale) and to measure the impact of the COVID-19 pandemic on access to medical care and extrafamilial support.

**Results**: Fifty-five families were contacted between November and December 2020. Quality of life scores were 88.1 ± 16.9 and 83.8 ± 13.9 for physical and psychosocial aspects, respectively. Symptoms of anxiety and depression were detected in 23.6% and 3.6% of respondent parents, respectively. A significant proportion of families reported cancelled health appointment (65.5%) and medication access issues (12.7%). Twenty-five families (45.5%) reported a significant decrease in their familial financial incomes.

**Conclusions**: PICU survivors and their families experience significant impact on medical care and familial organization during the COVID-19 pandemic.

References:

1. Cacioppo M et al 2021 Ann Phys Rehabil Med 64:101429, 2021.

## P222

### Limitation of life-sustaining treatment in critically ill patients admitted in ICU with SARS-CoV-2 pneumonia

#### A. Estella^1^, M. Recuerda^2^, A. Garrino^3^, P. Bustelo^3^, C. De La Fuente^4^, J. L. García Garmendia^5^, J. Garnacho-Montero^6^, S Group of work Infectious diseases Andalusian Society of Intensive Care SAMIUC^7^

##### ^1^Intensive Care Unit, University Hospital of Jerez, INiBICA Medicine Department Cadiz University, Intensive & Critical Care, Cadiz, Spain; ^2^Intensive Care Unit University Hospital of Puerto Real, Puerto Real, Spain; ^3^Intensive Care Unit, University Hospital of Jerez, Jerez, Spain; ^4^Intensive Care Unit, University Hospital Reina Sofia of Córdoba, Córdoba, Spain; ^5^Intensive Care Unit, Hospital San Juan de Dios Bormujos, Bormujos, Sevilla, Spain; ^6^Intensive Care Unit, University Hospital Virgen Macarena Sevilla, Sevilla, Spain; ^7^Intensive Care Unit, University Hospital of Jerez, INiBICA Medicine Department Cadiz University, SAMIUC, Cadiz, Spain

*Critical Care* 2021, **25**(**Suppl 1**): P222

**Introduction**: Limitation of life-sustaining treatment (LLST) is a recommended practice to avoid futility in critically ill patients who do not improve the medical condition despite invasive treatment. In SARS-CoV-2, LLST during ICU admission has been scarcely studied. Objective: To describe clinical characteristics of critically ill patients with severe SARS-CoV-2 pneumonia with a clinical decisión of LLST during ICU admission.

**Methods**: Prospective descriptive multicenter cohort study in 26 Intensive care units (ICU) from Andalusian region in Spain. Consecutive critically ill patients with confirmed SARS-CoV-2 infection were included from March 8 to May 30. Variables analyzed were demographic, severity scores, clinical condition and support therapy. Statistical analysis: Data were analyzed by IBM SPSS 24 and quantitative variables were expressed as a mean ± standard deviation.

**Results**: A total of 474 consecutive patients were included. Clinical decisión of LLST was recorded in 38 patients, 8.01%. Comorbidities were more frequent in the group of LLST, cardiovascular diseases: 50% versus 37.1%; renal disease: 21.1% versus 8.4%; hypertension: 60.5% versus 45.2%; neurological diseases: 21.1% versus 6.3% and cirrhosis disease: 5.3% versus 1.8%. There were not differences in malignancy and chronic respiratory disease between groups. Percentage of tracheostomy (30.5% Non LLST vs 27%) and hemofiltration (10.4% Non LLST vs 10.5%) was similar. Table 1 shows comparison according clinical decisión of LLST.

**Conclusions**: Age, comorbidities and severity of organ failure at ICU admission were associated with LLST decision during ICU stay.

**Table:** **Table 1** **(abstract P222)** Comparison according to clinical decision of LLST

SARS CoV-2 pneumonia	Patients without LLST n: 436	Patients with LLST n: 38
Age (years)	60.7 ± 11.9	70.3 ± 8.86
APACHE II at admission	12.5 ± 7.3	17.67 ± 8.1
SOFA at admission	5.47 ± 3	7.24 ± 2.74
SOFA 72 Hours	6.23 ± 3	8.41 ± 3.36
PaO2/FiO2 at admission	140.11 ± 78.1	140.92 ± 85.5
ICU lenght of stay (days)	16.02 ± 11.6	10.66 ± 10.5
Gender (male/female)	315/121	29/9

